# An *endo*‐Directing‐Group Strategy Unlocks Enantioselective (3+1+2) Carbonylative Cycloadditions of Aminocyclopropanes

**DOI:** 10.1002/anie.202205007

**Published:** 2022-06-24

**Authors:** Olga O. Sokolova, John F. Bower

**Affiliations:** ^1^ School of Chemistry University of Bristol Bristol BS8 1TS UK; ^2^ Department of Chemistry University of Liverpool Crown Street Liverpool L69 7ZD UK

**Keywords:** Cycloaddition, Cyclopropanes, Enantioselectivity, Rhodium, Synthetic Methods

## Abstract

An *endo*‐directing group strategy enables enantioselective (3+1+2) cycloadditions that are triggered by carbonylative C−C bond activation of cyclopropanes. These processes are rare examples of cycloadditions where C−C bond oxidative addition is enantiodetermining, and the first where this is achieved within the context of a multicomponent (higher order) reaction design.

## Graphical abstract

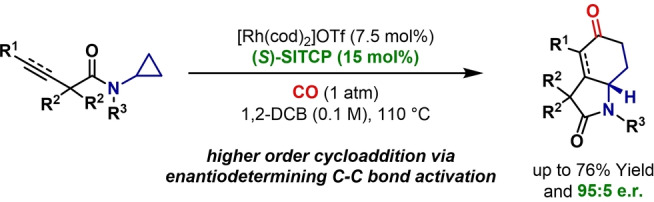

Enantioselective cycloadditions triggered by oxidative insertion of transition metals into C−C bonds (termed here as “C−C bond activation”) are an emerging class of process for the byproduct‐free construction of complex ring systems.^[1]^ Typically, strained rings are used to facilitate the C−C bond activation process, and a subsequent step, usually π‐insertion, is enantiodetermining [Scheme 1A, Eq. (1)].^[2]^ By contrast, processes where the C−C bond activation step is enantiodetermining are much rarer [Scheme 1A, Eq. (2)]. The groups of Cramer and Dong have developed (4+2)^[3]^ and related 2‐component cycloadditions[^4^, ^5^] that are triggered by enantiodetermining C−C bond activation of cyclobutanones. Enantiodetermining C−C bond activations of cyclopropanes are limited to Ye's Ni‐catalyzed (3+2) cycloadditions.^[6]^ To the best of our knowledge there are currently no examples of multicomponent (higher order) cycloadditions that involve enantiodetermining C−C bond activation.[^1l^, ^7^]

**Scheme 1:**
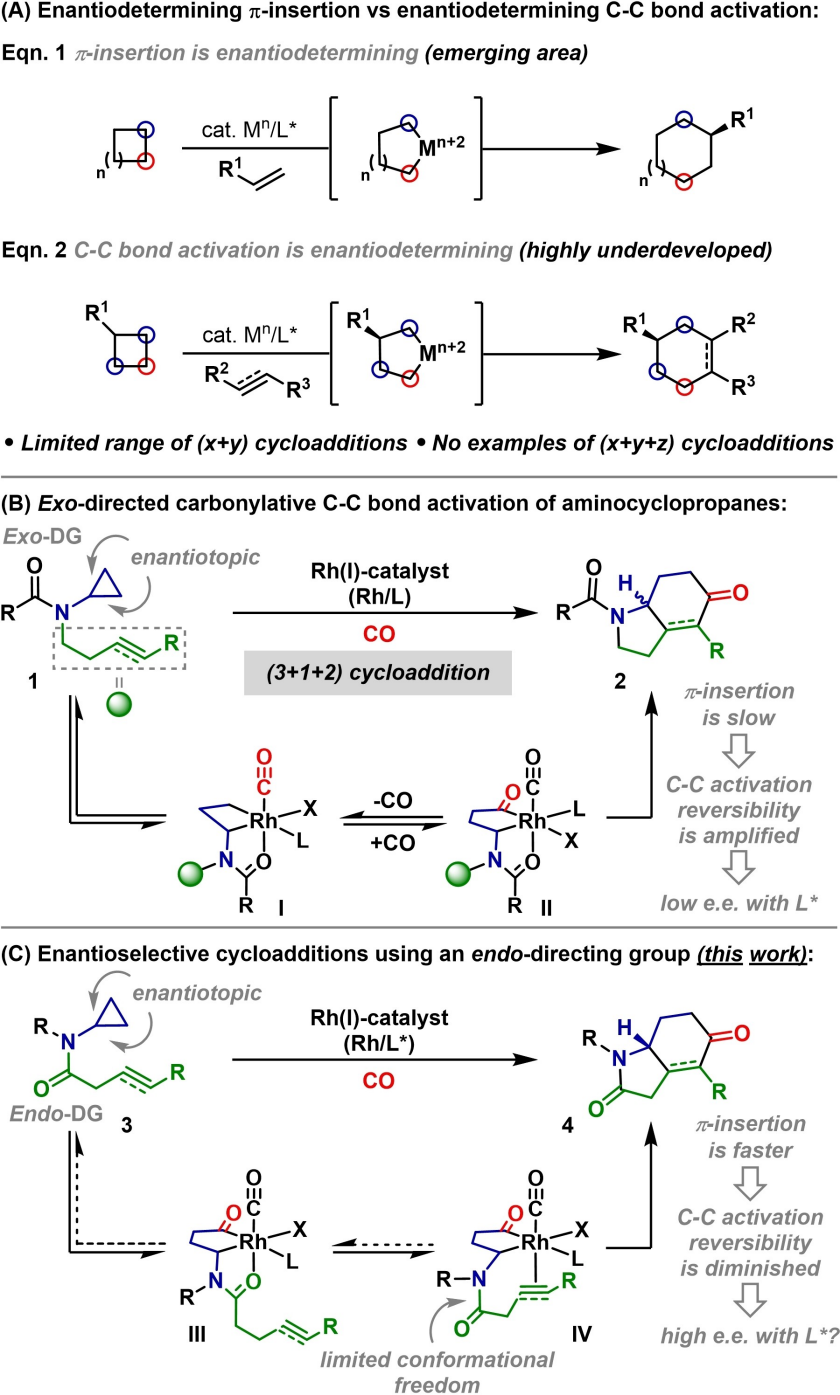
Introduction.

We have previously reported a series of processes that are triggered by directed carbonylative C−C bond activation of simple “non‐activated” cyclopropanes.^[8]^ Amongst these, aminocyclopropane‐based systems have proven to be especially versatile, enabling (3+1+2) cycloadditions (**1** to **2**),^[9]^ as well as a range of heterocyclization processes (Scheme 1B).[^10^, ^11^] To date, and despite a decade of intense efforts, enantioselective variants have remained elusive. As evidenced by stoichiometric studies, carbonylative C−C bond activation (**1** to **I** to **II**) is reversible, using either cationic (X=e.g. OTf)^[9b]^ or neutral (X=e.g. Cl) Rh‐precatalysts.^[12]^ This reversibility provides a major impediment to enantioselective variants because it is expected to erode any kinetic selectivity that chiral ligands might impart during C−C bond activation. To address this, we considered replacing the *exo*‐directing groups used in our previous (3+1+2) cycloadditions with an *endo*‐directing group (**3** to **4**, Scheme 1C). In this new design, the conformational constraints imposed by the amide directing group should enhance access to π‐complex **IV**, and, in turn, accelerate the formation of **4**. Accordingly, reversion to cyclopropane **3** should be suppressed, and enantioselectivity achieved during the C−C bond activation step should be transferred to the product with higher fidelity. In this study, we outline the successful realization of this idea, which has led to the first examples of processes involving enantioselective carbonylative C−C bond activation of cyclopropanes. In broader terms, these studies represent the first examples of multicomponent (higher order) cycloadditions that involve enantiodetermining C−C bond activation.^[13]^

Efforts to develop the processes envisaged in Scheme 1C commenced with examining the carbonylative cycloaddition of alkynyl system **3 a** (Table 1). Using a balloon pressure (1 atm) of CO,^[14]^ we found that [Rh(cod)_2_]OTf/PPh_3_ is an effective system, and this delivered target **4 a** in 77 % yield at 130 °C in 1,2‐DCB (0.1 M). Neutral Rh‐precatalysts (e.g. [Rh(cod)Cl]_2_) or those possessing strongly dissociating counterions (e.g. BARF) were not suitable, and more strongly coordinating solvents (e.g. PhCN) resulted in lower yields. Efforts to render the process enantioselective focused on evaluating a wide range of commercially available chiral ligands, from which selected results are outlined in Table 1 (further details are given in the Supporting Information). Bidentate ligands (e.g. **L‐1**–**L‐3**) were inefficient, leading to low yields and minimal enantioinduction. Monodentate phosphoramidate ligands (e.g. **L‐4**–**L‐6**) offered marginal improvements in yield, although enantioselectivity remained poor. Ultimately, we uncovered a significant electronic trend with respect to the P‐center, wherein electron rich phosphine **L‐9** delivered **4 a** in good yield and enantioselectivity (93 : 7 e.r.). More electron poor phosphite and phosphonite ligands **L‐7** and **L‐8** were significantly less effective. The promising result with **L‐9** was compromised by competing oxidation of the cyclohexenone ring of **4 a** to the corresponding phenol (21 % yield, not depicted). To suppress this, further optimization was conducted, resulting in the lower temperature protocol outlined in Table 2, which affords **4 a** as the sole product in 76 % yield and 95 : 5 e.r. During these studies, we confirmed that precatalysts with more dissociating counterions are less effective; for example, use of [Rh(cod)_2_]BF_4_ gave **4 a** in 47 % yield and 70 : 30 e.r. (see the Supporting Information for details). A 1 : 2 ratio of Rh : L* is optimal, but similar efficiencies can be achieved with lower ligand loadings; for example, use of 7.5 mol % **L‐9** under the conditions shown in Table 2 gave **4 a** in 68 % yield and 91 : 9 e.r. This observation suggests that only one phosphine ligand is required on the Rh‐center during the enantiodetermining step (cf. Scheme 1B, **1** to **I**).^[15]^

**Table 1:**
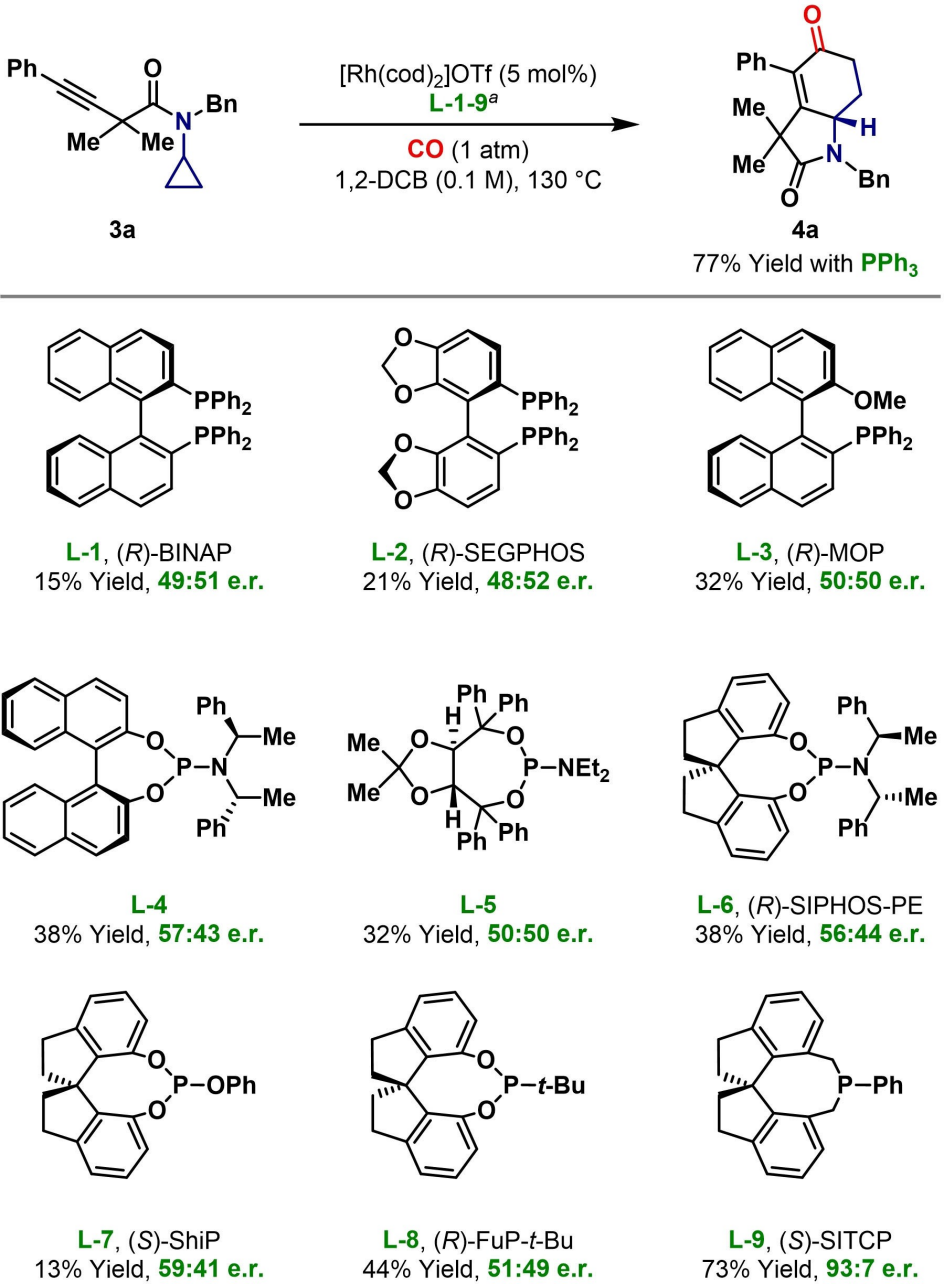
Optimization of the (3+1+2) cycloaddition process.

[a] Bidentate ligands: 5 mol %, monodentate ligands: 10 mol %.

**Table 2:**
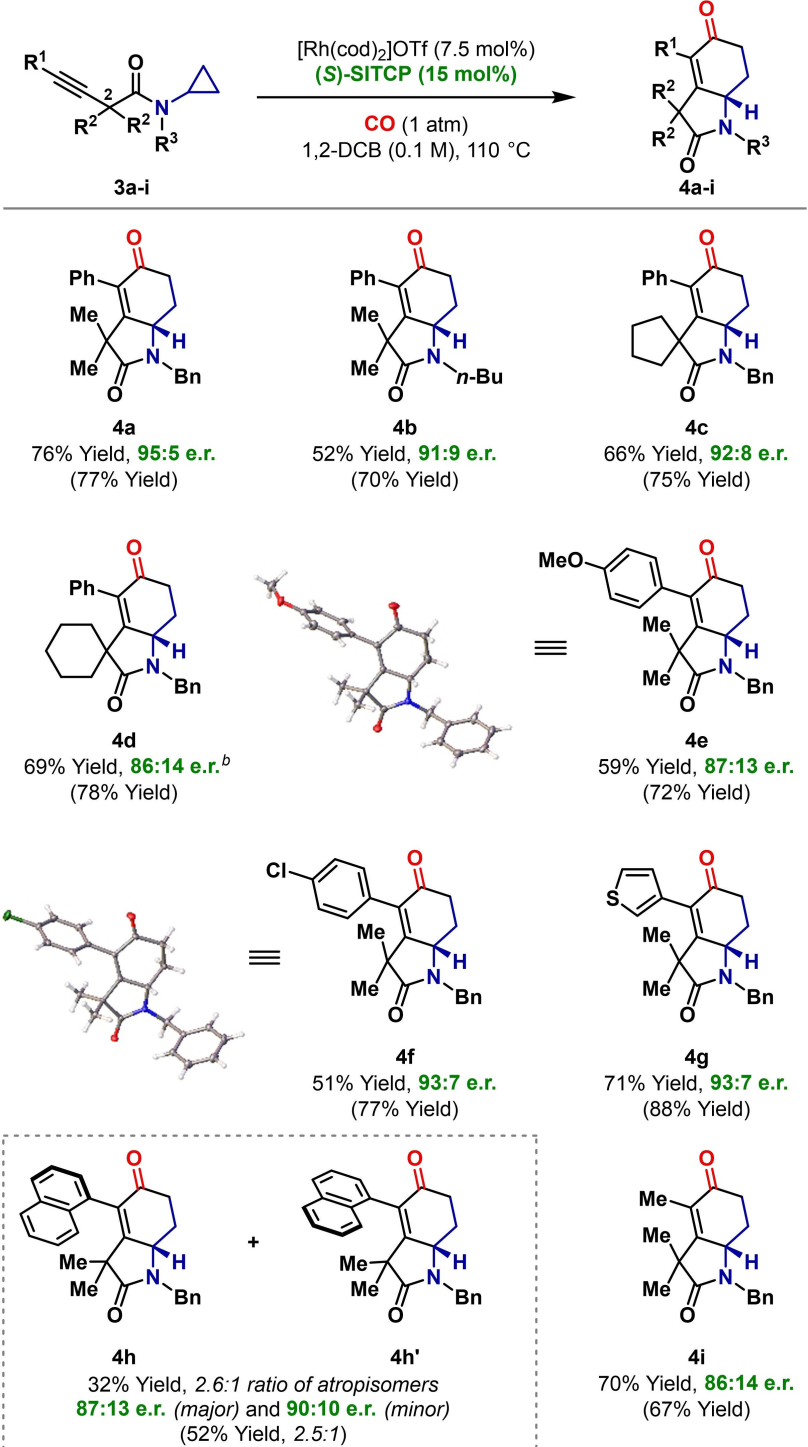
Enantioselective (3+1+2) cycloadditions of internal alkynes.^[a]^

[a] Yields using optimized non‐enantioselective conditions ([Rh(cod)_2_]OTf (5 mol %), PPh_3_ (10 mol %), CO (1 atm), 1,2‐DCB (0.1 M), 130 °C) are given in parentheses. [b] The reaction was run at 120 °C.

Having established optimal conditions with **3 a**, we explored the scope of the enantioselective protocol as outlined in Table 2. Because this class of cycloaddition has not been reported previously, yields under optimized non‐enantioselective conditions (PPh_3_) are also included in parentheses for each substrate. The protocol tolerates variation at R^3^ (e.g. **4 b**) and cyclic substituents can be introduced at R^2^ (**4 c** and **4 d**). Electronically diverse alkynes are tolerated; for example, aryl and heteroaryl systems **4 f** and **4 g** were generated with similar enantioselectivities. Even **3 h**, which contains a very bulky 1‐naphthyl substituent participated; in this case, the product was formed as a 2.6 : 1 ratio of atropisomers (**4 h** and **4 h′**), as a result of hindered rotation about the naphthyl‐alkenyl C−C bond. A methyl substituted alkyne participated to provide **4 i**, thereby demonstrating the viability of using aliphatic alkynes. Systems where R^2^=H are not effective and lead to only traces of cycloaddition product (see the Supporting Information), presumably because of the relatively high acidity of the C‐2 stereocenter of the starting material.^[16]^ The structures of **4 e** and **4 f** were confirmed by single crystal X‐ray diffraction. For the latter, the heavy atom effect allowed the assignment of absolute stereochemistry, and it is on this basis that other stereochemical assignments are made.^[17]^

Terminal alkynes are not suitable for the cycloaddition described here, possibly because of inhibitory formation of Rh‐vinylidene species.^[18]^ We have found that this limitation can be addressed by instead employing TMS‐protected alkynes (Scheme 2A). Cycloaddition of **3 j** and **3 k** proceeded smoothly to deliver targets **4 j** and **4 k** in 89 : 11 and 90 : 10 e.r., respectively. Here, the TMS‐protecting group “disappears” to unveil directly a C−H bond at C2 of the targets. It is unclear at what stage protodesilylation occurs, but the proton source for this is most likely adventitious water.^[19]^ Cycloadditions with alkenes are also possible as demonstrated by the efficient conversion of **3 l** to **4 l**, which proceeded with good enantioselectivity (89 : 11 e.r.) and very high diastereoselectivity (>15 : 1 d.r.), favoring the challenging *trans*‐fused bicycle. Compared to the alkyne unit of e.g. **3 a**, the alkene of **3 l** is expected to be a weaker donor ligand^[20]^ and is also sterically distinct. The similar enantioselectivities obtained for **4 l** and **4 a** are therefore notable, and are also consistent with the idea that asymmetry is established primarily during rhodacyclopentanone formation, rather than during π‐insertion.

**Scheme 2:**
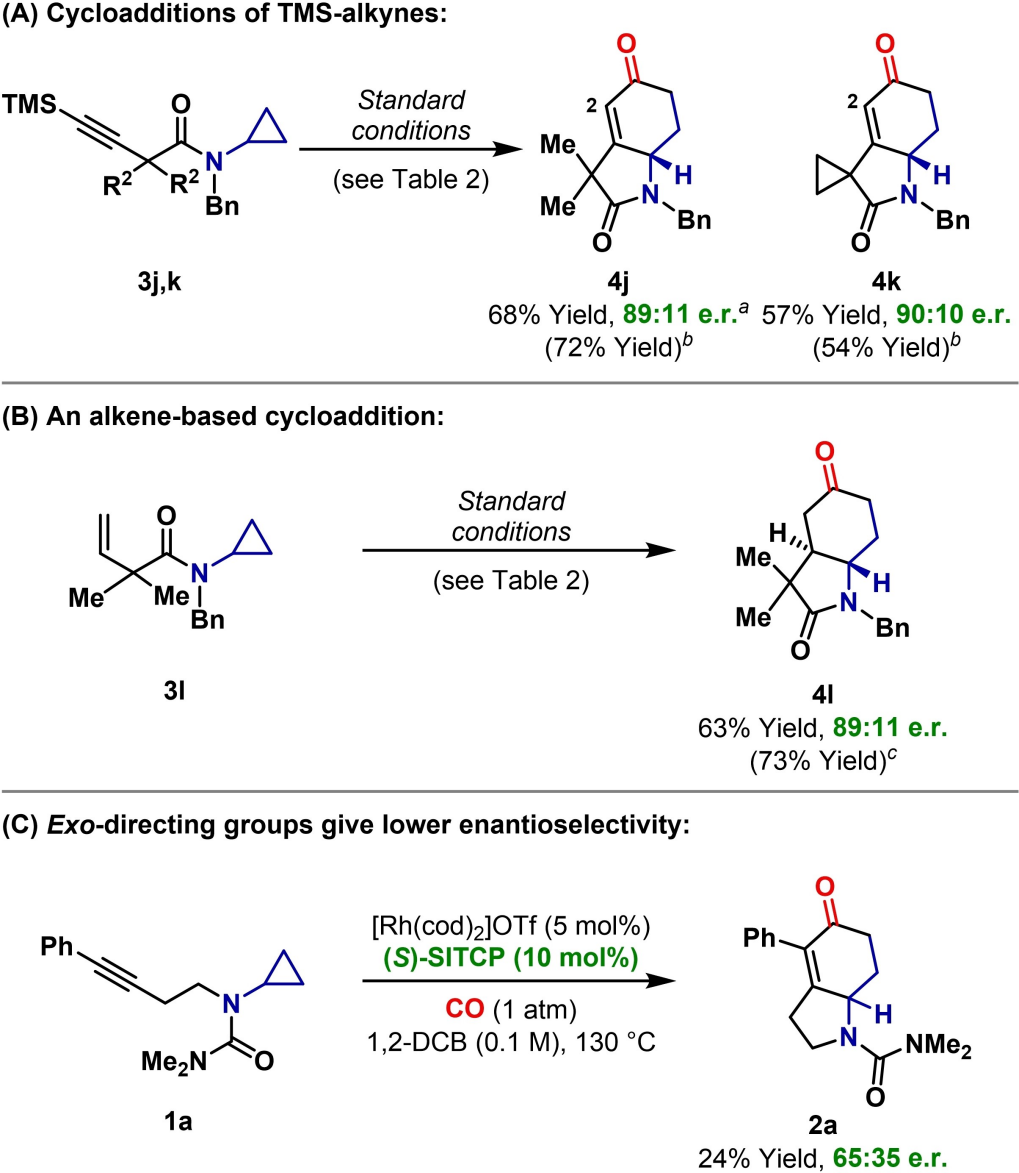
Other enantioselective (3+1+2) cycloadditions. [a] The reaction was run at 120 °C. [b] Yield using optimized non‐enantioselective conditions: [Rh(cod)_2_]OTf (5 mol %), PPh_3_ (10 mol %), CO (1 atm), 1,2‐DCB (0.1 M), 130 °C. [c] Yield using optimized non‐enantioselective conditions: [Rh(cod)Cl]_2_ (5 mol %), PPh_3_ (20 mol %), CO (1 atm), PhCN (0.1 M), 130 °C.

In further support of our reaction design, we have confirmed that the use of an *endo*‐directing group is critical; cycloaddition of system **1 a**, which possesses an *exo*‐directing group was chemically inefficient^[9a]^ and proceeded with low levels of enantioselectivity (**2 a**: 65 : 35 e.r.). Here, trapping of the rhodacyclopentanone is presumably slower, and this leads to greater reversibility for its formation, thereby eroding kinetically controlled enantioselectivity established during C−C bond activation (cf. **3 a** to **4 a**). Alternative rationalizations cannot be discounted on the basis of available data.

As shown in parentheses throughout Table 2 and Schemes 2A/B, the non‐enantioselective conditions using PPh_3_ offer very good levels of efficiency for this new *endo*‐directed (3+1+2) cycloaddition. To explore the reaction scope further, we evaluated these conditions on polysubstituted cyclopropanes, which are easy to prepare in a stereocontrolled fashion,^[21]^ but have previously proven challenging to harness in carbonylative cycloaddition processes.^[9a]^

Using PPh_3_, cycloaddition of *trans*‐1,2‐disubstituted system **3 m** was not efficient (15 % yield), and produced a 1 : 2 mixture of **4 m** : **4 m′**, derived from C−C bond activation of *bond a* vs. *bond b*, respectively (Scheme 3A). As supported by earlier stoichiometric studies,^[10a]^ rhodacyclopentanone formation via *bond a* is favored on steric grounds. Consequently, the predominant formation of **4 m′** is indicative of reversible rhodacyclopentanone formation, wherein Curtin–Hammett selectivity allows the minor C−C bond activation pathway (*bond b*) to be amplified by steps later in the cycle.^[22]^ The reason for greater reversibility in these cases (vs. Table 2) is unclear, but can be attributed to several factors, including: a) the higher reaction temperature required (150 °C vs. 110 °C) for these more demanding systems, and b) steric destabilization of the more heavily substituted rhodacyclopentanone (vs. **III**, Scheme 1C). Nevertheless, we were able to address this selectivity issue by switching PPh_3_ for P(C_6_F_5_)_3_—this allowed **3 m** (>99 % e.e.) to be converted to **4 m** (84 % yield) with complete regioselectivity and enantiospecificity (the Supporting Information details further optimization studies). Note that the process is also diastereospecific, such that the relative stereochemistry of the cyclopropane is transferred to the product. P(C_6_F_5_)_3_ is an electron poor phosphine, and has the correct properties to accelerate steps subsequent to rhodacyclopentanone formation (i.e. alkyne coordination/insertion or C−C reductive elimination), which might enforce transfer of the favored rhodacyclopentanone regioisomer (formed via *bond a*) to the target. These selective conditions transferred smoothly to **3 n** and **3 o**. The latter involves the insertion of an alkene into the rhodacyclopentanone intermediate (**III/IV**), and the high diastereoselectivity of this process enabled complete control of the C3a stereocenter.

**Scheme 3:**
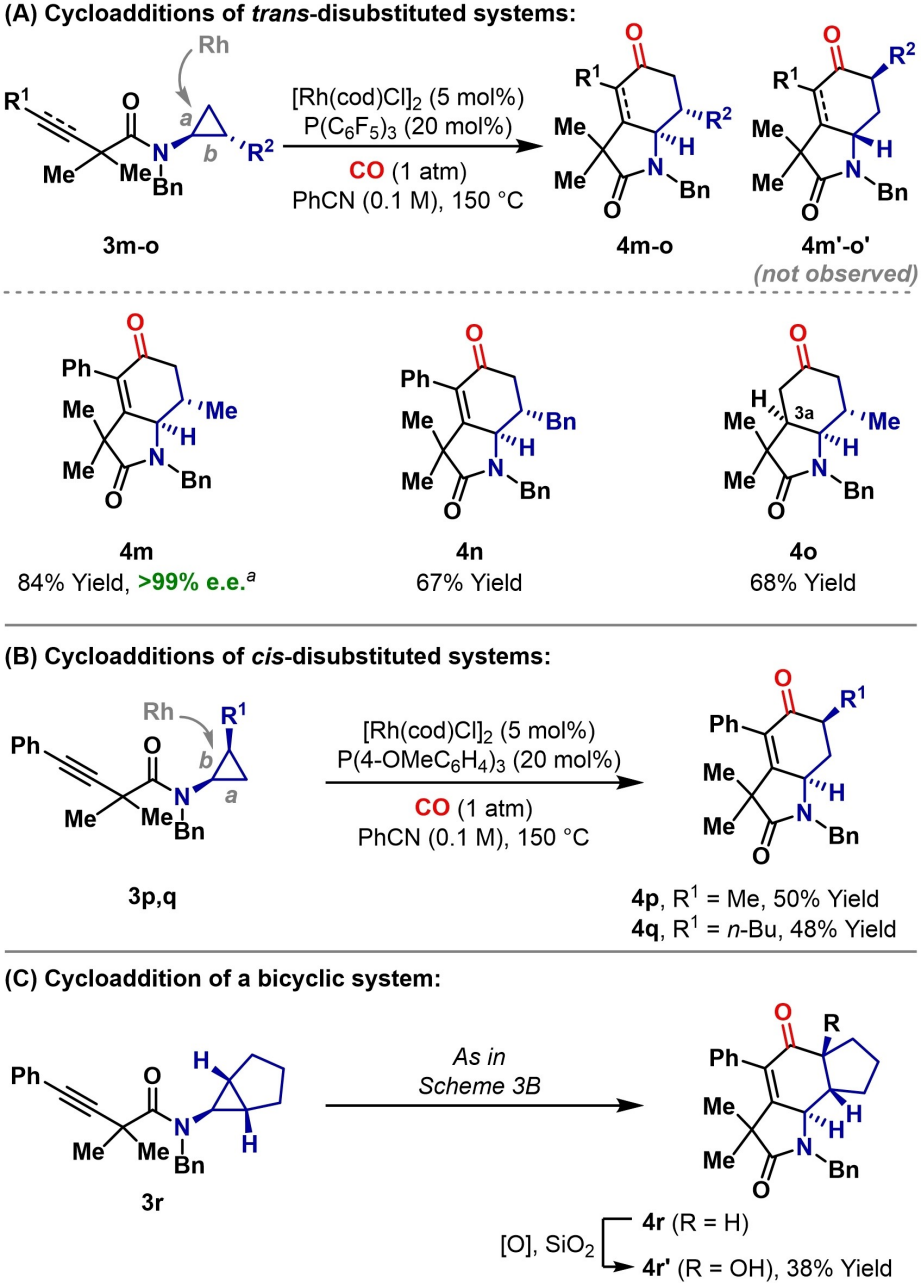
Stereospecific cycloadditions of polysubstituted cyclopropanes. [a] From enantioenriched **3 m** (>99 % e.e.).

Using PPh_3_ *, cis*‐1,2‐disubstituted system **3 p** provided **4 p**, the expected regioisomer derived from cleavage of *bond b*,^[9b]^ in 22 % yield (Scheme 3B). In this case, efficiency was improved by instead using a more electron rich phosphine [P(4‐MeOC_6_H_4_)_3_], which provided **4 p** in 50 % yield (the Supporting Information details further optimization studies). A similar result was obtained for the conversion of **3 q** to **4 q**. Finally, 1,2,3‐trisubstituted systems can be used, as evidenced by the conversion of **3 r** to **4 r** (Scheme 3C). In this case, C−C bond activation desymmetrizes the cyclopropane to provide **4 r** with complete levels of stereocontrol, as determined by ^1^H NMR analysis of crude material. Interestingly, we were unable to purify **4 r** because it rapidly underwent aerial oxidation to **4 r′** during chromatography.

In summary, we demonstrate the first examples of processes that involve the highly enantioselective carbonylative C−C bond activation of aminocyclopropanes. In broader terms, these are the first multicomponent (higher order) cycloadditions where C−C bond activation is enantiodetermining, offering a counterpoint to processes where enantioinduction is achieved at the stage of π‐insertion.^[2g]^ Our reaction design is based on the use of an *endo*‐directing group, which is proposed to facilitate rapid capture of the incipient rhodacyclopentanone, thereby minimizing the reversibility of its formation. This then allows kinetically controlled enantioselectivity achieved during C−C bond activation to be transferred to the product. We are currently investigating alternative “fast trapping” strategies with the aim of exploiting enantioselective C−C bond activations of non‐activated cyclopropanes in other contexts. This would establish these readily available carbocycles as redox active initiating motifs for enantioselective reaction design.

## Conflict of interest

The authors declare no conflict of interest.

## Supporting information

As a service to our authors and readers, this journal provides supporting information supplied by the authors. Such materials are peer reviewed and may be re‐organized for online delivery, but are not copy‐edited or typeset. Technical support issues arising from supporting information (other than missing files) should be addressed to the authors.

Supporting Information

Supporting Information

Supporting Information

## Data Availability

The data that support the findings of this study are available in the Supporting Information of this article.
