# Supplementary material for: An endo‐Directing‐Group Strategy Unlocks Enantioselective (3+1+2) Carbonylative Cycloadditions of Aminocyclopropanes
Source: Angew Chem Int Ed Engl. 2022 Jun 24;61(32):e202205007. doi: 10.1002/anie.202205007 (PMC9401022; doi:10.1002/anie.202205007)
Supplement: Supplementary file 3 — Supporting Information [file ANIE-61-0-s003.pdf]

Angewandte Chemie
Eine Zeitschrift der Gesellschaft Deutscher Chemiker

Supporting Information

An *endo*-Directing-Group Strategy Unlocks Enantioselective (3+1+2) Carbonylative Cycloadditions of Aminocyclopropanes

*O. O. Sokolova, J. F. Bower**

Supporting Information

Table of Contents

General experimental details S2
Experimental Procedures and Data S3
Substrate Synthesis and Catalysis for Table 2 .... S5
Substrate Synthesis and Catalysis for Scheme 2 .... S27
Substrate Synthesis and Catalysis for Scheme 3 .... S34
Selected Other Screened Systems S45
Optimization Studies S48
Reversibility Studies: S52
NMR Spectra of Novel Compounds S54
References S104

General experimental details

All materials for which a synthetic route is not described or referenced were purchased from commercial sources (Sigma-Aldrich, Alfa Aesar, Fluorochem and Strem) and used as received unless otherwise stated. Anhydrous solvents were obtained by distillation using standard procedures or by passage through drying columns supplied by Anhydrous Engineering Ltd. The removal of solvents in vacuo was achieved using both a Büchi rotary evaporator (bath temperatures up to 40 °C) at a pressure of either 15 mmHg (diaphragm pump) or 0.1 mmHg (oil pump), as appropriate, and a high vacuum line at room temperature. Catalytic reactions were carried out inside oven/flame dried glass reaction tubes equipped with a Suba-Seal® and a balloon of inert gas (or carbon monoxide in the case of carbonylation reactions). Flash column chromatography (FCC) was performed using silica gel (Aldrich 40-63 µm, 230-400 mesh). Thin layer chromatography was performed using aluminium backed 60 F_{254} silica plates. Visualisation was achieved by UV fluorescence or a basic $KMnO_4$ solution and heat. Proton nuclear magnetic resonance spectra (NMR) were recorded on the following spectrometers: JEOL ECS400, JEOL ECZ400, Varian 400-MR, Bruker Nano400, Varian VNMR 500, and Bruker Avance III HD 500 Cryoprobe. ^{1}H NMR spectra were recorded at 400 MHz or 500 MHz as stated. ^{13}C NMR spectra were recorded at 101 MHz or 126 MHz as stated. Chemical shifts (δ) are given in parts per million (ppm). Peaks are described as singlets (s), doublets (d), triplets (t), quartets (q), septets (sept), multiplets (m) and broad (br.). Coupling constants (*J*) are quoted to the nearest 0.5 Hz. All assignments of NMR spectra were based on 2D NMR data (COSY, HSQC, HMBC, TOCSY, and nOe experiments where appropriate). Where compounds were isolated as a mixture of isomers (e.g. rotamers), they are referred as A and B. NMR yields were determined by employing 1,4-dinitrobenzene as an internal standard. Mass spectra were determined by the University of Bristol mass spectrometry service using the following instruments: Bruker Daltonics FT-ICR-MS Apex 4e 7.0T FT-MS or Bruker Daltonics micrOTOF II (ESI), Shimadzu GCMS QP2010+ or Thermo Scientific Orbitrap Elite (EI), Bruker ultrafleXtreme 2 (MALDI), Thermo Scientific Orbitrap Elite (APCI) and Waters Synapt G2S (Nanospray). Infrared spectra were recorded on a Perkin Elmer Spectrum Two FT-IR spectrometer as thin films or solids compressed on a diamond plate. Melting points were determined using Reichert melting point apparatus. Optical rotations were measured using an ADP440+ polarimeter at the concentration and temperature stated. Enantiomeric excess was determined using an Agilent 1290 Infinity chiral SFC under the conditions noted for each compound.

Experimental Procedures and Data

General procedure A for the amide coupling with EDCI

To a solution of acid (1.00 eq) in DCM (0.3 M), EDCI (1.10 eq) was added at 0 °C and the reaction was stirred for 10 min. Then, the specified amine (1.00 eq) and DMAP (10 mol%) were added at 0 °C and the reaction was slowly warmed to r.t., stirred for 18 h and then concentrated in vacuo. 1.0 M aq. NaOH (5 mL/mmol) was added and the solution was extracted with EtOAc (3 × 3 mL/mmol). The organic extracts were combined, washed with 1.0 M aq. HCl (5 mL/mmol) and brine (5 mL/mmol), dried over $MgSO_4$ and concentrated *in vacuo*. The product was purified by flash column chromatography, under the conditions noted, to afford the target amide.

General procedure B for the preparation of aryl-substituted alkyne substrates via Sonogashira reaction

An oven-dried reaction tube, fitted with a magnetic stirrer, was charged with terminal alkyne (1.00 eq) and aryl halide (1.10-1.20 eq as noted). The tube was fitted with a rubber septum and purged with argon. Anhydrous Et_3N (1.0 M) was added and the solution was sparged with argon for approx. 5 min. $Pd(PPh_3)_4$ (2 mol%) and CuI (2 mol%) were added, the tube was sealed and stirred (at r.t., 60 or 80 °C as noted) for 2-16 h. The mixture was concentrated *in vacuo*. The residue was purified by flash column chromatography, under the conditions noted, to afford the desired aryl-substituted alkyne.

General procedure C for asymmetric carbonylative ring expansion of protected alkynyl aminocyclopropanes to cyclohexenones

An oven dried reaction tube, fitted with a magnetic stirrer, was charged with aminocyclopropane substrate (100 mol%) and $[Rh(cod)_2]OTf$ (7.5 mol%). In the glovebox, (*S*)-SITCP (15 mol%) was added. The tube was fitted with a rubber septum, taken out of the glovebox and anhydrous 1,2-DCB (0.1 M) was added via syringe. The reaction mixture was sparged with CO for ca. 10 seconds, then heated at 110 °C under a CO atmosphere (1 atm, balloon) for 96 h. The mixture was cooled to r.t., concentrated *in vacuo* and purified by flash column chromatography, under the conditions noted, to afford the target cyclohexenone.

General procedure D for carbonylative ring expansion of protected alkynyl aminocyclopropanes to cyclohexenones

An oven dried reaction tube, fitted with a magnetic stirrer, was charged with aminocyclopropane substrate (100 mol%), PPh_3 (10 mol%) and $[Rh(cod)_2]OTf$ (5 mol%). The tube was fitted with a rubber septum, evacuated and backfilled with nitrogen three times and anhydrous 1,2-DCB (0.1 M) was added via syringe. The reaction mixture was sparged with CO for approx. 10 seconds, then heated at 130 °C under a CO atmosphere (1 atm, balloon) for 48 h. The mixture was cooled to r.t., concentrated *in vacuo* and purified by flash column chromatography, under the conditions noted, to afford the target cyclohexenone.

Substrate Synthesis and Catalysis for Table 2

N-Benzyl-*N*-cyclopropyl-2,2-dimethyl-4-phenylbut-3-ynamide (3a)

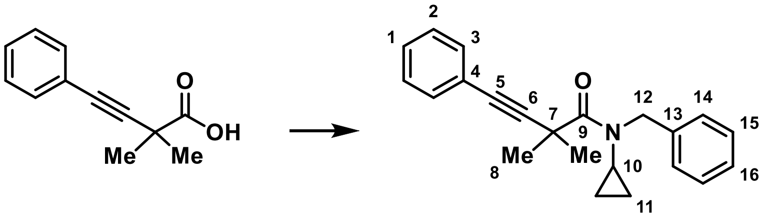

3a

General procedure A: *N*-Benzylcyclopropanamine (0.61 g, 4.12 mmol, prepared according to the literature procedure[1]) and 2,2-dimethyl-4-phenylbut-3-ynoic acid (0.78 g, 4.12 mmol, prepared according to the literature procedure[2]) was employed. Flash column chromatography (30% EtOAc/hexane) afforded the title compound **3a** (1.04 g, 79%) as a colorless solid; m.p. 47-48 °C (DCM/hexane); ν_{max} / cm^{-1}: 2985 (m), 1643 (s), 1394 (s), 1361 (m), 756 (s), 691 (s); ^1H NMR (DMSO-d_6, 500 MHz, 110 °C): δ 7.34 – 7.19 (m, 10H, C**1**-H, C**16**-H, 2 × C**2**-H, 2 × C**3**-H, 2 × C**14**-H and 2 × C**15**-H), 4.79 (s, 2H, C**12**-H_2), 3.08 – 3.00 (m, 1H, C**10**-H), 1.60 (s, 6H, 2 × C**8**-H_3), 0.78 – 0.71 (m, 4H, 2 × C**11**-H_2); ^{13}C NMR (DMSO-d_6, 126 MHz, 110 °C): δ 172.9 (C**9**), 138.0 (C**13**), 130.4, 127.7, 127.7, 127.5, 126.1, 126.0 (C**1**, C**2**, C**3**, C**14**, C**15** and C**16**), 122.2 (C**4**), 92.7 (C**6**), 82.8 (C**5**), 49.8 (C**12**), 37.3 (C**7**), 30.5 (C**10**), 28.1 (C**8**), 7.2 (C**11**); HRMS: (ESI^+) calculated for $C_{22}H_{24}NO$: 318.1852, found $[M+H]^+$: 318.1847.

1-Benzyl-3,3-dimethyl-4-phenyl-1,6,7,7a-tetrahydro-2*H*-indole-2,5(3*H*)-dione (4a)

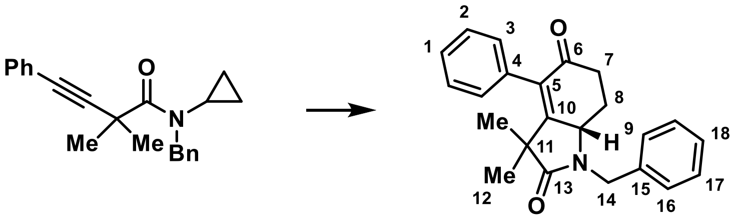

3a 4a

General procedure C: Amide **3a** (31.7 mg, 0.10 mmol) was employed. Flash column chromatography (40% EtOAc/hexane) afforded the title compound **(*S*)-4a** (26.3 mg, 76%) as a colorless oil; ν_{max} / cm^{-1}: 2930 (w), 1697 (s), 1674 (s), 1418 (m), 1242 (m), 701 (s); ^1H NMR ($CDCl_3$, 400 MHz): δ 7.40 – 7.25 (m, 8H, C**1**-H, C**18**-H, 2 × C**2**-H, 2 × C**16**-H and 2 × C**17**-H), 7.05 – 7.00 (m, 2H, 2 × C**3**-H), 5.11 (d, *J* = 14.9 Hz, 1H, C**14**-H_aH_b), 4.23 (dd, *J* = 11.7, 4.4 Hz, 1H, C**9**-H), 4.11 (d, *J* = 14.9 Hz, 1H, C**14**-H_aH_b), 2.73 (ddd, *J* = 17.7, 4.4, 2.4 Hz, 1H, C**7**-H_aH_b), 2.50 (dtd, *J* = 11.7, 4.8, 2.4 Hz, 1H, C**8**-H_aH_b), 2.38 (ddd, *J* = 17.7, 14.1, 4.8 Hz, 1H, C**7**-H_aH_b), 1.82 (dtd, *J* = 14.1, 11.7, 4.4 Hz, 1H, C**8**-H_aH_b), 1.34 (s, 3H, C**12**-H_3), 0.82 (s, 3H, C**12'**-H_3); ^{13}C

NMR ($CDCl_3$, 101 MHz): δ 196.8 (C**6**), 177.4 (C**13**), 162.4 (C**10**), 136.1, 136.0 (C**5** and C**15**), 134.1 (C**4**), 129.9 (C**3**), 129.1, 128.1, 128.1, 128.1, 128.1 (C**1**, C**2**, C**16**, C**17** and C**18**), 55.8 (C**9**), 46.0 (C**11**), 44.1 (C**14**), 35.8 (C**7**), 28.4 (C**8**), 26.2 (C**12**), 21.9 (C**12'**); HRMS: (ESI^+) calculated for $C_{23}H_{24}NO_2$: 346.1802, found $[M+H]^+$: 346.1786.

The enantiopurity of this compound was determined by chiral SFC against a racemic standard.

$[\alpha]_D^{23.7}$ = -22.9 (c = 0.41, $CHCl_3$).

Chiral SFC: (DAICEL CHIRALPAK-IB column (25 cm), CO_2:MeOH 88:12, 2 mL/min, 140 bars, 40 °C). Retention times: 13.4 minutes (major), 15.0 minutes (minor), e.r. = 95:5.

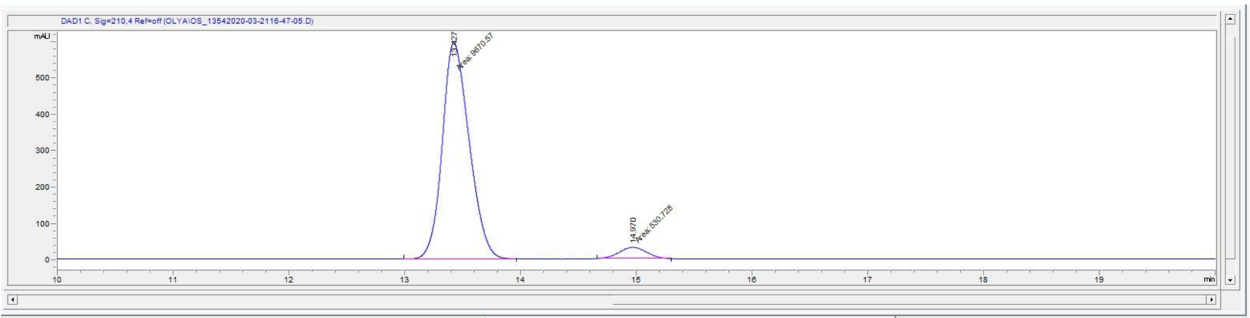

#	Time	Area	Height	Width	Area%	Symmetry
1	13.427	9670.6	595	0.2709	94.797	0.78
2	14.97	530.7	31.7	0.279	5.203	0.836

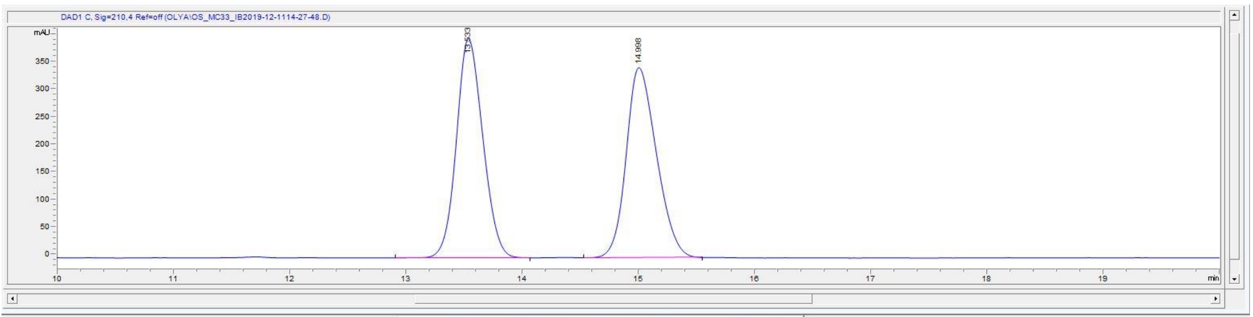

#	Time	Area	Height	Width	Area%	Symmetry
1	13.533	6273.5	396.9	0.2444	50.163	0.817
2	14.998	6232.7	344.2	0.2773	49.837	0.705

General procedure D: Amide **3a** (31.7 mg, 0.10 mmol) was employed. Flash column chromatography (40% EtOAc/hexane) afforded the title compound **4a** (26.5 mg, 77%) as a colorless oil.

N-Butyl-N-cyclopropyl-2,2-dimethyl-4-phenylbut-3-ynamide (3b)

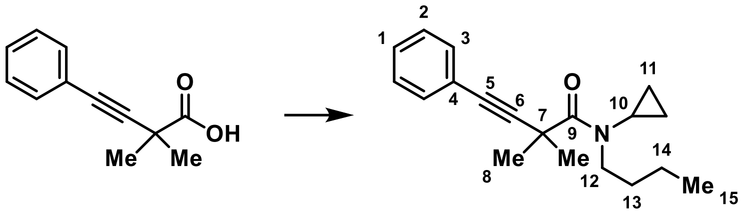

General procedure A: *N*-Butylcyclopropanamine (0.18 g, 1.59 mmol, prepared according to the literature procedure[3]) and 2,2-dimethyl-4-phenylbut-3-ynoic acid (0.30 g, 1.59 mmol, prepared according to the literature procedure[2]) were employed and the residue was purified by flash column chromatography (5-10% EtOAc/hexane) to afford the title compound **3b** (0.21 g, 47%) as a colorless oil; ν_{max} / cm^{-1}: 2957 (m), 2934 (m), 1648 (s), 1465 (m), 1399 (s), 1289 (m), 756 (s), 692 (s); ^{1}H NMR (DMSO-d_6, 500 MHz, 110 °C): δ 7.38 – 7.33 (m, 5H, 2 × C**2**-H, 2 × C**3**-H and C**1**-H), 3.49 (t, *J* = 7.6 Hz, 2H, C**12**-H_2), 2.94 (br. s, 1H, C**10**-H), 1.62 – 1.55 (m, 2H, C**13**-H_2), 1.52 (s, 6H, 2 × C**8**-H_3), 1.32 – 1.24 (m, 2H, C**14**-H_2), 0.88 – 0.80 (m, 5H, C**15**-H_3 and 2 × C**11**-H_aH_b), 0.71 – 0.65 (m, 2H, 2 × C**11**-H_aH_b); ^{13}C NMR (DMSO-d_6, 126 MHz, 110 °C): δ 172.3 (C**9**), 130.5, 128.0 (C**2** and C**3**), 127.6 (C**1**), 122.4 (C**4**), 93.0 (C**6**), 82.4 (C**5**), 46.2 (C**12**), 37.1 (C**7**), 29.7 (C**13**), 29.4 (C**10**), 28.0 (C**8**), 19.0 (C**14**), 12.9 (C**15**), 7.3 (C**11**); HRMS: (ESI^+) calculated for $C_{19}H_{26}NO$: 284.2009, found $[M+H]^+$:284.2009.

1-Butyl-3,3-dimethyl-4-phenyl-1,6,7,7a-tetrahydro-2*H*-indole-2,5(3*H*)-dione (4b)

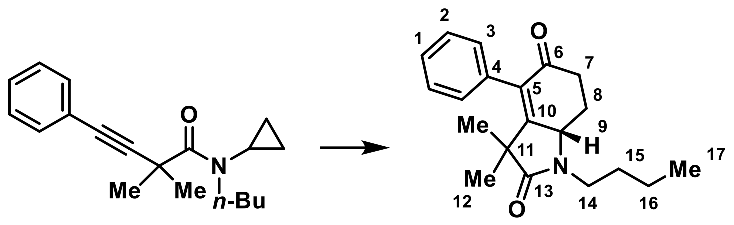

General procedure C: Amide **3b** (28.3 mg, 0.10 mmol) was employed. Flash column chromatography (40% EtOAc/hexane) afforded the title compound **(*S*)-4b** (16.1 mg, 52%) as a colorless oil; ν_{max} / cm^{-1}: 2957 (m), 2930 (m), 2871 (m), 1694 (s), 1674 (s), 1644 (m), 1462 (m), 1422 (m), 1241 (m), 1201 (m), 755 (m), 705 (s); ^{1}H NMR ($CDCl_3$, 400 MHz): δ 7.39 – 7.32 (m, 3H, 2 × C**2**-H and C**1**-H), 7.06 – 7.00 (m, 2H, 2 × C**3**-H), 4.43 (dd, *J* = 11.6, 4.1 Hz, 1H, C**9**-H), 3.69 (ddd, *J* = 13.8, 8.7, 7.3 Hz, 1H, C**14**-H_aH_b), 3.09 (ddd, *J* = 13.8, 8.5, 5.2 Hz, 1H, C**14**-H_aH_b), 2.85 – 2.76 (m, 1H, C**7**-H_aH_b), 2.61 – 2.46 (m, 2H, C**7**-H_aH_b and C**8**-H_aH_b), 1.91 – 1.78 (m, 1H, C**8**-H_aH_b), 1.65 – 1.48 (m, 2H, C**15**-H_2), 1.41 – 1.31 (m, 2H, C**16**-H_2), 1.30 (s, 3H, C**12**-H_3), 0.95

(t, J = 7.3 Hz, 3H, C**17**-H$_3$), 0.74 (s, 3H, C**12'**-H$_3$); ^{13}C NMR ($CDCl_3$, 101 MHz): δ 196.9 (C**6**), 177.0 (C**13**), 163.0 (C**10**), 136.0 (C**5**), 134.1 (C**4**), 129.9 (C**3**), 128.1 (C**1** and C**2**), 56.3 (C**9**), 46.0 (C**11**), 39.9 (C**14**), 35.9 (C**7**), 29.5 (C**15**), 28.6 (C**8**), 26.4 (C**12**), 21.9 (C**12'**), 20.2 (C**16**), 13.9 (C**17**); HRMS: (ESI$^+$) calculated for $C_{20}H_{26}NO_2$: 312.1958, found [M+H]$^+$: 312.1958.

The enantiopurity of this compound was determined by chiral SFC against a racemic standard.

$[\alpha]_D^{24.2}$ = 16.7 (c = 0.23, $CHCl_3$).

Chiral SFC: (DAICEL CHIRALPAK-IB column (25 cm), CO_2:MeOH 94:6, 2 mL/min, 140 bars, 40 °C). Retention times: 12.9 minutes (major), 13.5 minutes (minor), e.r. = 91:9.

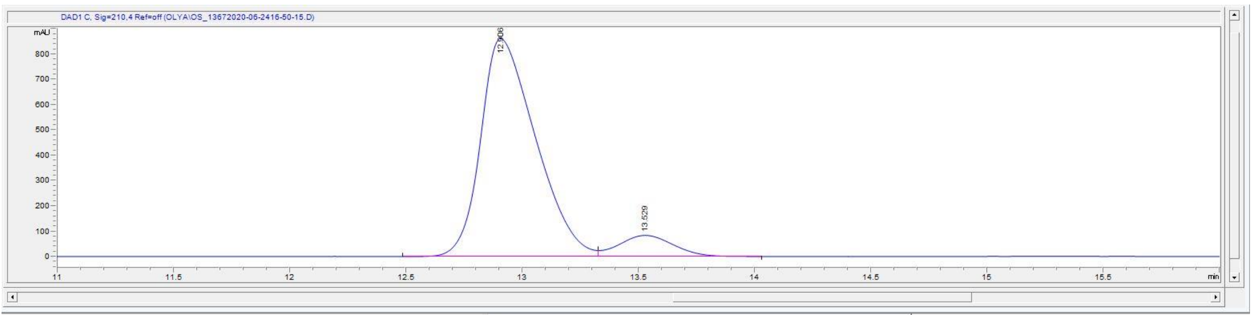

#	Time	Area	Height	Width	Area%	Symmetry
1	12.906	14125.3	859.6	0.2517	91.256	0.556
2	13.529	1353.5	83.5	0.2431	8.744	0.893

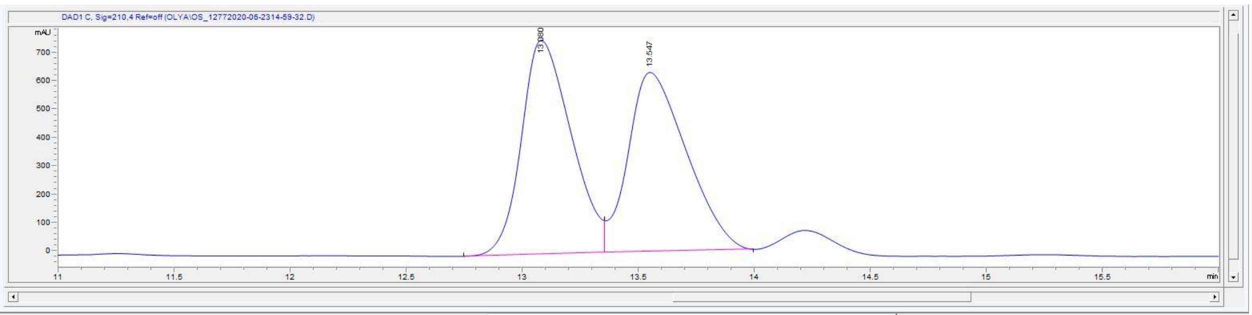

#	Time	Area	Height	Width	Area%	Symmetry
1	13.08	11353.8	758.9	0.2305	50.619	0.65
2	13.547	11076.1	635.6	0.2653	49.381	0.584

General procedure D: Amide **3b** (28.3 mg, 0.10 mmol) was employed. Flash column chromatography (35% EtOAc/hexane) afforded the title compound **4b** (21.9 mg, 70%) as a pale yellow oil.

1-(Phenylethynyl)cyclopentane-1-carboxylic acid

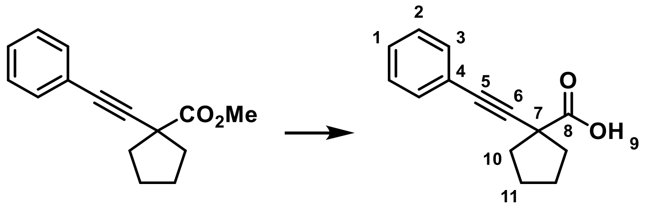

To a solution of the corresponding ester (1.00 g, 4.38 mmol, prepared according to the literature procedure[4]) in MeOH (8 mL) was added 4.0 M aq. NaOH (5.5 mL) and the reaction was stirred at r.t. for 16 h. The reaction mixture was concentrated *in vacuo*, diluted with water (20 mL) and extracted with Et_2O (20 mL). The aqueous portion was adjusted to pH 2 by addition of 6.0 M aq. HCl and then extracted with Et_2O (3 × 20 mL). The organic extracts were combined, dried over $MgSO_4$ and concentrated *in vacuo* to afford the title compound (0.92 g, 98%) as a colorless solid; m.p. 78-79 °C (DCM/hexane); ν_{max} / cm^{-1}: 2957 (br. m), 1703 (s), 1273 (m), 755 (m), 690 (m); ^1H NMR ($CDCl_3$, 400 MHz): δ 10.41 (br. s, 1H, O**9**-H), 7.45 – 7.40 (m, 2H, 2 × C**3**-H), 7.32 – 7.27 (m, 3H, C**1**-H and 2 × C**2**-H), 2.40 – 2.27 (m, 2H, 2 × C**10**-H_aH_b), 2.23 – 2.09 (m, 2H, 2 × C**10**-H_aH_b), 1.98 – 1.75 (m, 4H, 2 × C**11**-H_2); ^{13}C NMR ($CDCl_3$, 101 MHz): δ 179.6 (C**8**), 131.9 (C**3**), 128.3 (C**2**), 128.2 (C**1**), 123.2 (C**4**), 90.7 (C**6**), 82.9 (C**5**), 48.9 (C**7**), 39.5 (C**10**), 25.1 (C**11**); HRMS: (ESI^-) calculated for $C_{14}H_{13}O_2$: 213.0916, found $[M-H]^-$: 213.0909.

***N*-Benzyl-*N*-cyclopropyl-1-(phenylethynyl)cyclopentane-1-carboxamide (3c)**

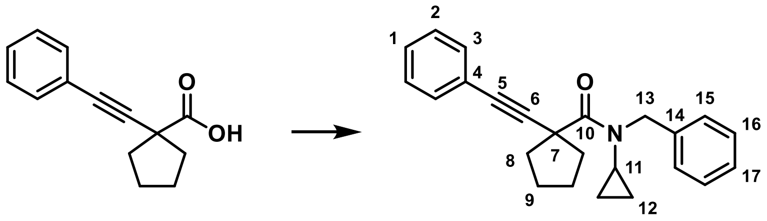

3c

General procedure A: *N*-Benzylcyclopropanamine (0.34 g, 2.31 mmol) and the preceding acid (0.50 g, 2.33 mmol) were employed and the residue was purified by flash column chromatography (5% EtOAc/hexane) to afford the title compound **3c** (0.61 g, 77%) as a colorless oil; ν_{max} / cm^{-1}: 2955 (m), 1645 (s), 1491 (m), 1453 (m), 1389 (s), 756 (s), 692 (s); ^1H NMR (DMSO-d_6, 500 MHz, 80 °C): δ 7.34 – 7.19 (m, 10H, C**1**-H, C**17**-H, 2 × C**2**-H, 2 × C**3**-H, 2 × C**15**-H and 2 × C**16**-H), 4.73 (br. s, 2H, C**13**-H_2), 3.06 – 2.93 (br. m, 1H, C**11**-H), 2.41 – 2.33 (m, 2H, 2 × C**8**-H_aH_b), 2.19 – 2.10 (m, 2H, 2 × C**8**-H_aH_b), 1.88 – 1.78 (m, 2H, 2 × C**9**-H_aH_b), 1.76 – 1.66 (m, 2H, 2 × C**9**-H_aH_b), 0.79 – 0.69 (m, 4H, 2 × C**12**-H_2); ^{13}C NMR (DMSO-d_6, 126 MHz, 80 °C): δ 172.39 (C**10**),

138.15 (C**14**), 130.68, 128.02, 127.89, 127.70, 126.31, 126.25 (C**1**, C**2**, C**3**, C**15**, C**16** and C**17**), 122.36 (C**4**), 92.71 (C**5**), 82.57 (C**6**), 49.54 (C**7**), 48.08 (C**13**), 38.87 (C**8**), 30.30 (C**11**), 23.97 (C**9**), 7.40 (C**12**); HRMS: (ESI^+) calculated for $C_{24}H_{26}NO$: 344.2009, found $[M+H]^+$: 344.1998.

Even at high temperature (80 °C), significant broadening of the ^{1}*H NMR signals and weak signal intensities of* ^{13}*C NMR was observed due to amide resonance.*

1'-benzyl-4'-phenyl-1',6',7',7a'-tetrahydrospiro[cyclopentane-1,3'-indole]-2',5'-dione (4c)

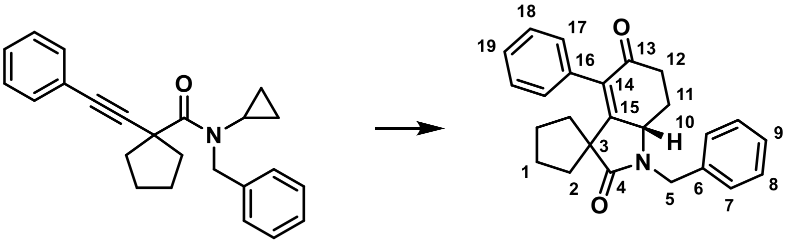

3c 4c

General procedure C: Amide **3c** (34.3 mg, 0.10 mmol) was employed. Flash column chromatography (50% EtOAc/hexane) afforded the title compound **(*S*)-4c** (24.5 mg, 66%) as a colorless oil ; ν_{max} / cm^{-1}: 2947 (m), 1693 (s), 1674 (s), 1443 (m), 1417 (s), 1313 (m), 1269 (m), 1174 (m); ^{1}H NMR ($CDCl_3$, 400 MHz): δ 7.41 – 7.27 (m, 8H, C**9**-H, C**19**-H, 2 × C**7**-H, 2 × C**8**-H and 2 × C**18**-H), 7.06 – 7.00 (m, 2H, 2 × C**17**-H), 5.09 (d, *J* = 14.9 Hz, 1H, C**5**-H_aH_b), 4.23 (dd, *J* = 11.8, 4.2 Hz, 1H, C**10**-H), 4.11 (d, *J* = 14.9 Hz, 1H, C**5**-H_aH_b), 2.73 (ddd, *J* = 17.8, 4.5, 2.2 Hz, 1H, C**12**-H_aH_b), 2.51 – 2.43 (m, 1H, C**11**-H_aH_b), 2.43 – 2.32 (m, 1H, C**12**-H_aH_b), 2.15 – 2.07 (m, 1H, C**2**-H_aH_b), 1.96 – 1.48 (m, 7H, C**1**-H_2, C**1'**-H_aH_b, C**2'**-H_2, C**2**-H_aH_b and C**11**-H_aH_b), 0.74 – 0.61 (m, 1H, C**1'**-H_aH_b); ^{13}C NMR ($CDCl_3$, 126 MHz): δ 196.9 (C**13**), 178.9 (C**4**), 163.5 (C**15**), 136.2 (C**6**), 134.9 (C**14**), 134.1 (C**16**), 130.2 (C**17**), 129.1, 128.2, 128.1 (C**7**, C**8** and C**18**), 128.0, 128.0 (C**9** and C**19**), 56.9 (C**10**), 54.9 (C**3**), 44.1 (C**5**), 42.1 (C**2**), 35.9 (C**12**), 34.2 (C**2'**), 28.2 (C**11**), 27.4 (C**1'**), 27.2 (C**1**); HRMS: (ESI^+) calculated for $C_{25}H_{26}NO_2$: 372.1958, found $[M+H]^+$: 372.1948.

The enantiopurity of this compound was determined by chiral SFC against a racemic standard.

$[\alpha]_D^{23.8}$ = -48.1 (c = 0.11, $CHCl_3$).

Chiral SFC: (DAICEL CHIRALPAK-IB column (25 cm), CO_2:MeOH 80:20, 2 mL/min, 140 bars, 40 °C). Retention times: 9.6 minutes (major), 11.0 minutes (minor), e.r. = 92:8.

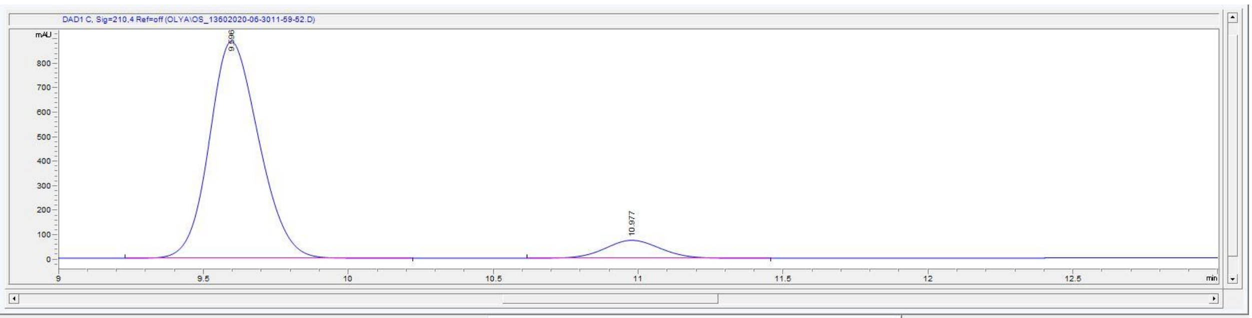

#	Time	Area	Height	Width	Area%	Symmetry
1	9.596	10519.9	885.4	0.1826	91.552	0.779
2	10.977	970.7	73.1	0.2069	8.448	0.921

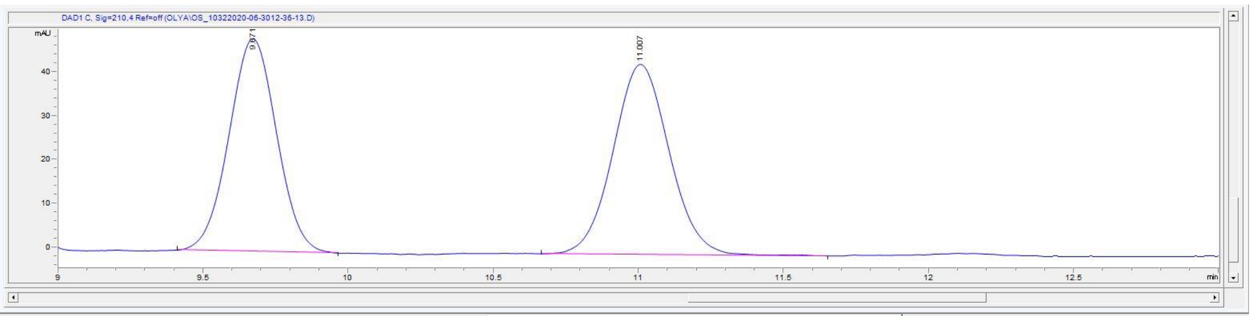

#	Time	Area	Height	Width	Area%	Symmetry
1	9.671	550.8	48.3	0.1812	48.616	0.98
2	11.007	582.2	43.1	0.2094	51.384	0.916

General procedure D: Amide **3c** (34.3 mg, 0.10 mmol) was employed. Flash column chromatography (30% EtOAc/hexane) afforded the title compound **4c** (27.9 mg, 75%) as a pale brown oil.

1-(Phenylethynyl)cyclohexane-1-carboxylic acid

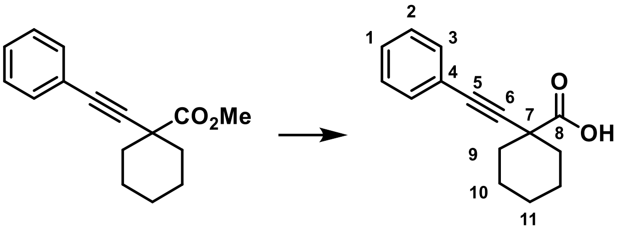

To a solution of the corresponding ester (2.08 g, 8.58 mmol, prepared according to the literature procedure[4]) in MeOH (17 mL) was added 4.0 M aq. NaOH (10.7 mL) and the reaction was stirred at r.t. for 16 h. The reaction mixture was concentrated *in vacuo*, diluted with water (20 mL) and extracted with Et_2O (20 mL). The aqueous portion was adjusted to pH 2 by addition of 6.0 M aq. HCl and then extracted with Et_2O (3 × 20 mL). The organic extracts were combined, dried over $MgSO_4$ and concentrated *in vacuo* to afford the title compound (1.65 g, 84%) as a colorless solid; m.p. 72-73 °C ($CDCl_3$); ν_{max} / cm^{-1}: 2933 (m), 1704 (s), 1444 (m), 1284 (m), 1258 (m), 755 (m);

^{1}H NMR ($CDCl_3$, 400 MHz): δ 7.49 – 7.43 (m, 2H, 2 × C**3**-H), 7.33 – 7.27 (m, 3H, C**1**-H and 2 × C**2**-H), 2.11 – 2.01 (m, 2H, 2 × C**9**-H_aH_b), 1.93 – 1.61 (m, 7H, 2 × C**9**-H_aH_b, 2 × C**10**-H_2 and C**11**-H_aH_b), 1.36 – 1.22 (m, 1H, C**11**-H_aH_b); ^{13}C NMR ($CDCl_3$, 101 MHz): δ 179.6 (C**8**), 131.9 (C**3**), 128.3 (C**2**), 128.3 (C**1**), 123.2 (C**4**), 89.1 (C**6**), 85.0 (C**5**), 44.4 (C**7**), 34.8 (C**9**), 25.5 (C**11**), 22.6 (C**10**); HRMS: (ESI^-) calculated for $C_{15}H_{15}O_2$: 227.1072, found $[M-H]^-$: 227.1081.

_N_-Benzyl-_N_-cyclopropyl-1-(phenylethynyl)cyclohexane-1-carboxamide (3d)

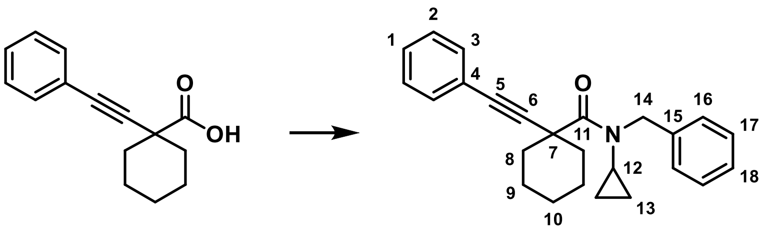

3d

General procedure A: _N_-Benzylcyclopropanamine (0.36 g, 2.45 mmol) and the preceding acid (0.56 g, 2.45 mmol) were employed and the residue was purified by flash column chromatography (5% EtOAc/hexane) to afford the title compound **3d** (0.67 g, 77%) as a pale yellow oil; ν_{max} / cm^{-1}: 2932 (m), 1651 (s), 1386 (m), 756 (m), 692 (m); ^{1}H NMR (DMSO-d_6, 500 MHz, 110 °C): δ 7.34 – 7.19 (m, 10H, C**1**-H, C**18**-H, 2 × C**2**-H, 2 × C**3**-H, 2 × C**16**-H and 2 × C**17**-H), 4.81 (s, 2H, C**14**-H_2), 3.03 – 2.95 (m, 1H, C**12**-H), 2.10 – 2.02 (m, 2H, 2 × C**8**-H_aH_b), 1.95 – 1.87 (m, 2H, 2 × C**8**-H_aH_b), 1.82 – 1.71 (m, 2H, 2 × C**9**-H_aH_b), 1.70 – 1.61 (m, 3H, 2 × C**9**-H_aH_b and C**10**-H_aH_b), 1.34 – 1.23 (m, 1H, C**10**-H_aH_b), 0.73 – 0.69 (m, 2H, 2 × C**13**-H_2); ^{13}C NMR (DMSO-d_6, 126 MHz, 110 °C): δ 172.8 (C**11**), 138.0 (C**15**), 130.4, 127.8, 127.6, 127.5, 126.2, 126.0 (C**1**, C**2**, C**3**, C**16**, C**17** and C**18**), 122.2 (C**4**), 90.4 (C**6**), 86.1 (C**5**), 49.8 (C**14**), 43.1 (C**7**), 34.9 (C**8**), 30.3 (C**12**), 24.5 (C**10**), 21.6 (C**9**), 7.1 (C**13**); HRMS: (ESI^+) calculated for $C_{25}H_{28}NO$: 358.2165, found $[M+H]^+$: 358.2159.

1'-Benzyl-4'-phenyl-1',6',7',7a'-tetrahydrospiro[cyclohexane-1,3'-indole]-2',5'-dione (4d) and 1'-benzyl-5'-hydroxy-4'-phenylspiro[cyclohexane-1,3'-indolin]-2'-one (4d')

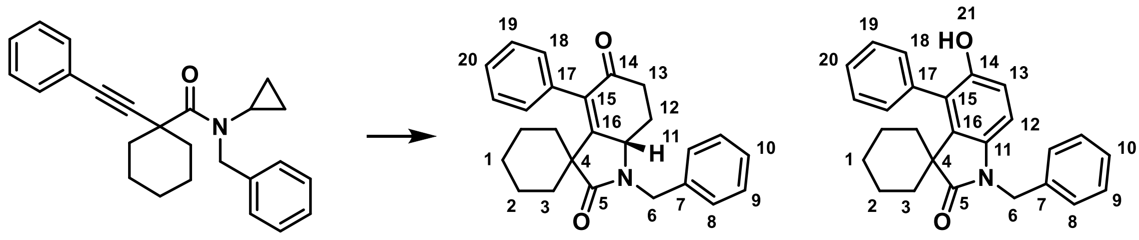

3d 4d 4d'

General procedure C: *In a modification to the general procedure, the reaction was heated to 120 °C.* Amide **3d** (35.8 mg, 0.10 mmol) was employed. Flash column chromatography (40% EtOAc/hexane) afforded the title compound **(*S*)-4d** (26.8 mg, 69%) as a yellow oil; ν_{max} / cm^{-1}: 2924 (m), 1674 (s), 1414 (m), 1258 (m), 1078 (m), 910 (m); ^{1}H NMR (CDCl$_3$, 400 MHz): δ 7.41 – 7.23 (m, 8H, C**10**-H, C**20**-H, 2 × C**8**-H, 2 × C**9**-H and 2 × C**19**-H), 7.04 – 6.99 (m, 2H, 2 × C**18**-H), 5.11 (d, *J* = 14.9 Hz, 1H, C**6**-H$_a$H$_b$), 4.18 (dd, *J* = 11.7, 4.2 Hz, 1H, C**11**-H), 4.06 (d, *J* = 14.9 Hz, 1H, C**6**-H$_a$H$_b$), 2.71 (ddd, *J* = 17.9, 4.5, 2.3 Hz, 1H, C**13**-H$_a$H$_b$), 2.49 – 2.42 (m, 1H, C**12**-H$_a$H$_b$), 2.40 – 2.20 (m, 2H, C**2**-H$_a$H$_b$ and C**13**-H$_a$H$_b$), 1.97 – 1.47 (m, 6H, C**1**-H$_2$, C**2'**-H$_2$, C**3**-H$_a$H$_b$ and C**12**-H$_a$H$_b$), 1.45 – 1.36 (m, 1H, C**3'**-H$_a$H$_b$), 1.24 – 1.15 (m, 1H, C**2**-H$_a$H$_b$), 1.09 (td, *J* = 13.2, 4.7 Hz, 1H, C**3'**-H$_a$H$_b$), 0.86 – 0.72 (m, 1H, C**3**-H$_a$H$_b$); ^{13}C NMR (CDCl$_3$, 101 MHz): δ 197.2 (C**14**), 176.5 (C**5**), 162.5 (C**16**), 136.3 (C**7**), 135.6 (C**15**), 134.3 (C**17**), 130.1 (C**18**), 129.1, 128.1, 128.0 (C**8**, C**9**, C**10**, C**19** and C**20**), 55.6 (C**11**), 48.6 (C**4**), 43.6 (C**6**), 35.8 (C**13**), 35.1 (C**1**), 30.1 (C**3**), 28.2 (C**12**), 25.1 (C**3'**), 21.6 (C**2**), 20.9 (C**2'**); HRMS: (ESI$^+$) calculated for $C_{26}H_{28}NO_2$: 386.2115, found [M+H]$^+$: 386.2112.

The enantiopurity of this compound was determined by chiral SFC against a racemic standard.

$[\alpha]_D^{23.9}$ = -20.5 (c = 0.57, $CHCl_3$).

Chiral SFC: (DAICEL CHIRALPAK-IB column (25 cm), CO_2:MeOH 88:12, 2 mL/min, 140 bars, 40 °C). Retention times: 16.7 minutes (major), 21.3 minutes (minor), e.r. = 86:14.

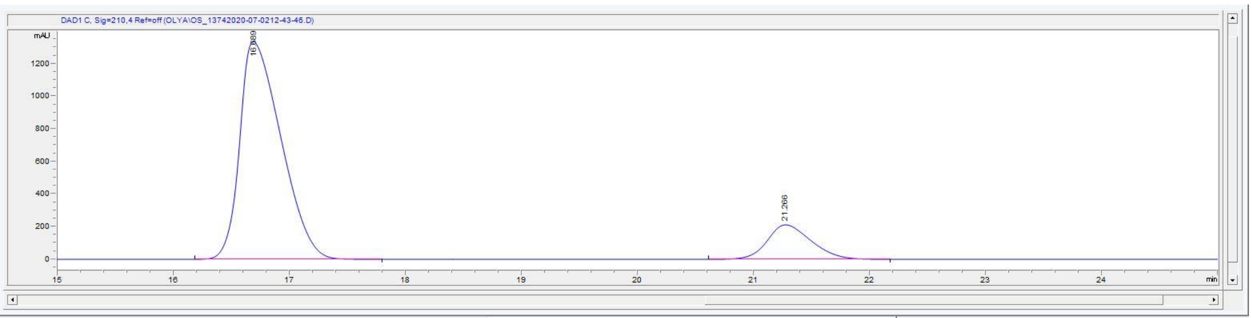

#	Time	Area	Height	Width	Area%	Symmetry
1	16.689	32790.2	1332.3	0.3871	85.593	0.483
2	21.266	5519.3	210.8	0.4015	14.407	0.701

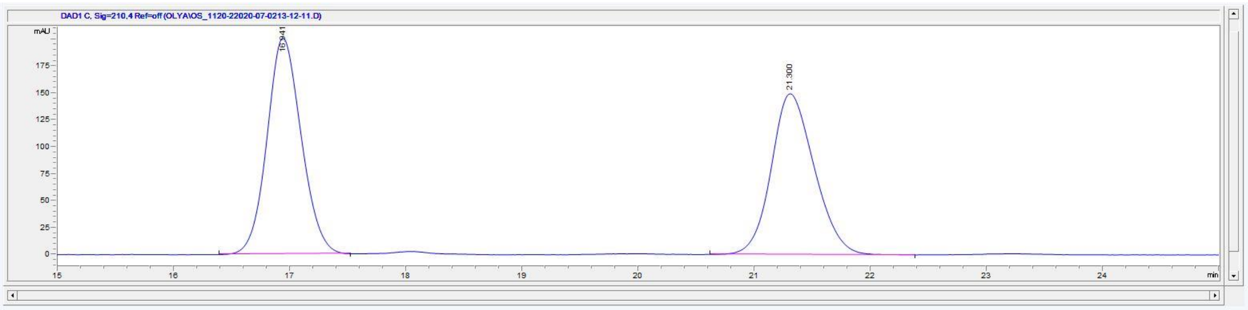

#	Time	Area	Height	Width	Area%	Symmetry
1	16.941	4112.4	200.9	0.3128	51.685	0.854
2	21.3	3844.2	149.3	0.3964	48.315	0.767

Note: Under non-optimised reaction conditions, competing oxidation of the cyclohexenone ring of **4d** to the corresponding phenol **4d'** was observed.

General procedure D: *In a modification to the general procedure, 10 mol% [Rh(cod)$_2$]OTf and 20 mol% PPh$_3$ were used and the reaction was heated for 72 h.* Amide **3d** (35.8 mg, 0.10 mmol) was employed. Flash column chromatography (30% EtOAc/hexane) afforded cyclohexenone **4d** (10.2 mg, 26%) and phenol **4d'** (7.5 mg, 20%) as pale yellow oils.

General procedure D: Amide **3d** (35.8 mg, 0.10 mmol) was employed. Flash column chromatography (30% EtOAc/hexane) afforded the title compound **4d** (29.9 mg, 78%) as a pale brown oil.

Data for **4d'**: ν_{max} / cm^{-1}: 3356 (br. m), 2924 (s), 1675 (s), 1613 (s), 1458 (s), 1340 (s), 1075 (s); ^{1}H NMR (CDCl$_3$, 400 MHz): δ 7.49 – 7.16 (m, 10H, C**10**-H, C**20**-H, 2 × C**8**-H, 2 × C**9**-H, 2 × C**18**-H and 2 × C**19**-H), 6.69 (d, *J* = 8.4 Hz, 1H, C**12**-H), 6.52 (d, *J* = 8.4 Hz, 1H, C**13**-H), 4.81 (s, 2H, C**6**-H_2), 4.16 (s, 1H, O**21**-H), 2.18 – 2.03 (m, 2H, 2 × C**2**-H_aH_b), 1.62 – 1.53 (m, 5H, C**1**-H_aH_b and 2 × C**3**-H_2), 1.34 – 1.26 (m, 2H, 2 × C**2**-H_aH_b), 0.69 – 0.55 (m, 1H, C**1**-H_aH_b); ^{13}C NMR (CDCl$_3$, 101 MHz): δ 179.9 (C**5**), 149.4 (C**14**), 136.6, 135.3, 132.8, 132.8, 131.5, 129.2, 129.1, 128.9, 127.6, 127.3, 126.6 (C**7**, C**8**, C**9**, C**10**, C**11**, C**15**, C**16**, C**17**, C**18**, C**19** and C**20**), 113.3 (C**12**), 109.1 (C**13**), 48.8 (C**4**), 43.2 (C**6**), 32.4 (C**3**), 25.3 (C**1**), 20.5 (C**2**); HRMS: (ESI$^+$) calculated for $C_{26}H_{26}NO_2$: 384.1958, found $[M+H]^+$: 384.1956.

N-benzyl-*N*-cyclopropyl-2,2-dimethyl-4-(trimethylsilyl)but-3-ynamide (3j)

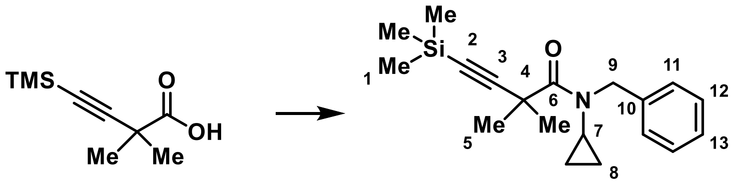

3j

General procedure A: *N*-Benzylcyclopropanamine (2.96 g, 20.07 mmol) and 2,2-dimethyl-4-(trimethylsilyl)but-3-ynoic acid (3.70 g, 20.07 mmol, prepared according to the literature procedure[5]) were employed and the residue was purified by flash column chromatography (20% EtOAc/hexane) to afford the title compound **3j** (4.69 g, 75%) as a colorless solid; m.p. 57-58 °C (DCM/hexane); ν_{max} / cm^{-1}: 2959 (w), 2155 (w), 1650 (s), 1395 (m), 1249 (m), 878 (m), 840 (s); ^{1}H NMR (DMSO-d_6, 500 MHz, 110 °C): δ 7.35 – 7.30 (m, 2H, 2 × C**11**-H or 2 × C**12**-H), 7.26 – 7.20 (m, 3H, C**13**-H and 2 × C**11**-H or 2 × C**12**-H), 4.75 (s, 2H, C**9**-H_2), 3.03 – 2.96 (br. m, 1H, C**7**-H), 1.49 (s, 6H, 2 × C**5**-H_3), 0.72 – 0.67 (m, 4H, 2 × C**8**-H_2), 0.05 (s, 9H, 3 × C**1**-H_3); ^{13}C NMR (DMSO-d_6, 126 MHz, 110 °C): δ 172.6 (C**6**), 137.9 (C**10**), 127.6, 126.1 (C**11** and C**12**), 126.0 (C**13**), 109.4 (C**2**), 86.9 (C**3**), 49.6 (C**9**), 37.5 (C**4**), 30.4 (C**7**), 27.9 (C**5**), 7.2 (C**8**), -0.9 (C**1**); HRMS: (ESI^+) calculated for $C_{19}H_{28}NOSi$: 314.1935, found $[M+H]^+$: 314.1933.

N-Benzyl-*N*-cyclopropyl-2,2-dimethylbut-3-ynamide (3j')

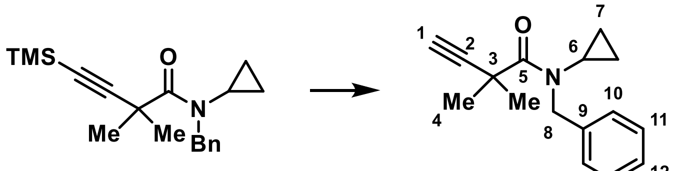

3j **3j'**

Trimethylsilyl alkyne **3j** (3.50 g, 11.16 mmol) was dissolved in MeOH (22 mL) and the solution was cooled to 0°C. Potassium carbonate (4.63 g, 33.49 mmol) was added at once, the reaction was warmed to r.t. and stirred for 2 h. The reaction mixture was concentrated *in vacuo*, diluted with 1M HCl (50 mL) and extracted with EtOAc (3 × 30 mL). The organic extracts were combined, dried over $MgSO_4$ and concentrated *in vacuo*. The residue was purified by flash column chromatography (10% EtOAc/hexane) to afford the title compound **3j'** (2.21 g, 82%) as a colorless oil; ν_{max} / cm^{-1}: 3288 (w), 3235 (w), 2987 (w), 1644 (s), 1396 (s), 1168 (w), 1029 (m), 698 (m); ^{1}H NMR (DMSO-d_6, 500 MHz, 110 °C): δ 7.33 – 7.29 (m, 2H, 2 × C**11**-H), 7.24 – 7.19 (m, 3H, C**12**-H and 2 × C**10**-H), 4.72 (s, 2H, C**8**-H_2), 3.07 – 3.04 (br. m, 1H, C**6**-H), 3.03 (br. s, 1H, C**1**-H), 1.52 (s, 6H, 2 × C**4**-H_3), 0.74 – 0.70 (m, 4H, 2 × C**7**-H_2); ^{13}C NMR (DMSO-d_6, 126 MHz, 110 °C): δ 172.7 (C**5**), 138.0 (C**9**), 127.6 (C**11**), 126.1 (C**10**), 126.0 (C**12**), 86.9 (C**2**), 73.2 (C**1**), 49.6

(C**8**), 36.6 (C**3**), 30.5 (C**6**), 28.0 (C**4**), 7.3 (C**7**); HRMS: (ESI$^+$) calculated for $C_{16}H_{20}NO$: 242.1529, found $[M+H]^+$: 242.1547.

***N*-Benzyl-*N*-cyclopropyl-4-(4-methoxyphenyl)-2,2-dimethylbut-3-ynamide (3e)**

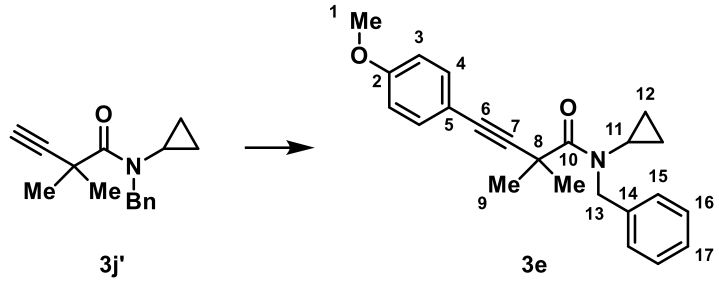

3j' **3e**

General procedure B: Aminocyclopropane **3j'** (200.0 mg, 0.83 mmol) and 4-iodoanisole (213.4 mg, 0.91 mmol) were employed and the reaction was stirred for 2 h at r.t. Flash column chromatography (10% EtOAc/hexane) afforded alkyne **3e** (246.1 mg, 85%) as an orange solid; m.p. 60-61 °C (DCM/hexane); ν_{max} / cm^{-1}: 2935 (w), 1645 (s), 1509 (s), 1247 (s), 1167 (s), 1029 (m), 831 (s); ^{1}H NMR (DMSO-d_6, 500 MHz, 110 °C): δ 7.33 – 7.20 (m, 5H, 2 × C**15**-H, 2 × C**16**-H and C**17**-H), 7.18 – 7.13 (m, 2H, 2 × C**4**-H), 6.87 – 6.83 (m, 2H, 2 × C**3**-H), 4.79 (s, 2H, C**13**-H_2), 3.77 (s, 3H, C**1**-H_3), 3.07 – 3.00 (br. m, 1H, C**11**-H), 1.59 (s, 6H, 2 × C**9**-H_3), 0.76 – 0.69 (m, 4H, 2 × C**12**-H_2); ^{13}C NMR (DMSO-d_6, 126 MHz, 110 °C): δ 173.1 (C**10**), 158.9 (C**2**), 138.0 (C**14**), 131.9 (C**4**), 127.6, 126.1 (C**15** and C**16**), 126.0 (C**17**), 114.3 (C**5**), 113.7 (C**3**), 91.1 (C**7**), 82.7 (C**6**), 54.8 (C**1**), 49.8 (C**13**), 37.3 (C**8**), 30.4 (C**11**), 28.1 (C**9**), 7.2 (C**12**); HRMS: (ESI$^+$) calculated for $C_{23}H_{26}NO_2$: 348.1958, found $[M+H]^+$: 348.1953.

1-Benzyl-4-(4-methoxyphenyl)-3,3-dimethyl-1,6,7,7a-tetrahydro-2*H*-indole-2,5(3*H*)-dione (4e)

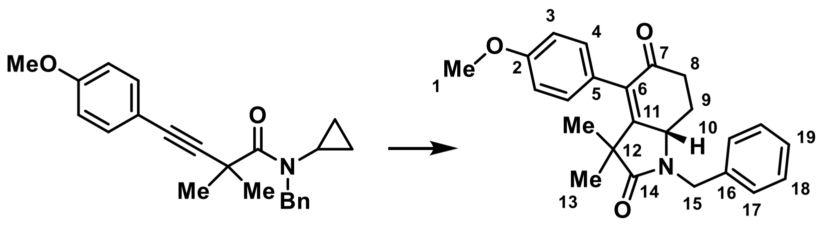

3e **4e**

General procedure C: Amide **3e** (34.7 mg, 0.10 mmol) was employed. Flash column chromatography (40% EtOAc/hexane) afforded the title compound **(*S*)-4e** (22.1 mg, 59%) as a colorless oil; ν_{max} / cm^{-1}: 2931 (w), 1694 (s), 1673 (s), 1510 (s), 1418 (m), 1243 (s), 1173 (s), 726 (s); ^{1}H NMR ($CDCl_3$, 400 MHz): δ 7.39 – 7.23 (m, 5H, C**19**-H, 2 × C**17**-H and 2 × C**18**-H), 6.96

– 6.85 (m, 4H, 2 × C**3**-H and 2 × C**4**-H), 5.09 (d, *J* = 14.9 Hz, 1H, C**15**-H_aH_b), 4.20 (dd, *J* = 11.7, 4.4 Hz, 1H, C**10**-H), 4.09 (d, *J* = 14.9 Hz, 1H, C**15**-H_aH_b), 3.80 (s, 3H, C**1**-H_3), 2.71 (ddd, *J* = 17.8, 4.4, 2.3 Hz, 1H, C**8**-H_aH_b), 2.51 – 2.43 (m, 1H, C**9**-H_aH_b), 2.35 (ddd, *J* = 17.8, 14.2, 4.9 Hz, 1H, C**8**-H_aH_b), 1.85 – 1.72 (m, 1H, C**9**-H_aH_b), 1.33 (s, 3H, C**13**-H_3), 0.85 (s, 3H, C**13'**-H_3); ^{13}C NMR ($CDCl_3$, 101 MHz): δ 197.1 (C**7**), 177.5 (C**14**), 162.6 (C**11**), 159.4 (C**2**), 136.0 (C**16**), 135.7 (C**6**), 131.0 (2 signals, C**4** and C**19**), 129.1, 128.1 (C**17** and C**18**), 126.1 (C**5**), 113.6 (C**3**), 55.8 (C**10**), 55.3 (C**1**), 46.0 (C**12**), 44.1 (C**15**), 35.8 (C**8**), 28.4 (C**9**), 26.2 (C**13**), 22.0 (C**13'**); HRMS: (ESI$^+$) calculated for $C_{24}H_{25}NNaO_3$: 398.1727, found $[M+Na]^+$: 398.1725. The enantiopurity of this compound was determined by chiral SFC against a racemic standard.

$[\alpha]_D^{23.9}$ = -55.1 (c = 0.13, $CHCl_3$).

Chiral SFC: (DAICEL CHIRALPAK-IA column (25 cm), CO_2:MeOH 88:12, 2 mL/min, 140 bars, 40 °C). Retention times: 7.7 minutes (minor), 8.4 minutes (major), e.r. = 87:13.

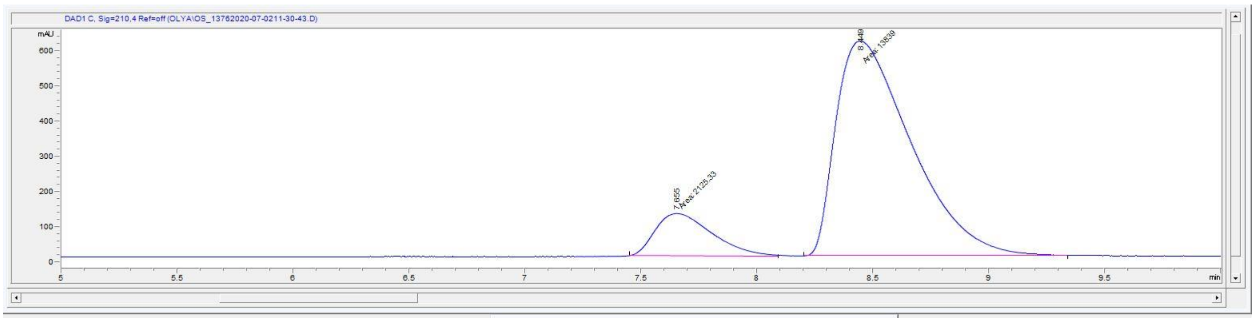

#	Time	Area	Height	Width	Area%	Symmetry
1	7.655	2125.3	122.3	0.2897	13.313	0.536
2	8.449	13839	612.2	0.3768	86.687	0.483

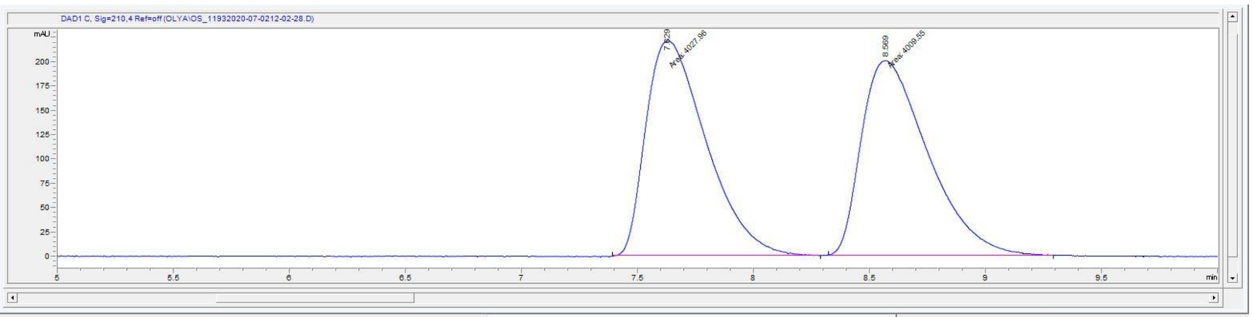

#	Time	Area	Height	Width	Area%	Symmetry
1	7.629	4028	221.7	0.3029	50.115	0.534
2	8.569	4009.5	200.4	0.3334	49.885	0.546

General procedure D: Amide **3e** (34.7 mg, 0.10 mmol) was employed. Flash column chromatography (30% EtOAc/hexane) afforded the title compound **4e** (27.1 mg, 72%) as a colorless solid; m.p. 160-161 °C (DCM/hexane). The structure and *relative* stereochemistry of this compound was determined unambiguously by X-ray crystallography.

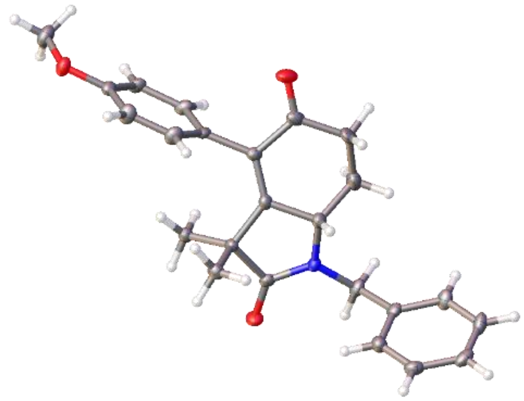

Crystal image of **4e** generated in Olex2,[6] ellipsoids at 50% probability, the water solvent molecule omitted for clarity; Crystal data for **4e**: $C_{24}H_{27}NO_4$, MW = 393.46, orthorhombic, space group $P2_1/c$, a = 9.4740(7) Å, b = 11.4261(8) Å, c = 19.3296(16) Å, V = 2037.0(3) $Å^3$, α = 90.00°, β = 103.220(5)°, γ = 90.00°, Z = 4, D_c = 1.283 g/cm^3 , Mo-Kα radiation, λ = 0.71073 Å, μ = 0.087 mm^{-1}, T = 100 K; colorless plate, crystal size 0.419 × 0.344 × 0.149 mm^3, Bruker Apex II diffractometer, 18092 reflections were collected, 4836 were unique, R_{int} = 0.0727; refinement on F^2 gave R_1 = 0.0583 and wR_2 = 0.1673, GOF = 1.012 for 268 refined parameters.

***N*-Benzyl-4-(4-chlorophenyl)-*N*-cyclopropyl-2,2-dimethylbut-3-ynamide (3f)**

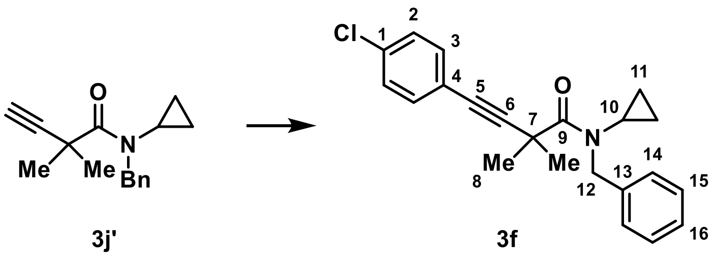

3j'   3f

General procedure B: Aminocyclopropane **3j'** (200.0 mg, 0.83 mmol) and 1-chloro-4-iodobenzene (217.0 mg, 0.91 mmol) were employed and the reaction was stirred for 2 h at r.t. Flash column chromatography (10% EtOAc/hexane) afforded alkyne **3f** (261.4 mg, 89%) as an orange solid; m.p. 83-84 °C (DCM/hexane); ν_{max} / cm^{-1}: 2986 (w), 1646 (s), 1490 (m), 1397 (s), 1089 (m), 828 (m); ^1H NMR (DMSO-d_6, 500 MHz, 110 °C): δ 7.35 – 7.29 (m, 4H, 2 × C**2**-H and 2 × C**3**-H), 7.25 – 7.19 (m, 5H, 2 × C**14**-H, 2 × C**15**-H and C**16**-H), 4.78 (s, 2H, C**12**-H_2), 2.98 (br. s, overlapped by water, 1H, C**10**-H), 1.59 (s, 6H, 2 × C**8**-H_3), 0.74 – 0.70 (m, 4H, 2 × C**11**-H_2); ^{13}C NMR (DMSO-d_6, 126 MHz, 110 °C): δ 172.8 (C**9**), 138.0 (C**14**), 132.6 (C**1**), 132.2, 128.0, 127.8, 126.1 (C**2**, C**3**, C**14** and C**15**), 126.1 (C**16**), 121.0 (C**4**), 93.9 (C**6**), 81.7 (C**5**), 49.9 (C**12**), 37.3 (C**7**), 30.5 (C**10**), 28.0 (C**8**), 7.3 (C**11**); HRMS: (ESI^+) calculated for $C_{22}H_{23}{}^{35}ClNO$: 352.1463, found $[M+H]^+$: 352.1471.

1-Benzyl-4-(4-chlorophenyl)-3,3-dimethyl-1,6,7,7a-tetrahydro-2*H*-indole-2,5(3*H*)-dione (4f)

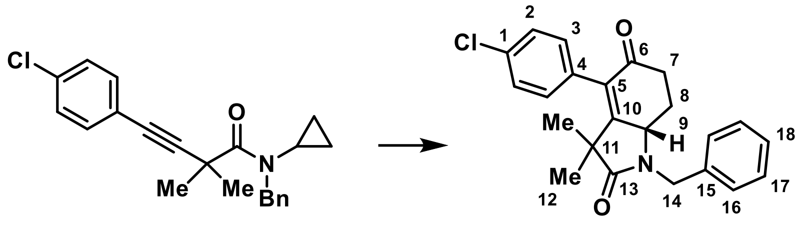

General procedure C: Amide **3f** (35.2 mg, 0.10 mmol) was employed. Flash column chromatography (40% EtOAc/hexane) afforded the title compound **(*S*)-4f** (19.5 mg, 51%) as a colorless solid; m.p. 214-215 °C (DCM/hexane); ν_{max} / cm^{-1}: 2929 (w), 1695 (s), 1674 (s), 1418 (m), 1242 (m), 1088 (m), 728 (s), 702 (s); ^1H NMR ($CDCl_3$, 400 MHz): δ 7.39 – 7.28 (m, 5H, C**18**-H, 2 × C**17**-H and 2 × C**16**-H), 7.28 – 7.23 (m, 2H, 2 × C**2**-H), 6.99 – 6.93 (m, 2H, 2 × C**3**-H), 5.10 (d, *J* = 14.9 Hz, 1H, C**14**-$\underline{H}_aH_b$), 4.21 (dd, *J* = 11.7, 4.4 Hz, 1H, C**9**-H), 4.09 (d, *J* = 14.9 Hz, 1H, C**14**-$H_a\underline{H}_b$), 2.71 (ddd, *J* = 17.8, 4.4, 2.4 Hz, 1H, C**7**-$\underline{H}_aH_b$), 2.52 – 2.45 (m, 1H, C**8**-$\underline{H}_aH_b$), 2.36 (ddd, *J* = 17.8, 14.3, 4.9 Hz, 1H, C**7**-$H_a\underline{H}_b$), 1.80 (dtd, *J* = 14.3, 11.7, 4.4 Hz, 1H, C**8**-$H_a\underline{H}_b$), 1.32 (s, 3H, C**12**-$\underline{H}_3$), 0.85 (s, 3H, C**12'**-$\underline{H}_3$); ^{13}C NMR ($CDCl_3$, 101 MHz): δ 196.5 (C**6**), 177.1 (C**13**), 163.1 (C**10**), 135.9 (C**15**), 135.0 (C**5**), 134.3 (C**4**), 132.5 (C**1**), 131.3 (C**3**), 129.1, 128.5, 128.1, 128.1 (C**2**, C**16**, C**17** and C**18**), 55.8 (C**9**), 46.0 (C**11**), 44.2 (C**14**), 35.8 (C**7**), 28.4 (C**8**), 26.2 (C**12**), 22.1 (C**12'**); HRMS: (ESI^+) calculated for $C_{23}H_{22}{}^{35}ClNNaO_2$: 402.1231, found $[M+Na]^+$: 402.1240.

The structure and *absolute* configuration of this compound was determined unambiguously by X-ray crystallography.

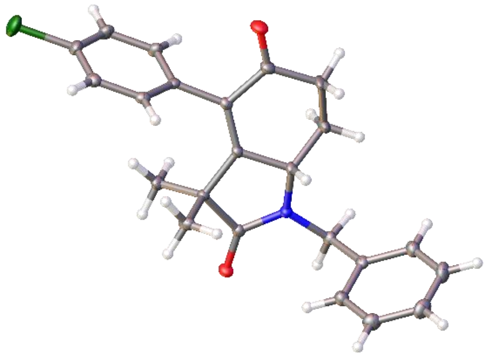

Crystal image of **(*S*)-4f** generated in Olex2,[6] ellipsoids at 50% probability; Crystal data for **(*S*)-4f**: $C_{23}H_{22}ClNO_2$, MW = 379.86, orthorhombic, space group $P2_12_12_1$, a = 8.9513(2) Å, b = 11.5502(3) Å, c = 18.9200(4) Å, V = 1956.13(8) $Å^3$, α = 90.00°, β = 90.00°, γ = 90.00°, Z = 4, D_c = 1.290 g/cm^3 , Mo-Kα radiation, λ = 0.71073 Å, μ = 0.213 mm^{-1}, T = 100 K; colorless block, crystal size

0.467 × 0.378 × 0.256 mm^3, Bruker Apex II diffractometer, 44550 reflections were collected, 5790 were unique, R_{int} = 0.0445; refinement on F^2 gave R_1 = 0.0330 and wR_2 = 0.0822, GOF = 1.030 for 246 refined parameters; flack parameter -0.04(2).

The enantiopurity of this compound was determined by chiral SFC against a racemic standard.

$[\alpha]_D^{24.2}$ = -30.8 (c = 0.26, $CHCl_3$).

Chiral SFC: (DAICEL CHIRALPAK-IB column (25 cm), CO_2:MeOH 88:12, 2 mL/min, 140 bars, 40 °C). Retention times: 14.9 minutes (minor), 15.6 minutes (major), e.r. = 93:7.

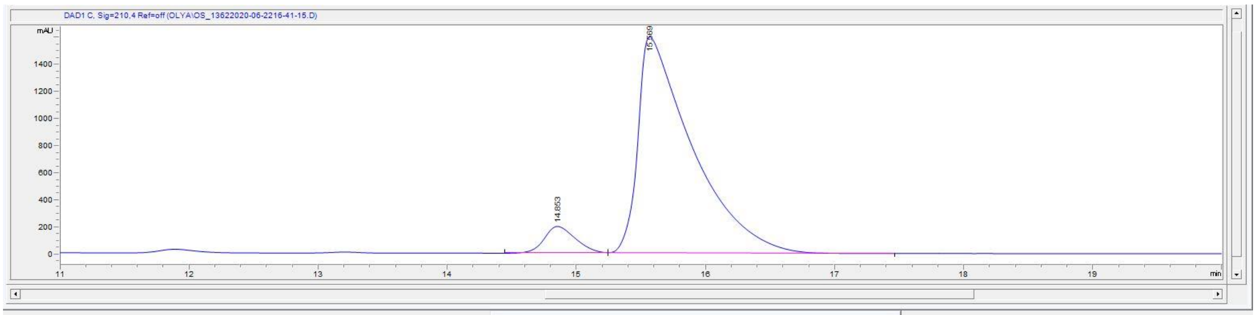

#	Time	Area	Height	Width	Area%	Symmetry
1	14.853	3362	197.6	0.2625	6.708	0.71
2	15.569	46759.6	1594.2	0.3962	93.292	0.266

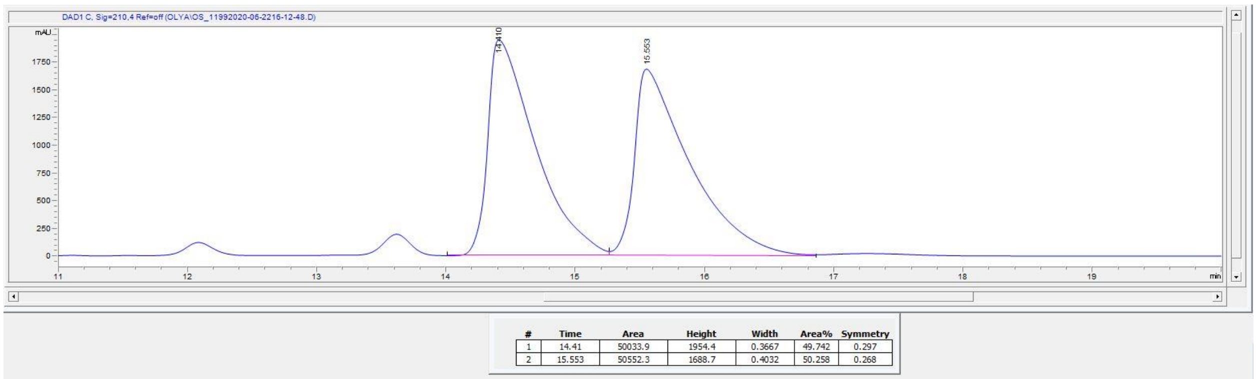

#	Time	Area	Height	Width	Area%	Symmetry
1	14.41	50033.9	1954.4	0.3667	49.742	0.297
2	15.553	50552.3	1688.7	0.4032	50.258	0.268

General procedure D: Amide **3f** (35.2 mg, 0.10 mmol) was employed. Flash column chromatography (30% EtOAc/hexane) afforded the title compound **4f** (29.2 mg, 77%) as a pale yellow oil.

N-Benzyl-N-cyclopropyl-2,2-dimethyl-4-(thiophen-3-yl)but-3-ynamide (3g)

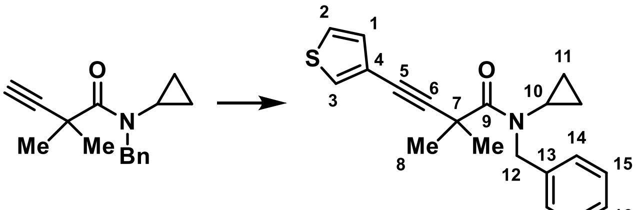

General procedure B: Aminocyclopropane **3j'** (200.0 mg, 0.83 mmol) and 3-bromothiophene (85 µL, 0.91 mmol) were employed and the reaction was stirred for 16 h at 60 °C. Flash column chromatography (10% EtOAc/hexane) afforded alkyne **3g** (263.1 mg, 98%) as an orange solid; m.p. 74-75 °C (DCM/hexane); ν_{max} / cm^{-1}: 2985 (w), 1644 (s), 1396 (s), 1361 (m), 783 (m); ^{1}H NMR (DMSO-d_6, 500 MHz, 110 °C): δ 7.47 – 7.42 (m, 2H, C**2**-H and C**3**-H), 7.34 – 7.20 (m, 5H, 2 × C**14**-H, 2 × C**15**-H and C**16**-H), 6.92 (dd, J = 4.9, 1.3 Hz, 1H, C**1**-H), 4.78 (s, 2H, C**12**-H_2), 3.00 (br. s, overlapped by water, 1H, C**10**-H), 1.58 (s, 6H, 2 × C**8**-H_3), 0.75 – 0.70 (m, 4H, 2 × C**11**-H_2); ^{13}C NMR (DMSO-d_6, 126 MHz, 110 °C): δ 173.0 (C**9**), 138.0 (C**13**), 128.8, 128.1, 127.7, 126.2, 126.1, 125.6 (C**1**, C**2**, C**3**, C**14**, C**15** and C**16**), 121.1 (C**4**), 91.9 (C**6**), 78.3 (C**5**), 49.9 (C**12**), 37.3 (C**7**), 30.5 (C**10**), 28.1 (C**8**), 7.3 (C**11**); HRMS: (ESI$^+$) calculated for $C_{20}H_{22}NOS$: 324.1417, found $[M+H]^+$: 324.1424.

1-Benzyl-3,3-dimethyl-4-(thiophen-3-yl)-1,6,7,7a-tetrahydro-2H-indole-2,5(3H)-dione (4g)

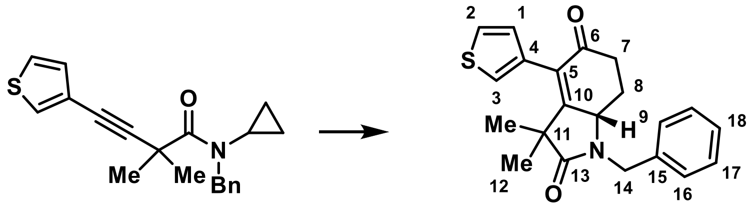

General procedure C: Amide **3g** (32.3 mg, 0.10 mmol) was employed. Flash column chromatography (40% EtOAc/hexane) afforded the title compound **(*S*)-4g** (25.0 mg, 71%) as a colorless oil; ν_{max} / cm^{-1}: 1694 (s), 1673 (s), 1645 (m), 1417 (s), 1241 (m), 1196 (m), 732 (s), 697 (s); ^{1}H NMR ($CDCl_3$, 400 MHz): δ 7.39 – 7.22 (m, 6H, C**2**-H, C**18**-H, 2 × C**16**-H and 2 × C**17**-H), 7.00 (dd, J = 2.9, 1.3 Hz, 1H, C**3**-H), 6.81 (dd, J = 5.0, 1.3 Hz, 1H, C**1**-H), 5.09 (d, J = 15.0 Hz, 1H, C**14**-H_aH_b), 4.20 (dd, J = 11.7, 4.4 Hz, 1H, C**9**-H), 4.08 (d, J = 15.0 Hz, 1H, C**14**-H_aH_b), 2.76 – 2.65 (m, 1H, C**7**-H_aH_b), 2.51 – 2.42 (m, 1H, C**8**-H_aH_b), 2.41 – 2.28 (m, 1H, C**7**-H_aH_b), 1.86 – 1.72 (m, 1H, C**8**-H_aH_b), 1.36 (s, 3H, C**12**-H_3), 0.88 (s, 3H, C**12'**-H_3); ^{13}C NMR ($CDCl_3$, 126 MHz): δ 196.5 (C**6**), 177.4 (C**13**), 163.8 (C**10**), 135.9 (C**15**), 133.6 (C**4**), 131.2 (C**5**), 129.4 (C**2**),

129.1, 128.1, 128.0 (C**16**, C**17** and C**18**), 125.3 (C**1**), 124.7 (C**3**), 56.0 (C**9**), 46.0 (C**11**), 44.1 (C**14**), 35.8 (C**7**), 28.4 (C**8**), 26.3 (C**12**), 21.4 (C**12'**); HRMS: (ESI^+) calculated for $C_{21}H_{21}NNaO_2S$: 374.1185, found $[M+Na]^+$: 374.1196.

The enantiopurity of this compound was determined by chiral SFC against a racemic standard.

$[\alpha]_D^{23.7}$ = -27.4 (c = 0.38, $CHCl_3$).

Chiral SFC: (DAICEL CHIRALPAK-IA column (25 cm), CO_2:MeOH 88:12, 2 mL/min, 140 bars, 40 °C). Retention times: 7.5 minutes (minor), 10.6 minutes (major), e.r. = 93:7.

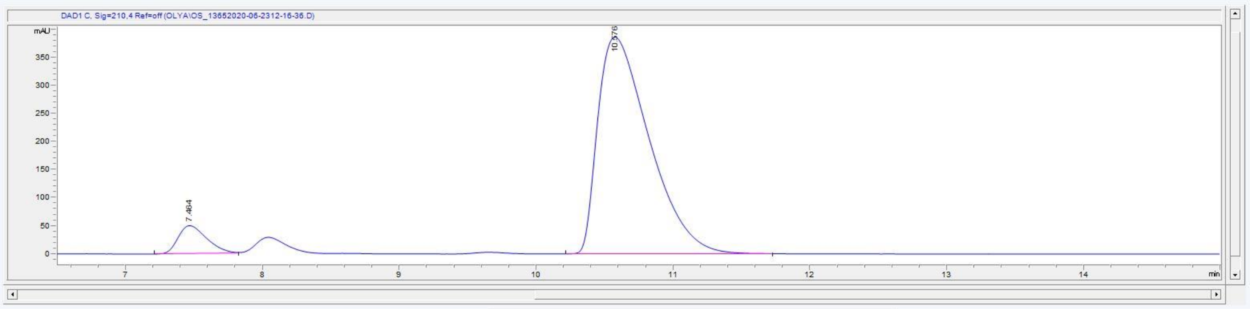

#	Time	Area	Height	Width	Area%	Symmetry
1	7.464	745	50.3	0.2309	6.883	0.618
2	10.576	10079.2	385.3	0.4032	93.117	0.465

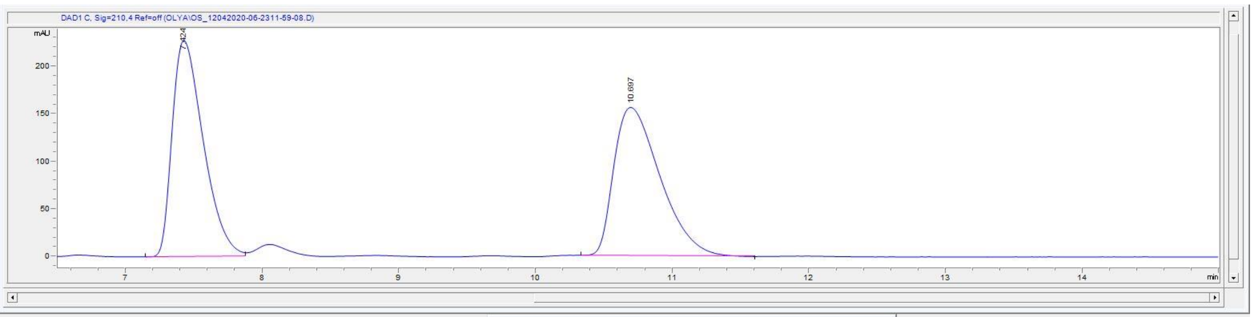

#	Time	Area	Height	Width	Area%	Symmetry
1	7.424	3621.4	228.1	0.2473	49.618	0.561
2	10.697	3677.2	156.1	0.3688	50.382	0.537

General procedure D: Amide **3g** (32.3 mg, 0.10 mmol) was employed. Flash column chromatography (40% EtOAc/hexane) afforded the title compound **4g** (30.9 mg, 88%) as a yellow oil.

***N*-Benzyl-*N*-cyclopropyl-2,2-dimethyl-4-(naphthalen-1-yl)but-3-ynamide (3h)**

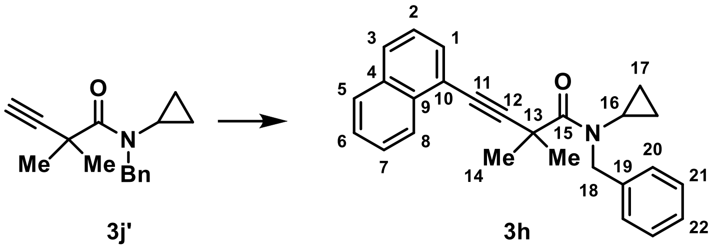

3j'   3h

General procedure B: Aminocyclopropane **3j'** (200.0 mg, 0.83 mmol) and 1-iodonaphthalene (231.2 mg, 0.91 mmol) were employed and the reaction was stirred for 3 h at r.t. Flash column chromatography (10% EtOAc/hexane) afforded alkyne **3h** (277.1 mg, 91%) as an orange oil; ν_{max} / cm^{-1}: 2984 (w), 1643 (s), 1394 (s), 1165 (m), 799 (s), 774 (s); ^{1}H NMR (DMSO-d_6, 500 MHz, 110 °C): δ 8.15 (br. d, *J* = 8.3 Hz, 1H, C**8**-H), 7.95 – 7.91 (m, 1H, C**5**-H), 7.89 (br. d, *J* = 8.0 Hz, 1H, C**3**-H), 7.61 – 7.53 (m, 2H, C**1**-H and C**7**-H), 7.47 – 7.40 (m, 2H, C**2**-H and C**6**-H), 7.30 – 7.17 (m, 5H, 2 × C**20**-H, 2 × C**21**-H and C**22**-H), 4.83 (s, 2H, C**18**-H_2), 3.11 (br. s, 1H, C**16**-H), 1.72 (s, 6H, 2 × C**14**-H_3), 0.80 – 0.70 (m, 4H, 2 × C**17**-H_2); ^{13}C NMR (DMSO-d_6, 126 MHz, 110 °C): δ 173.0 (C**15**), 138.0 (C**19**), 132.4, 132.2 (C**4** and C**9**), 129.4, 128.0, 127.8, 127.7, 126.4, 126.2, 126.1, 125.9, 124.8, 124.7 (C**1**, C**2**, C**3**, C**5**, C**6**, C**7**, C**8**, C**20**, C**21** and C**22**), 119.6 (C**10**), 97.8 (C**12**), 81.0 (C**11**), 49.8 (C**18**), 37.8 (C**13**), 30.6 (C**16**), 28.3 (C**14**), 7.3 (C**17**); HRMS: (ESI^+) calculated for $C_{26}H_{26}NO$: 368.2009, found $[M+H]^+$: 368.2013.

1-Benzyl-3,3-dimethyl-4-(naphthalen-1-yl)-1,6,7,7a-tetrahydro-2*H*-indole-2,5(3*H*)-dione (4h)

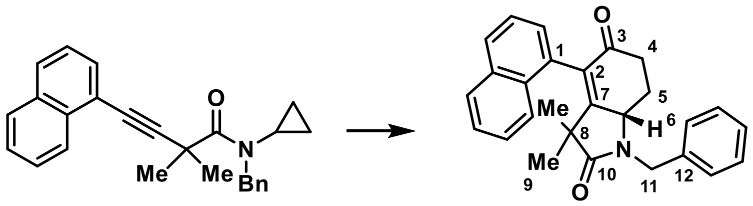

3h   4h

General procedure C: Amide **3h** (36.7 mg, 0.10 mmol) was employed. Flash column chromatography (40% EtOAc/hexane) afforded the title compound **(*S*)-4h** (12.8 mg, 32%) as a colorless oil. The product was obtained as a mixture of atropisomers (**4h** and **4h'**, *A*:*B*, 1:2) due to restricted rotation around the C**1**-C**2** bond. ν_{max} / cm^{-1}: 1696 (s), 1673 (s), 1644 (m), 1418 (m), 1242 (m), 801 (m), 777 (m), 733 (m), 709 (m); ^{1}H NMR ($CDCl_3$, 400 MHz): δ 7.89 – 7.84 (m, 4H, 2 × Ar-H, *A*+*B*), 7.51 – 7.28 (m, 18H, 9 × Ar-H, *A*+*B*), 7.18 (d, *J* = 7.0 Hz, 1H, Ar-H, *B*), 7.14 (d,

J = 7.2 Hz, 1H, Ar-H, *A*), 5.17 (d, J = 14.9 Hz, 1H, C**11**-H_aH_b, *B*), 5.17 (d, J = 14.9 Hz, C**11**-H_aH_b, *A*), 4.40 – 4.28 (m, 2H, C**6**-H, *A*+*B*), 4.16 – 4.09 (m, 2H, C**11**-H_aH_b, *A*+*B*), 2.89 – 2.75 (m, 2H, C**4**-H_aH_b, *A*+*B*), 2.66 – 2.53 (m, 2H, C**5**-H_aH_b, *A*+*B*), 2.52 – 2.40 (m, 2H, C**4**-H_aH_b, *A*+*B*), 2.09 – 1.89 (m, 2H, C**5**-H_aH_b, *A*+*B*), 1.36 (s, 3H, C**9**-H_3, *B*), 0.98 (s, 3H, C**9**-H_3, *A*), 0.82 (s, 3H, C**9'**-H_3, *A*), 0.56 (s, 3H, C**9'**-H_3, *B*); ^{13}C NMR ($CDCl_3$, 126 MHz): δ 196.6 (C**3**, *A*), 196.3 (C**3**, *B*), 177.5 (C**10**, *A*), 177.4 (C**10**, *B*), 164.0 (C**7**, *B*), 163.7 (C**7**, *A*), 136.0 (C**12**, *A*), 136.0 (C**12**, *B*), 134.7, 134.2, 133.7, 133.4, 132.6, 132.4, 132.0, 131.3, 129.2, 129.0, 128.7, 128.6, 128.2, 128.2, 128.2, 128.1, 127.8, 126.5, 126.1, 126.0, 125.5, 125.3, 125.1, 125.0 (2 × Ar-C, 10 × Ar-CH, C**1** and C**2**, *A*+*B*), 56.1 (C**6**, *B*), 55.9 (C**6**, *A*), 46.2 (C**8**, *A*), 46.1 (C**8**, *B*), 44.2 (C**11**, *B*), 44.2 (C**11**, *A*), 36.0 (C**4**, *A*), 35.9 (C**4**, *B*), 28.6 (C**5**, *B*), 28.3 (C**5**, *A*), 26.0 (C**9**, *B*), 23.8 (C**9**, *A*), 23.1 (C**9'**, *A*), 20.7 (C**9'**, *B*); HRMS: (ESI$^+$) calculated for $C_{27}H_{26}NO_2$: 396.1958, found $[M+H]^+$: 396.1968.

The enantiopurity of this compound was determined by chiral SFC against a racemic standard.

$[\alpha]_D^{23.8}$ = -6.93 (c = 0.47, $CHCl_3$, mixture of atropisomers).

Chiral SFC: (DAICEL CHIRALPAK-IA column (25 cm), CO_2:MeOH 88:12, 2 mL/min, 140 bars, 40 °C). Retention times: major diastereomer – 11.2 minutes (minor), 16.0 minutes (major), e.r. = 87:13; minor diastereomer – 10.3 minutes (minor), 14.0 minutes (major), e.r. = 90:10.

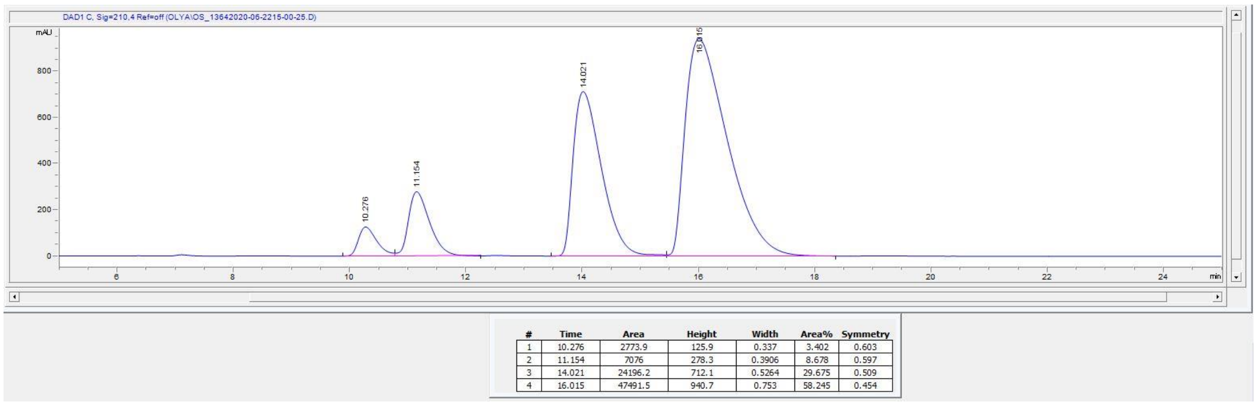

#	Time	Area	Height	Width	Area%	Symmetry
1	10.276	2773.9	125.9	0.337	3.402	0.603
2	11.154	7076	278.3	0.3906	8.678	0.597
3	14.021	24196.2	712.1	0.5264	29.675	0.509
4	16.015	47491.5	940.7	0.753	58.245	0.454

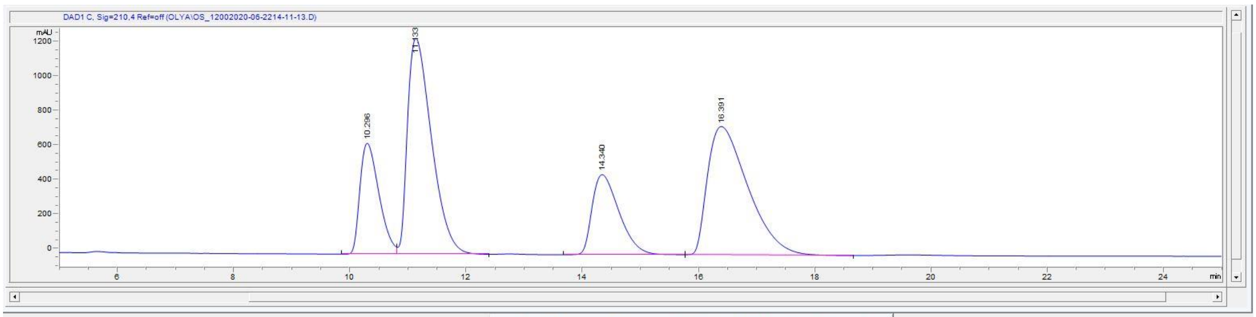

#	Time	Area	Height	Width	Area%	Symmetry
1	10.296	14644.3	640.2	0.3589	14.397	0.582
2	11.133	35896.9	1246.6	0.45	35.290	0.499
3	14.34	15094.4	463.3	0.5001	14.839	0.551
4	16.391	36083.4	742.9	0.7407	35.474	0.485

General procedure D: Amide **3h** (36.7 mg, 0.10 mmol) was employed. Flash column chromatography (30-40% EtOAc/hexane) afforded the title compound **4h** (20.7 mg, 52%) as a colorless oil. The product was obtained as a mixture of atropisomers (**4h** and **4h',** *A*:*B*, 1:2.6).

N-Benzyl-*N*-cyclopropyl-2,2-dimethylpent-3-ynamide (3i)

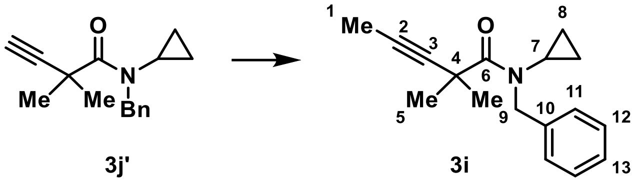

To a solution of terminal alkyne **3j'** (200.0 mg, 0.83 mmol) in THF (0.1 M) at -78 °C was added *n*-BuLi (1.00 mmol, 1.5 M in hexanes) and the solution was stirred for 1 h under nitrogen. Methyl iodide (103 μL, 1.66 mmol) was added and the reaction was slowly warmed to r.t. and stirred for 16 h. Sat. aq. NH_4Cl (20 mL) was added and the solution was extracted with EtOAc (3 × 10 mL). The organic extracts were combined, washed with brine (20 mL), dried over $MgSO_4$ and concentrated *in vacuo*. Flash column chromatography (10% EtOAc/hexane) afforded alkyne **3i** (144.5 mg, 68%) as a colorless oil; ν_{max} / cm^{-1}: 2983 (m), 2935 (m); 1643 (s), 1454 (m), 1394 (s), 1237 (s), 1167 (m), 1027 (m), 1001 (s), 727 (s), 670 (s); ^{1}H NMR (DMSO-d_6, 500 MHz, 110 °C): δ 7.36 – 7.19 (m, 5H, 2 × C**11**-H, 2 × C**12**-H and C**13**-H), 4.75 (s, 2H, C**9**-H_2), 2.96 (br. s, 1H, C**7**-H), 1.63 (s, 3H, C**1**-H_3), 1.45 (s, 6H, 2 × C**5**-H_3), 0.70 – 0.66 (m, 4H, 2 × C**8**-H_2); ^{13}C NMR (DMSO-d_6, 126 MHz, 110 °C): δ 173.5 (C**6**), 138.2 (C**10**), 127.7, 126.1 (C**11** and C**12**), 126.0 (C**13**), 82.2 (C**3**), 78.7 (C**2**), 49.7 (C**9**), 36.7 (C**7**), 30.4 (C**4**), 28.2 (C**5**), 7.0 (C**8**), 2.3 (C**1**); HRMS: (ESI$^+$) calculated for $C_{17}H_{22}NO$: 256.1696, found $[M+H]^+$: 256.1691.

1-Benzyl-3,3,4-trimethyl-1,6,7,7a-tetrahydro-2*H*-indole-2,5(3*H*)-dione (4i)

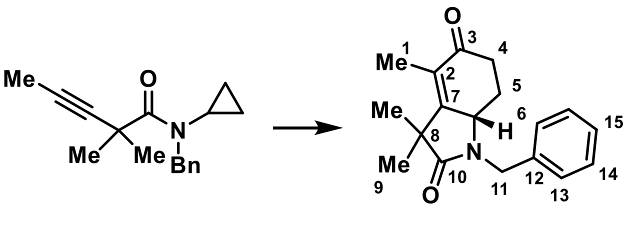

General procedure C: Amide **3i** (25.5 mg, 0.10 mmol) was employed. Flash column chromatography (50% EtOAc/hexane) afforded the title compound **(*S*)-4i** (19.9 mg, 70%) as a colorless oil; ν_{max} / cm^{-1}: 1698 (s), 1670 (s), 1652 (s), 1419 (m), 1316 (m), 1246 (m), 704 (m); ^{1}H

NMR ($CDCl_3$, 400 MHz): δ 7.35 – 7.19 (m, 5H, C**15**-H, 2 × C**13**-H and 2 × C**14**-H), 5.01 (d, *J* = 15.0 Hz, 1H, C**11**-$\underline{H}_aH_b$), 4.10 (d, *J* = 15.0 Hz, 1H, C**11**-$H_a\underline{H}_b$), 4.02 – 3.95 (m, 1H, C**6**-H), 2.59 (ddd, *J* = 18.0, 4.6, 2.1 Hz, 1H, C**4**-$\underline{H}_aH_b$), 2.38 – 2.29 (m, 1H, C**5**-$\underline{H}_aH_b$), 2.20 (ddd, *J* = 18.0, 14.1, 5.0 Hz, 1H, C**4**-$H_a\underline{H}_b$), 1.86 (d, *J* = 2.1 Hz, 3H, C**1**-$\underline{H}_3$), 1.68 – 1.56 (m, 1H, C**5**-$H_a\underline{H}_b$), 1.54 (s, 3H, C**9**-$\underline{H}_3$), 1.34 (s, 3H, C**9'**-$\underline{H}_3$); ^{13}C NMR ($CDCl_3$, 101 MHz): δ 197.5 (C**3**), 177.8 (C**10**), 160.5 (C**7**), 136.0 (C**12**), 129.7 (C**2**), 129.0, 128.0, 128.0 (C**13**, C**14** and C**15**), 56.0 (C**6**), 45.3 (C**8**), 44.2 (C**11**), 35.5 (C**4**), 28.4 (C**5**), 23.1 (C**9**), 22.8 (C**9'**), 10.9 (C**1**); HRMS: (ESI^+) calculated for $C_{18}H_{21}NNaO_2$: 306.1465, found $[M+Na]^+$: 306.1470.

The enantiopurity of this compound was determined by chiral SFC against a racemic standard.

$[\alpha]_D^{24.2}$ = -72.1 (c = 0.23, $CHCl_3$).

Chiral SFC: (DAICEL CHIRALPAK-IB column (25 cm), CO_2:MeOH 94:6, 2 mL/min, 140 bars, 40 °C). Retention times: 11.9 minutes (minor), 12.5 minutes (major), e.r. = 86:14.

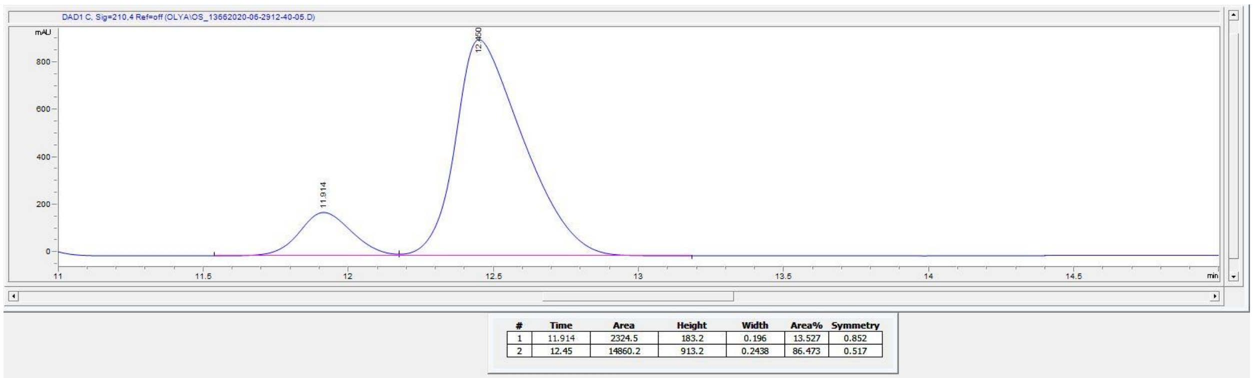

#	Time	Area	Height	Width	Area%	Symmetry
1	11.914	2324.5	183.2	0.196	13.527	0.852
2	12.45	14860.2	913.2	0.2438	86.473	0.517

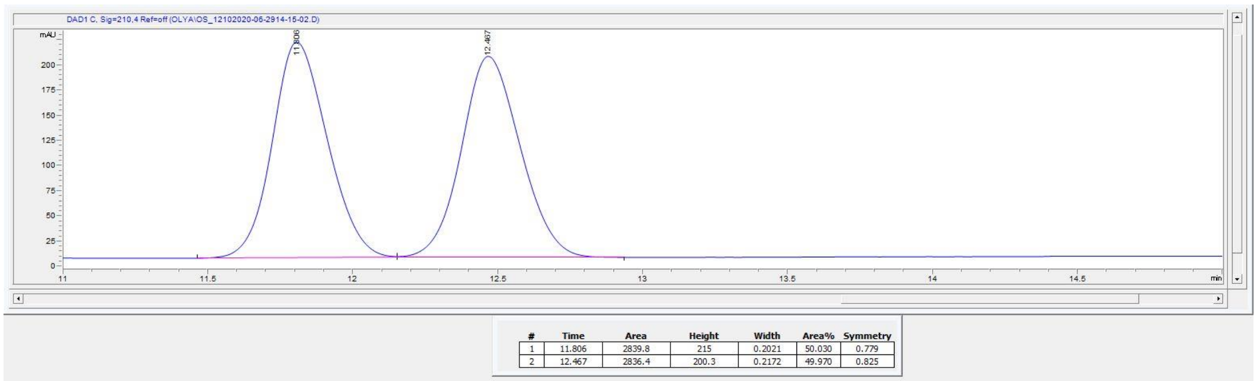

#	Time	Area	Height	Width	Area%	Symmetry
1	11.806	2839.8	215	0.2021	50.030	0.779
2	12.467	2836.4	200.3	0.2172	49.970	0.825

General procedure D: Amide **3i** (25.5 mg, 0.10 mmol) was employed. Flash column chromatography (30-40% EtOAc/hexane) afforded the title compound **4i** (19.1 mg, 67%) as a colorless oil.

Substrate Synthesis and Catalysis for Scheme 2

1-Benzyl-3,3-dimethyl-1,6,7,7a-tetrahydro-2*H*-indole-2,5(3*H*)-dione (4j)

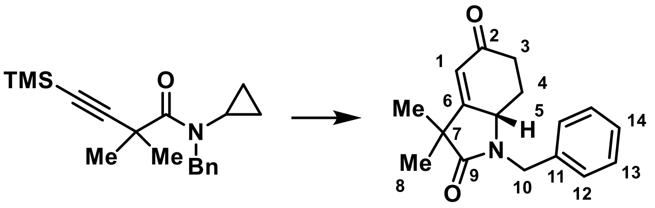

3j **4j**

General procedure C: *In a modification to the general procedure, the reaction was heated to 120 °C.* Amide **3j** (31.4 mg, 0.10 mmol) was employed. Flash column chromatography (50% EtOAc/hexane) afforded the title compound **(*S*)-4j** (18.4 mg, 68%) as a yellow oil; ν_{max} / cm^{-1}: 2967 (w), 1676 (s), 1413 (m), 1233 (m), 1191 (m), 703 (m); ^{1}H NMR ($CDCl_3$, 400 MHz): δ 7.37 – 7.21 (m, 5H, 2 × C**12**-H, 2 × C**13**-H and C**14**-H), 5.90 (d, *J* = 1.2 Hz, 1H, C**1**-H), 5.07 (d, *J* = 15.0 Hz, 1H, C**10**-H_aH_b), 4.09 (ddd, *J* = 11.7, 4.7, 2.3 Hz, 1H, C**5**-H), 4.01 (d, *J* = 15.0 Hz, 1H, C**10**-H_aH_b), 2.58 – 2.50 (m, 1H, C**3**-H_aH_b), 2.39 (dtd, *J* = 11.7, 4.7, 2.3 Hz, 1H, C**4**-H_aH_b), 2.25 (ddd, *J* = 17.6, 14.3, 4.7 Hz, 1H, C**3**-H_aH_b), 1.71 – 1.59 (m, 1H, C**4**-H_aH_b), 1.35 (s, 3H, C**8**-H_3), 1.28 (s, 3H, C**8'**-H_3); ^{13}C NMR ($CDCl_3$, 101 MHz): δ 197.4 (C**2**), 177.0 (C**9**), 169.2 (C**6**), 135.9 (C**11**), 129.1, 129.1, 128.1 (C**12**, C**13** and C**14**), 121.1 (C**1**), 55.1 (C**5**), 44.8 (C**7**), 44.1 (C**10**), 35.7 (C**3**), 28.8 (C**4**), 25.3 (C**8**), 21.5 (C**8'**); HRMS: (ESI$^+$) calculated for $C_{17}H_{19}NNaO_2$: 292.1308, found $[M+Na]^+$: 292.1308.

The enantiopurity of this compound was determined by chiral SFC against a racemic standard.

$[\alpha]_D^{23.6}$ = -51.3 (c = 0.07, $CHCl_3$).

Chiral SFC: (DAICEL CHIRALPAK-IB column (25 cm), CO_2:MeOH 88:12, 2 mL/min, 140 bars, 40 °C). Retention times: 5.2 minutes (minor), 6.1 minutes (major), e.r. = 89:11.

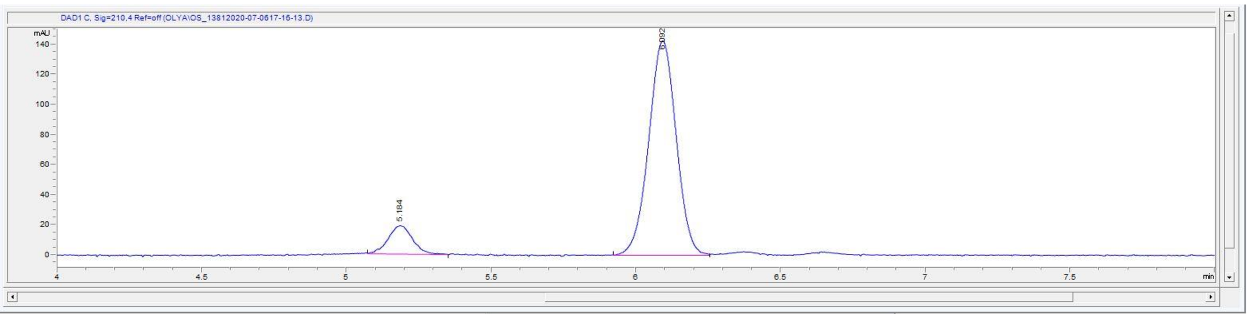

#	Time	Area	Height	Width	Area%	Symmetry
1	5.184	117.8	19.4	0.0729	11.047	0.91
2	6.092	948.9	143.4	0.0967	88.953	1.015

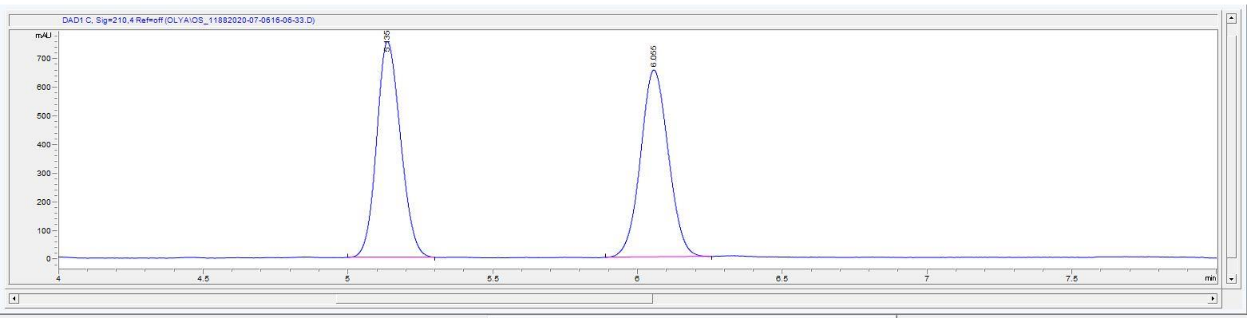

#	Time	Area	Height	Width	Area%	Symmetry
1	5.135	4353.7	753.1	0.0859	49.869	0.802
2	6.055	4376.6	651.5	0.1019	50.131	0.901

General procedure D: Amide **3j** (31.4 mg, 0.10 mmol) was employed. Flash column chromatography (50% EtOAc/hexane) afforded the title compound **4j** (19.3 mg, 72%) as a pale yellow oil.

Note: Substrate **3j'**, bearing a terminal alkyne, provides **4j** in 20% yield[a] under non-enantioselective reaction conditions. TMS-protected alkyne **3j** provides **4j** in 72% yield.

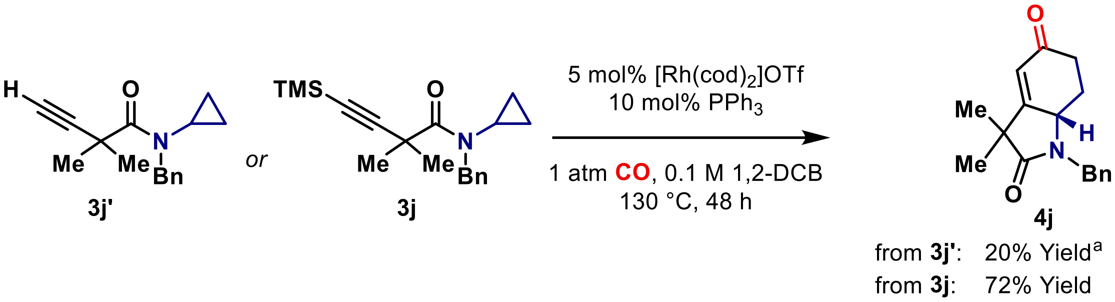

[a]Yield was determined by ^{1}H NMR spectroscopy.

***N*-Benzyl-*N*-cyclopropyl-1-((trimethylsilyl)ethynyl)cyclopropane-1-carboxamide (3k)**

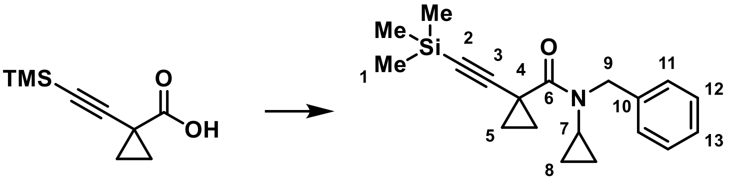

3k

General procedure A: *N*-Benzylcyclopropanamine (0.49 g, 3.30 mmol) and the corresponding acid (0.60 g, 3.30 mmol, prepared according to the literature procedure[7]) were employed and the residue was purified by flash column chromatography (20% EtOAc/hexane) to afford the title compound **3k** (0.51, 50%) as a colorless oil; ν_{max} / cm^{-1}: 2956 (m), 2831 (m), 1633 (m), 1406 (m), 1249 (m), 1026 (s), 841(s), 697 (m); ^{1}H NMR (DMSO-*d*$_6$, 500 MHz, 110 °C): δ 7.35 – 7.19 (m, 5H, 2 × C**11**-H, 2 × C**12**-H and C**13**-H), 4.62 (s, 2H, C**9**-H$_2$), 2.98 – 2.92 (m, 1H, C**7**-H), 1.44 – 1.36 (m, 2H, 2 × C**5**-H$_a$H$_b$), 1.21 – 1.15 (m, 2H, 2 × C**5**-H$_a$H$_b$), 0.86 – 0.76 (m, 4H, 2 × C**8**-H$_2$), 0.09 (s, 9H, 3 × C**1**-H$_3$); ^{13}C NMR (DMSO-*d*$_6$, 126 MHz, 110 °C): 169.5 (C**6**), 137.9 (C**10**), 127.8, 126.5, 126.3 (C**11**, C**12** and C**13**), 107.0 (C**2**), 82.8 (C**3**), 50.0 (C**9**), 30.8 (C**7**), 17.7 (C**4**), 17.2

(C**5**), 8.2 (C**8**), -0.7 (C**1**); HRMS: (ESI$^+$) calculated for $C_{19}H_{26}NOSi$: 312.1767, found [M+H]$^+$: 312.1754.

1'-Benzyl-1',6',7',7a'-tetrahydrospiro[cyclopropane-1,3'-indole]-2',5'-dione (4k)

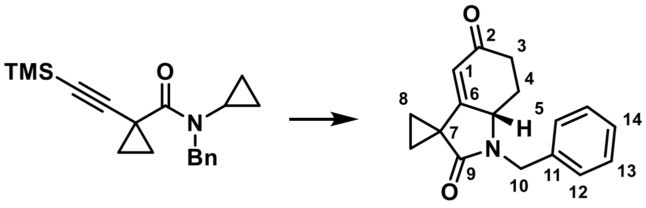

3k **4k**

General procedure C: Amide **3k** (31.2 mg, 0.10 mmol) was employed. Flash column chromatography (50% EtOAc/hexane) afforded the title compound **(*S*)-4k** (15.4 mg, 57%) as a yellow oil; ν_{max} / cm^{-1}: 2925 (w), 1657 (s), 1672 (s), 1435 (m), 1411 (m), 1353 (m), 1231 (m), 1193 (m), 733 (s), 702 (s); ^{1}H NMR ($CDCl_3$, 400 MHz): δ 7.38 – 7.24 (m, 5H, 2 × C**12**-H, 2 × C**13**-H and C**14**-H), 5.54 (dd, *J* = 2.5, 1.0 Hz, 1H, C**1**-H), 4.96 (d, *J* = 15.0 Hz, 1H, C**10**-$\underline{H}_aH_b$), 4.27 (d, *J* = 15.0 Hz, 1H, C**10**-$H_a\underline{H}_b$), 4.24 (ddd, *J* = 11.8, 4.4, 2.4 Hz, 1H, C**5**-H), 2.60 – 2.53 (m, 1H, C**3**-$\underline{H}_aH_b$), 2.38 (dtd, *J* = 11.8, 4.7, 2.4 Hz, 1H, C**4**-$\underline{H}_aH_b$), 2.24 (ddd, *J* = 17.7, 13.9, 4.7 Hz, 1H, C**3**-$H_a\underline{H}_b$), 1.86 – 1.69 (m, 3H, C**4**-$H_a\underline{H}_b$, C**8**-$\underline{H}_aH_b$, C**8'**-$\underline{H}_aH_b$), 1.34 – 1.31 (m, 2H, C**8**-$H_a\underline{H}_b$, C**8'**-$H_a\underline{H}_b$); ^{13}C NMR ($CDCl_3$, 101 MHz): δ 196.7 (C**2**), 174.1 (C**9**), 166.7 (C**6**), 136.2 (C**11**), 129.1, 128.2, 128.0 (C**12**, C**13** and C**14**), 116.2 (C**1**), 57.9 (C**5**), 45.0 (C**10**), 36.1 (C**3**), 29.0 (C**4**), 28.0 (C**7**), 23.7 (C**8**), 17.5 (C**8'**); HRMS: (ESI$^+$) calculated for $C_{17}H_{18}NO_2$: 268.1332, found [M+H]$^+$: 268.1332.

The enantiopurity of this compound was determined by chiral SFC against a racemic standard.

$[\alpha]_D^{23.8}$ = -9.0 (c = 0.25, $CHCl_3$).

Chiral SFC: (DAICEL CHIRALPAK-IB column (25 cm), CO_2:MeOH 88:12, 2 mL/min, 140 bars, 40 °C). Retention times: 9.7 minutes (minor), 10.4 minutes (major), e.r. = 90:10.

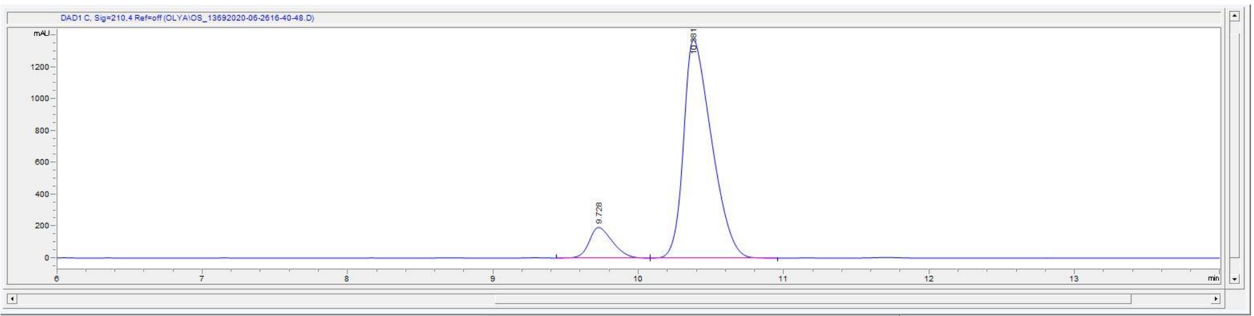

#	Time	Area	Height	Width	Area%	Symmetry
1	9.728	2150.6	192.4	0.1704	10.491	0.67
2	10.381	18348.5	1369.9	0.2023	89.509	0.535

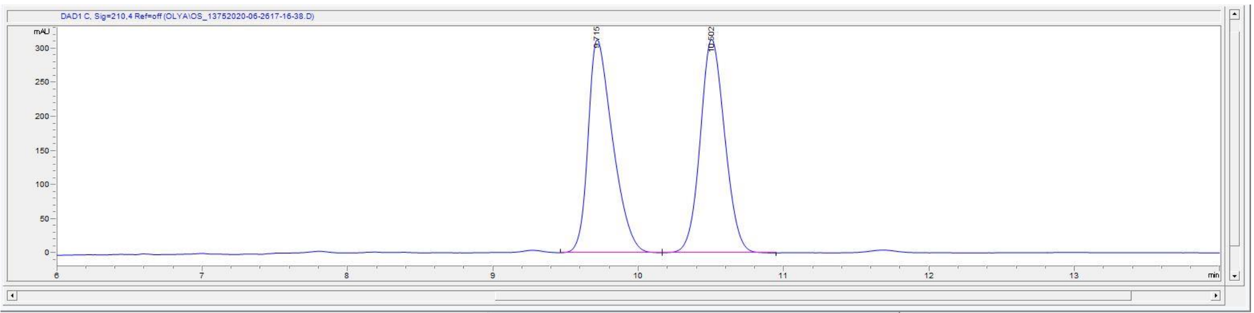

#	Time	Area	Height	Width	Area%	Symmetry
1	9.715	3682	313.8	0.1769	49.947	0.561
2	10.502	3689.8	314.6	0.1788	50.053	0.84

General procedure D: Amide **3k** (31.2 mg, 0.10 mmol) was employed. Flash column chromatography (50% EtOAc/hexane) afforded the title compound **4k** (14.4 mg, 54%) as a pale yellow oil.

***N*-Benzyl-*N*-cyclopropyl-2,2-dimethylbut-3-enamide (3l)**

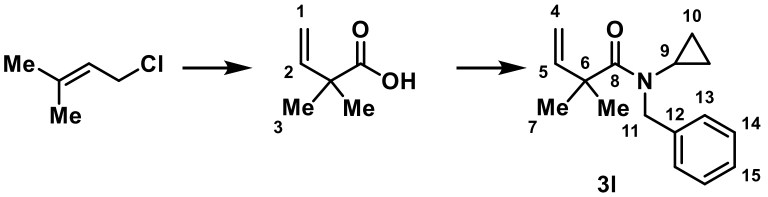

2,2-Dimethylbut-3-enoic acid was prepared according to a literature procedure.[8] 1-Chloro-3-methyl-2-butene was added dropwise to a stirred suspension of magnesium (4.65 g, 191.24 mmol) in dry THF (60 mL) at room temperature. Once an exotherm was observed, the reaction mixture was cooled to 0 °C and the rest of 1-chloro-3-methyl-2-butene (10.78 mL in total, 95.62 mmol) was added dropwise. The reaction was warmed to r.t., stirred for 2 h and the reaction mixture was cannulated into dry ice (approx. 200 g). After stirring for 3 h at r.t., 1.0 M HCl (100 mL) was added and the solution was extracted with Et_2O (3 × 50 mL). The combined organic layer was dried over $MgSO_4$ and concentrated *in vacuo* to afford the desired acid (7.69 g, 70%, 15:1 mixture of regioisomers) as a pale yellow oil which was used in the next step without further purification; ^{1}H

NMR ($CDCl_3$, 400 MHz): δ 6.05 (dd, *J* = 17.4, 10.6 Hz, 1H, C**2**-H), 5.15 (dd, *J* = 17.4, 0.8 Hz, 1H, C**1**-H_{trans}), 5.11 (dd, *J* = 10.6, 0.8 Hz, 1H, C**1**-H_{cis}), 1.33 (s, 6H, 2 × C**3**-H_3).The spectroscopic properties of this compound were consistent with the data available in the literature.[8]

N-Benzyl-*N*-cyclopropyl-2,2-dimethylbut-3-enamide **3l** was prepared according to **general procedure A**. *N*-Benzylcyclopropanamine (1.29 g, 8.76 mmol) and the preceding acid (1.00 g, 8.76 mmol) were employed and the residue was purified by flash column chromatography (10% EtOAc/hexane) to afford the title compound **3l** (1.13 g, 53%) as a colorless oil; ν_{max} / cm^{-1}: 2975 (w), 1630 (s), 1545 (m), 1388 (s), 995 (m), 913 (m), 698 (m); ^1H NMR ($CDCl_3$, 400 MHz): δ 7.34 – 7.13 (m, 5H, 2 × C**13**-H, 2 × C**14**-H and C**15**-H), 6.10 (dd, *J* = 17.6, 10.6 Hz, 1H, C**5**-H), 5.09 (d, *J* = 17.6 Hz, 1H, C**4**-H_{trans}), 5.05 (d, *J* = 10.6 Hz, 1H, C**4**-H_{cis}), 4.58 (s, 2H, C**11**-H_2), 2.69 (br. s, 1H, C**9**-H), 1.37 (s, 6H, 2 × C**7**-H_3), 0.73 – 0.58 (m, 4H, 2 × C**10**-H_2); ^{13}C NMR ($CDCl_3$, 126 MHz): δ 178.5 (C**8**), 143.9 (C**5**), 138.5 (br., C**12**), 128.6, 127.1, 126.9 (C**13**, C**14** and C**15**), 112.2 (C**4**), 51.4 (br., C**11**), 46.0 (C**6**), 30.8 (br., C**9**), 26.9 (C**7**), 8.7 (br., C**10**); HRMS: (ESI^+) calculated for $C_{16}H_{22}NO$: 244.1696, found $[M+H]^+$: 244.1691.

1-Benzyl-3,3-dimethylhexahydro-2*H*-indole-2,5(3*H*)-dione (4l)

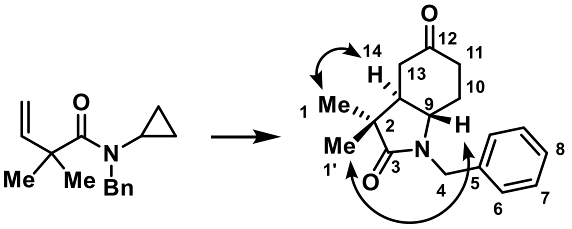

3l 4l

General procedure C: Amide **3l** (24.3 mg, 0.10 mmol) was employed. Flash column chromatography (50% EtOAc/hexane) afforded the title compound **(*R*,*S*)-4l** (17.2 mg, 63%) as a yellow oil; ν_{max} / cm^{-1}: 2952 (m), 1688 (s), 1405 (m), 1267 (m), 1177 (w), 753 (w), 703 (m); ^1H NMR ($CDCl_3$, 400 MHz): δ 7.34 – 7.23 (m, 3H, 2 × C**7**-H and C**8**-H), 7.22 – 7.17 (m, 2H, 2 × C**6**-H), 4.92 (d, *J* = 15.0 Hz, 1H, C**4**-H_aH_b), 4.05 (d, *J* = 15.0 Hz, 1H, C**4**-H_aH_b), 3.26 – 3.15 (m, 1H, C**9**-H), 2.54 – 2.46 (m, 1H, C**11**-H_aH_b), 2.43 – 2.36 (m, 1H, C**13**-H_aH_b), 2.31 – 2.16 (m, 3H, C**10**-H_aH_b, C**11**-H_aH_b, C**13**-H_aH_b), 1.85 – 1.74 (m, 1H, C**14**-H), 1.60 – 1.47 (m, 1H, C**10**-H_aH_b), 1.18 (s, 3H, C**1**-H_3), 0.97 (s, 3H, C**1'**-H_3); ^{13}C NMR ($CDCl_3$, 101 MHz): δ 207.9 (C**12**), 180.5 (C**3**), 136.9 (C**5**), 128.9, 128.0 (C**6** and C**7**), 127.8 (C**8**), 56.5 (C**9**), 50.8 (C**14**), 44.4 (C**4**), 42.7 (C**2**), 39.8 (C**13**), 39.3 (C**11**), 28.4 (C**10**), 23.0 (C**1**), 17.0 (C**1'**); HRMS: (ESI^+) calculated for $C_{17}H_{22}NO_2$: 272.1645, found $[M+H]^+$: 272.1651.

The relative stereochemistry of this compound was corroborated by nOe experiments (as indicated on the compound structure). A strong nOe was observed between C**14**-H and C**1**-H_3. A strong nOe was observed between C**9**-H and C**1'**-H_3. No significant nOe was observed between C**9**-H and C**14**-H.

The enantiopurity of this compound was determined by chiral SFC against a racemic standard. The absolute stereochemistry in **(*R*,*S*)-4l** was assigned by analogy with **(*S*)-4f**.

$[\alpha]_D^{24.2}$ = -14.4 (c = 0.50, $CHCl_3$).

Chiral SFC: (DAICEL CHIRALPAK-IB column (25 cm), CO_2:MeOH 94:6, 2 mL/min, 140 bars, 40 °C). Retention times: 10.8 minutes (minor), 12.1 minutes (major), e.r. = 89:11.

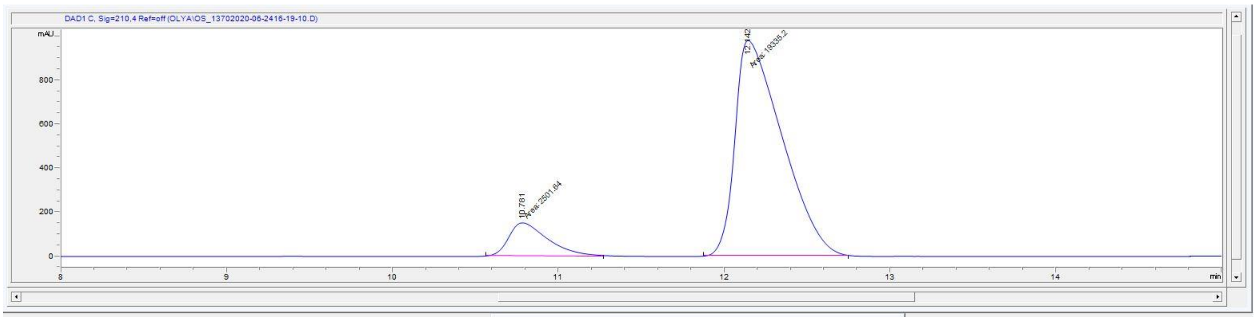

#	Time	Area	Height	Width	Area%	Symmetry
1	10.781	2501.6	150.9	0.2763	11.456	0.509
2	12.142	19335.2	978.4	0.3294	88.544	0.386

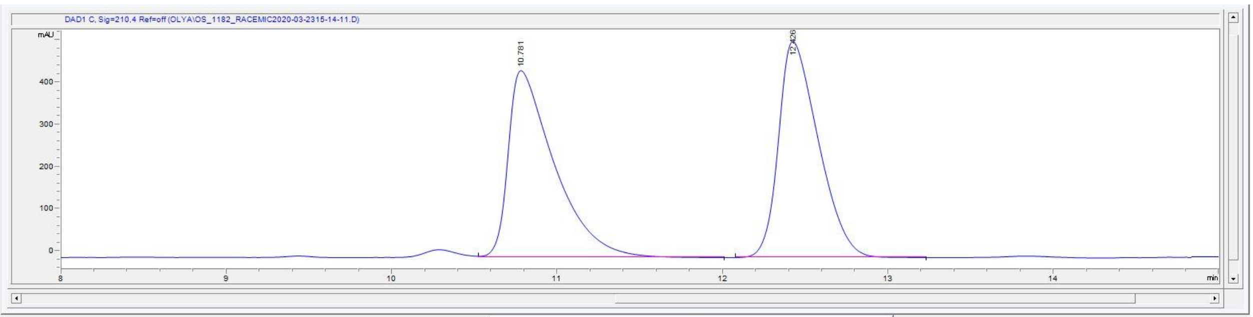

#	Time	Area	Height	Width	Area%	Symmetry
1	10.781	8623	442.6	0.2853	50.124	0.359
2	12.426	8580.2	512.2	0.2574	49.876	0.546

Racemic standard: An oven dried reaction tube, fitted with a magnetic stirrer, was charged with aminocyclopropane substrate **3l** (24.3 mg, 0.10 mmol), $[Rh(cod)Cl]_2$ (2.5 mg, 0.005 mmol) and PPh_3 (5.2 mg, 0.02 mmol). The tube was fitted with a rubber septum and purged with argon. Anhydrous PhCN (1.0 mL) was added and the reaction was sparged with CO for approx. 10 seconds. The tube was heated to 130 °C, under a CO atmosphere (1 balloon) and stirred for 72 h. The mixture was cooled to r.t. and concentrated *in vacuo*. An *in situ* yield (80%) was obtained by using 1,4-dinitrobenzene as an internal standard for ^{1}H NMR spectroscopy. The residue was purified by flash column chromatography (50% EtOAc/hexane) to afford **4l** (19.7 mg, 73%) as a pale yellow oil.

_N,N_,4-Trimethyl-5-oxo-2,3,5,6,7,7a-hexahydro-1_H_-indole-1-carboxamide (2a)

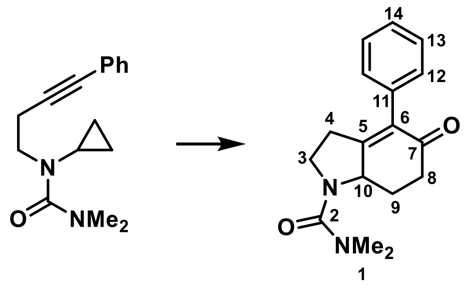

1a 2a

General procedure C: *In a modification to the general procedure, the reaction was heated to 130 °C for 48 h.* Amide **1a** (25.6 mg, 0.10 mmol, prepared according to the literature procedure[9]) was employed. Flash column chromatography (EtOAc) afforded the title compound **2a** (5.3 mg, 24%) as a yellow oil; ^{1}H NMR ($CDCl_3$, 400 MHz): δ 7.39-7.29 (m, 3H, 2 × C**13**-H and C**14**-H), 7.16-7.13 (m, 2H, 2 × C**12**-H), 4.87 (m, 1H, C**10**-H), 3.53-3.44 (m, 2H, C**3**-H_2), 2.88 (s, 6H, 2 × C**1**-H_3), 2.80 (m, 1H, C**4**-H_2), 2.69 (m, 1H, C**4**-H_2), 2.62-2.53 (m, 3H, C**8**-H_2 and 1 × C**9**-H_2), 1.77 (m, 1H, C**9**-H_2). The spectroscopic properties of this compound were consistent with the data available in the literature.[9]

The enantiopurity of this compound was determined by chiral SFC against a racemic standard (prepared according to the literature procedure[9]).

Chiral SFC: (DAICEL CHIRALPAK-IA column (25 cm), CO_2:MeOH 88:12, 2 mL/min, 140 bars, 40 °C). Retention times: 10.8 minutes (minor), 12.1 minutes (major), e.r. = 35:65.

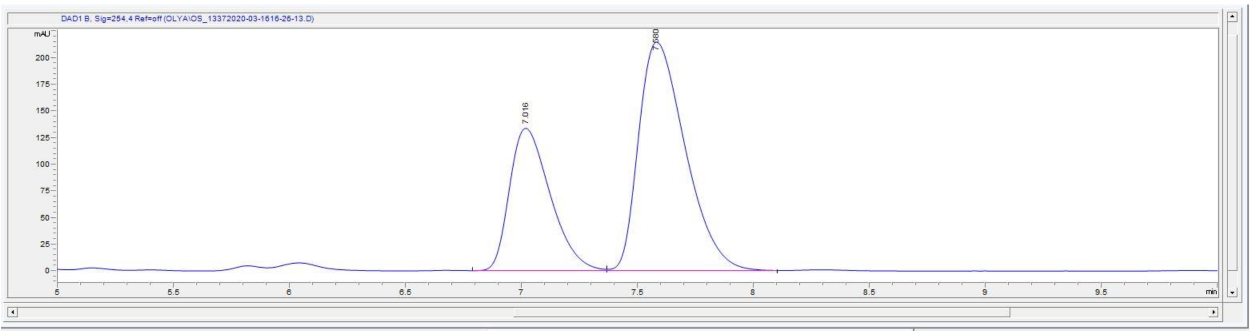

#	Time	Area	Height	Width	Area%	Symmetry
1	7.016	1631.9	134.1	0.188	35.120	0.627
2	7.58	3014.7	215.5	0.2172	64.880	0.593

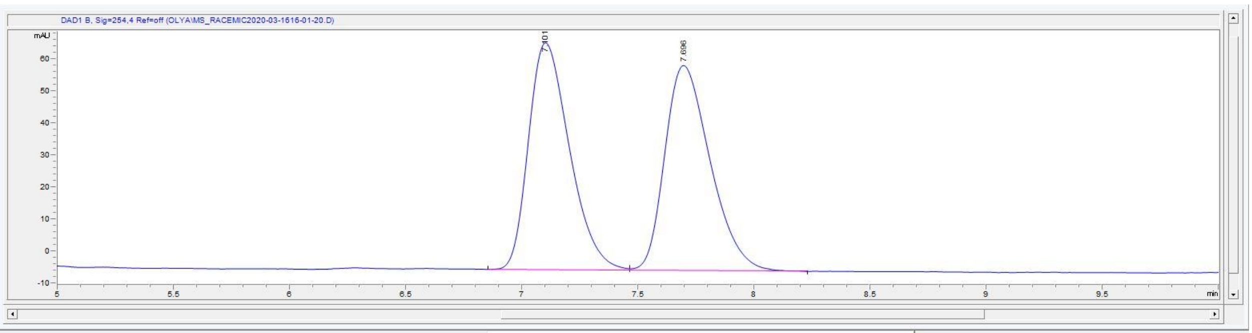

#	Time	Area	Height	Width	Area%	Symmetry
1	7.101	869.6	70.9	0.1892	49.985	0.672
2	7.696	870.2	64	0.2125	50.015	0.664

Substrate Synthesis and Catalysis for Scheme 3

N-Benzyl-2,2-dimethyl-*N*-((1*S**,2*S**)-2-methylcyclopropyl)-4-phenylbut-3-ynamide (3m)

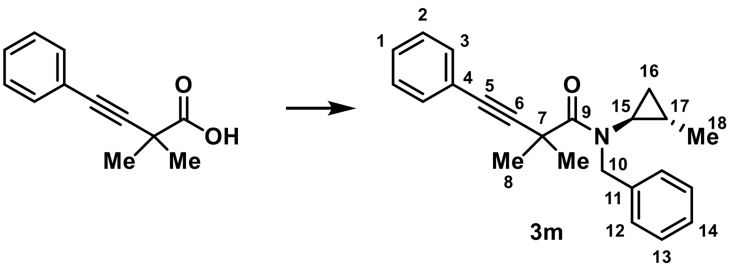

General procedure A: (1*S**,2*S**)-*N*-Benzyl-2-methylcyclopropan-1-amine (0.43 g, 2.66 mmol, prepared according to the literature procedure[10]) and 2,2-dimethyl-4-phenylbut-3-ynoic acid (0.50 g, 2.66 mmol) were employed and the residue was purified by flash column chromatography (5% EtOAc/hexane) to afford the title compound **3m** (0.69 g, 79%) as a colorless solid; m.p. 75-76 °C (DCM/hexane); ν_{max} / cm^{-1}: 2988 (m), 1651 (s), 1492 (m), 1454 (m), 1394 (s), 1169 (m), 1077 (m), 756 (s), 692 (s); ^{1}H NMR (DMSO-d_6, 500 MHz, 110 °C): δ 7.34 – 7.19 (m, 10H, 2 × C**2**-H, 2 × C**3**-H, 2 × C**12**-H, 2 × C**13**-H, C**1**-H and C**14**-H), 4.81 (s, 2H, C**10**-H_2), 2.63 (br. s, 1H, C**15**-H), 1.59 (s, 6H, 2 × C**8**-H_3), 1.11 – 1.02 (m, 1H, C**17**-H), 0.93 (d, *J* = 6.1 Hz, 3H, C**18**-H_3), 0.91 – 0.85 (m, 1H, C**16**-H_aH_b), 0.51 – 0.46 (m, 1H, C**16**-H_aH_b); ^{13}C NMR (DMSO-d_6, 126 MHz, 110 °C): δ 172.8 (C**9**), 137.8 (C**11**), 130.5, 127.8, 127.7, 127.6, 126.3, 126.1 (C**1**, C**2**, C**3**, C**12**, C**13** and C**14**), 122.2 (C**4**), 92.7 (C**6**), 82.8 (C**5**), 50.1 (C**10**), 37.9 (C**15**), 37.2 (C**7**), 28.1 (C**8**), 27.9 (C**8'**), 16.5 (C**18**), 15.1 (C**17**), 14.8 (C**16**); HRMS: (ESI$^+$) calculated for $C_{23}H_{25}NO$: 332.2009, found [M+H]$^+$: 332.2000.

Data for enantioenriched **(*S*,*S*)-3m** (prepared from (*R*)-propylene oxide[11]):

$[\alpha]_D^{23.5}$ = +19.8 (c = 0.33, $CHCl_3$).

Chiral SFC: (DAICEL CHIRALPAK-IB column (25 cm), CO_2:*i*-PrOH 98:2, 2 mL/min, 100 bars, 40 °C). Retention times: 27.0 minutes (major), e.r. > 99:1.

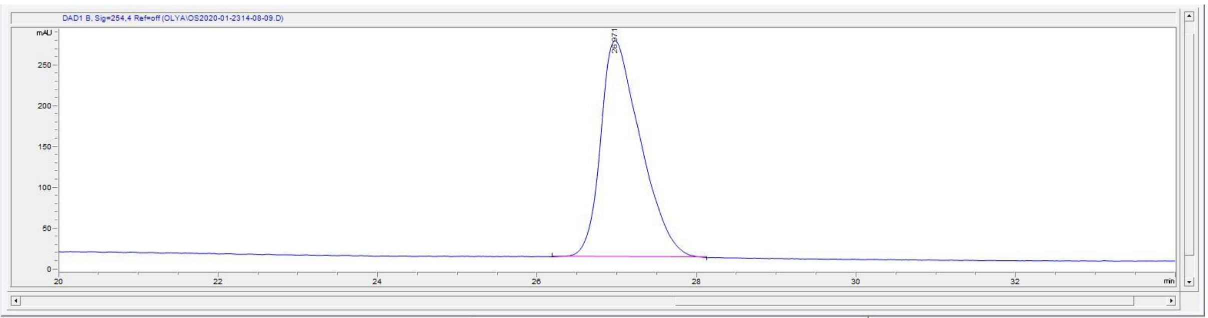

#	Time	Area	Height	Width	Area%	Symmetry
1	26.971	9300.9	266.1	0.4878	100.000	0.559

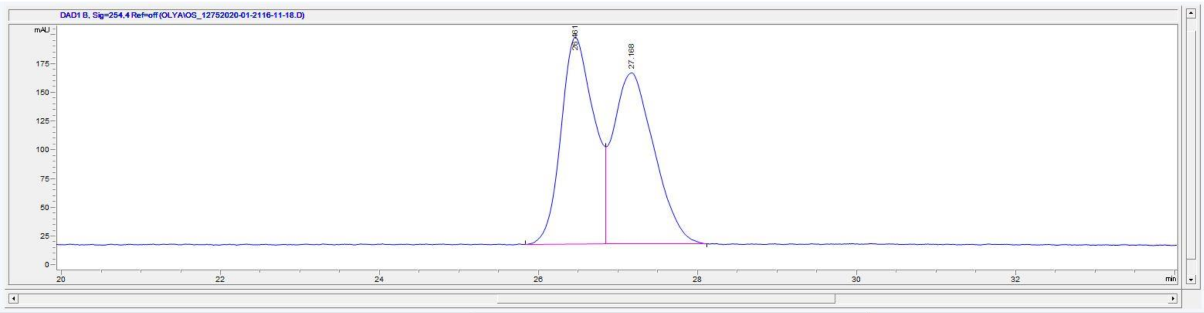

#	Time	Area	Height	Width	Area%	Symmetry
1	26.461	5211.1	180.3	0.4193	49.497	0.762
2	27.168	5317	149.1	0.4861	50.503	0.743

(7*S,7a*R**)-1-Benzyl-3,3,7,7a-tetramethyl-4-phenyl-1,6,7,7a-tetrahydro-2*H*-indole-2,5(3*H*)-dione (4m)**

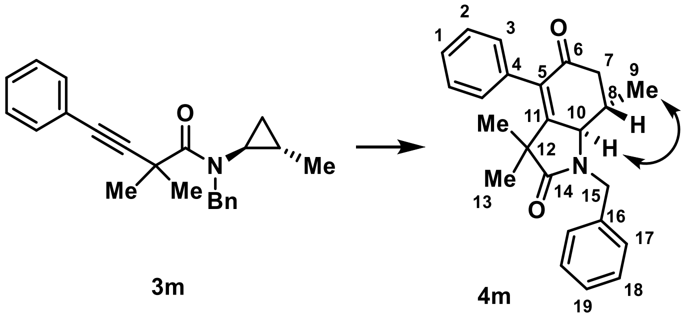

3m **4m**

An oven dried reaction tube, fitted with a magnetic stirrer, was charged with aminocyclopropane substrate **3m** (27.5 mg, 0.10 mmol), $[Rh(cod)Cl]_2$ (2.5 mg, 0.005 mmol) and $P(C_6F_5)_3$ (10.6 mg, 0.02 mmol). The tube was fitted with a rubber septum and purged with argon. Anhydrous PhCN (1.0 mL) was added and the reaction was sparged with CO for ca. 10 seconds. The tube was heated to 150 °C, under a CO atmosphere (1 balloon) and stirred for 72 h. The mixture was cooled to r.t. and concentrated *in vacuo*. The residue was purified by flash column chromatography (10-25% EtOAc/hexane) to afford **4m** (30.3 mg, 84%) as a colorless oil, the product was isolated as a single diastereomer (>15:1 d.r.); ν_{max} / cm^{-1}: 1697 (s), 1673 (s), 1410 (s), 1295 (m), 1239 (m), 919 (m), 730 (s), 700 (s); ^{1}H NMR ($CDCl_3$, 400 MHz): δ 7.41 – 7.27 (m, 6H, 2 × C**2**-H, 2 × C**18**-H, C**1**-H

and C**19**-H), 7.22 – 7.17 (m, 2H, 2 × C**17**-H), 7.08 – 7.00 (m, 2H, 2 × C**3**-H), 5.17 (d, *J* = 15.6 Hz, 1H, C**15**-H_aH_b), 4.50 (d, *J* = 15.6 Hz, 1H, C**15**-H_aH_b), 4.11 (d, *J* = 9.9 Hz, 1H, C**10**-H), 2.59 (dd, *J* = 17.1, 3.6 Hz, 1H, C**7**-H_aH_b), 2.40 – 2.27 (m, 1H, C**8**-H), 2.21 (dd, *J* = 17.1, 13.1 Hz, 1H, C**7**-H_aH_b), 1.34 (s, 3H, C**13**-H_3), 1.24 (d, *J* = 6.3 Hz, 3H, C**9**-H_3), 0.88 (s, 3H, C**13'**-H_3); ^{13}C NMR ($CDCl_3$, 101 MHz): δ 197.0 (C**6**), 179.4 (C**14**), 162.3 (C**11**), 136.6, 136.3 (C**5** and C**16**), 134.0 (C**4**), 130.0 (C**3**), 129.0, 128.1, 128.1, 127.7, 127.4 (C**1**, C**2**, C**17**, C**18** and C**19**), 63.1 (C**10**), 46.6 (C**15**), 45.8 (C**7**), 45.5 (C**12**), 38.1 (C**8**), 26.5 (C**13**), 22.7 (C**13'**), 20.8 (C**9**); HRMS: (ESI^+) calculated for $C_{20}H_{26}NO_2$: 360.1958, found $[M+H]^+$: 360.1949.

The relative stereochemistry of this compound was corroborated by nOe experiments (as indicated on the compound structure). A strong nOe was observed between C**10**-H and C**9**-H_3. No significant nOe was observed between C**10**-H and C**8**-H.

Data for enantioenriched **(*S*,*R*)-4m** (obtained from enantioenriched amide **(*S*,*S*)-3m**):

$[\alpha]_D^{23.4}$ = +61.4 (c = 0.15, $CHCl_3$).

Chiral SFC: (DAICEL CHIRALPAK-IB column (25 cm), CO_2:MeOH 88:12, 2 mL/min, 140 bars, 40 °C). Retention times: 13.7 minutes (minor), 17.3 minutes (major), e.r. > 99:1.

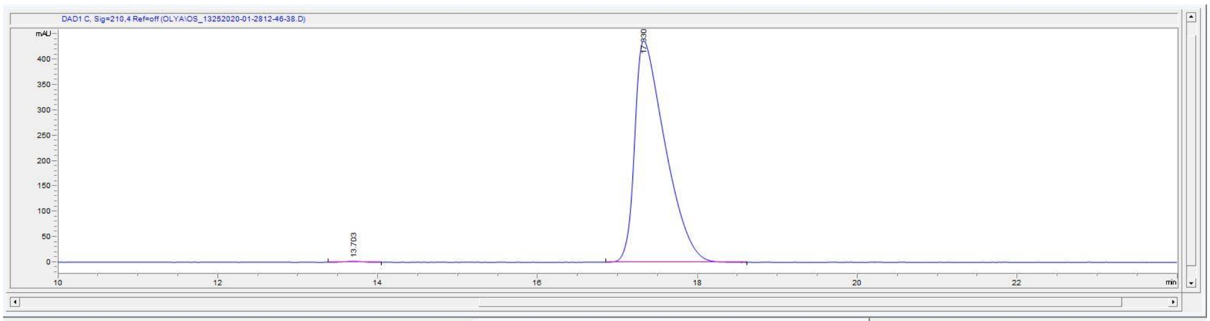

#	Time	Area	Height	Width	Area%	Symmetry
1	13.703	27	1.7	0.1948	0.232	1.082
2	17.33	11595.1	437.3	0.3914	99.768	0.402

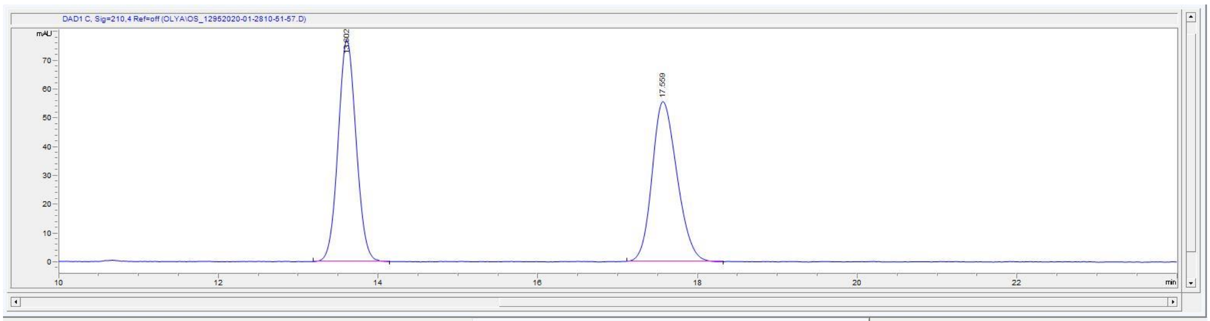

#	Time	Area	Height	Width	Area%	Symmetry
1	13.602	1203.4	76.7	0.245	50.110	0.894
2	17.559	1198.1	55.3	0.3307	49.890	0.749

N-Benzyl-*N*-((1*S**,2*S**)-2-benzylcyclopropyl)-2,2-dimethyl-4-phenylbut-3-ynamide (3n)

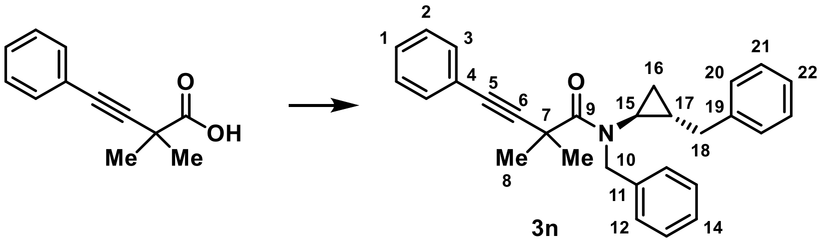

General procedure A: (1*S**,2*S**)-*N*,2-dibenzylcyclopropan-1-amine (0.25 g, 1.05 mmol, prepared according to the literature procedure[10]) and 2,2-dimethyl-4-phenylbut-3-ynoic acid (0.20 g, 1.05 mmol) were employed and the residue was purified by flash column chromatography (5% EtOAc/hexane) to afford the title compound **3n** (0.25 g, 59%) as a pale yellow oil; ν_{max} / cm^{-1}: 2986 (m), 1644 (s), 1495 (m), 1454 (m), 1394 (s), 1167 (m), 1029 (m), 756 (s), 728 (s), 691 (s); ^{1}H NMR (DMSO-d_6, 500 MHz, 110 °C): δ 7.33 – 7.11 (m, 13H, 2 × C**2**-H, 2 × C**3**-H, 2 × C**12**-H, 2 × C**13**-H, 2 × C**21**-H, C**1**-H, C**14**-H and C**22**-H), 7.07 – 7.04 (m, 2H, 2 × C**20**-H), 4.85 (d, *J* = 16.0 Hz, 1H, C**10**-H_aH_b), 4.74 (d, *J* = 16.0 Hz, 1H, C**10**-H_aH_b), 2.85 (br. s, 1H, C**15**-H), 2.75 (dd, *J* = 14.5, 5.3 Hz, 1H, C**18**-H_aH_b), 2.25 (dd, *J* = 14.5, 8.1 Hz, 1H, C**18**-H_aH_b), 1.59 (s, 3H, C**8**-H_3), 1.59 (s, 3H, C**8'**-H_3), 1.41 – 1.33 (m, 1H, C**17**-H), 0.97 – 0.89 (m, 1H, C**16**-H_aH_b), 0.71 – 0.65 (m, 1H, C**16**-H_aH_b); ^{13}C NMR (DMSO-d_6, 126 MHz, 110 °C): δ 172.8 (C**9**), 139.7 (C**19**), 137.8 (C**11**), 130.5, 127.8, 127.8, 127.7, 127.6, 127.5, 126.2, 126.1, 125.3 (C**1**, C**2**, C**3**, C**12**, C**13**, C**14**, C**20**, C**21** and C**22**), 122.1 (C**4**), 92.7 (C**6**), 82.9 (C**5**), 50.0 (C**10**), 37.2 (C**7**), 36.8 (C**18**), 36.7 (C**15**), 28.1 (C**8**), 28.0 (C**8'**), 21.0 (C**17**), 14.1 (C**16**); HRMS: (ESI^+) calculated for $C_{29}H_{30}NO$: 408.2322, found $[M+H]^+$: 408.2323.

(7*S**,7a*R**)-1,7-Dibenzyl-3,3-dimethyl-4-phenyl-1,6,7,7a-tetrahydro-2*H*-indole-2,5(3*H*)-dione (4n)

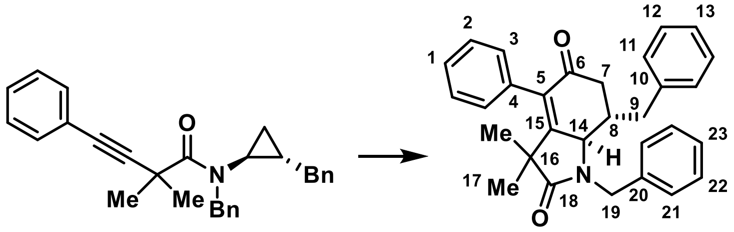

3n 4n

An oven dried reaction tube, fitted with a magnetic stirrer, was charged with aminocyclopropane substrate **3n** (40.8 mg, 0.10 mmol), $[Rh(cod)Cl]_2$ (2.5 mg, 0.005 mmol) and $P(C_6F_5)_3$ (10.6 mg, 0.02 mmol). The tube was fitted with a rubber septum and purged with argon. Anhydrous PhCN

(1.0 mL) was added and the reaction was sparged with CO for ca. 10 seconds. The tube was heated to 150 °C, under a CO atmosphere (1 balloon) and stirred for 72 h. The mixture was cooled to r.t. and concentrated *in vacuo*. The residue was purified by flash column chromatography (10-25% EtOAc/hexane) to afford **4n** (29.4 mg, 67%) as a pale yellow oil, the product was isolated as a single diastereomer (>15:1 d.r.); ν_{max} / cm^{-1}: 1698 (s), 1672 (s), 1495 (m), 1409 (m), 1243 (m), 911 (m), 728 (s), 699 (s); ^1H NMR ($CDCl_3$, 400 MHz): δ 7.44 – 7.14 (m, 11H, 2 × C**2**-$\underline{H}$, 2 × C**11**-$\underline{H}$, 2 × C**12**-$\underline{H}$, 2 × C**22**-$\underline{H}$, C**1**-$\underline{H}$, C**13**-$\underline{H}$ and C**23**-$\underline{H}$), 7.05 – 6.97 (m, 2H, 2 × C**21**-$\underline{H}$), 6.87 – 6.80 (m, 2H, 2 × C**3**-$\underline{H}$), 5.05 (d, *J* = 16.0 Hz, 1H, C**19**-$\underline{H}_aH_b$), 4.83 (d, *J* = 16.0 Hz, 1H, C**19**-$H_a\underline{H}_b$), 4.32 (d, *J* = 9.8 Hz, 1H, C**14**-$\underline{H}$), 3.50 (dd, *J* = 12.7, 3.1 Hz, 1H, 1H, C**9**-$\underline{H}_aH_b$), 2.50 – 2.25 (m, 3H, C**8**-$\underline{H}$, C**7**-$\underline{H}_aH_b$, C**9**-$H_a\underline{H}_b$), 2.10 – 1.98 (m, 1H, C**7**-$H_a\underline{H}_b$), 1.37 (s, 3H, C**17**-$\underline{H}_3$), 0.91 (s, 3H, C**17'**-$\underline{H}_3$); ^{13}C NMR ($CDCl_3$, 101 MHz): δ 196.7 (C**6**), 179.8 (C**18**), 162.1 (C**15**), 137.6 (C**20**), 136.9 (C**5**), 136.6 (C**10**), 133.9 (C**4**), 130.0, 129.2, 129.1, 128.8, 128.2, 128.1, 127.8, 127.2, 126.8 (C**1**, C**2**, C**3**, C**11**, C**12**, C**13**, C**21**, C**22** and C**23**), 62.7 (C**14**), 47.6 (C**19**), 45.4 (C**16**), 44.5 (C**8**), 41.8 (C**7**), 40.1 (C**9**), 26.6 (C**17**), 22.9 (C**17'**); HRMS: (ESI^+) calculated for $C_{30}H_{30}NO_2$: 436.2271, found $[M+H]^+$: 436.2247.

_N_-Benzyl-2,2-dimethyl-_N_-((1_S_*,2_S_*)-2-methylcyclopropyl)but-3-enamide (3o)

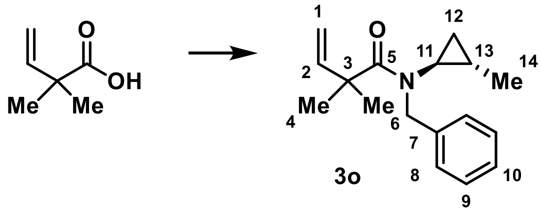

General procedure A: (1*S**,2*S**)-*N*-Benzyl-2-methylcyclopropan-1-amine (0.71 g, 4.38 mmol, prepared according to the literature procedure[10]) and 2,2-dimethylbut-3-enoic acid (0.50 g, 4.38 mmol) were employed and the residue was purified by flash column chromatography (10% EtOAc/hexane) to afford the title compound **3o** (0.61 g, 54%) as a pale yellow oil; ν_{max} / cm^{-1}: 2974 (w), 1644 (s), 1630 (s), 1388 (m), 1173 (m), 912 (m), 998 (s); ^1H NMR ($CDCl_3$, 400 MHz): δ 7.34 – 7.12 (m, 5H, 2 × C**8**-$\underline{H}$, 2 × C**9**-$\underline{H}$ and C**10**-$\underline{H}$), 6.09 (dd, *J* = 17.6, 10.7 Hz, 1H, C**2**-$\underline{H}$), 5.13 – 5.01 (m, 2H, C**1**-$\underline{H}_2$), 4.59 (d, *J* = 15.9 Hz, 1H, C**6**-$\underline{H}_aH_b$), 4.53 (d, *J* = 15.9 Hz, 1H, C**6**-$H_a\underline{H}_b$), 2.29 (br. s, 1H, C**11**-$\underline{H}$), 1.36 (s, 6H, 2 × C**4**-$\underline{H}_3$), 1.00 – 0.87 (m, 4H, C**13**-$\underline{H}$ and C**14**-$\underline{H}_3$), 0.73 (br. s, 1H, C**12**-$\underline{H}_aH_b$), 0.45 – 0.38 (m, 1H, C**12**-$H_a\underline{H}_b$); ^{13}C NMR ($CDCl_3$, 101 MHz): δ 178.2 (C**5**), 144.0 (C**2**), 138.2 (br., C**7**), 128.6, 127.1, 127.1 (C**8**, C**9** and C**10**), 112.4 (C**1**), 51.6 (br.,

C**6**), 45.9 (C**3**), 38.2 (br., C**11**), 26.9 (C**4**), 26.9 (C**4'**), 17.4 (C**14**), 16.3 (br., C**12**), 16.2 (C**13**); HRMS: (ESI$^+$) calculated for $C_{17}H_{24}NO$: 258.1852, found $[M+H]^+$: 258.1844.

(3a*R,7*R**,7a*S**)-1-Benzyl-3,3,7-trimethylhexahydro-2*H*-indole-2,5(3*H*)-dione (4o)**

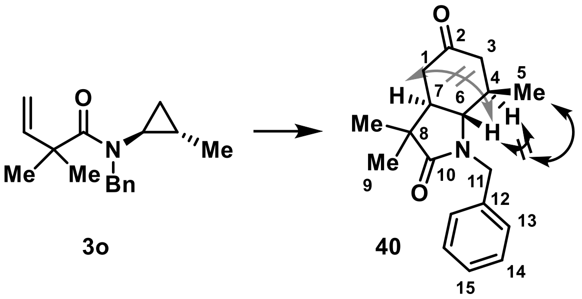

An oven dried reaction tube, fitted with a magnetic stirrer, was charged with aminocyclopropane substrate **3o** (25.7 mg, 0.10 mmol), $[Rh(cod)Cl]_2$ (2.5 mg, 0.005 mmol) and $P(C_6F_5)_3$ (10.6 mg, 0.02 mmol). The tube was fitted with a rubber septum and purged with argon. Anhydrous PhCN (1.0 mL) was added and the reaction was sparged with CO for ca. 10 seconds. The tube was heated to 150 °C, under a CO atmosphere (1 balloon) and stirred for 72 h. The mixture was cooled to r.t. and concentrated *in vacuo*. The residue was purified by flash column chromatography (60-70% EtOAc/hexane) to afford **4o** (19.4 mg, 68%) as a pale yellow oil, the product was isolated as a single diastereomer (>15:1 d.r.); ν_{max} / cm^{-1}: 2961 (m), 1687 (s), 1399 (m), 1315 (m), 1260 (m), 1137 (m), 733 (m), 701 (m); ^{1}H NMR ($CDCl_3$, 400 MHz): δ 7.34 – 7.20 (m, 3H, 2 × C**14**-H and C**15**-H), 7.15 – 7.09 (m, 2H, 2 × C**13**-H), 4.85 (d, *J* = 16.0 Hz, 1H, C**11**-$\underline{H}_aH_b$), 4.51 (d, *J* = 16.0 Hz, 1H, C**11**-$H_a\underline{H}_b$), 3.13 (dd, *J* = 9.6, 9.6 Hz, 1H, C**6**-H), 2.49 – 2.22 (m, 3H, C**1**-$\underline{H}_2$ and C**3**-$\underline{H}_aH_b$), 2.08 – 1.87 (m, 3H, C**3**-$H_a\underline{H}_b$, C**4**-H and C**7**-H), 1.21 (s, 3H, C**9**-$\underline{H}_3$), 1.06 – 1.02 (m, 6H, C**5**-$\underline{H}_3$ and C**9'**-$\underline{H}_3$); ^{13}C NMR ($CDCl_3$, 126 MHz): δ 207.7 (C**2**), 181.8 (C**10**), 137.4 (C**12**), 128.8 (C**14**), 127.4 (C**13**), 126.9 (C**15**), 63.6 (C**6**), 50.3 (C**7**), 49.1 (C**3**), 45.9 (C**11**), 42.2 (C**8**), 39.3 (C**1**), 37.0 (C**4**), 23.3 (C**9**), 21.2 (C**9'**), 17.6 (C**5**); HRMS: (ESI$^+$) calculated for $C_{18}H_{23}NNaO_2$: 308.1621, found $[M+Na]^+$: 308.1616. The relative stereochemistry of this compound was corroborated by nOe experiments (as indicated on the compound structure) and assigned in analogy with **4l**. No nOe was observed between C**6**-H and C**7**-H or between C**6**-H and C**4**-H. An nOe was observed between C**6**-H and C**5**-$\underline{H}_3$.

N-Benzyl-2,2-dimethyl-*N*-((1*S**,2*R**)-2-methylcyclopropyl)-4-phenylbut-3-ynamide (3p)

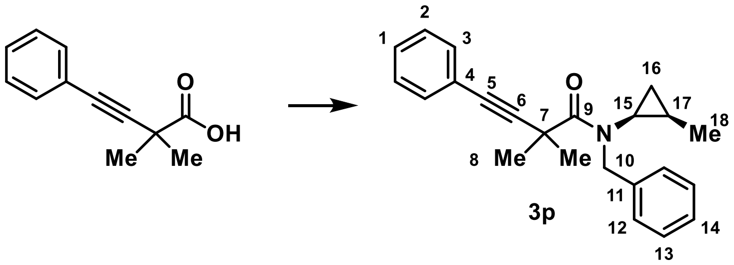

General procedure A: (1*S**,2*R**)-*N*-Benzyl-2-methylcyclopropan-1-amine (0.30 g, 1.86 mmol, prepared according to the literature procedure[10]) and 2,2-dimethyl-4-phenylbut-3-ynoic acid (0.35 g, 1.86 mmol) were employed and the residue was purified by flash column chromatography (5% EtOAc/hexane) to afford the title compound **3p** (0.31 g, 51%) as a colorless solid; m.p. 53-54 °C (DCM/hexane); ν_{max} / cm^{-1}: 2986 (m), 2258 (w), 1647 (s), 1395 (s), 1233 (m), 1162 (m), 755 (s), 691 (s); ^{1}H NMR (DMSO-*d*$_6$, 500 MHz, 110 °C): δ 7.31 – 7.11 (m, 10H, 2 × C**2**-H, 2 × C**3**-H, 2 × C**12**-H, 2 × C**13**-H, C**1**-H and C**14**-H), 5.45 (d, *J* = 16.2 Hz, 1H, C**10**-H_aH_b), 4.42 (d, *J* = 16.2 Hz, 1H, C**10**-H_aH_b), 2.74 (br. s, 1H, C**15**-H), 1.56 (s, 6H, 2 × C**8**-H_3), 1.07 – 0.93 (m, 4H, C**17**-H and C**18**-H_3), 0.73 – 0.64 (m, 1H, C**16**-H_aH_b), 0.39 (s, 1H, C**16**-H_aH_b); ^{13}C NMR (DMSO-*d*$_6$, 126 MHz, 110 °C): δ 173.8 (C**9**), 137.6 (C**11**), 130.5, 127.8, 127.8, 127.6, 126.5, 126.2 (C**1**, C**2**, C**3**, C**12**, C**13** and C**14**), 122.1 (C**4**), 92.6 (C**6**), 82.9 (C**5**), 51.2 (C**10**), 37.4 (C**7**), 34.5 (C**15**), 28.3 (C**8**), 28.0 (C**8'**), 13.4 (C**17**), 12.3 (C**16**), 11.7 (C**18**); HRMS: (ESI$^+$) calculated for $C_{23}H_{26}NO$: 332.2009, found $[M+H]^+$: 332.2014.

(6*S**,7a*R**)-1-Benzyl-3,3,6-trimethyl-4-phenyl-1,6,7,7a-tetrahydro-2*H*-indole-2,5(3*H*)-dione (4p)

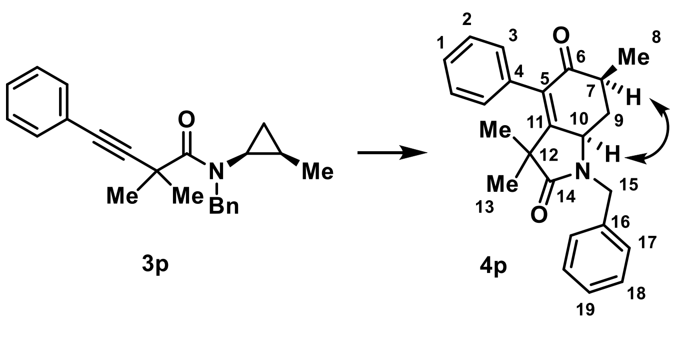

An oven dried reaction tube, fitted with a magnetic stirrer, was charged with aminocyclopropane substrate **3p** (33.1 mg, 0.10 mmol), $[Rh(cod)Cl]_2$ (2.5 mg, 0.005 mmol) and $P(4\text{-}OMeC_6H_4)_3$ (7.0 mg, 0.02 mmol). The tube was fitted with a rubber septum and purged with argon. Anhydrous PhCN (1.0 mL) was added and the reaction was sparged with CO for ca. 10 seconds. The tube was heated to 150 °C, under a CO atmosphere (1 balloon) and stirred for 72 h. The mixture was cooled to r.t. and concentrated *in vacuo*. The residue was purified by flash column chromatography (10-

25% EtOAc/hexane) to afford **4p** (18.0 mg, 50%) as a colorless oil, the product was isolated as a single diastereomer (>15:1 d.r.); ν_{max} / cm^{-1}: 2931 (m), 1677 (s), 1401 (m), 1208 (m), 910 (m), 729 (s), 699 (s); ^{1}H NMR ($CDCl_3$, 400 MHz): δ 7.35 – 7.19 (m, 8H, 2 × C**2**-H, 2 × C**17**-H, 2 × C**18**-H, C**1**-H and C**19**-H), 6.98 – 6.91 (m, 2H, 2 × C**3**-H), 5.07 (d, *J* = 15.0 Hz, 1H, C**15**-H_aH_b), 4.21 (dd, *J* = 11.5, 4.3 Hz, 1H, C**10**-H), 3.99 (d, *J* = 15.0 Hz, 1H, C**15**-H_aH_b), 2.41 (ddd, *J* = 11.6, 4.3, 4.3 Hz, 1H, C**9**-H_aH_b), 2.36 – 2.25 (m, 1H, C**7**-H), 1.58 (ddd, *J* = 13.3, 11.6, 11.5 Hz, 1H, C**9**-H_aH_b), 1.27 (s, 3H, C**13**-H_3), 1.17 (d, *J* = 6.8 Hz, 3H, C**8**-H_3), 0.73 (s, 3H, C**13''**-H_3); ^{13}C NMR ($CDCl_3$, 126 MHz): δ 199.2 (C**6**), 177.6 (C**14**), 161.6 (C**11**), 136.0 (C**16**), 135.7 (C**5**), 134.3 (C**4**), 130.0 (br., C**3**), 129.1, 128.1, 128.0, 128.0 (C**1**, C**2**, C**17**, C**18** and C**19**), 55.5 (C**10**), 46.0 (C**12**), 44.0 (C**15**), 40.3 (C**7**), 36.7 (C**9**), 26.1 (C**13**), 21.8 (C**13'**), 16.3 (C**8**); HRMS: (ESI$^+$) calculated for $C_{24}H_{26}NO_2$: 360.1958, found $[M+H]^+$: 360.1960.

The relative stereochemistry of this compound was corroborated by nOe experiments (as indicated on the compound structure). A strong nOe was observed between C**10**-H and C**7**-H.

N-Benzyl-*N*-((1*S**,2*R**)-2-butylcyclopropyl)-2,2-dimethyl-4-phenylbut-3-ynamide (3q)

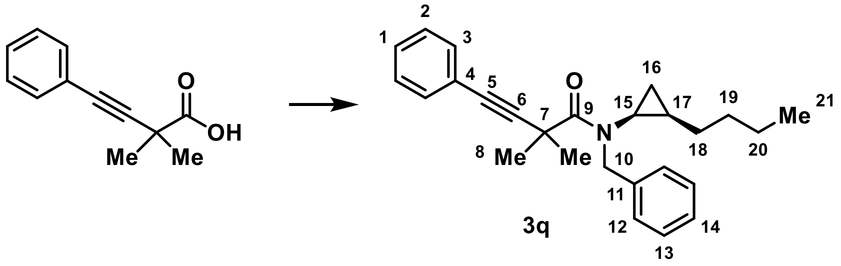

3q

General procedure A: (1*S**,2*R**)-*N*-Benzyl-2-butylcyclopropan-1-amine (0.40 g, 1.97 mmol, prepared according to the literature procedure[3]) and 2,2-dimethyl-4-phenylbut-3-ynoic acid (0.37 g, 1.97 mmol) were employed and the residue was purified by flash column chromatography (10% EtOAc/hexane) to afford the title compound **3q** (0.43 g, 58%) as a colorless oil; ν_{max} / cm^{-1}: 2928 (m), 1650 (s), 1454 (m), 1394 (s), 1232 (m), 1162 (m), 1030 (m), 755 (s), 690 (s); ^{1}H NMR (DMSO-d_6, 500 MHz, 110 °C): δ 7.33 – 7.19 (m, 8H, 2 × C**2**-H, 2 × C**12**-H, 2 × C**13**-H, C**1**-H, C**14**-H), 7.16 – 7.11 (m, 2H, 2 × C**3**-H), 5.48 (d, *J* = 16.3 Hz, 1H, C**10**-H_aH_b), 4.44 (d, *J* = 16.3 Hz, 1H, C**10**-H_aH_b), 2.78 (br. s, 1H, C**15**-H), 1.57 (s, 3H, C**8**-H_3), 1.56 (s, 3H, C**8'**-H_3), 1.52 – 1.42 (m, 1H, C**18**-H_aH_b), 1.39 – 1.24 (m, 4H, C**19**-H_2 and C**20**-H_2), 1.06 – 0.91 (m, 2H, C**18**-H_aH_b and C**17**-H), 0.85 (t, *J* = 7.2 Hz, 3H, C**21**-H_3), 0.71 – 0.63 (m, 1H, C**16**-H_aH_b), 0.46 – 0.39 (m, 1H, C**16**-H_aH_b); ^{13}C NMR (DMSO-d_6, 126 MHz, 110 °C): δ 173.6 (C**9**), 137.6 (C**11**), 130.5 (C**3**), 127.8, 127.7, 127.6, 126.4, 126.2 (C**1**, C**2**, C**12**, C**13** and C**14**), 122.1 (C**4**), 92.5 (C**6**), 82.8 (C**5**), 51.1 (C**10**), 37.3 (C**7**), 34.5 (C**15**), 30.7 (C**19**), 28.2, 28.0 (C**8** and C**8'**), 26.3 (C**18**), 21.4 (C**20**),

19.2 (C**17**), 13.1 (C**21**), 11.0 (C**16**); HRMS: (ESI$^+$) calculated for $C_{26}H_{32}NO$: 374.2478, found $[M+H]^+$: 374.2481.

(6*S,7a*R**)-1-Benzyl-6-butyl-3,3-dimethyl-4-phenyl-1,6,7,7a-tetrahydro-2*H*-indole-2,5(3*H*)-dione (4q) and (R)-1-benzyl-4-((E)-benzylidene)-5-((E)-hex-1-en-1-yl)-3,3-dimethyl-pyrrolidin-2-one (4q')**

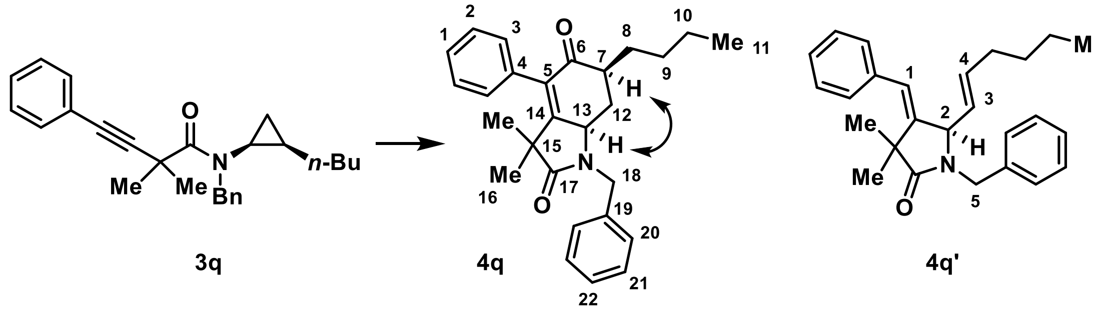

An oven dried reaction tube, fitted with a magnetic stirrer, was charged with aminocyclopropane substrate **3q** (37.4 mg, 0.10 mmol), $[Rh(cod)Cl]_2$ (2.5 mg, 0.005 mmol) and $P(4\text{-}OMeC_6H_4)_3$ (7.0 mg, 0.02 mmol). The tube was fitted with a rubber septum and purged with argon. Anhydrous PhCN (1.0 mL) was added and the reaction was sparged with CO for ca. 10 seconds. The tube was heated to 150 °C, under a CO atmosphere (1 balloon) and stirred for 72 h. The mixture was cooled to r.t. and concentrated *in vacuo*. The residue was purified by flash column chromatography (20% EtOAc/hexane) to afford **4q** (19.1 mg, 48%, isolated as a single diastereomer, >15:1 d.r.) and **4q'** (2.2 mg, 6%) as colorless oils.

Data for **4q**: ν_{max} / cm^{-1}: 2957 (m), 2930 (m), 2863 (m), 1698 (s), 1672 (s), 1418 (s), 1360 (m), 1310 (m), 1238 (m), 734 (s), 701 (s); ^{1}H NMR ($CDCl_3$, 400 MHz): δ 7.41 – 7.27 (m, 8H, 2 × C**2**-H, 2 × C**20**-H, 2 × C**21**-H, C**1**-H and C**22**-H), 7.03 – 6.98 (m, 2H, 2 × C**3**-H), 5.15 (d, *J* = 14.9 Hz, 1H, C**18**-$\underline{H_a}H_b$), 4.23 (dd, *J* = 11.6, 4.3 Hz, 1H, C**13**-H), 4.08 (d, *J* = 14.9 Hz, 1H, C**18**-$H_a\underline{H_b}$), 2.50 (ddd, *J* = 11.6, 4.3, 4.3 Hz, 1H, C**12**-$\underline{H_a}H_b$), 2.29 – 2.20 (m, 1H, C**7**-H), 1.96 – 1.87 (m, 1H, C**8**-$\underline{H_a}H_b$), 1.60 (ddd, *J* = 13.4, 11.6, 11.6 Hz, 1H, C**12**-$H_a\underline{H_b}$), 1.55 – 1.47 (m, 1H, C**8**-$H_a\underline{H_b}$), 1.36 – 1.23 (m, 7H, C**16**-H_3, C**9**-H_2 and C**10**-H_2), 0.91 (t, *J* = 7.0 Hz, 3H, C**11**-H_3), 0.81 (s, 3H, C**16'**-H_3); ^{13}C NMR ($CDCl_3$, 126 MHz): δ 199.0 (C**6**), 177.6 (C**17**), 161.2 (C**14**), 136.1 (C**19**), 134.5 (C**5**), 130.0 (br., C**3**), 129.1, 128.1, 128.1, 128.1, 128.0, 128.0 (C**1**, C**2**, C**4**, C**20**, C**21** and C**22**), 55.7 (C**13**), 45.9 (C**15**), 45.0 (C**7**), 44.1 (C**18**), 33.7 (C**12**), 30.1 (C**8**), 28.9 (C**9**), 26.1 (C**16**), 22.9 (C**10**), 21.9 (C**16'**), 14.1 (C**11**); HRMS: (ESI$^+$) calculated for $C_{27}H_{31}NNaO_2$: 424.2247, found $[M+Na]^+$: 424.2240. The relative stereochemistry of this compound was corroborated by nOe

experiments (as indicated on the compound structure). A strong nOe was observed between C**7**-H and C**13**-H. No significant nOe was observed between C**8**-H_2 and C**13**-H.

Data for **4q'** *(characteristic signals only)*: ^{1}H NMR ($CDCl_3$, 400 MHz): δ 6.45 (d, *J* = 2.2 Hz, 1H, C**1**-H), 5.44 (dt, *J* = 15.3, 6.9 Hz, 1H, C**4**-H), 5.11 (d, *J* = 15.0 Hz, 1H, C**5**-H_aH_b), 5.00 (ddt, *J* = 15.3, 8.8, 1.4 Hz, 1H, C**3**-H), 4.75 (dd, *J* = 8.8, 2.2 Hz, 1H, C**2**-H), 3.89 (d, *J* = 15.0 Hz, 1H, C**5**-H_aH_b).

N-Benzyl-*N*-((1*R**,5*S**,6*r*)-bicyclo[3.1.0]hexan-6-yl)-2,2-dimethyl-4-phenylbut-3-ynamide (3r)

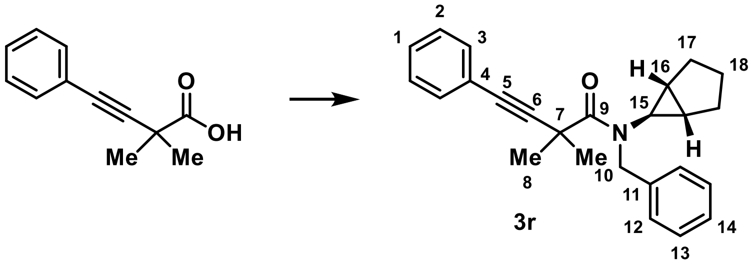

3r

General procedure A: (1*R**,5*S**,6*r*)-*N*-benzylbicyclo[3.1.0]hexan-6-amine (0.30 g, 1.60 mmol, prepared according to the literature procedure[3]) and 2,2-dimethyl-4-phenylbut-3-ynoic acid (0.30 g, 1.60 mmol) were employed and the residue was purified by flash column chromatography (10% EtOAc/hexane) to afford the title compound **3r** (0.40 g, 70%) as a colorless oil; ν_{max} / cm^{-1}: 2934 (m), 1643 (s), 1492 (m), 1393 (s), 1356 (m), 1165 (m), 755 (s), 691 (s); ^{1}H NMR (DMSO-d_6, 500 MHz, 110 °C): δ 7.32 – 7.17 (m, 10H, 2 × C**2**-H, 2 × C**3**-H, 2 × C**12**-H, 2 × C**13**-H, C**1**-H and C**14**-H), 4.76 (s, 2H, C**10**-H_2), 2.70 (br. s, 1H, C**15**-H), 1.67 – 1.54 (m, 12H, 2 × C**17**-H_2, 2 × C**8**-H_3, C**18**-H_2), 1.49 – 1.39 (m, 1H, C**16**-H), 0.95 – 0.82 (m, 1H, C**16**-H); ^{13}C NMR (DMSO-d_6, 126 MHz, 110 °C): δ 172.6 (C**9**), 137.9 (C**11**), 130.4, 127.8, 127.6, 127.5, 126.3, 126.0 (C**1**, C**2**, C**3**, C**12**, C**13** and C**14**), 122.2 (C**4**), 92.8 (C**6**), 82.9 (C**5**), 49.8 (C**10**), 37.7 (C**15**), 37.3 (C**7**), 28.0 (C**8**), 26.6, 26.2 (C**17** and C**18**), 20.7 (C**16**); HRMS: (ESI^+) calculated for $C_{25}H_{28}NO$: 358.2165, found $[M+H]^+$: 358.2164.

1-Benzyl-5a-hydroxy-3,3-dimethyl-4-phenyl-5a,6,7,8,8a,8b-hexahydrocyclopenta-[*g*]idole-2,5(1*H*,3*H*)-dione (4r')

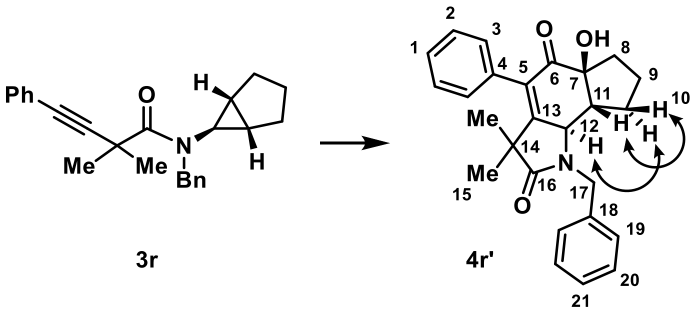

3r 4r'

An oven dried reaction tube, fitted with a magnetic stirrer, was charged with aminocyclopropane substrate **3r** (35.8 mg, 0.10 mmol), $[Rh(cod)Cl]_2$ (2.5 mg, 0.005 mmol) and $P(4\text{-}OMeC_6H_4)_3$ (7.0 mg, 0.02 mmol). The tube was fitted with a rubber septum and purged with argon. Anhydrous PhCN (1.0 mL) was added and the reaction was sparged with CO for ca. 10 seconds. The tube was heated to 150 °C, under a CO atmosphere (1 balloon) and stirred for 72 h. The mixture was cooled to r.t. and concentrated *in vacuo*. The residue was purified by flash column chromatography (10-25% EtOAc/hexane) to afford **4r'** (15.3 mg, 38%) as a pale yellow oil, the product was isolated as a single diastereomer (>15:1 d.r.); ν_{max} / cm^{-1}: 3406 (br. m), 2969 (m), 2932 (m), 1695 (s), 1680 (s), 1416 (m), 1074 (m), 910 (m), 731 (s), 700 (s); ^{1}H NMR ($CDCl_3$, 400 MHz): δ 7.43 – 7.27 (m, 6H, 2 × C**2**-$\underline{H}$, 2 × C**20**-$\underline{H}$, C**1**-$\underline{H}$ and C**21**-$\underline{H}$), 7.21 – 7.16 (m, 2H, 2 × C**19**-$\underline{H}$), 7.14 – 7.09 (m, 2H, 2 × C**3**-$\underline{H}$), 5.16 (d, *J* = 16.0 Hz, 1H, C**17**-$\underline{H}_aH_b$), 4.31 (d, *J* = 16.0 Hz, 1H, C**17**-$H_a\underline{H}_b$), 4.20 (d, *J* = 9.6 Hz, 1H, C**12**-$\underline{H}$), 3.42 (s, 1H, C**7**-O$\underline{H}$), 2.61 – 2.54 (m, 1H, C**11**-$\underline{H}$), 2.30 – 2.17 (m, 1H, C**10**-$\underline{H}_aH_b$), 2.01 – 1.82 (m, 3H, C**10**-$H_a\underline{H}_b$, C**8**-$\underline{H}_aH_b$, C**9**-$\underline{H}_aH_b$), 1.76 – 1.59 (m, 2H, C**8**-$H_a\underline{H}_b$, C**9**-$H_a\underline{H}_b$), 1.45 (s, 3H, C**15**-H_3), 0.80 (s, 3H, C**15'**-H_3); ^{13}C NMR ($CDCl_3$, 126 MHz): δ 198.6 (C**6**), 177.9 (C**16**), 161.6 (C**13**), 136.0, 133.8, 133.3 (C**4**, C**5** and C**18**), 130.3, 129.1, 128.5, 128.1, 127.7, 127.1 (C**1**, C**2**, C**3**, C**19**, C**20** and C**21**), 82.7 (C**7**), 58.1 (C**12**), 52.3 (C**11**), 46.1 (C**14**), 44.6 (C**17**), 37.2 (C**8**), 29.8 (C**10**), 26.6 (C**15**), 21.7, 21.7 (C**9** and C**15'**); HRMS: (ESI^+) calculated for $C_{26}H_{28}NO_3$: 402.2064, found $[M+H]^+$: 402.2052.

The relative stereochemistry of this compound was corroborated by nOe experiment (as indicated on the compound structure). An nOe was observed between C**11**-$\underline{H}$ and C**10**-$\underline{H}_aH_b$. An nOe was observed between C**12**-$\underline{H}$ and C**10**-$H_a\underline{H}_b$.

Selected Other Screened Systems

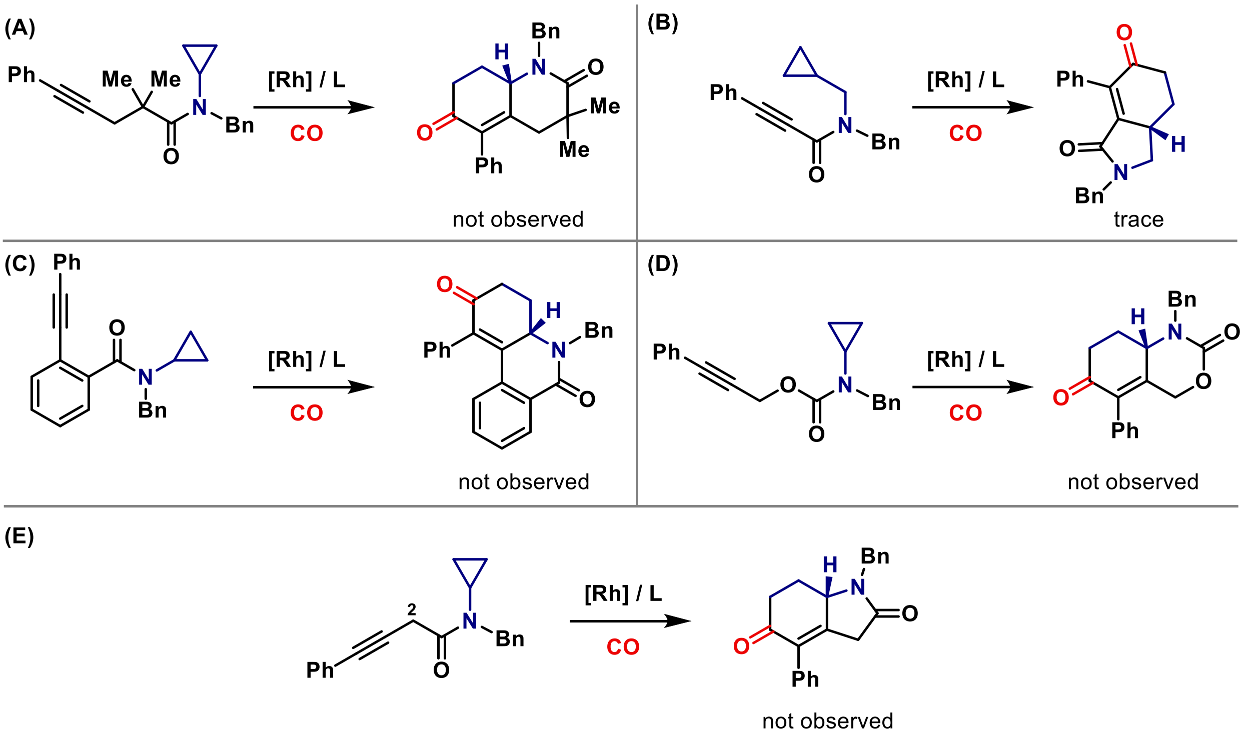

The failure of **(A)**, **(C)** and **(D)** is attributed to the more demanding requirements of forming a 6-membered ring. The failure of **(B)** is attributed to the much more demanding C-C bond activation of the aminomethylcyclopropane unit. The failure of **(E)** is attributed to the high acidity of the C2 center.

***N*-Benzyl-*N*-cyclopropylpropiolamide**

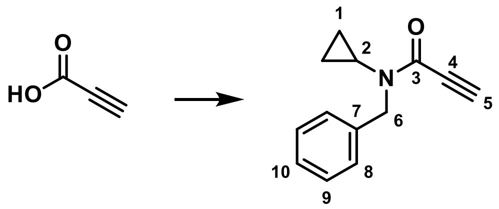

To a stirring solution of *N*-Benzylcyclopropanamine (1.47 g, 10.0 mmol), DCC (2.27 g, 11.0 mmol) and DMAP (1.2 mg, 0.01 mmol) in DCM (30 mL) at 0 °C was added propiolic acid (0.67 mL, 10.8 mmol) in DCM (10 mL). The reaction was stirred for 2 h before being filtered through a pad of celite, washing with DCM (2 × 10 mL). The organic solution was concentrated *in vacuo*. FCC (15% EtOAc/hexane) provided the title compound (2.00 g, quantitative, 3:1 mixture of rotamers A:B) as a pale yellow solid; m.p. 102–105 °C ($CHCl_3$); ν_{max} / cm^{-1}: 3206 (m), 2096 (m), 1615 (s), 1395 (m), 1303 (m); ^{1}H NMR ($CDCl_3$, 400 MHz): δ 7.38–7.25 (10H, m, C**8**-H, C**9**-H, C**10**-H, A+B), 4.77 (2H, s, C**6**-H_2, B), 4.60 (2H, s, C**6**-H_2, A), 3.20 (1H, s, C**5**-H, A), 3.08 (1H, s,

C**5**-H, B), 2.66 (1H, m, C**2**-H, A), 2.52 (1H, m, C**2**-H, B), 0.88–0.87 (4H, m, C**1**-H_2, A), 0.82 (2H, m, C**1**-H_2, B), 0.69 (2H, m, C**1**-H_2, B); ^{13}C NMR ($CDCl_3$, 101 MHz): δ 155.9 (C**3**, A), 155.2 (C**3**, B), 136.9 (C**7**, A), 136.8 (C**7**, B), 128.8, 128.6, 128.0, 127.8, 127.5, 127.2 (C**8**, C**9**, C**10**, A+B), 80.2 (C**5**, A), 78.7 (C**4**, A), 77.1 (C**5**, B), 76.6 (C**4**, B), 53.4 (C**6**, B), 49.8 (C**6**, A), 30.3 (C**2**, A), 28.1 (C**2**, B), 9.2 (C**1**, A), 7.1 (C**1**, B); HRMS: (ESI^+) Calculated for $C_{13}H_{14}NO$: 200.1070. Found $[M + H]^+$: 200.1064.

_N_-Benzyl-_N_-cyclopropyl-3-phenylpropiolamide

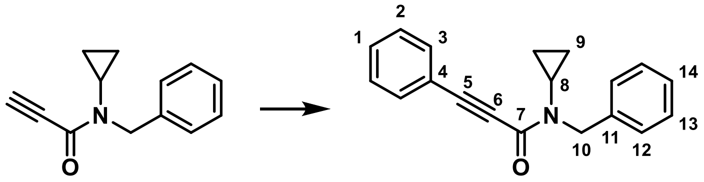

An oven dried reaction tube, fitted with a magnetic stirrer, was charged with the preceding amide (1.00 g, 5.00 mmol) and iodobenzene (0.67 mL, 6.00 mmol) were dissolved in dry TEA (10 mL) and the solution was sparged with argon. $Pd(PPh_3)_2Cl_2$ (70.2 mg, 2 mol%) and CuI (19.1 mg, 2 mol%) were added, the tube was sealed and the reaction mixture was heated at 80 °C for 16 h. The mixture was cooled to r.t., filtered through a pad of silica, rinsed with DCM (30 mL) and concentrated *in vacuo*. Flash column chromatography (20% EtOAc/hexane) afforded the title compound (0.59 g, 43%, 1:0.33 mixture of rotamers *A*:*B*) as a yellow oil; ν_{max} / cm^{-1}: 2216 (m), 1628 (s), 1490 (m), 1399 (s), 1303 (m), 758 (m), 690 (m); ^{1}H NMR ($CDCl_3$, 500 MHz): δ 7.58 – 7.54 (m, 2H, 2 × C**3**-H, *A*), 7.48 – 7.26 (m, 18H, 2 × C**3**-H, *B* and 2 × C**2**-H, 2 × C**12**-H, 2 × C**13**-H, C**1**-H, C**14**-H, *A*+*B*), 4.84 (s, 2H, C**10**-H_2, *B*), 4.66 (s, 2H, C**10**-H_2, *A*), 2.73 (tt, *J* = 6.8, 4.1 Hz, 1H, C**8**-H, *A*), 2.59 (tt, *J* = 7.1, 4.2 Hz, 1H, C**8**-H, *B*), 0.98 – 0.90 (m, 4H, 2 × C**9**-H_2, *A*), 0.87 – 0.82 (m, 2H, 2 × C**9**-H_aH_b, *B*), 0.75 – 0.71 (m, 2H, 2 × C**9**-H_aH_b, *B*); ^{13}C NMR ($CDCl_3$,126 MHz): δ 157.2 (C**7**, *A*), 156.6 (C**7**, *B*), 137.4 (C**11**, *A*+*B*), 132.6 (C**3**, *A*+*B*), 130.2, 130.1, 128.9, 128.7, 128.7, 128.6, 128.2, 127.8, 127.5, 127.4 (C**1**, C**2**, C**12**, C**13** and C**14**, *A*+*B*), 121.1 (C**4**, *A*), 120.6 (C**4**, *B*), 91.5 (C**5**, *A*), 90.0 (C**5**, *B*), 83.2 (C**6**, *A*), 82.6 (C**6**, *B*), 53.8 (C**10**, *B*), 49.9 (C**10**, *A*), 30.5 (C**8**, *A*), 28.4 (C**8**, *B*), 9.2 (C**9**, *A*), 7.4 (C**9**, *B*); HRMS: (ESI^+) calculated for $C_{19}H_{18}NO$: 276.1383, found $[M+H]^+$: 276.1377.

7-Benzyl-2-phenyl-7-azabicyclo[4.2.0]oct-1-ene-3,8-dione

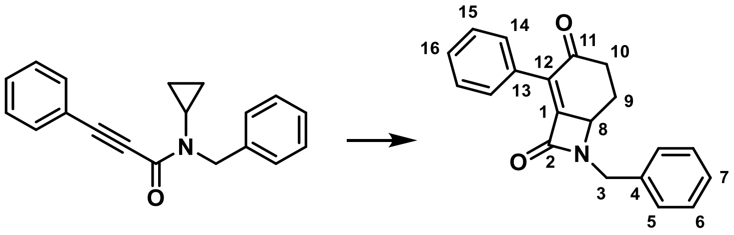

An oven dried reaction tube, fitted with a magnetic stirrer, was charged with aminocyclopropane substrate (27.5 mg, 0.10 mmol), $[Rh(cod)Cl]_2$ (2.5 mg, 0.005 mmol) and PPh_3 (5.2 mg, 0.02 mmol). The tube was fitted with a rubber septum and purged with argon. Anhydrous 1,2-DCB (1.0 mL) was added and the reaction was sparged with CO for ca. 10 seconds. The tube was heated to 130 °C, under a CO atmosphere (1 balloon) and stirred for 72 h. The mixture was cooled to r.t. and concentrated *in vacuo*. The residue was purified by flash column chromatography (10-25% EtOAc/hexane) to afford the [2.4.0] system (4.7 mg, 15%) as a yellow oil; ν_{max} / cm^{-1}: 2925 (w), 1746 (s), 1684 (s), 1379 (m), 1139 (m), 741 (m), 694 (m); ^{1}H NMR ($CDCl_3$, 400 MHz): δ 7.72 – 7.66 (m, 2H, 2 × C**14**-H), 7.45 – 7.30 (m, 8H, C**7**-H, C**16**-H, 2 × C**5**-H, 2 × C**6**-H and 2 × C**15**-H), 4.64 (d, *J* = 14.8 Hz, 1H, C**3**-$\underline{H}_aH_b$), 4.51 (d, *J* = 14.8 Hz, 1H, C**3**-$H_a\underline{H}_b$), 4.12 (dd, *J* = 11.4, 4.6 Hz, 1H, C**8**-H), 2.70 (ddd, *J* = 18.9, 4.9, 2.0 Hz, 1H, C**10**-$\underline{H}_aH_b$), 2.43 (ddd, *J* = 18.9, 13.9, 4.9 Hz, 1H, C**10**-$H_a\underline{H}_b$), 2.10 (dtd, *J* = 11.8, 4.6, 2.0 Hz, 1H, C**9**-$\underline{H}_aH_b$), 1.74 (dtd, *J* = 13.9, 11.4, 4.9 Hz, 1H, C**9**-$H_a\underline{H}_b$); ^{13}C NMR ($CDCl_3$, 101 MHz): δ 196.6 (C**11**), 160.6 (C**2**), 154.6 (C**1**), 135.2 (C**4**), 132.7 (C**13**), 130.4 (C**12**), 130.3 (C**14**), 129.8, 129.2, 128.8, 128.5, 128.4 (C**5**, C**6**, C**7**, C**15** and C**16**), 56.3 (C**8**), 46.7 (C**3**), 36.8 (C**10**), 28.2 (C**9**); HRMS: (ESI^+) calculated for $C_{20}H_{18}NO_2$: 304.1332, found $[M+H]^+$: 304.1322.

Optimization Studies

Survey of chiral ligands for asymmetric (3+1+2) cycloaddition:

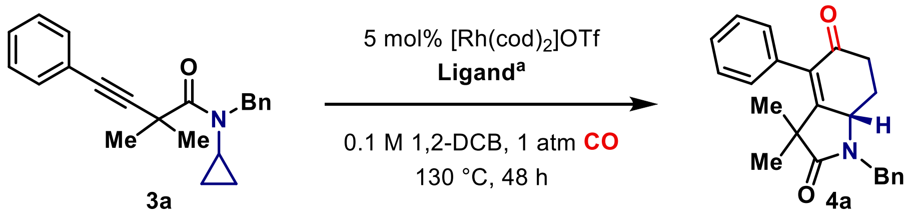

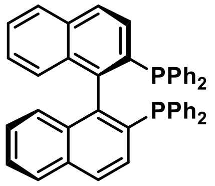

(*R*)-BINAP
15% Yield
49:51 e.r.

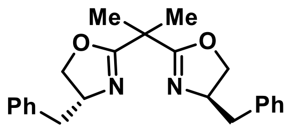

(*R*,*R*)-4-benzyl bisoxazoline
11% Yield
47:53 e.r.

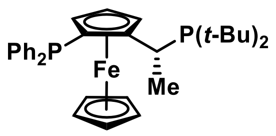

Josiphos SL-J002-1
7% Yield
46.5:53.5 e.r.

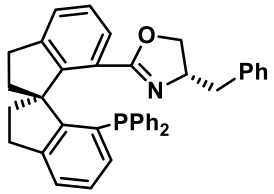

(*Ra*,*S*)-Ph-Bn-SIPHOX
5% Yield
49:51 e.r.

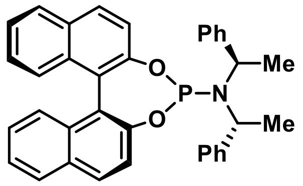

(*S*,*R*,*R*)-BINOL phosphoramidite
38% Yield
57:43 e.r.

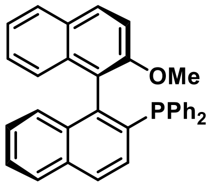

(*R*)-MOP
32% Yield
50:50 e.r.

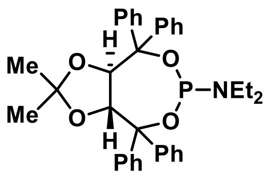

(3a*R*,8a*R*)-phosphoramidite
32% Yield
50:50 e.r.

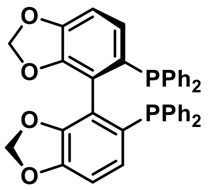

(*R*)-SEGPHOS
21% Yield
48:52 e.r.

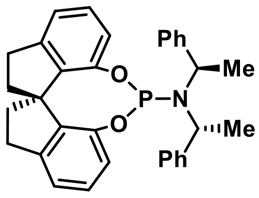

(*R*)-SIPHOS-PE
38% Yield
56:44 e.r.

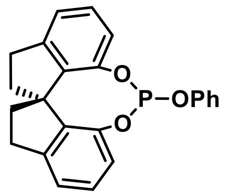

(*S*)-ShiP
13% Yield
59:41 e.r.

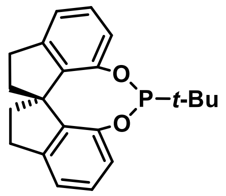

(*R*)-FuP-tBu
44% Yield
51:49 e.r.

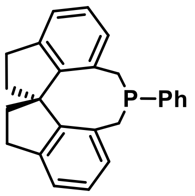

(*S*)-SITCP
73% Yield
93:7 e.r.

Yields were determined by ^{1}H NMR spectroscopy using 1,4-dinitrobenzene as a standard.

[a] Bidentate ligands: 5 mol%, monodentate ligands: 10 mol%.

Selected additional optimization experiments for the enantioselective transformation:

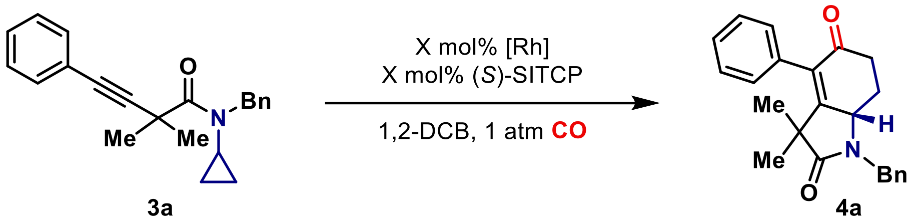

Entry	Rh[I] (mol %)	(*S*)-SITCP (mol %)	1,2-DCB (M)	Time (h)	T (°C)	Yield of 4a[a] (%)	3a [a] (%)	e.r.
1	[Rh(cod)$_2$]OTf (5)	10	0.1	48	130	73	0 (+21% **4a'**)	93:7
2	**[Rh(cod)Cl]$_2$ (5)**	20	0.1	48	130	0	>99	-
3	**[Rh(cod)$_2$]BF$_4$ (5)**	10	0.1	48	130	47	0	70:30
4	[Rh(cod)$_2$]OTf (5)	10	**0.05**	48	130	92	0 (+7% **4a'**)	92:8
5	[Rh(cod)$_2$]OTf (5)	10	0.1	**72**	**115**	66	32	95:5
6	**[Rh(cod)$_2$]OTf (7.5)**	15	0.1	72	115	83	trace	90:10
7	[Rh(cod)$_2$]OTf (7.5)	15	0.1	**96**	110	78 (76)	7	95:5
8	[Rh(cod)$_2$]OTf (7.5)	**11.25**	0.1	96	110	66	-	94.5:5.5
9	[Rh(cod)$_2$]OTf (7.5)	**7.5**	0.1	96	110	68	-	91:9
10	[Rh(cod)$_2$]OTf (7.5)	**18.75**	0.1	96	110	63	-	95:5

[a]Yields were determined by ^{1}H NMR spectroscopy. Isolated yields are given in parentheses.

Selected optimization experiments for cycloadditions of polysubstituted cyclopropanes:

trans-disubstituted system:

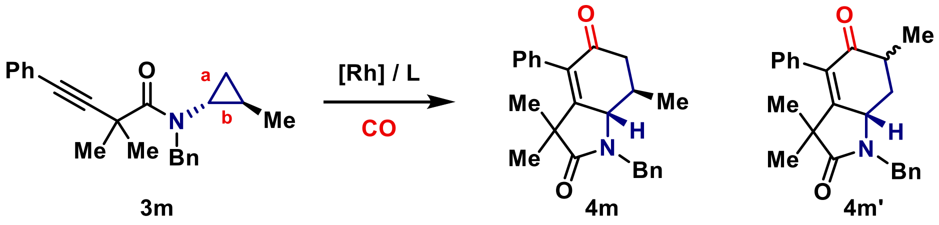

Entry	Rh[I] (mol %)	Ligand (mol %)	Solvent (M)	Time (h)	T (°C)	4m[a] (%)	4m'[a] (%)
1	$[Rh(cod)_2]OTf$ (5)	PPh_3, 10	1,2-DCB, 0.1	48	130	5	10 (1:1 d.r.)
2	**$[Rh(cod)Cl]_2$ (5)**	PPh_3, 20	1,2-DCB, 0.1	48	130	11	trace
3	$[Rh(cod)Cl]_2$ (5)	PPh_3, 20	**PhCN, 0.1**	48	130	6	24 (1:1 d.r.)
4	**$[Rh(cod)_2]OTf$ (5)**	PPh_3, 10	PhCN, 0.1	48	130	15	5 (1:1 d.r.)
5	$[Rh(cod)Cl]_2$ (5)	PPh_3, 20	PhCN, 0.1	**72**	**150**	63	22 (1.2:1 d.r.)
6	$[Rh(cod)Cl]_2$ (5)	**$P(4\text{-}OMeC_6H_4)_3$, 20**	PhCN, 0.1	72	150	62	4 (2:1 d.r.)
7	$[Rh(cod)Cl]_2$ (5)	**$P(3,4,5\text{-}F_3C_6H_4)_3$, 20**	PhCN, 0.1	72	150	57	trace
8	$[Rh(cod)Cl]_2$ (5)	**$P(C_6F_5)_3$, 20**	PhCN, 0.1	72	150	86 (84)	-

[a]Yields were determined by ^{1}H NMR spectroscopy. Isolated yields are given in parentheses.

cis-disubstituted system:

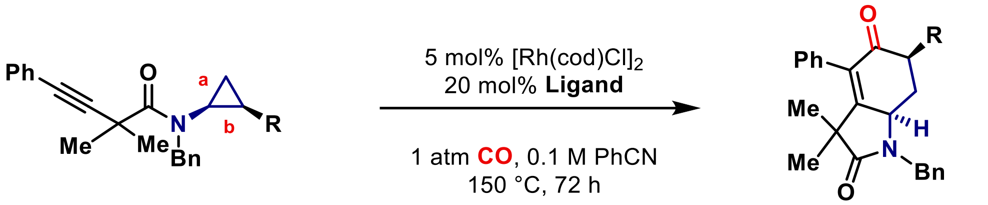

3p R = Me
3q R = n-Bu

4p R = Me, >15:1 d.r.
4q R = n-Bu, >15:1 d.r.

Entry	R	Ligand	4p/q[a] (%)
1	Me	$P(C_6F_5)_3$	9
2	Me	PPh_3	22
3	Me	P(3,5-$Me_2C_6H_3)_3$	28
4	Me	P(4-$OMeC_6H_4)_3$	**51 (50)**
5	Me	P((2,4,6-$OMe)_3C_6H_2)_3$	<10
6	*n*-Bu	P(4-$OMeC_6H_4)_3$	**45 (48)**
7	*n*-Bu	P(3,5-Me_2-4-$OMeC_6H_2)_3$	35

[a]Yields were determined by ^{1}H NMR spectroscopy. Isolated yields are given in parentheses.

Reversibility Studies:

Previous studies within the Bower group provide some insight into reversible C-C bond activation using cationic[10] and neutral[12] Rh-precatalysts. Obtained observations are summarized below:

Neutral monomeric complex **II** was synthesized by addition of two equivalents of $P(3,5\text{-}(CF_3)_2C_6H_3)_3$ to the dimeric complex **I**. Subsequent exposure of **II** to AgOTf resulted in quantitative conversion to model cationic rhodacyclopentanone complex **III** (Scheme 1A).

When *neutral* rhodacycle **II** was heated in the presence of aminocyclopropane **IV**, containing an ethyl carbamate protecting group, both cyclopropanes **IV** and **IV'** were observed in 73% and 44% yield respectively (Scheme 1B). Rhodacycle **II'** and was starting rhodacycle **II** were not observed. The formation of enamines **V** and **V'** also occurred in 14% and 10% yield respectively; these likely form *via* oxidative insertion of the Rh-catalyst into the aminocyclopropane followed by β-hydride elimination and C-H reductive elimination. These observations suggest that rhodacyclopentanone **II** can extrude CO and reductively eliminate cyclopropane **IV'** to yield an active catalyst which can subsequently insert into either cyclopropane **IV** or **IV'**. Examination of ^{31}P NMR spectra revealed 81% conversion to a species which was tentatively assigned as **VI**. These investigations suggest that the neutral Rh(I)-system provides some degree of reversibility.[12]

Exposure of *cationic* rhodacyclic complex **III** to stoichiometric quantities of aminocyclopropane **IV** under an atmosphere of CO resulted in formation of a new rhodacyclopentanone complex **III′** in 13% yield (+70% **III** remaining) and cyclopropane **IV'** in 9% yield (Scheme 1C). In the inverse experiment, rhodacycle **III** and cyclopropane **IV** were both formed in 5% yield. These exchange experiments show that reversible rhodacyclopentanone formation takes place under an atmosphere of CO using model cationic complexes **III**/**III′**.[10]

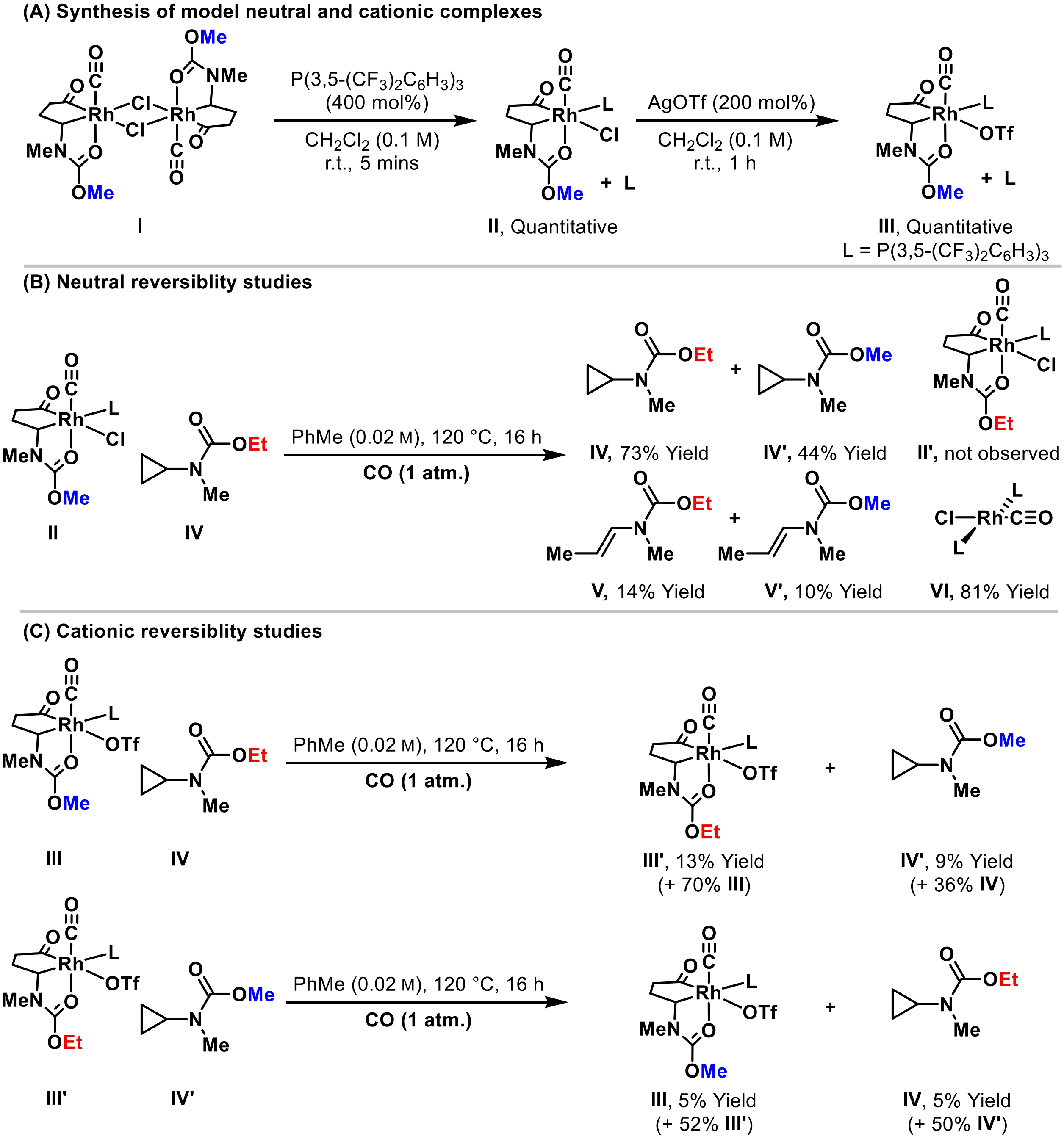

Scheme 1: Rhodacyclopentanone reversibility studies.

NMR Spectra of Novel Compounds

N-Benzyl-*N*-cyclopropyl-2,2-dimethyl-4-phenylbut-3-ynamide (3a)

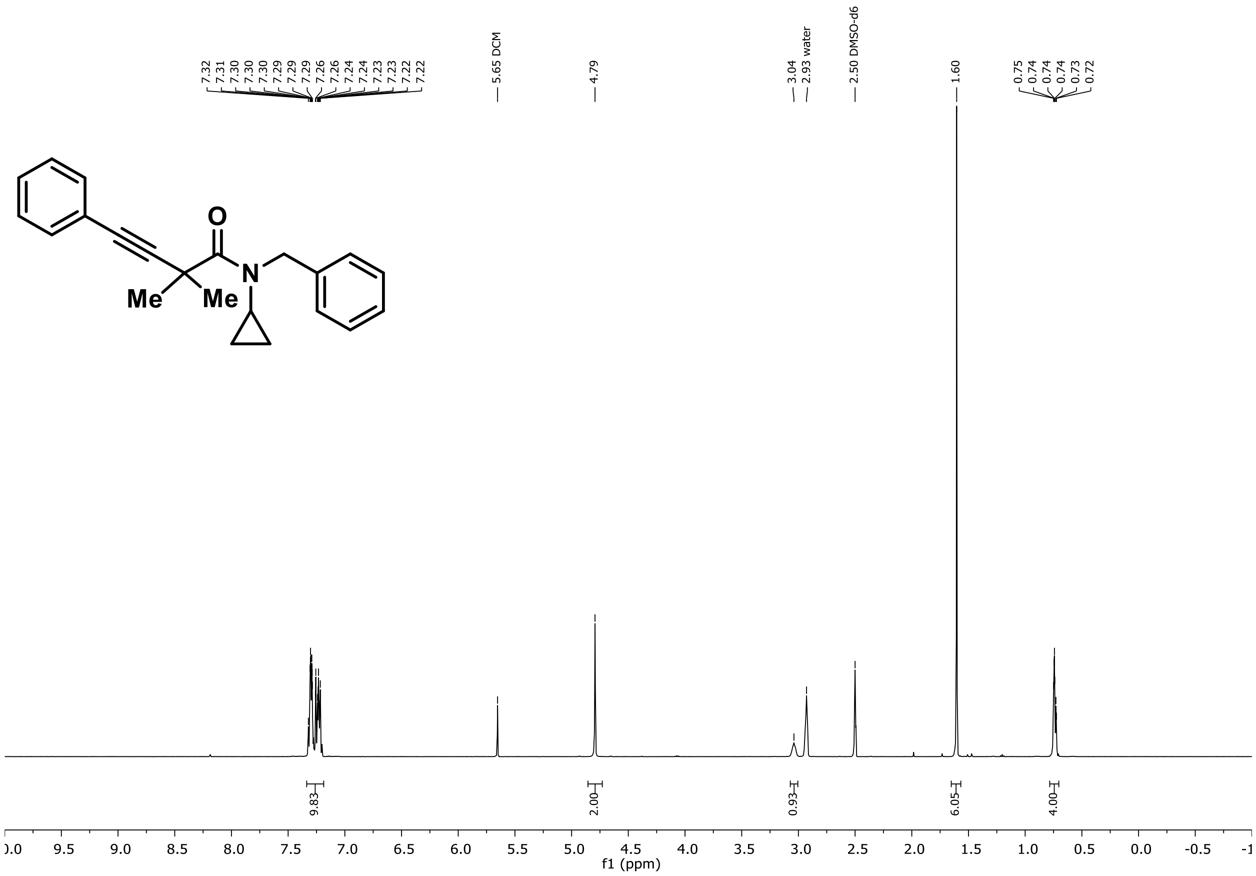

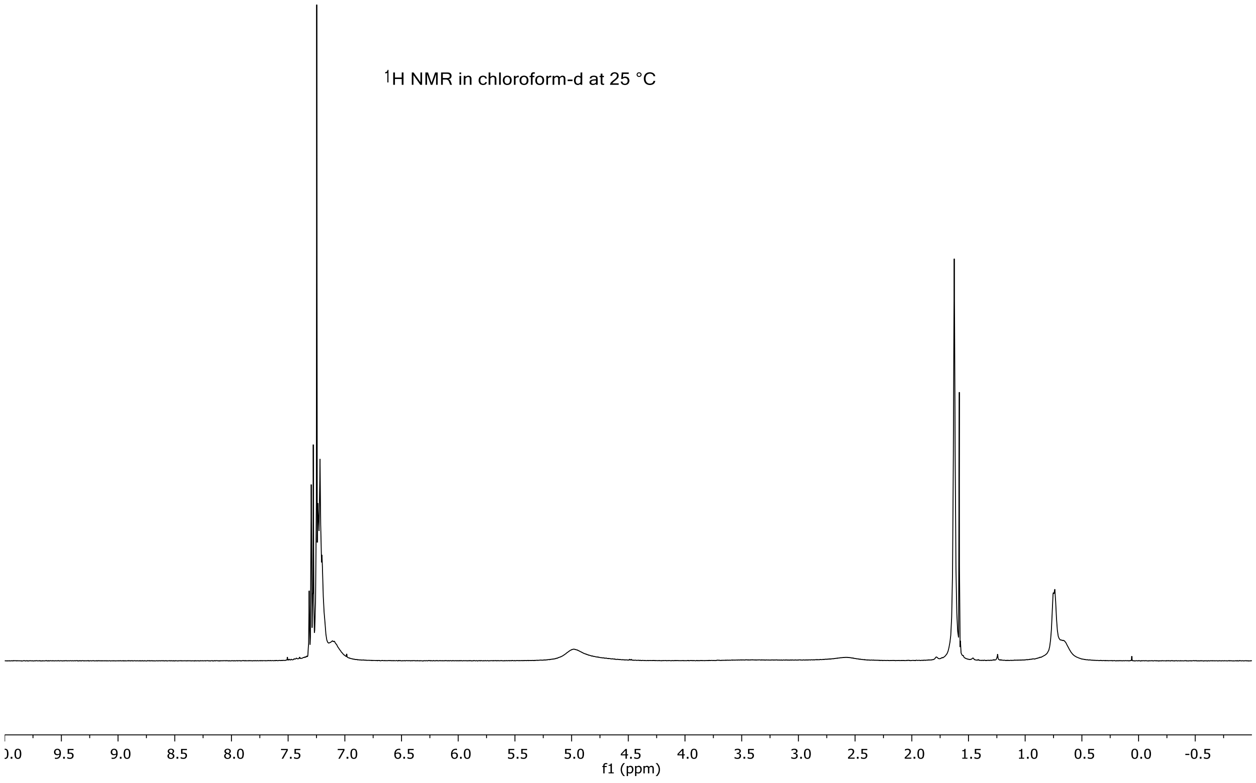

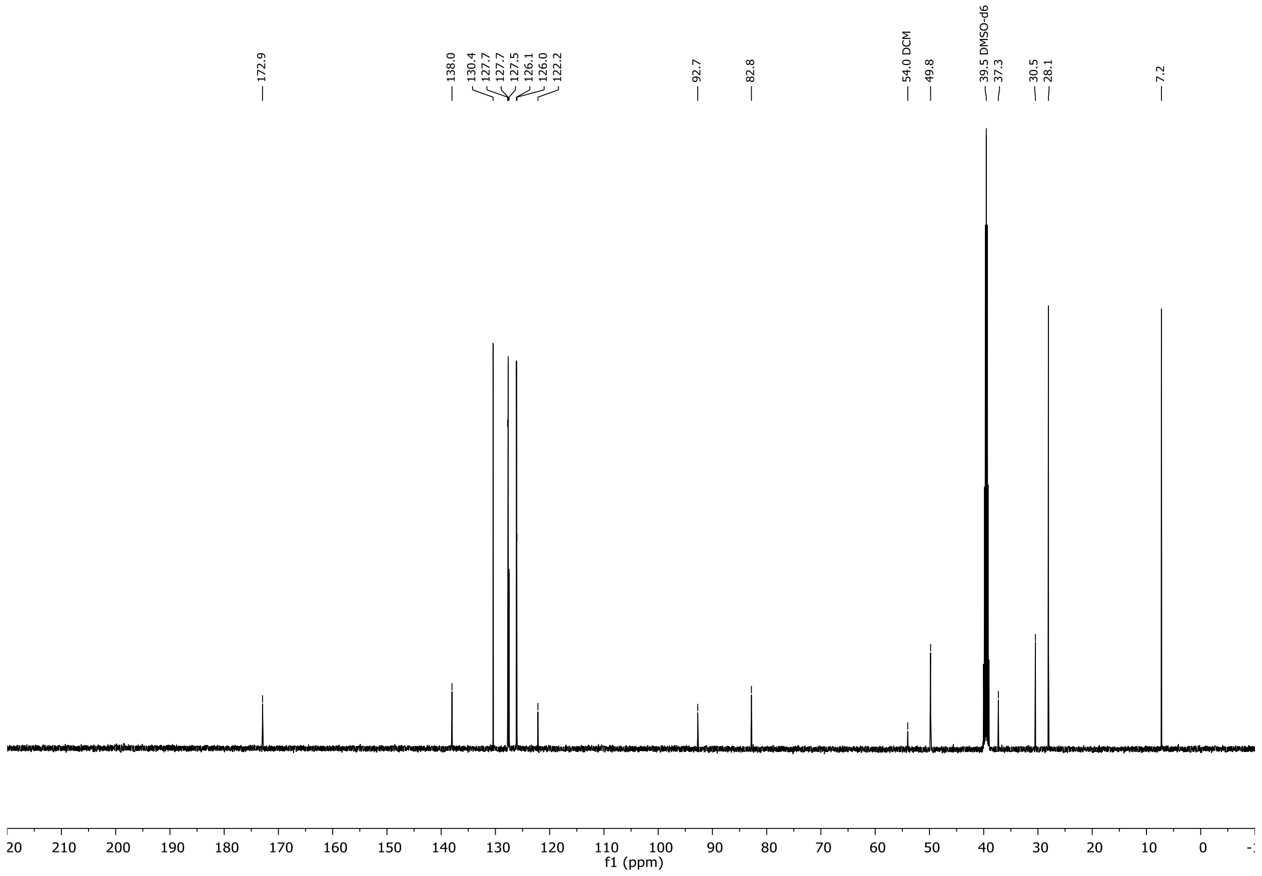
172.9
138.0
130.4
127.7
127.7
127.5
126.1
126.0
122.2
92.7
82.8
54.0 DCM
49.8
39.5 DMSO-d6
37.3
30.5
28.1
7.2
f1 (ppm)

1-Benzyl-3,3-dimethyl-4-phenyl-1,6,7,7a-tetrahydro-2*H*-indole-2,5(3*H*)-dione (4a)

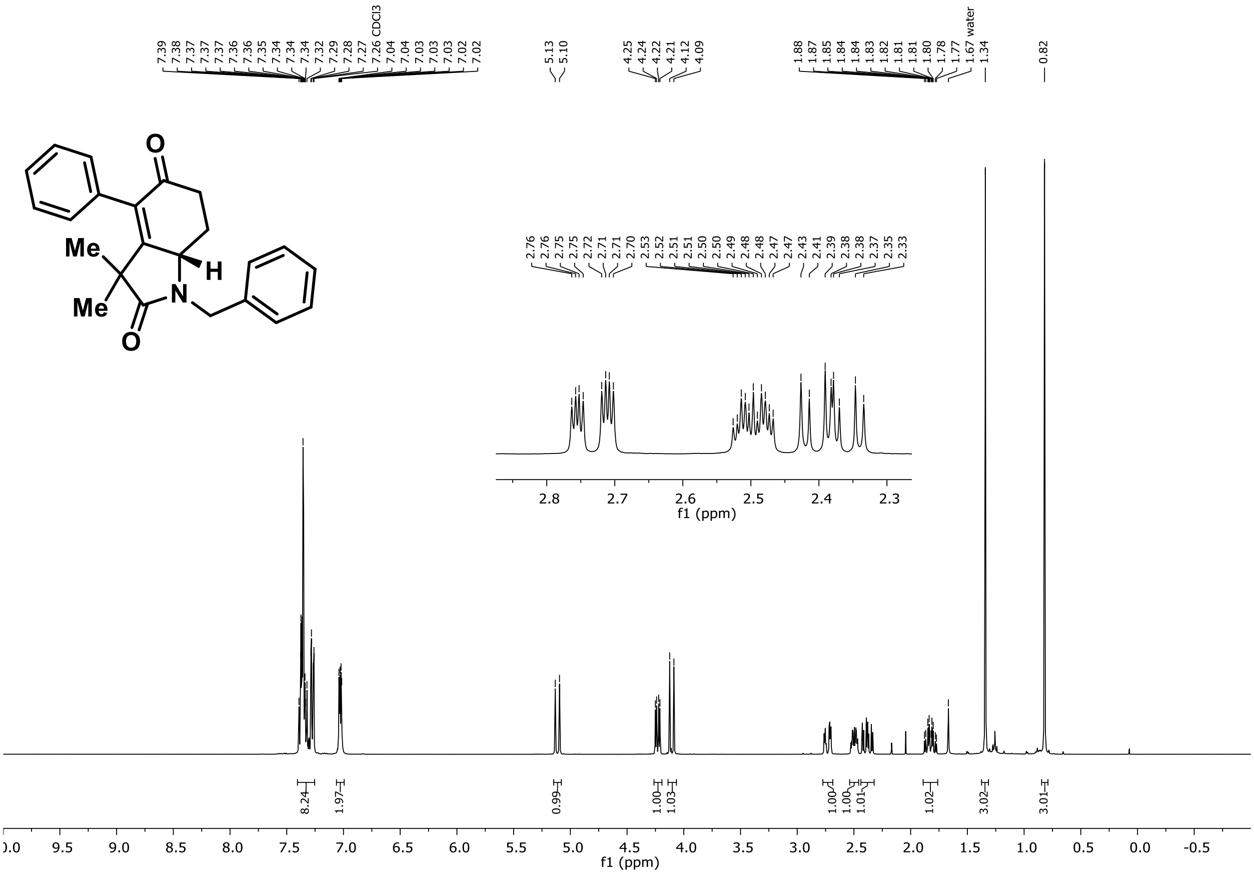

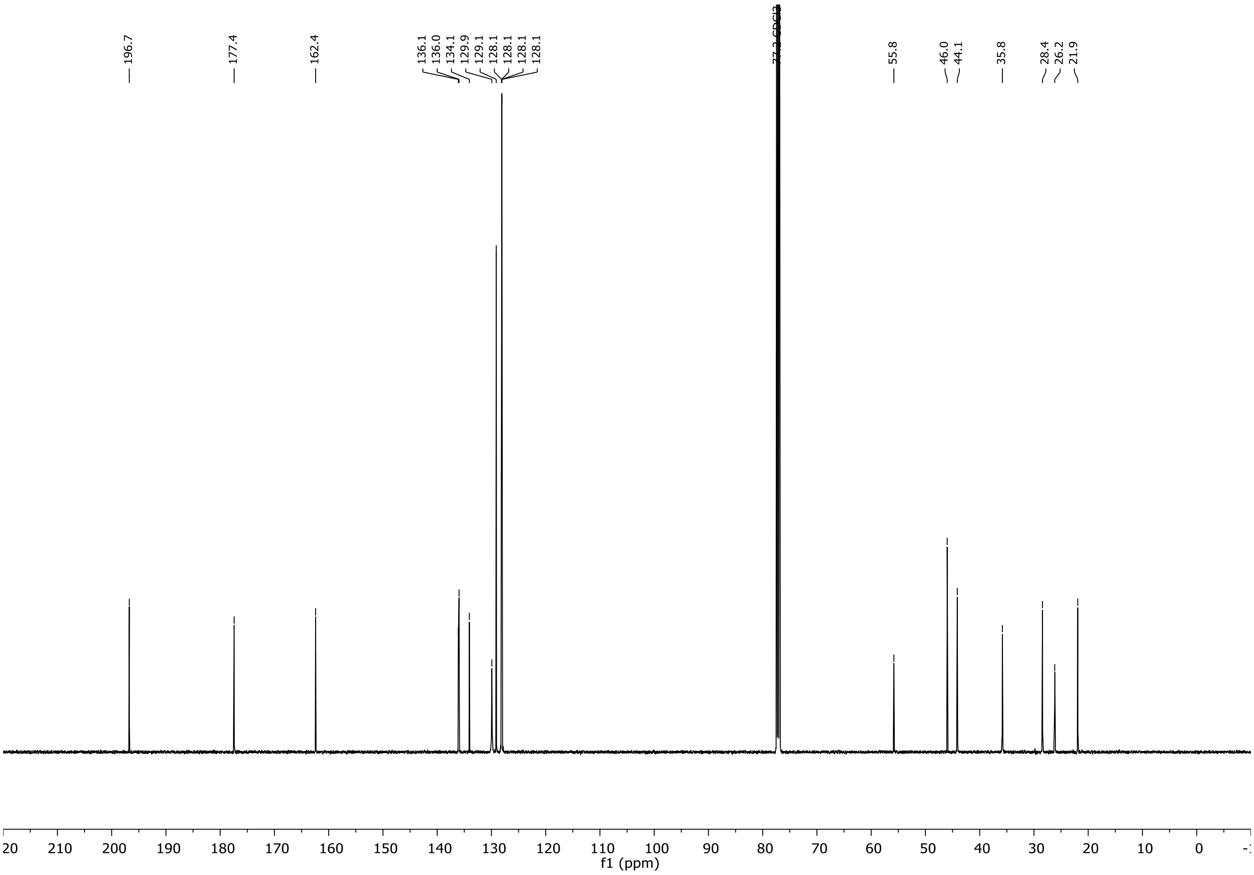

N-Butyl-*N*-cyclopropyl-2,2-dimethyl-4-phenylbut-3-ynamide (3b)

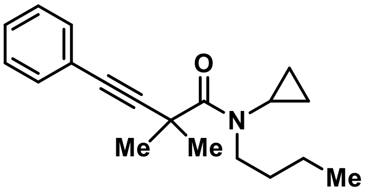

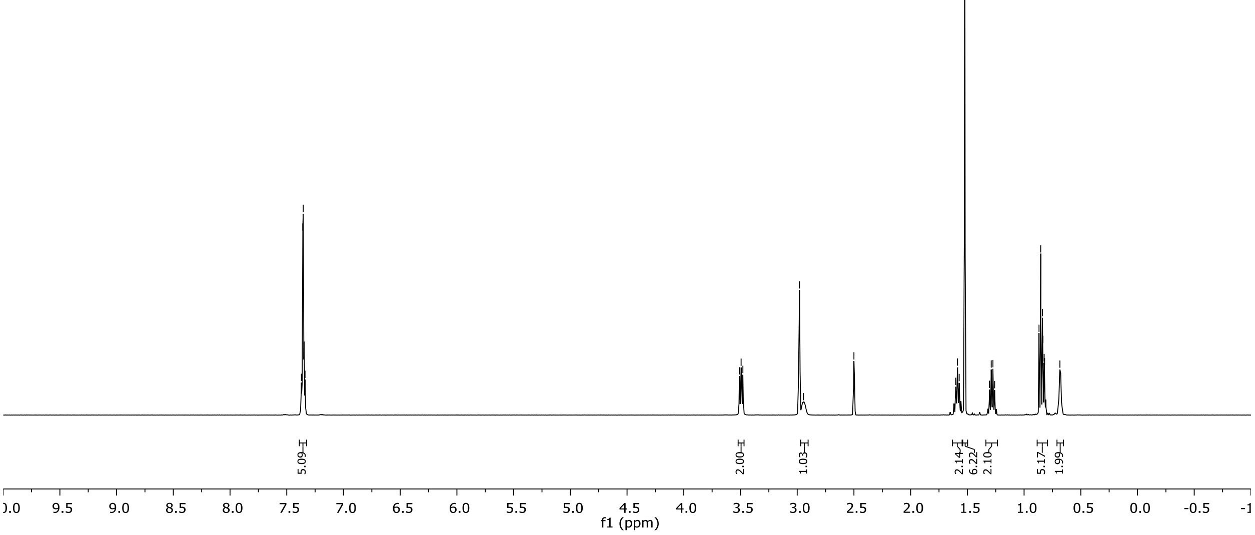

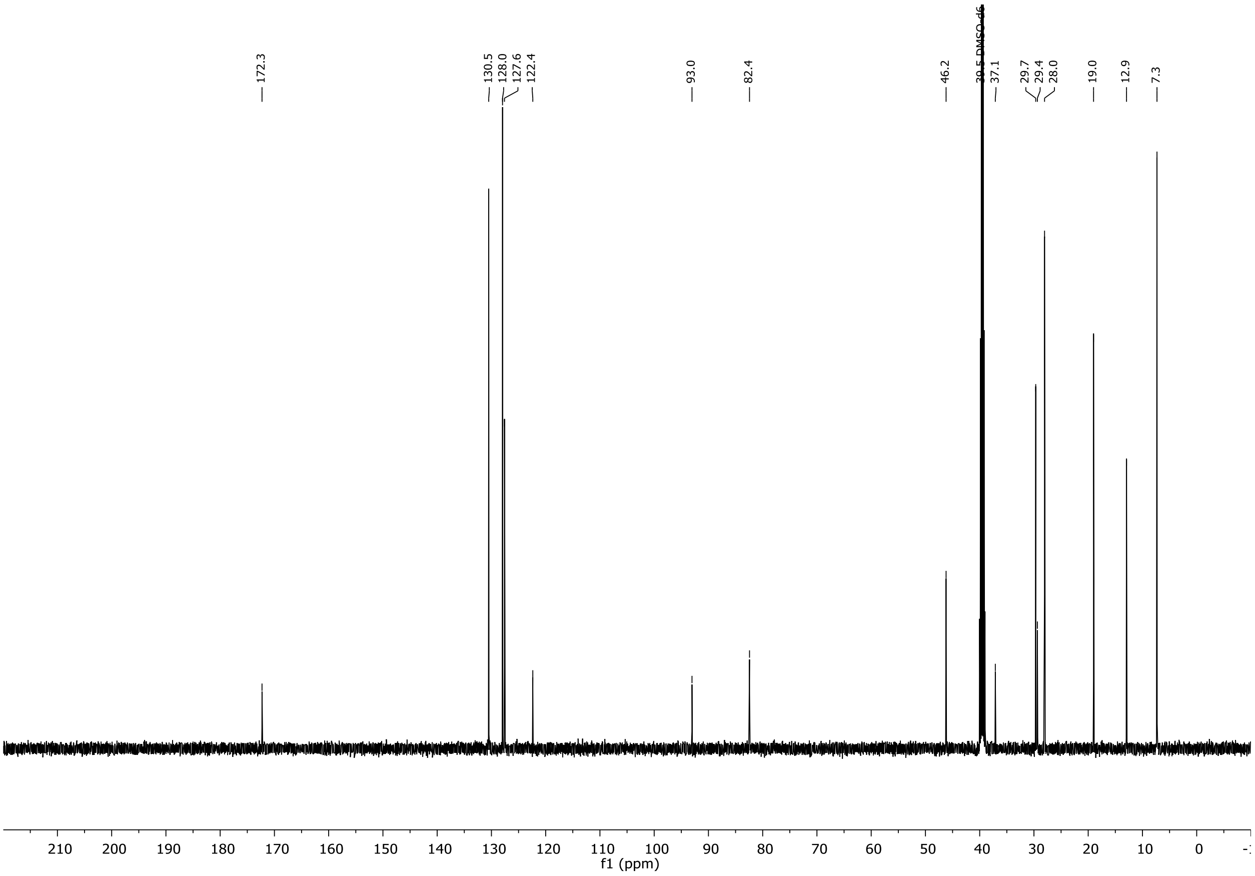

1-Butyl-3,3-dimethyl-4-phenyl-1,6,7,7a-tetrahydro-2*H*-indole-2,5(3*H*)-dione (4b)

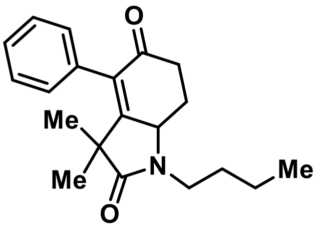

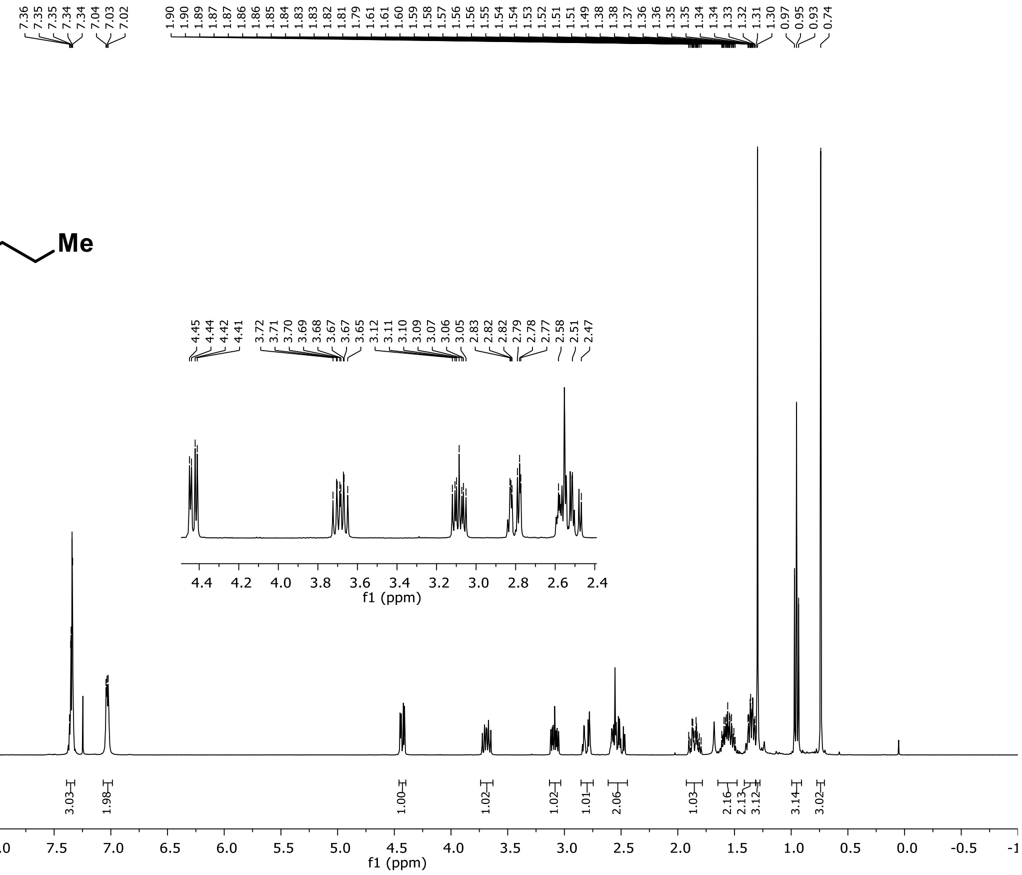

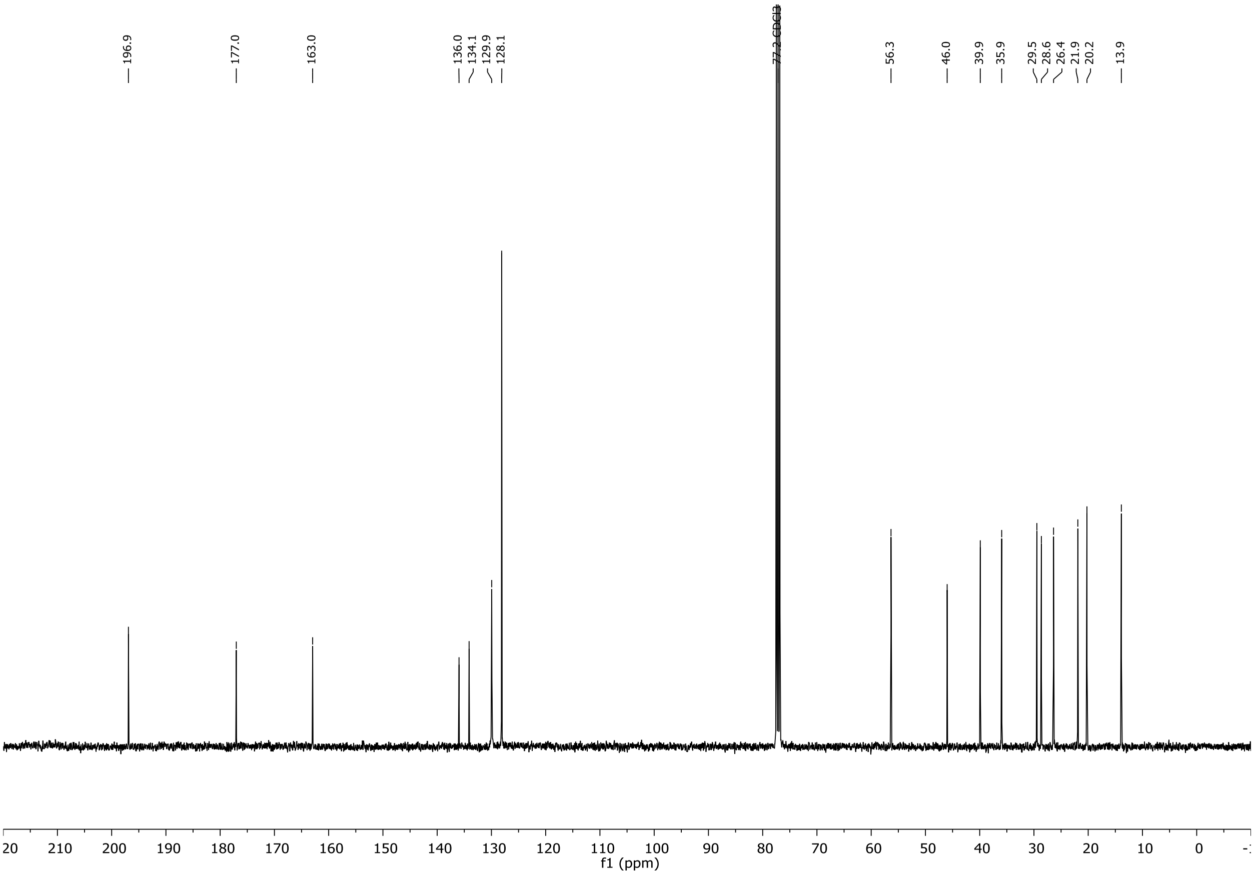

1-(Phenylethynyl)cyclopentane-1-carboxylic acid

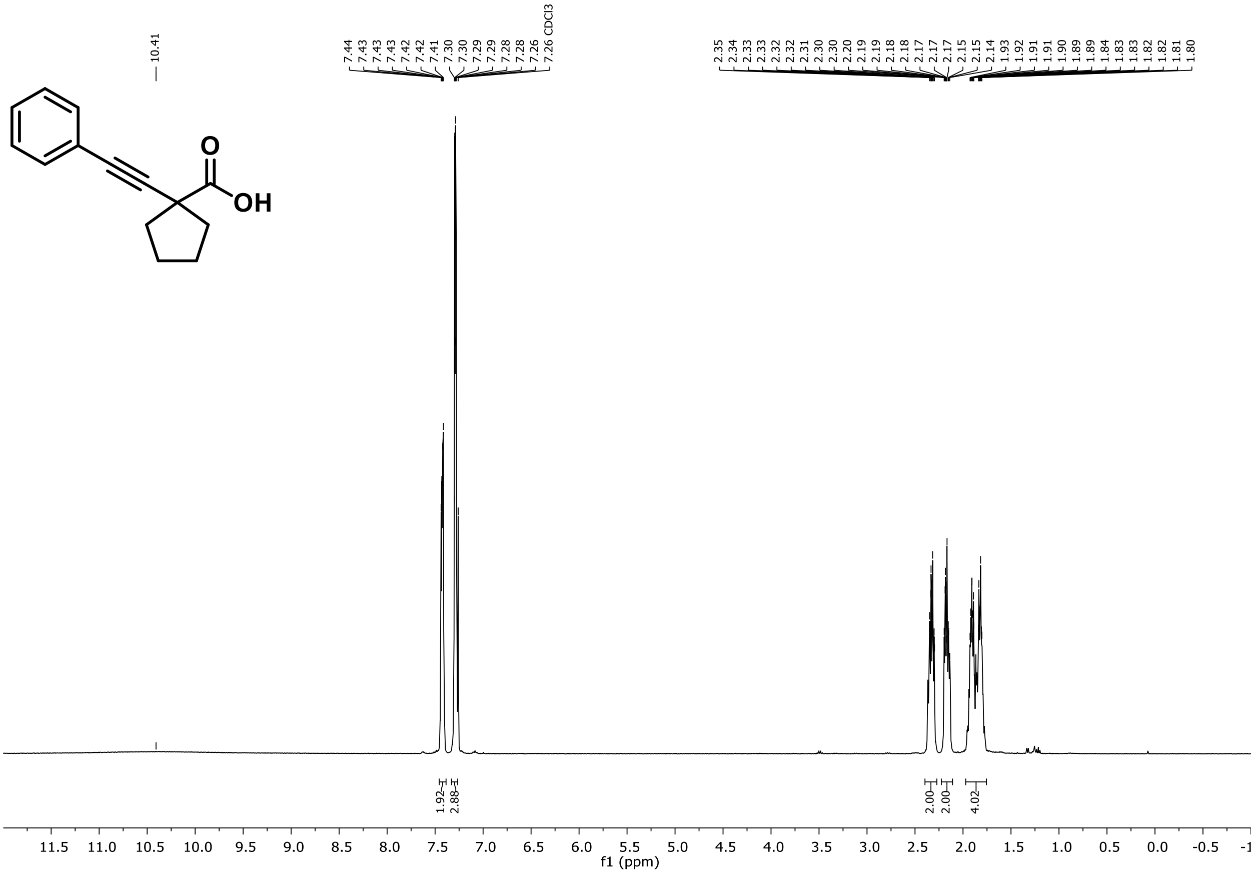

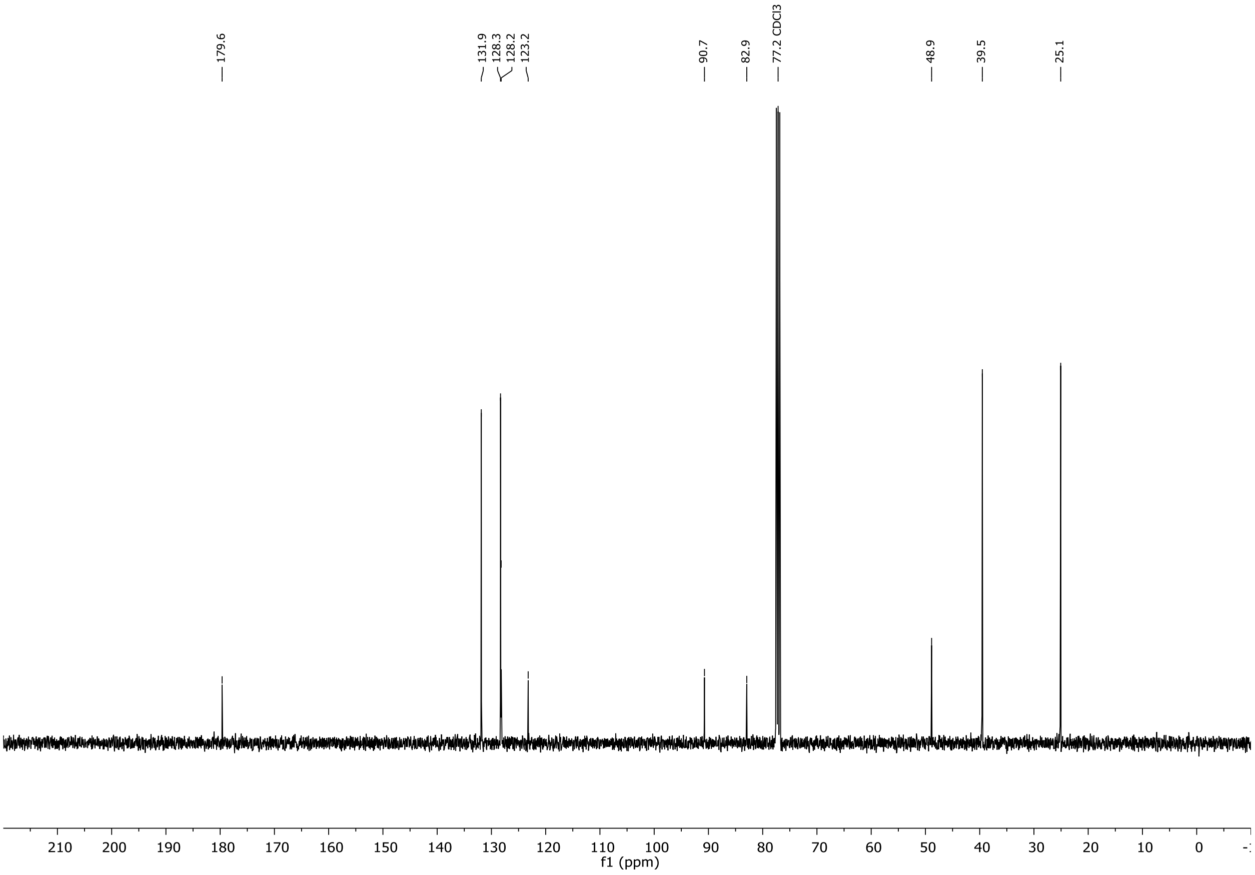

N-Benzyl-*N*-cyclopropyl-1-(phenylethynyl)cyclopentane-1-carboxamide (3c)

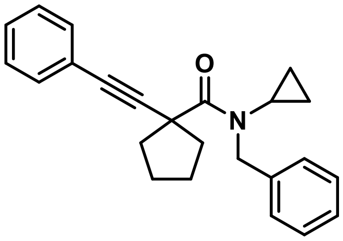

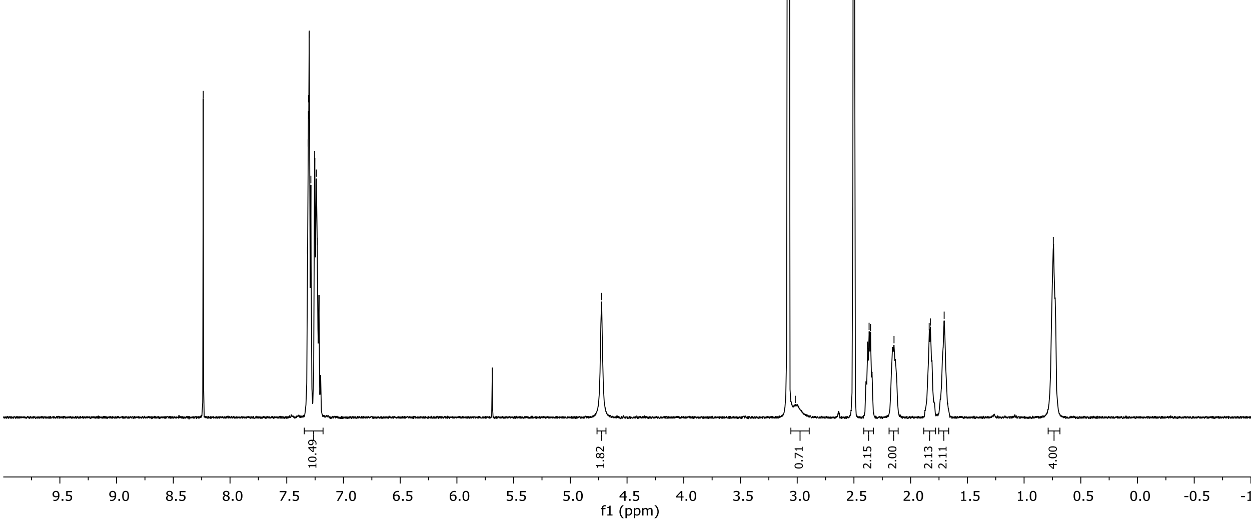

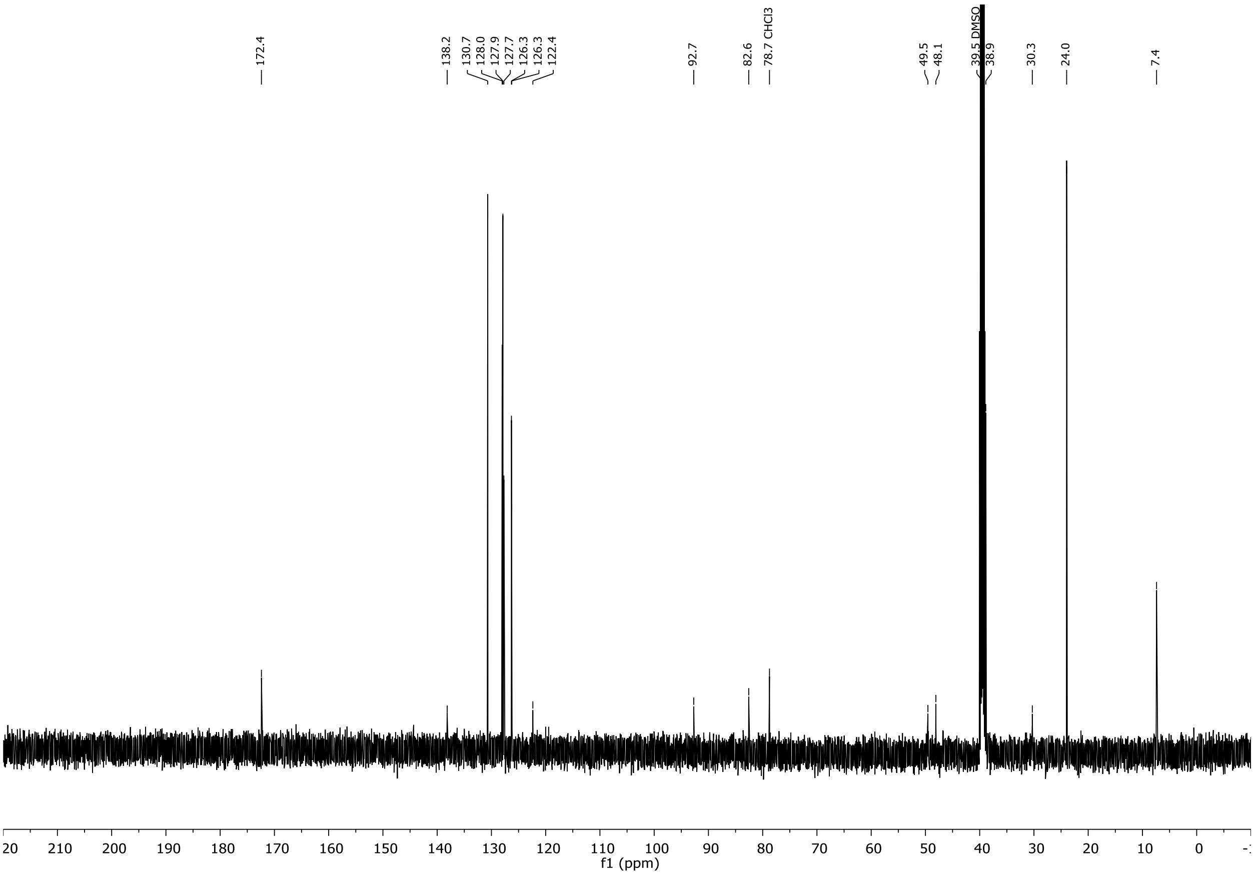

1'-Benzyl-4'-phenyl-1',6',7',7a'-tetrahydrospiro[cyclopentane-1,3'-indole]-2',5'-dione (4c)

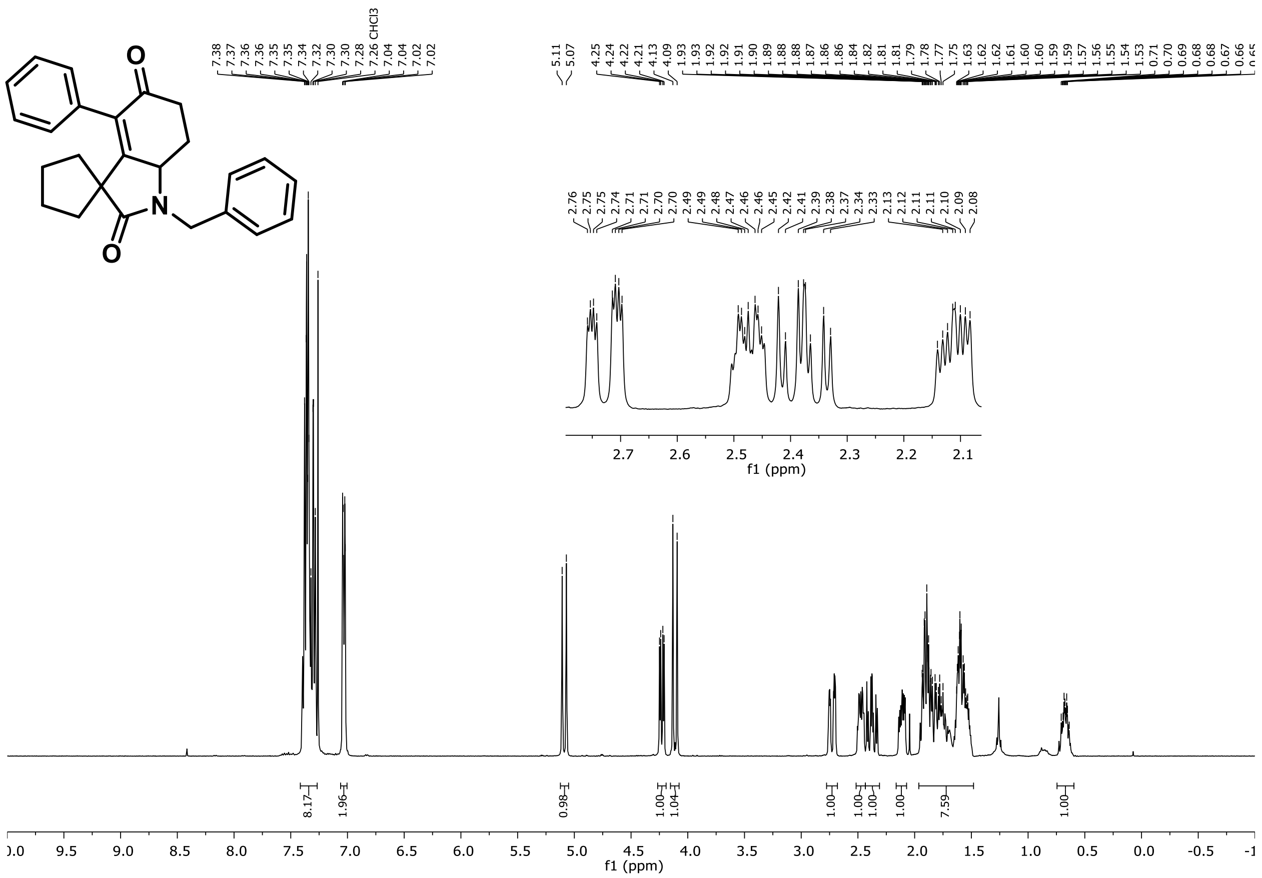

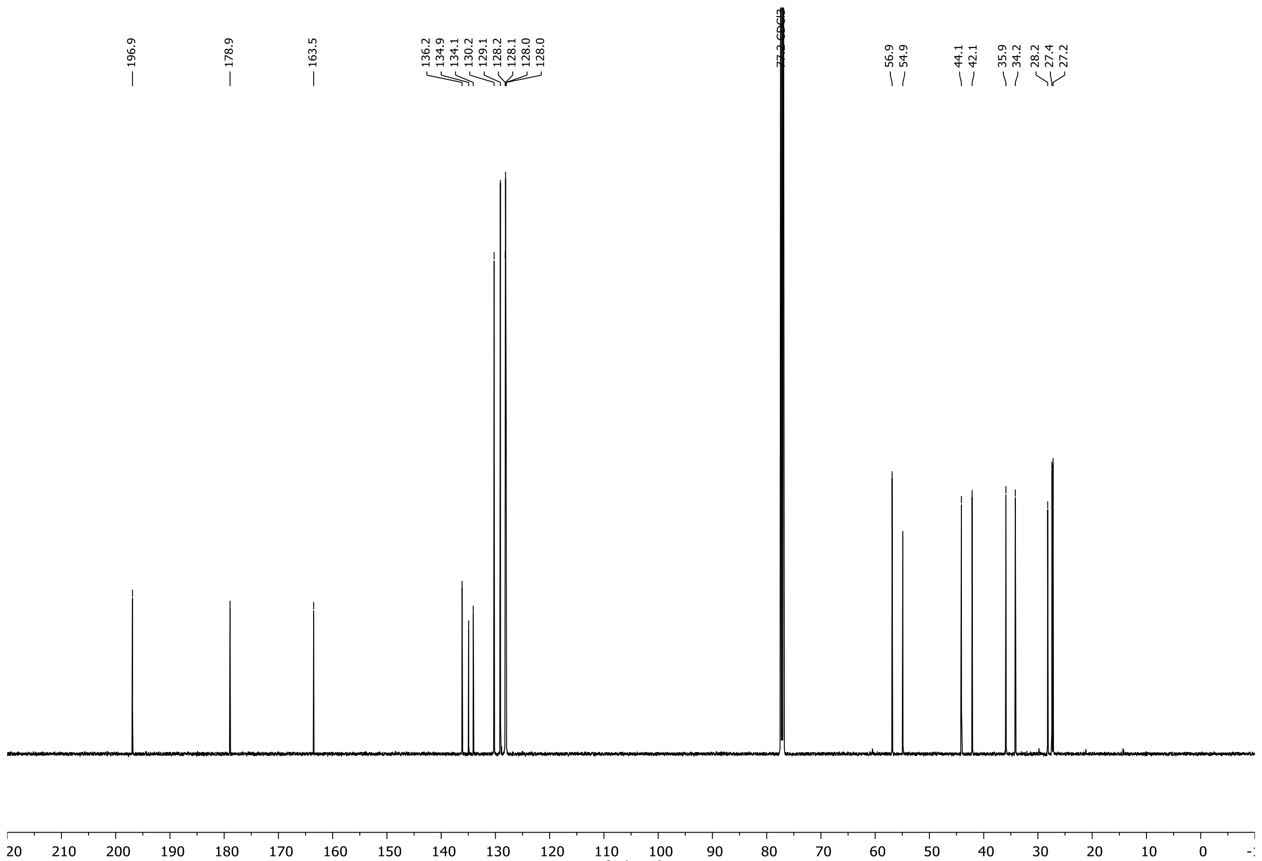

1-(Phenylethynyl)cyclohexane-1-carboxylic acid

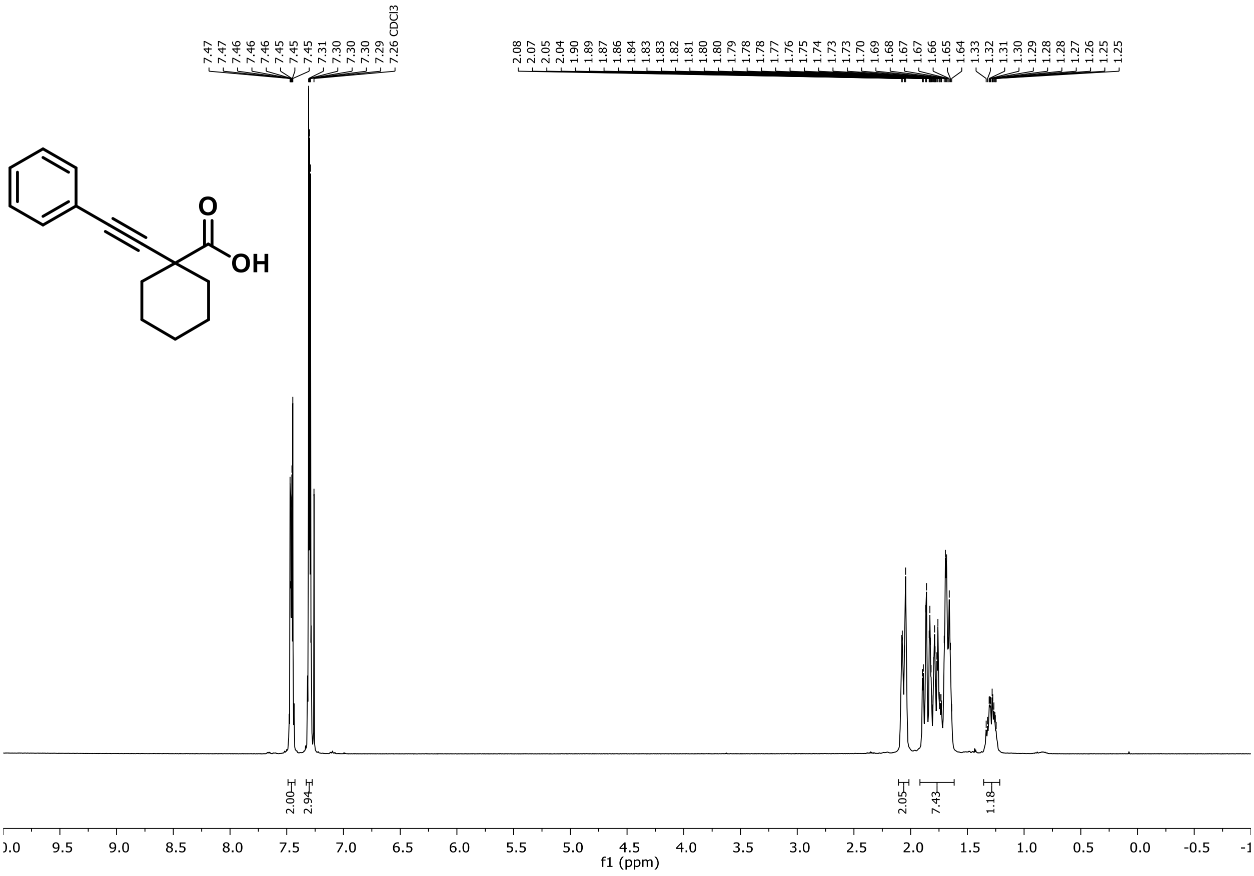

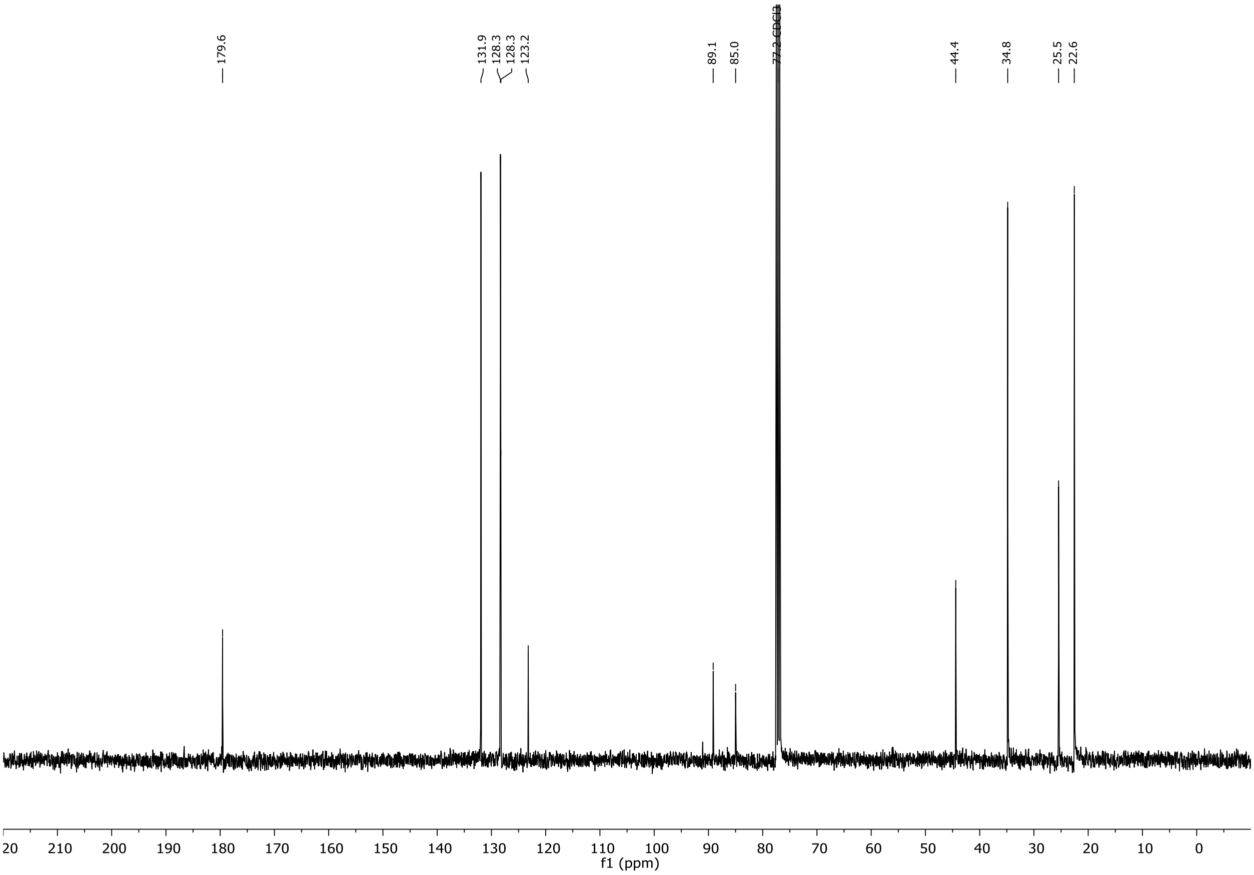

N-Benzyl-*N*-cyclopropyl-1-(phenylethynyl)cyclohexane-1-carboxamide (3d)

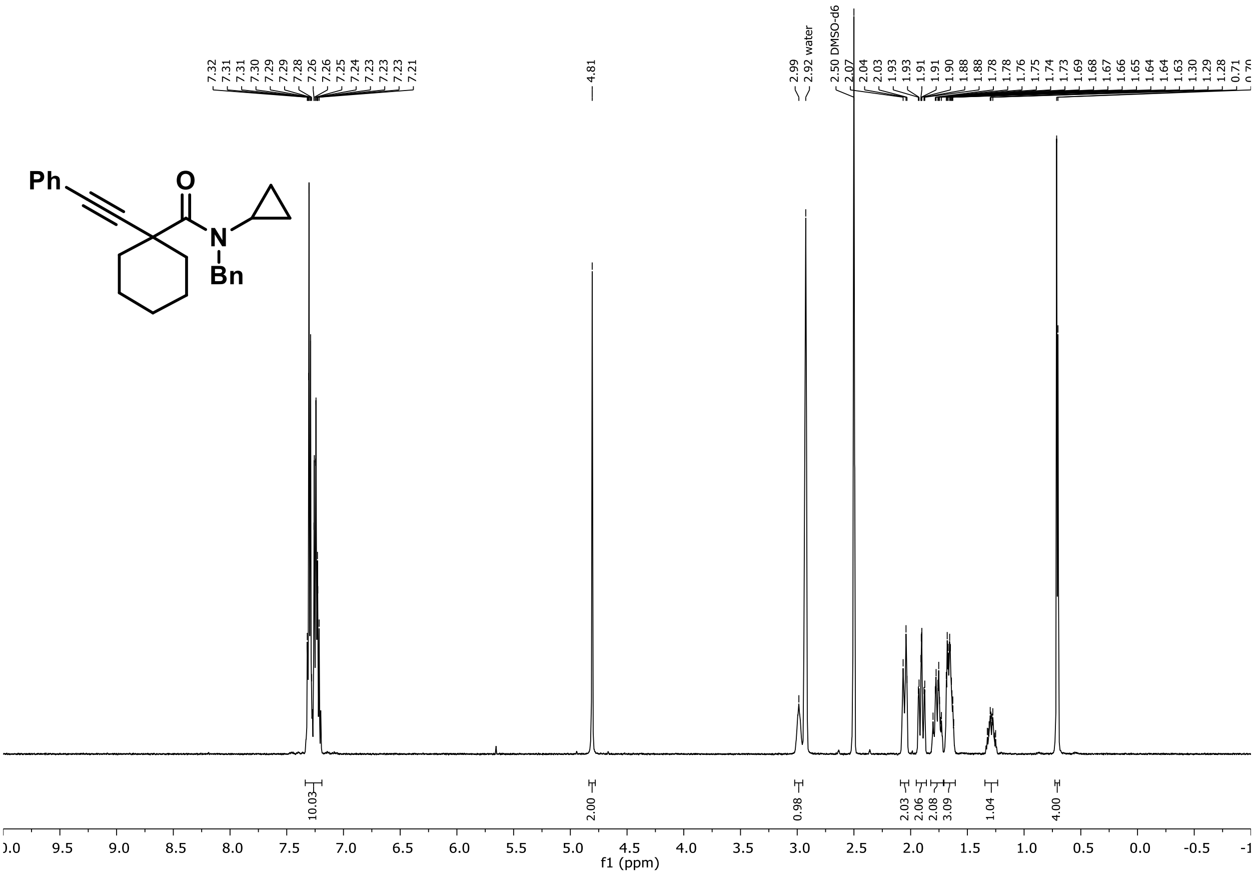

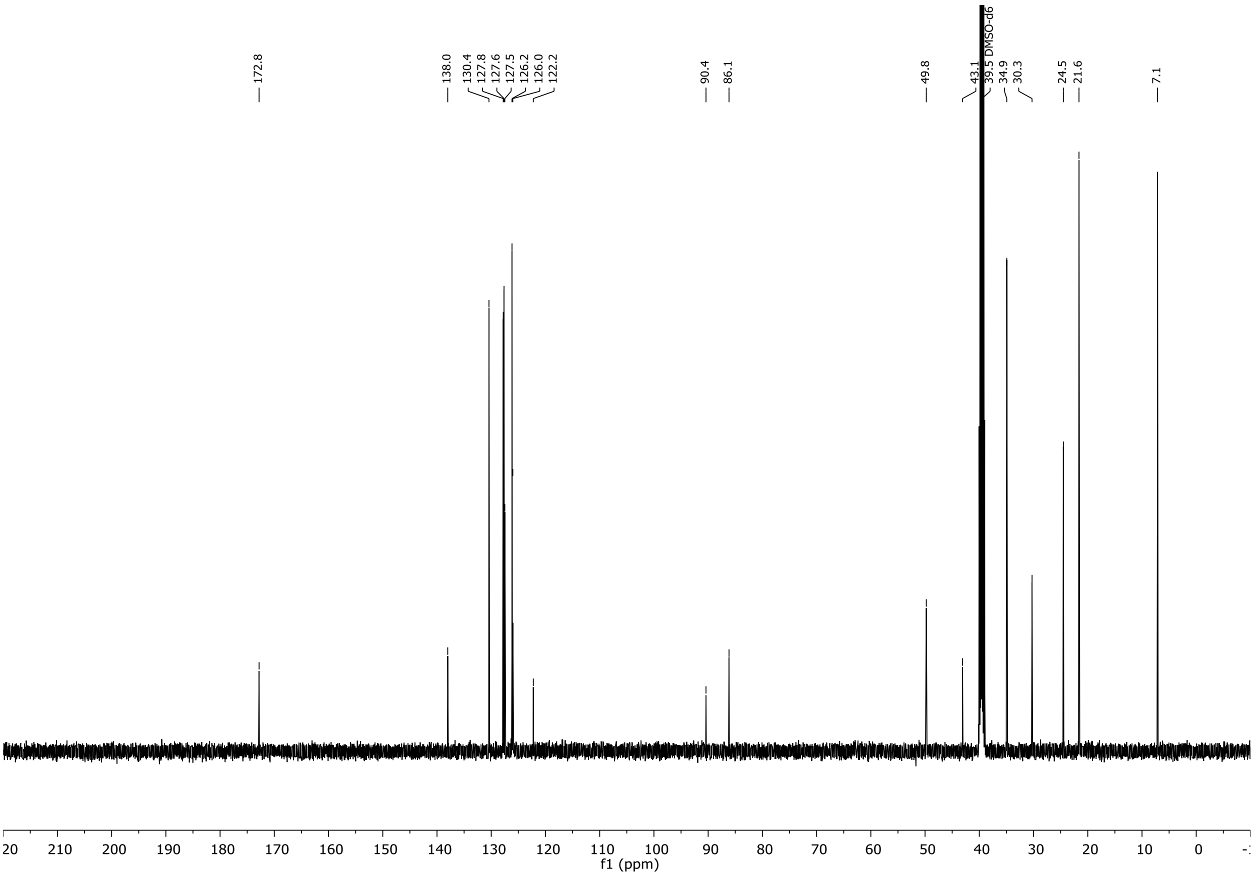

1'-Benzyl-4'-phenyl-1',6',7',7a'-tetrahydrospiro[cyclohexane-1,3'-indole]-2',5'-dione (4d)

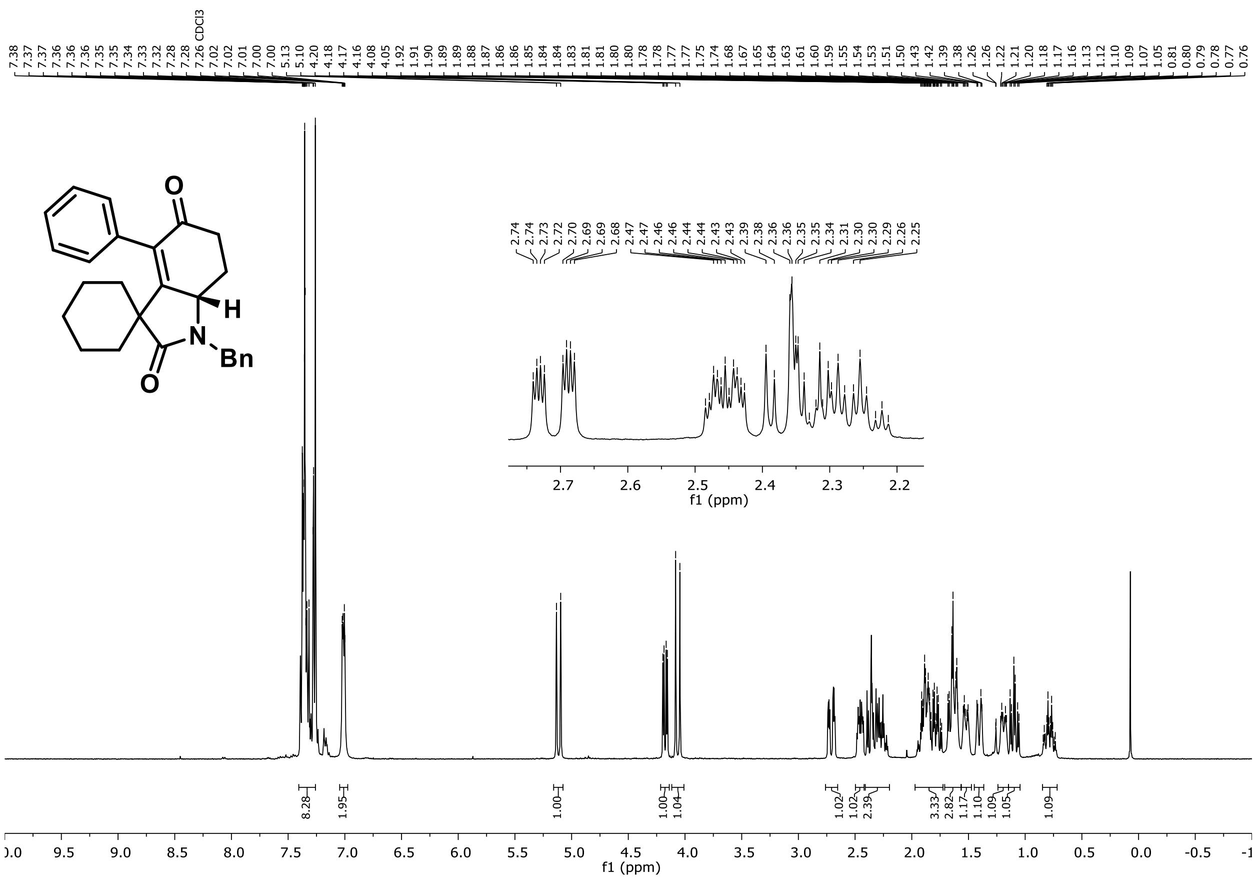

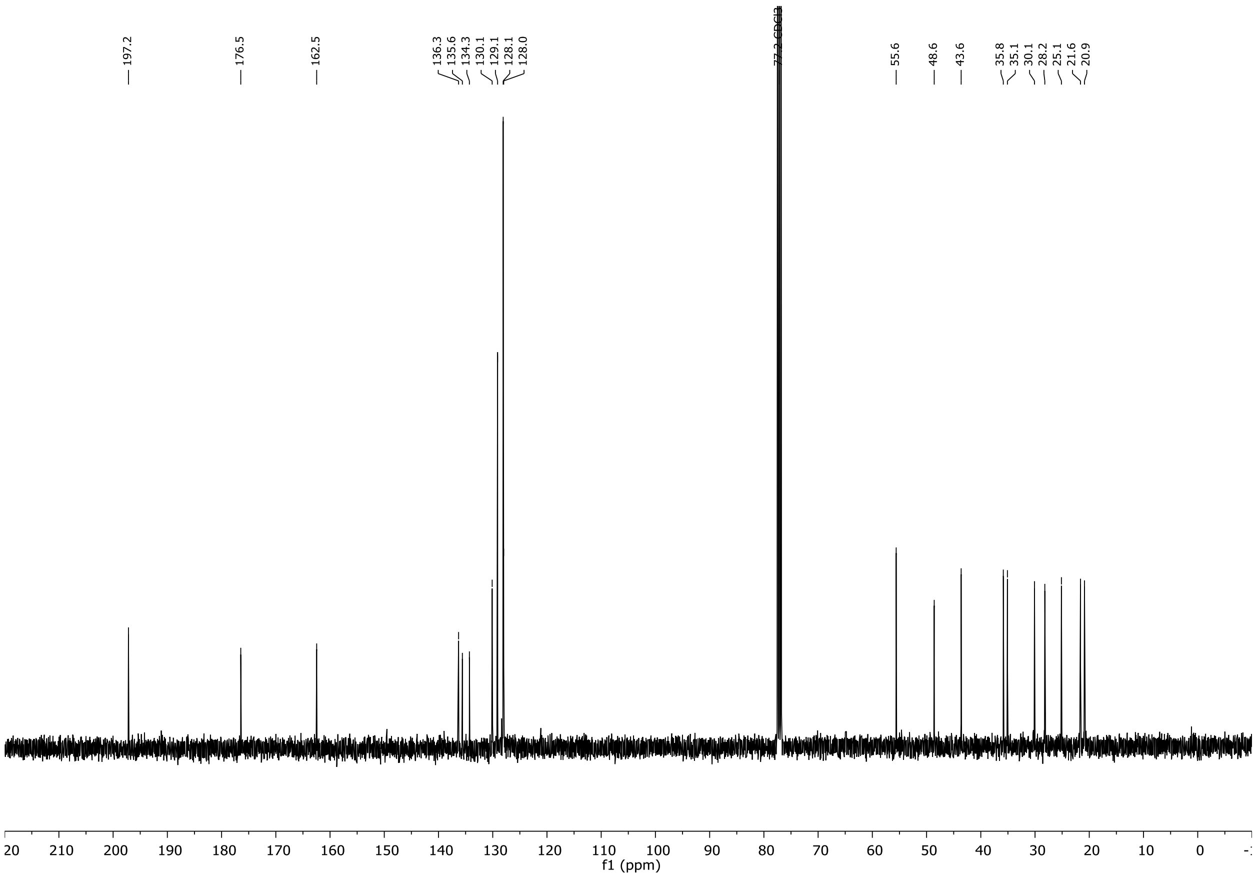

1'-Benzyl-5'-hydroxy-4'-phenylspiro[cyclohexane-1,3'-indolin]-2'-one (4d')

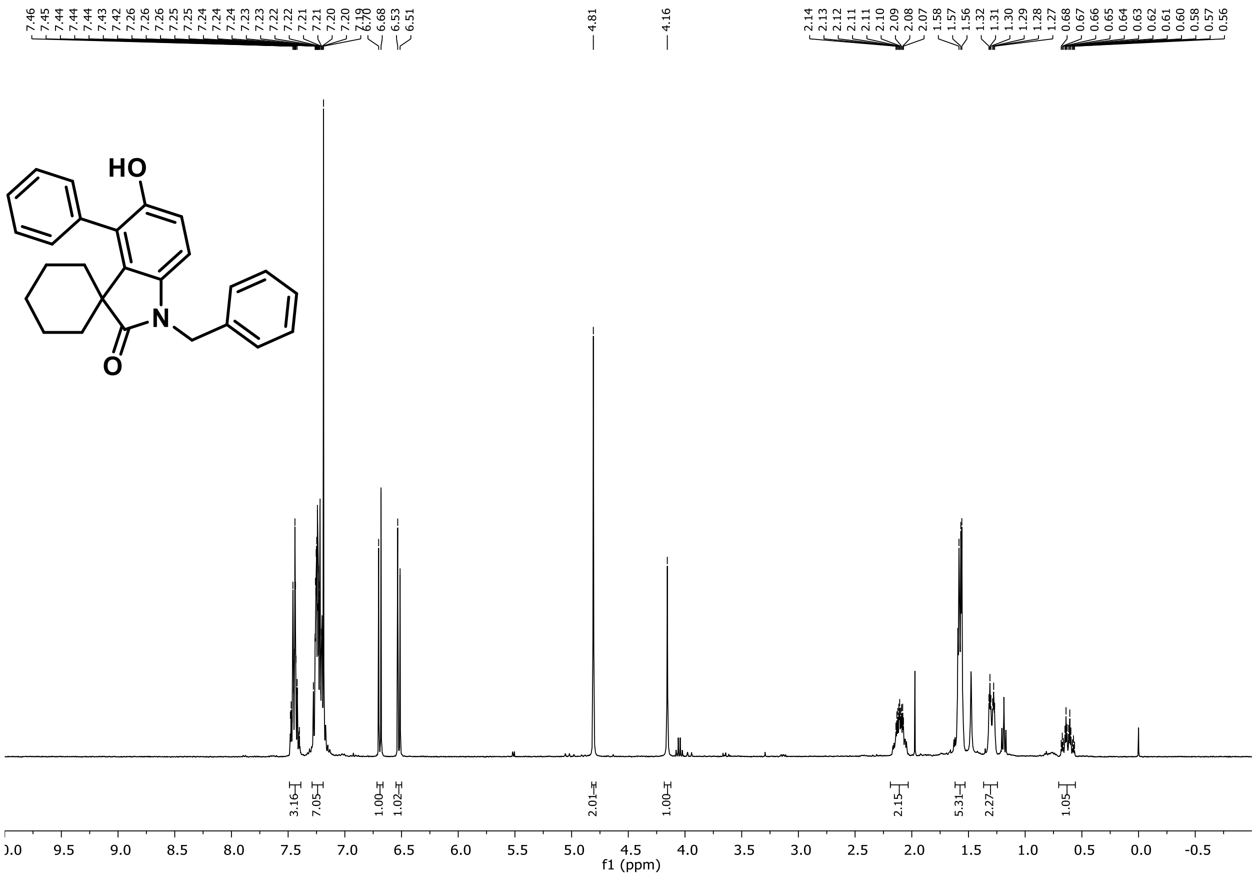

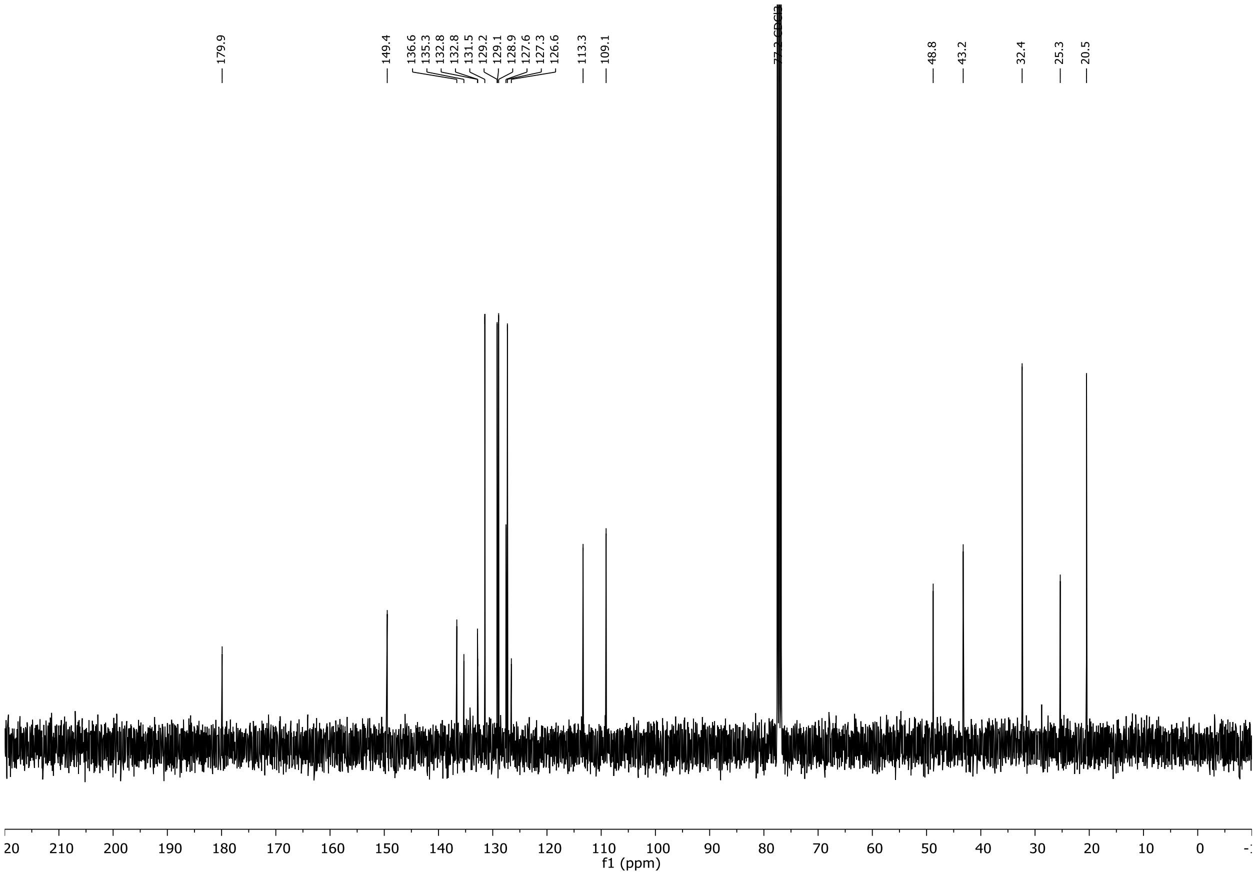

N-benzyl-*N*-cyclopropyl-2,2-dimethyl-4-(trimethylsilyl)but-3-ynamide (3j)

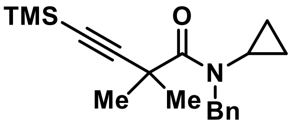

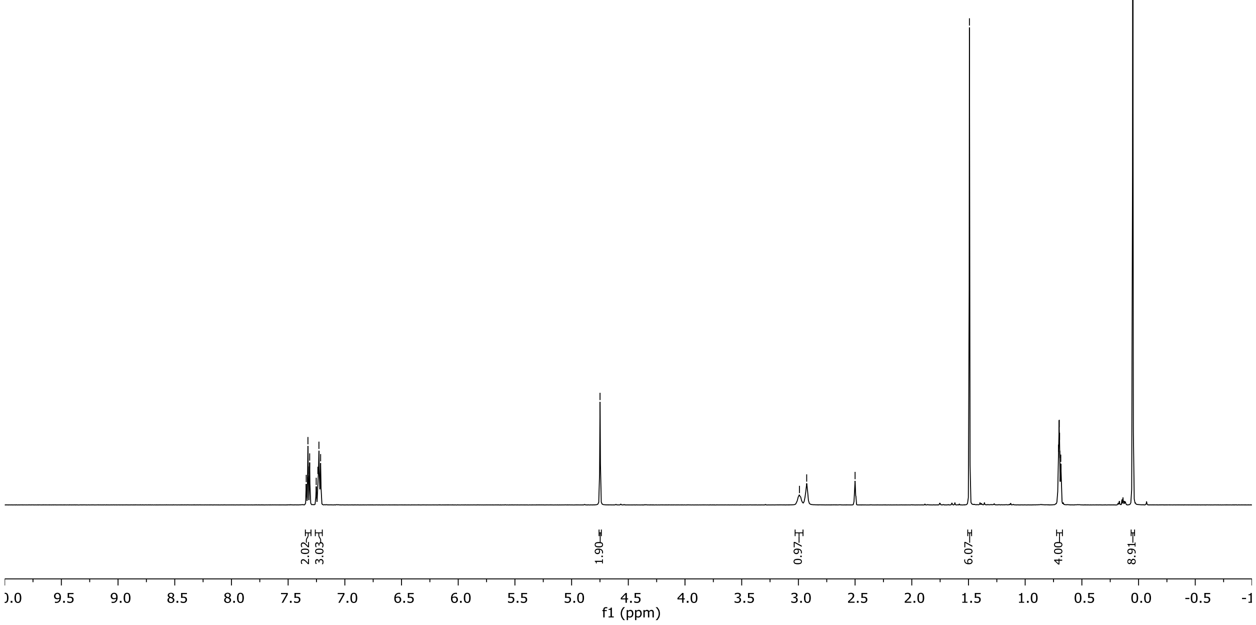

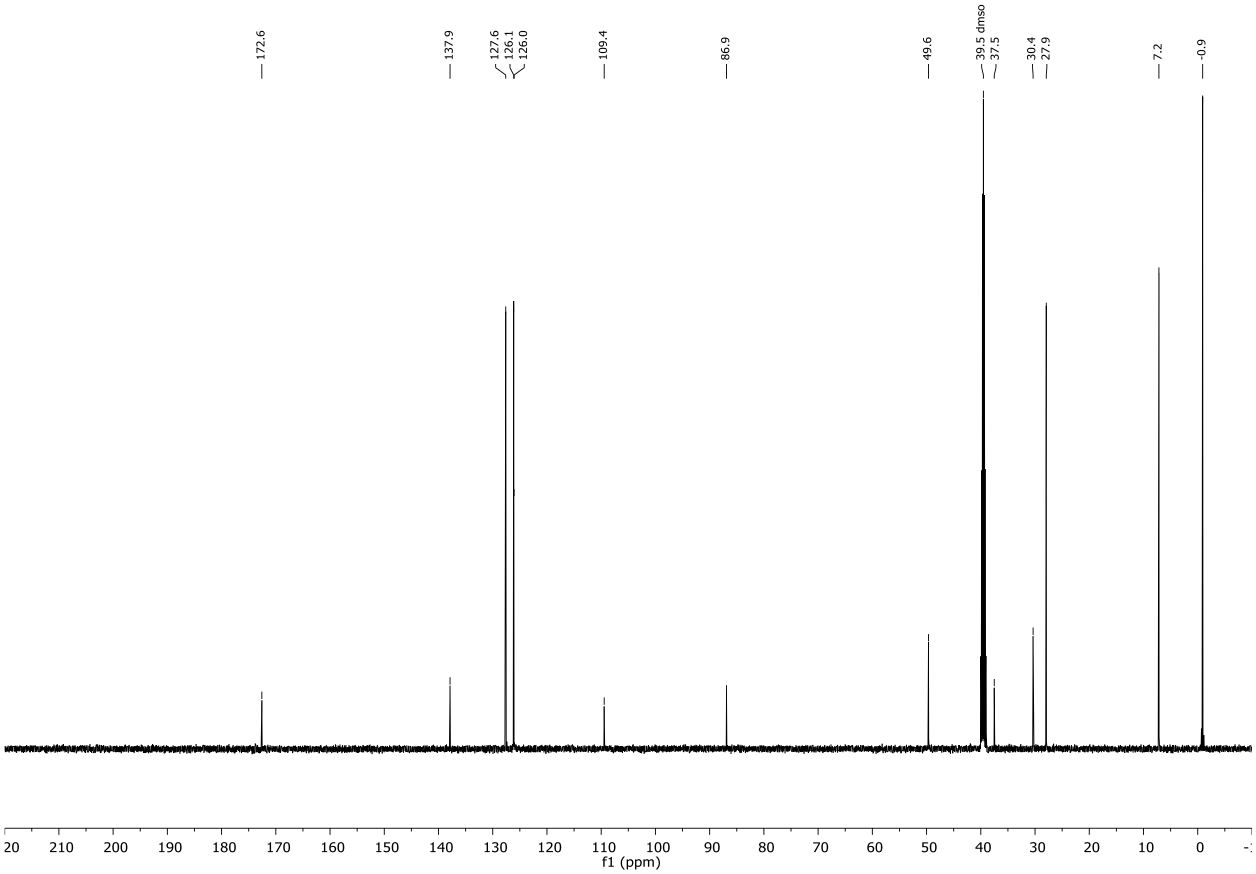

N-Benzyl-*N*-cyclopropyl-2,2-dimethylbut-3-ynamide (3j')

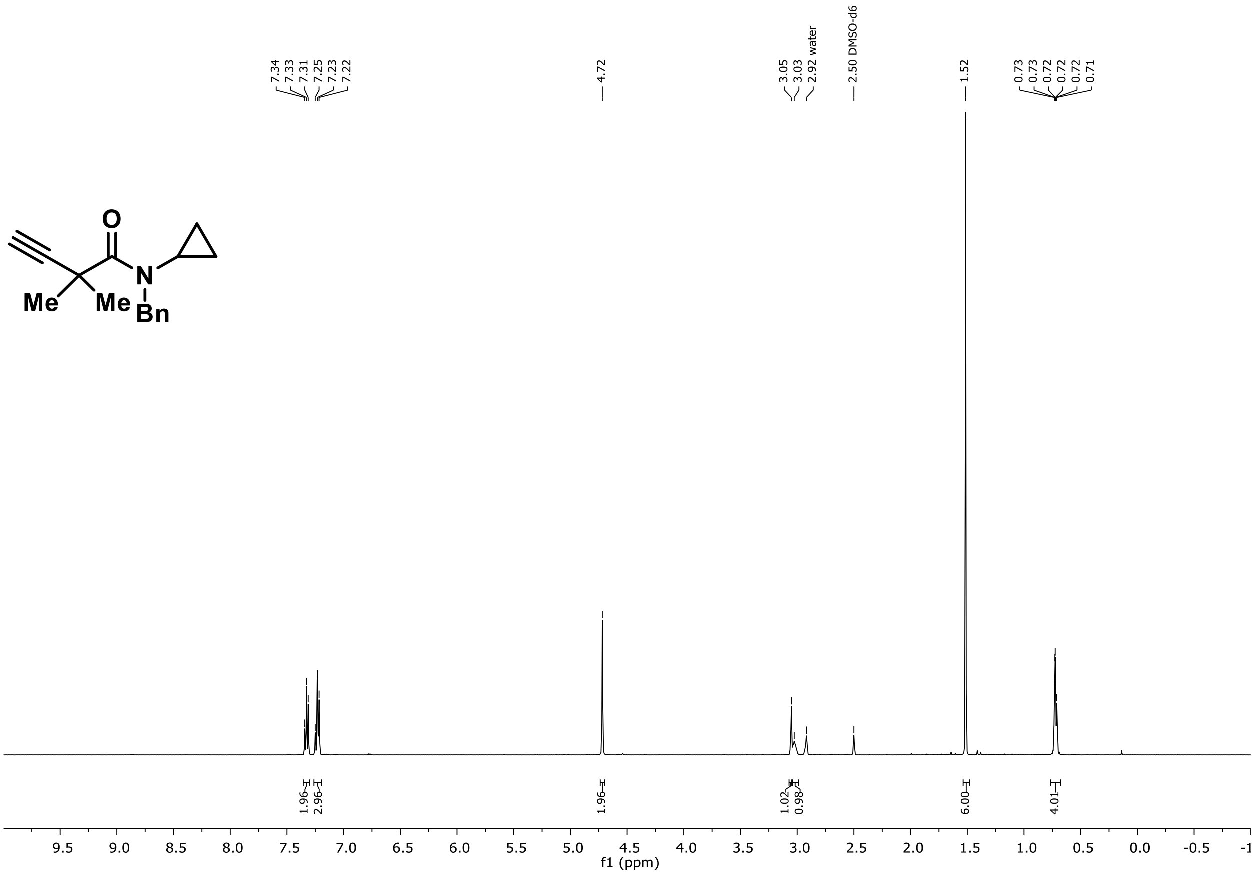

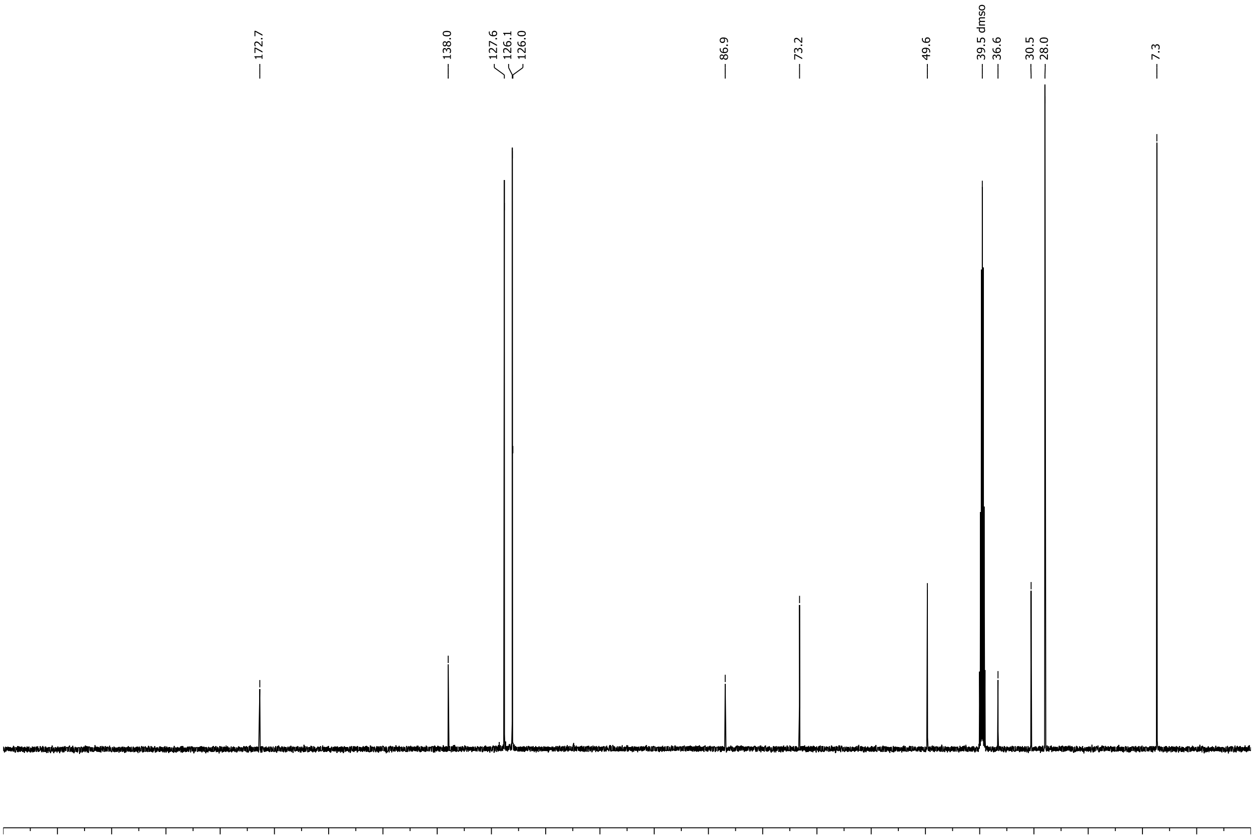

N-Benzyl-*N*-cyclopropyl-4-(4-methoxyphenyl)-2,2-dimethylbut-3-ynamide (3e)

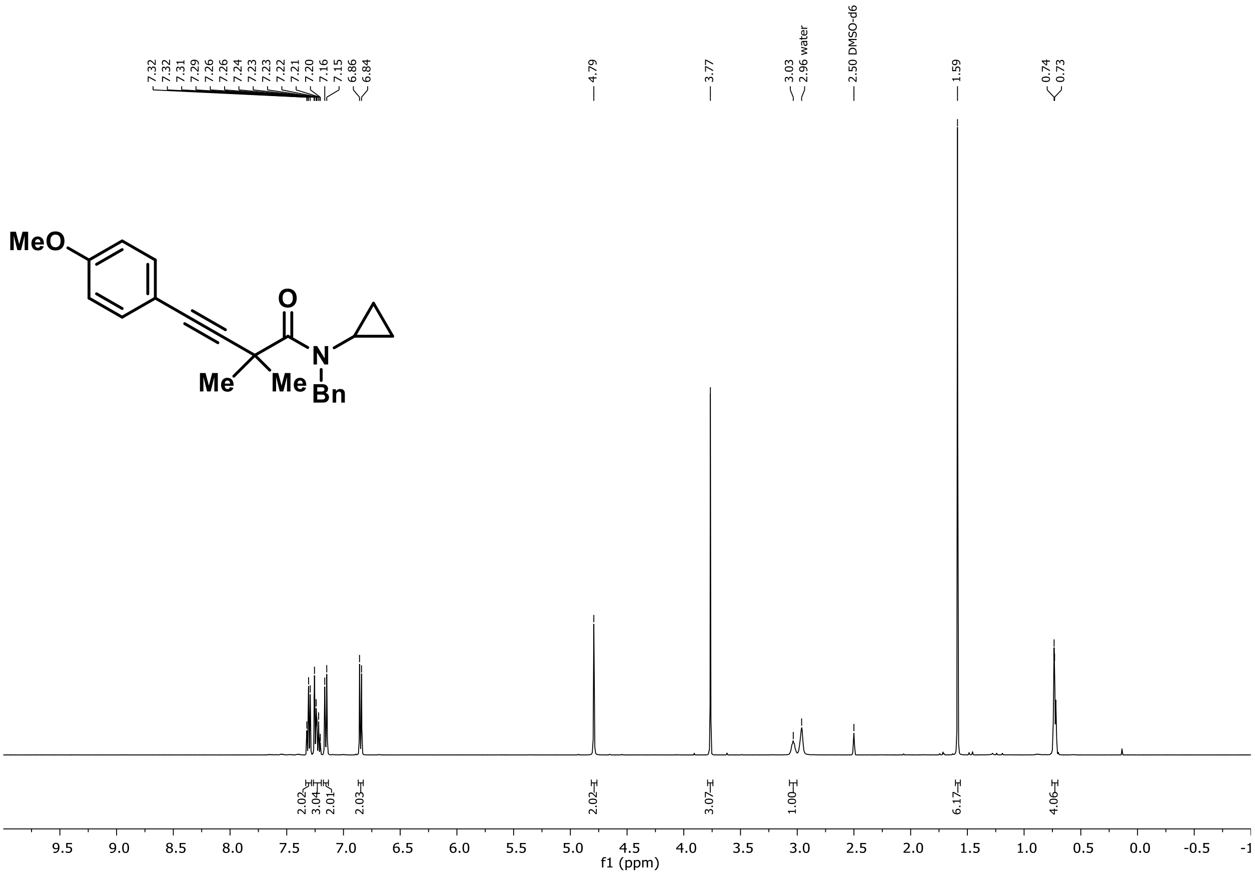

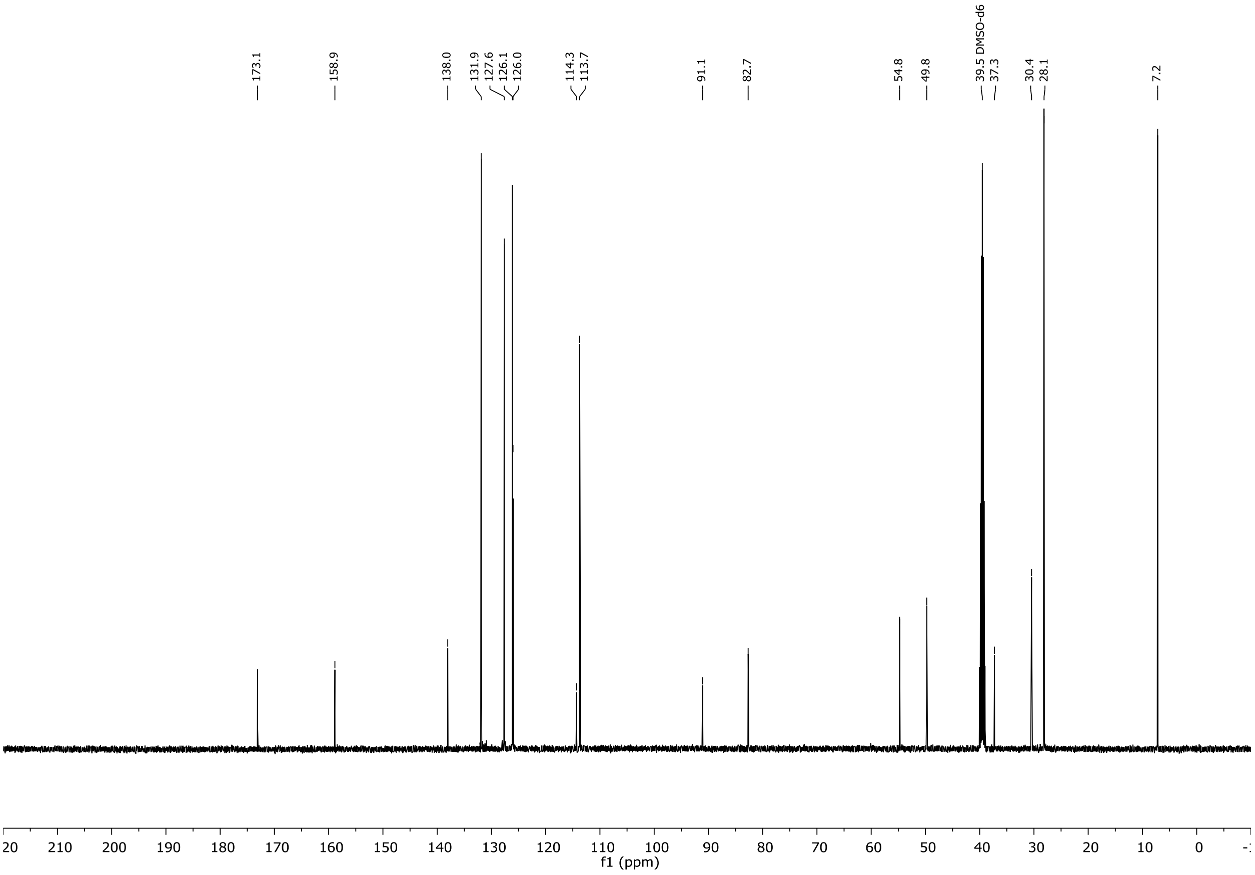

1-Benzyl-4-(4-methoxyphenyl)-3,3-dimethyl-1,6,7,7a-tetrahydro-2*H*-indole-2,5(3*H*)-dione (4e)

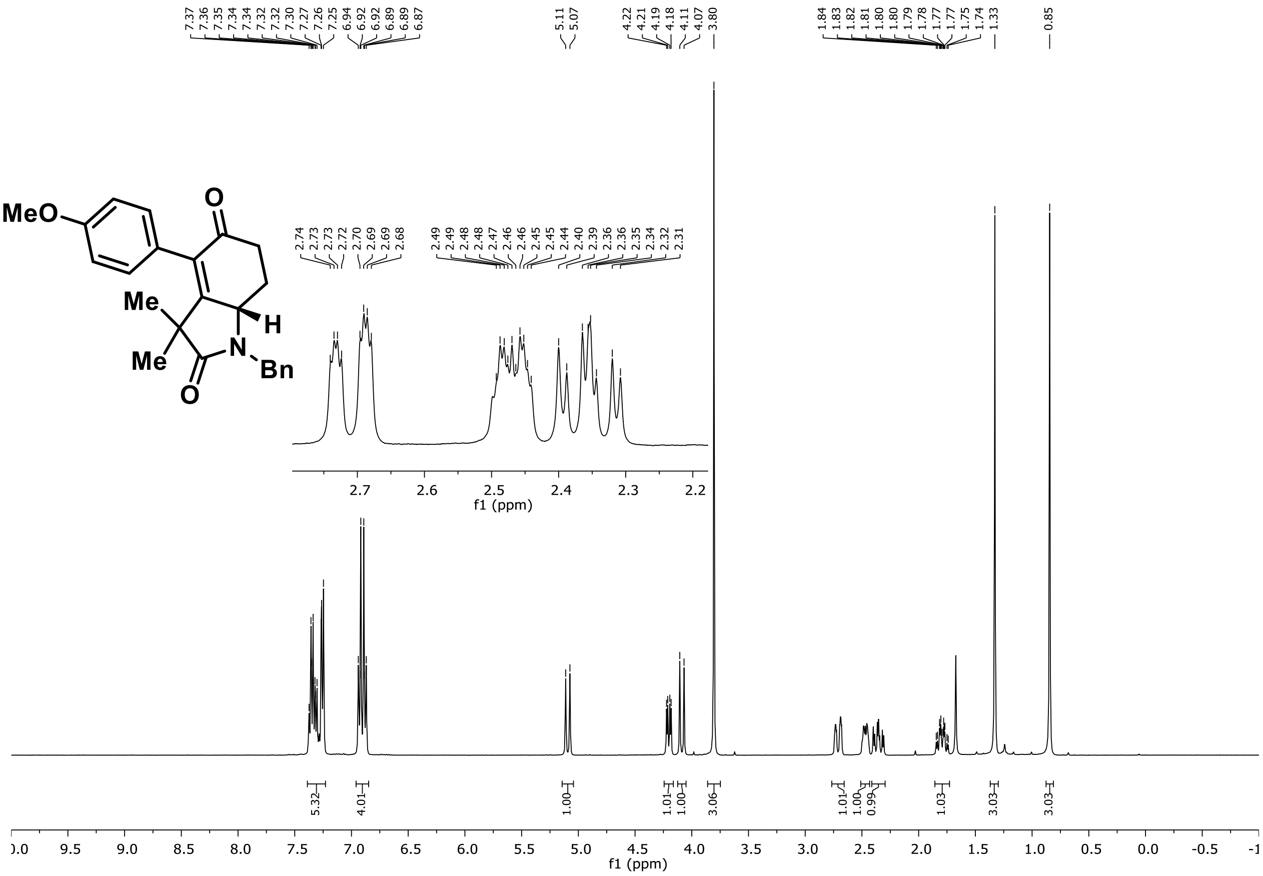

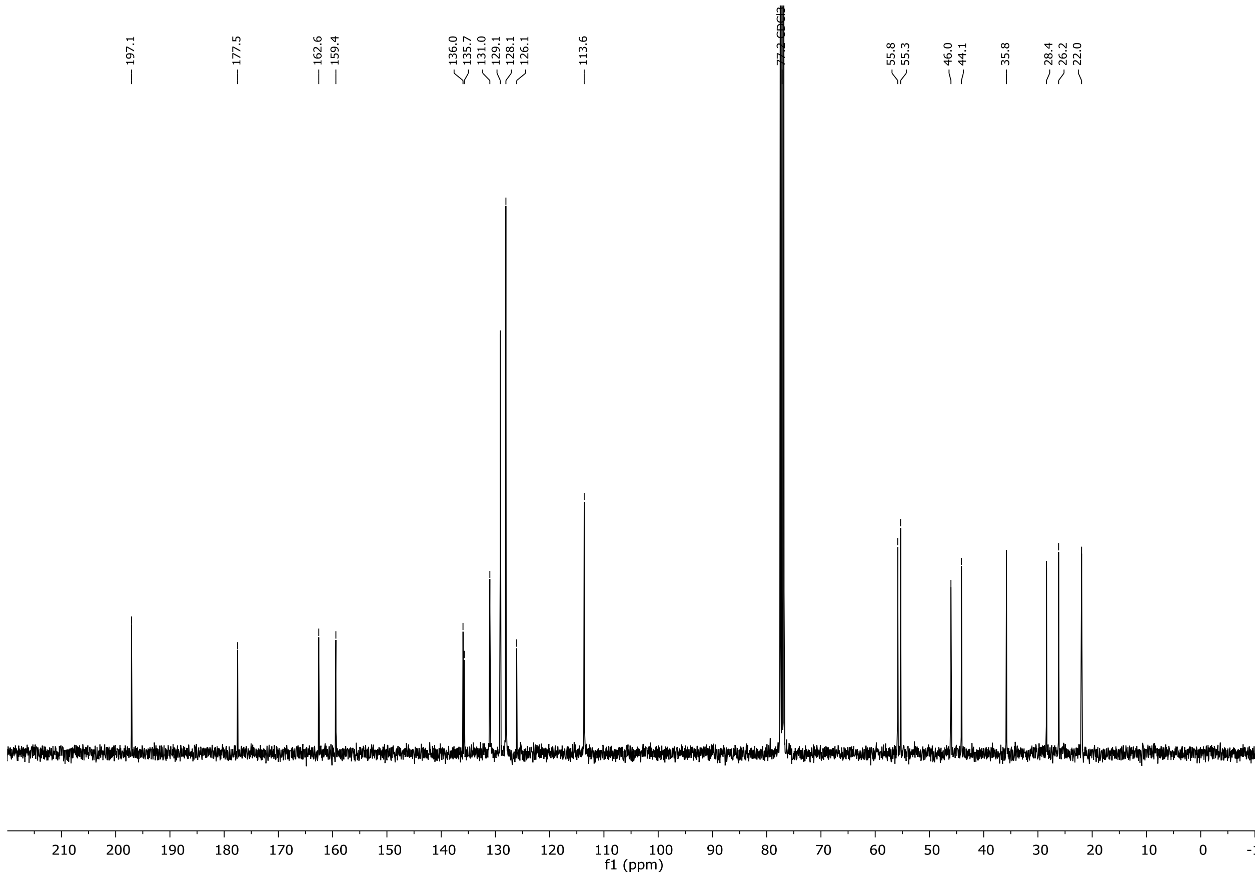

N-Benzyl-4-(4-chlorophenyl)-*N*-cyclopropyl-2,2-dimethylbut-3-ynamide (3f)

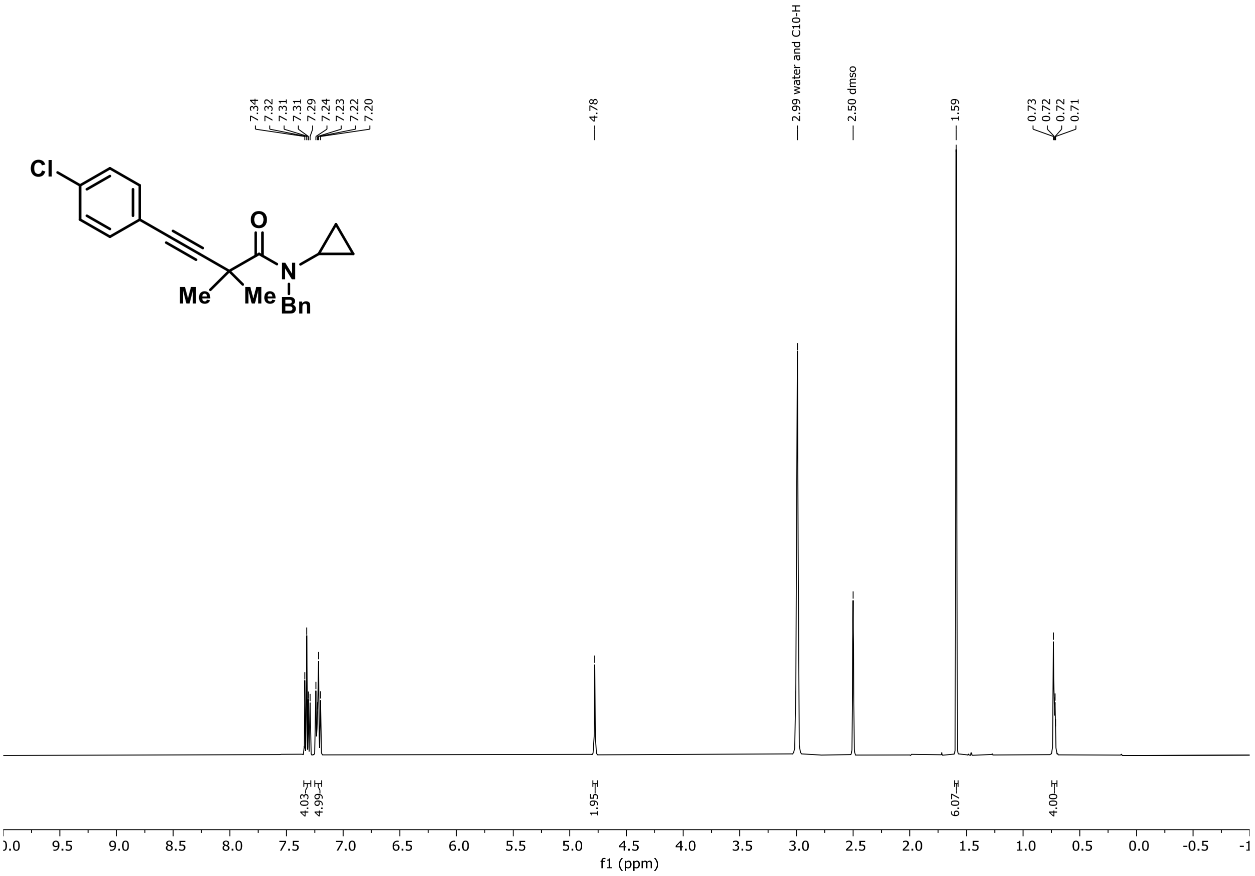

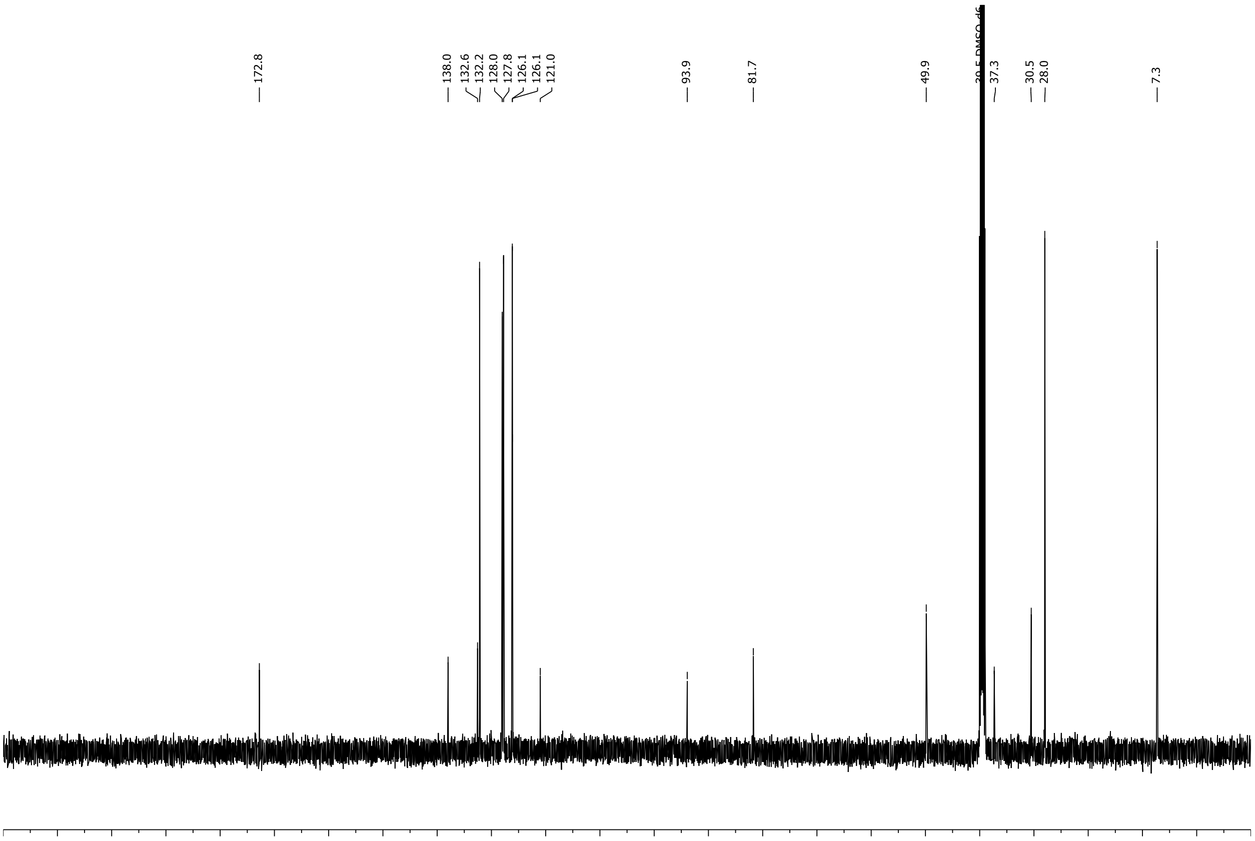

1-Benzyl-4-(4-chlorophenyl)-3,3-dimethyl-1,6,7,7a-tetrahydro-2*H*-indole-2,5(3*H*)-dione (4f)

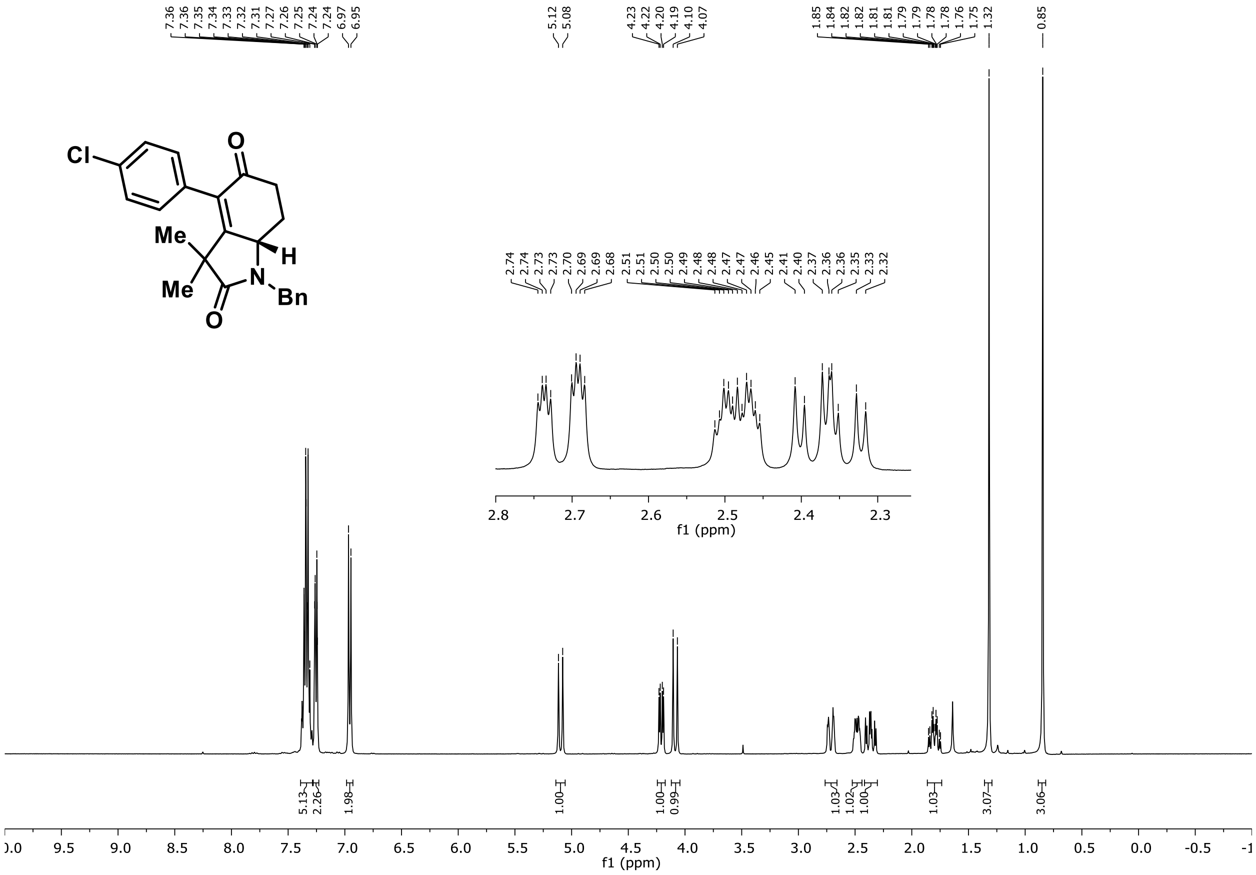

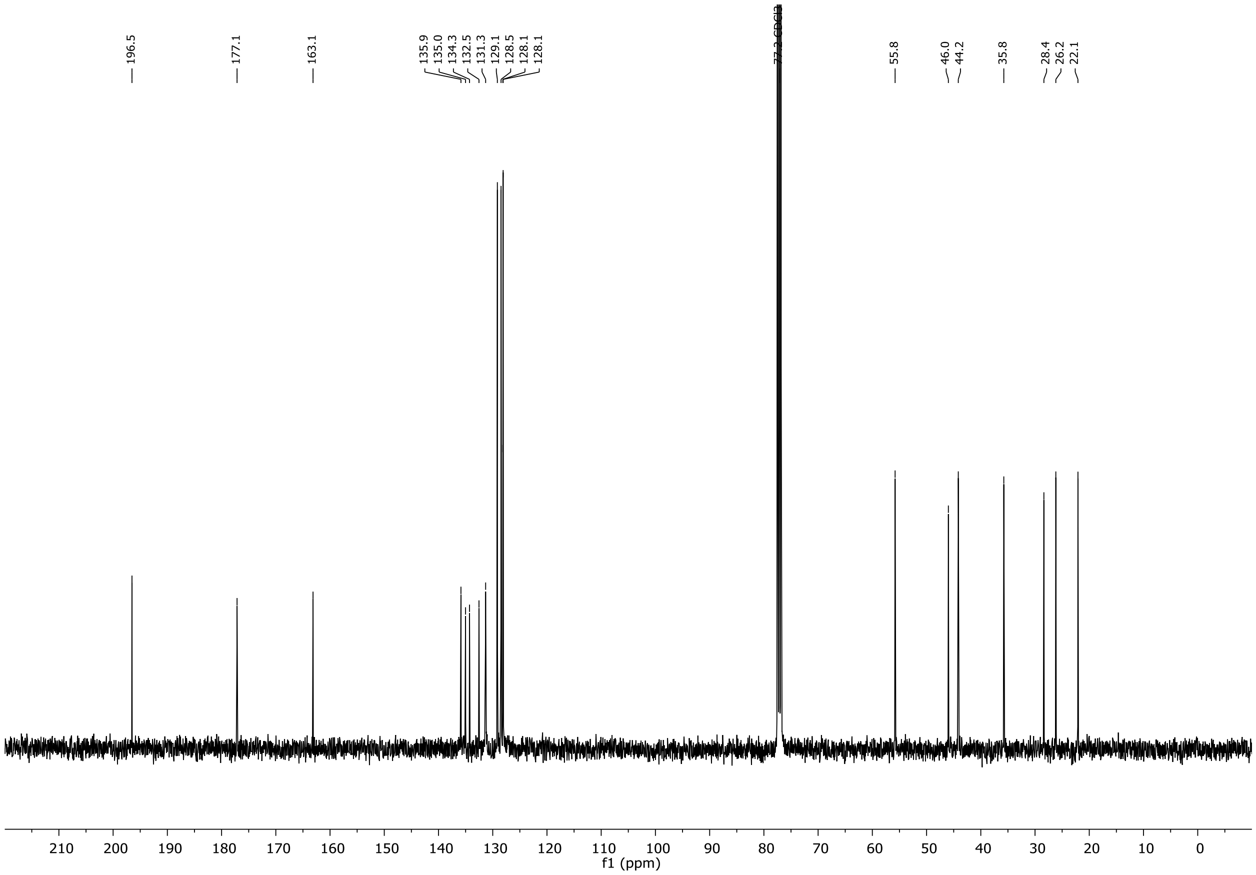

N-Benzyl-*N*-cyclopropyl-2,2-dimethyl-4-(thiophen-3-yl)but-3-ynamide (3g)

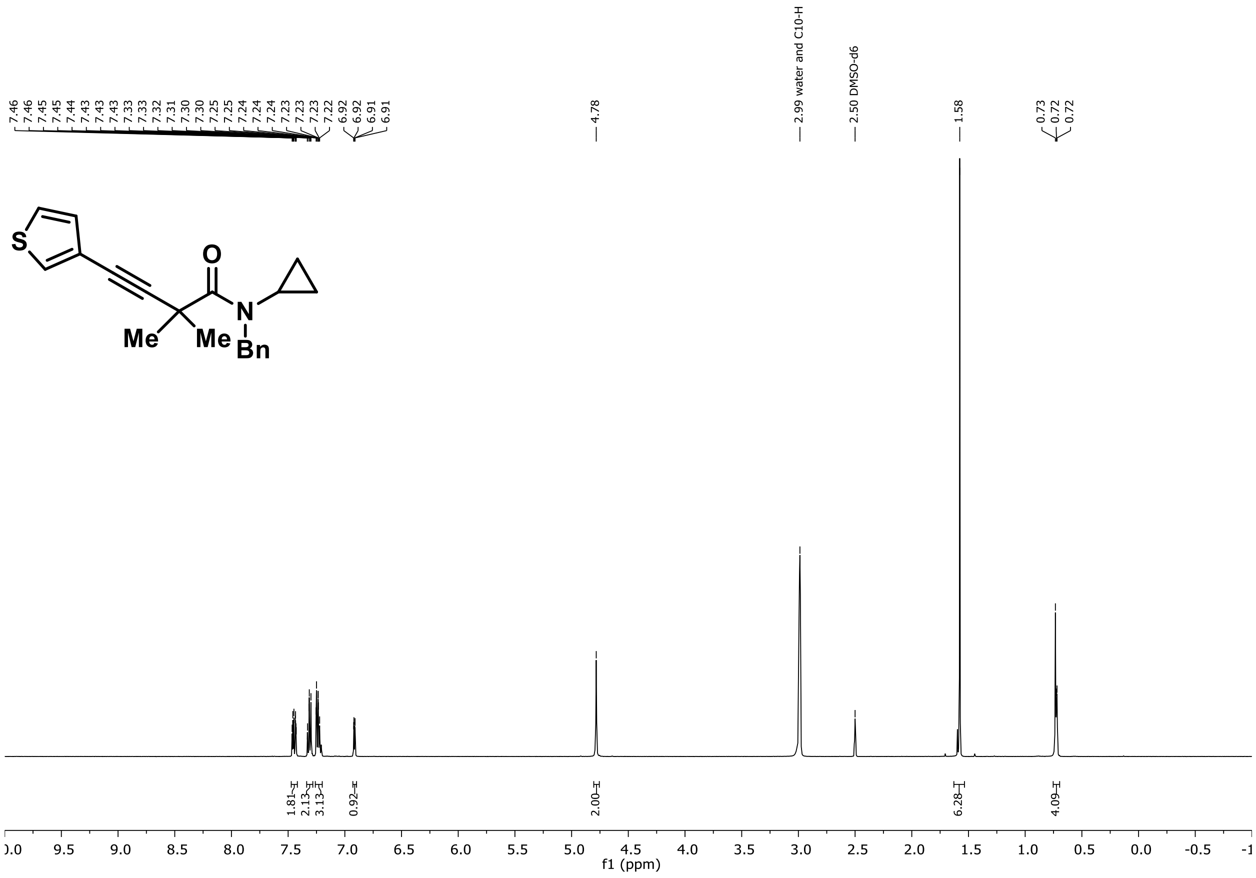

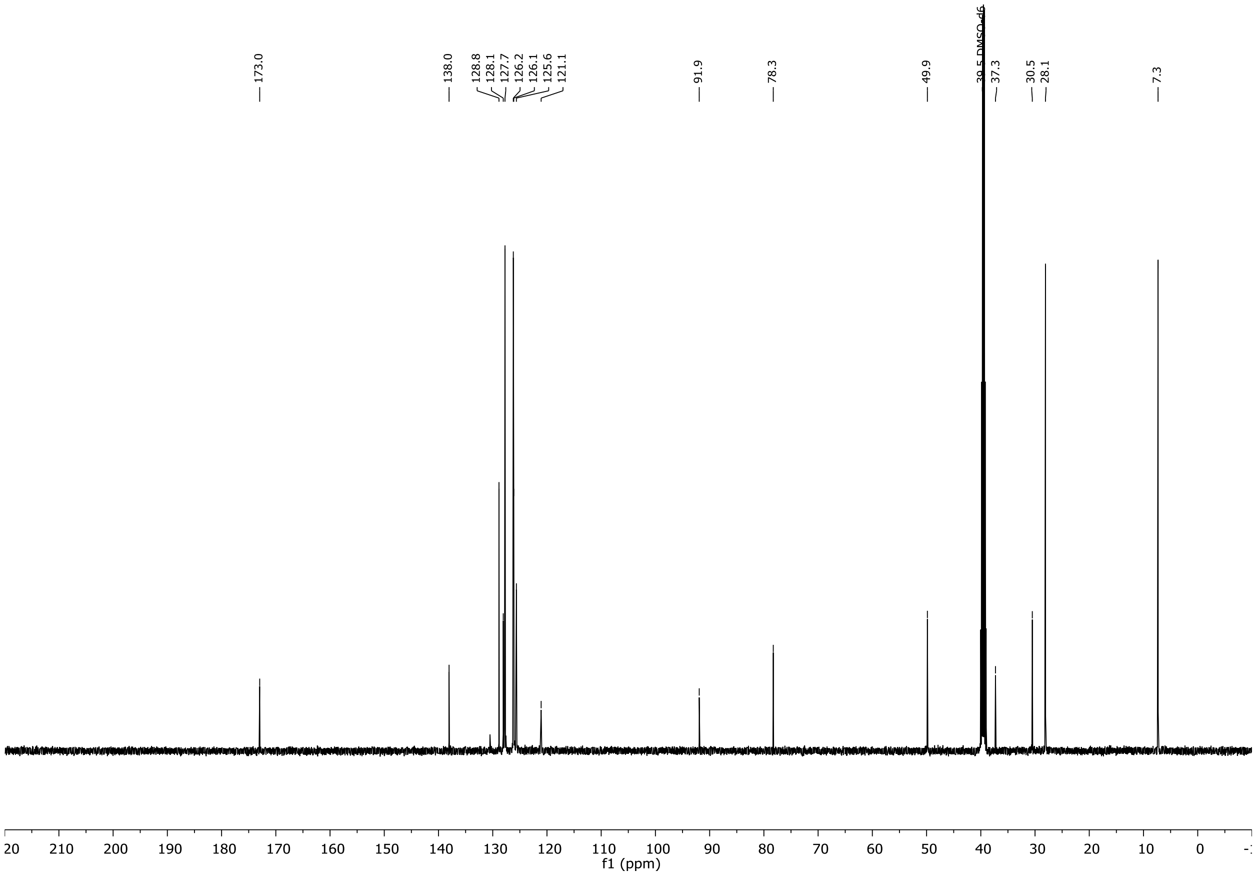

1-Benzyl-3,3-dimethyl-4-(thiophen-3-yl)-1,6,7,7a-tetrahydro-2*H*-indole-2,5(3*H*)-dione (4g)

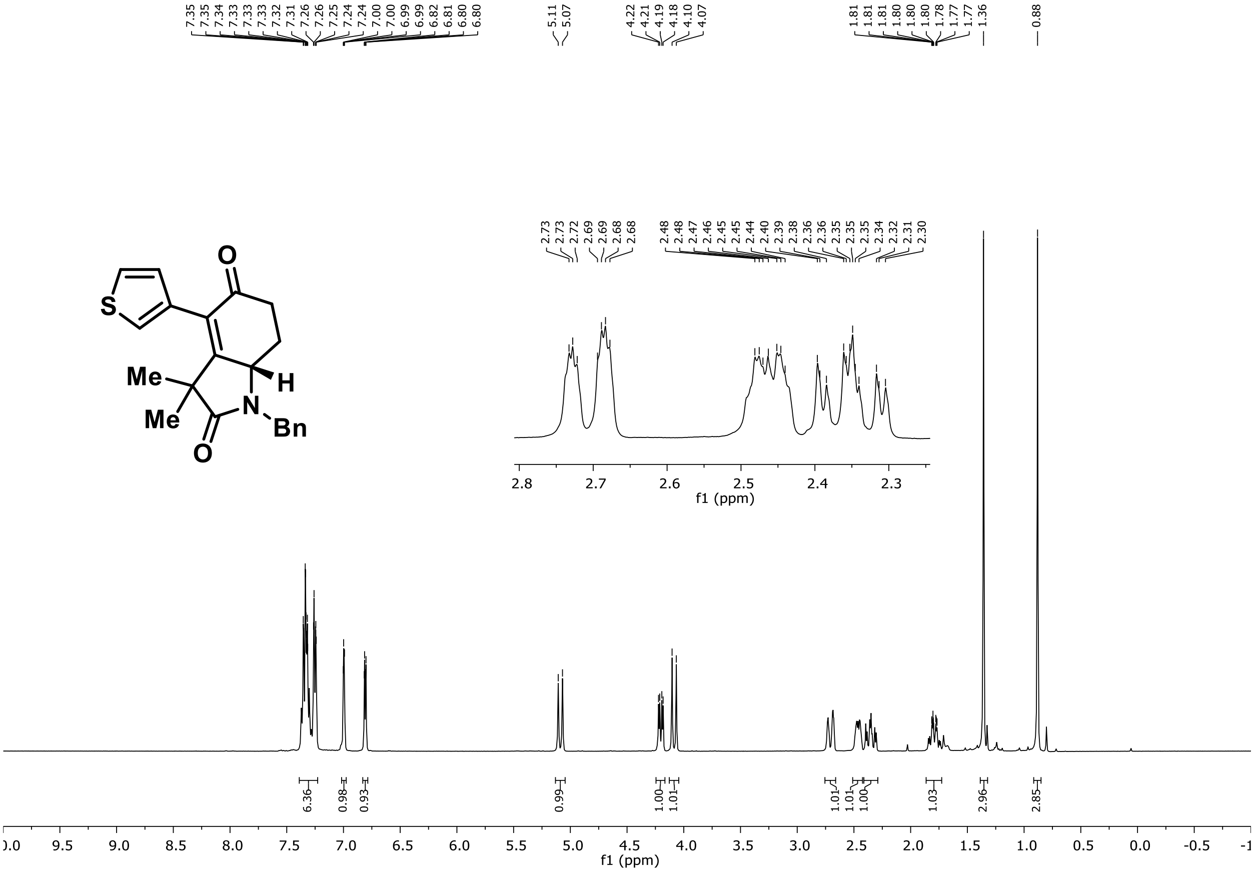

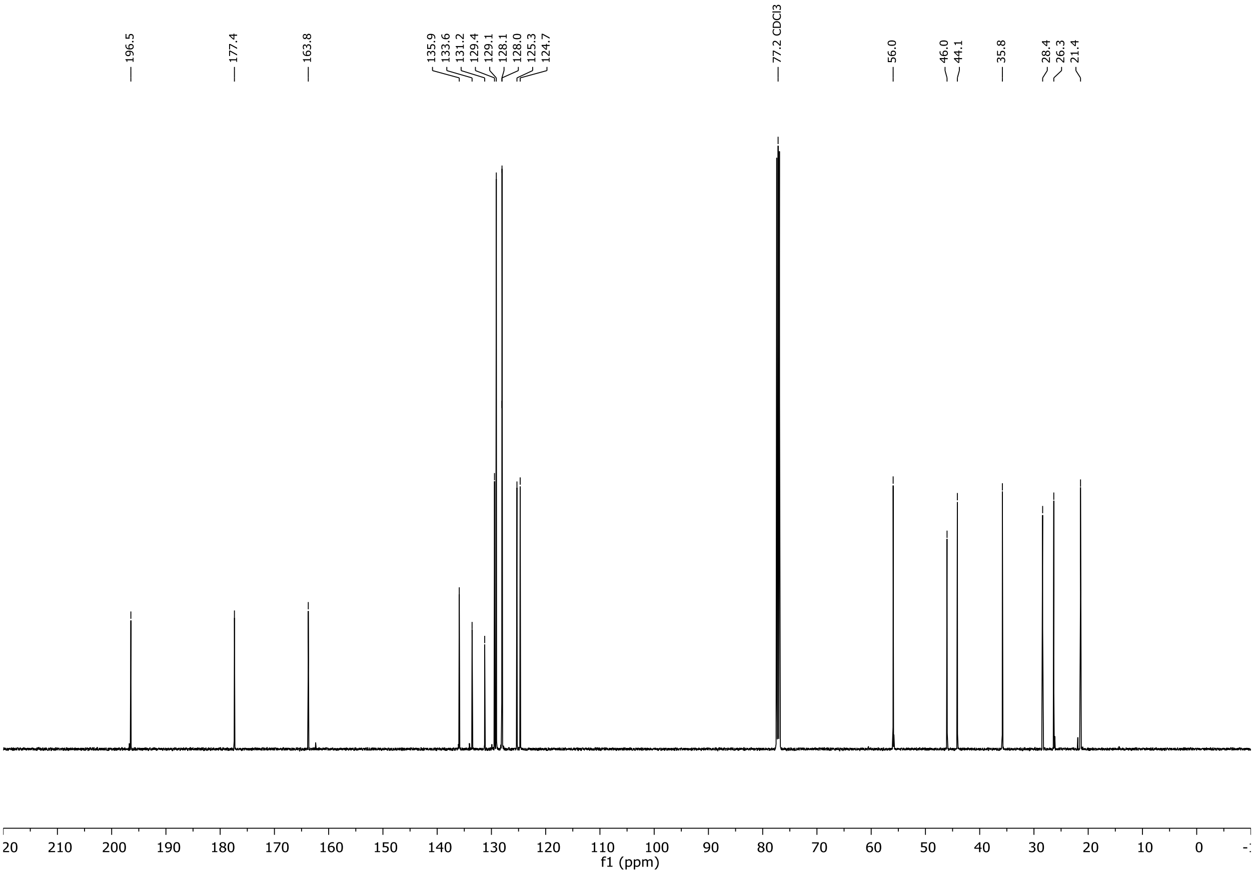

N-Benzyl-N-cyclopropyl-2,2-dimethyl-4-(naphthalen-1-yl)but-3-ynamide (3h)

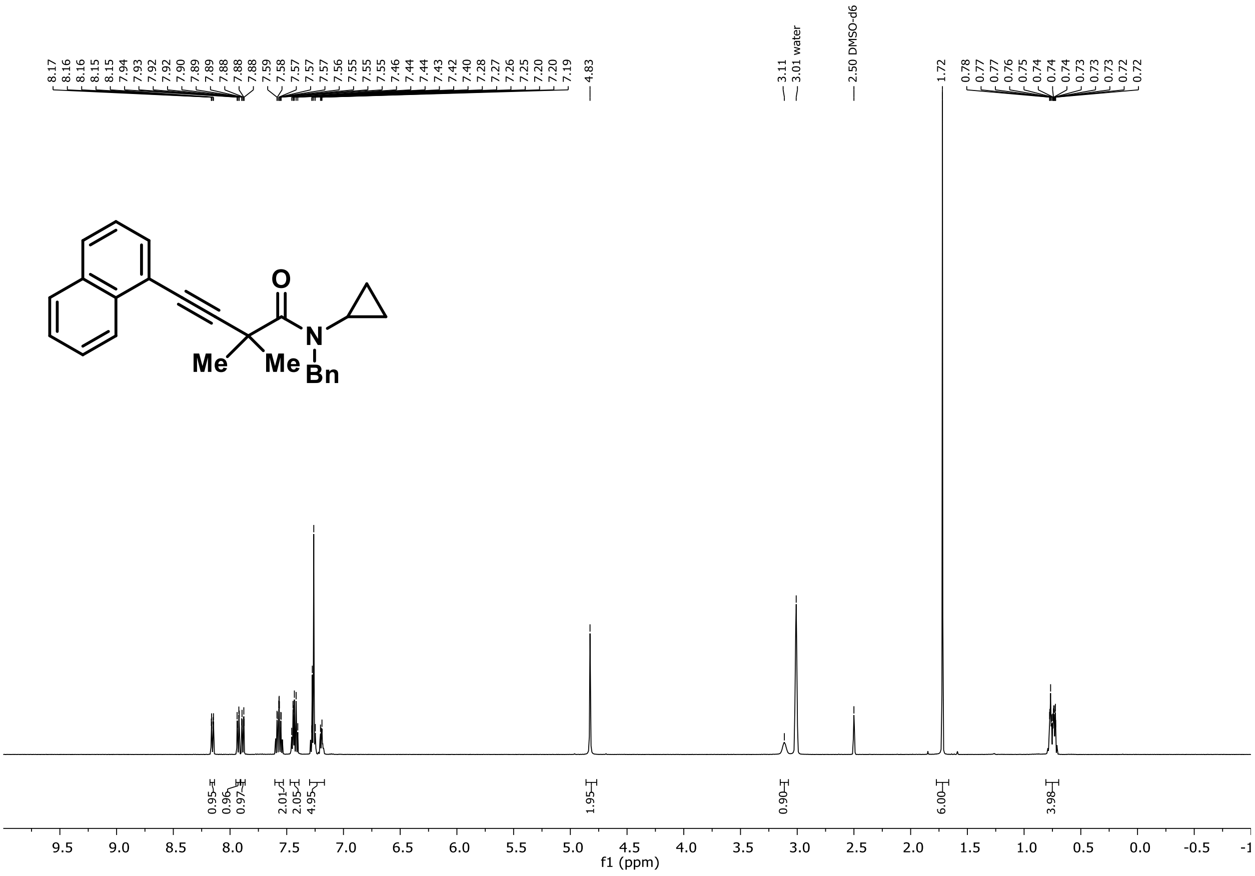

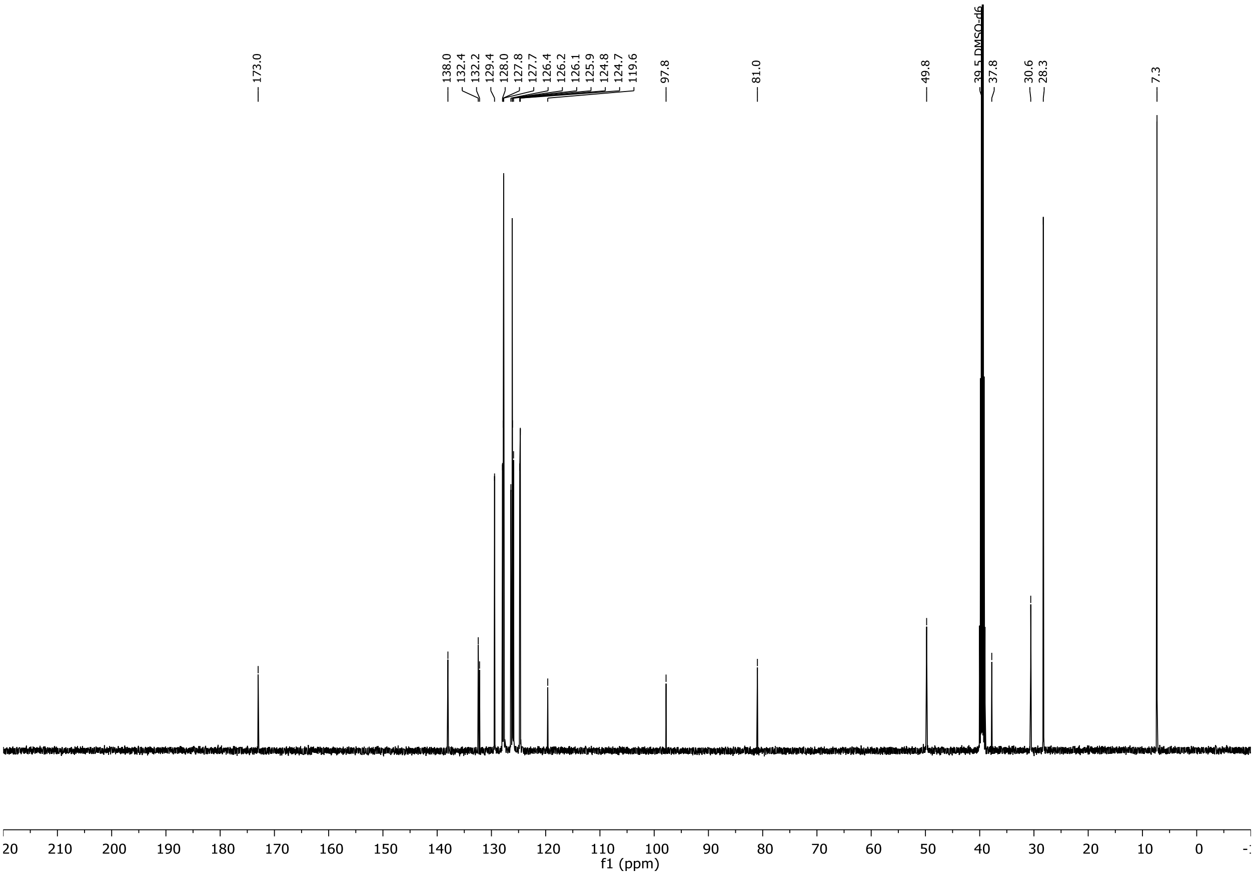

1-Benzyl-3,3-dimethyl-4-(naphthalen-1-yl)-1,6,7,7a-tetrahydro-2*H*-indole-2,5(3*H*)-dione (4h and 4h')

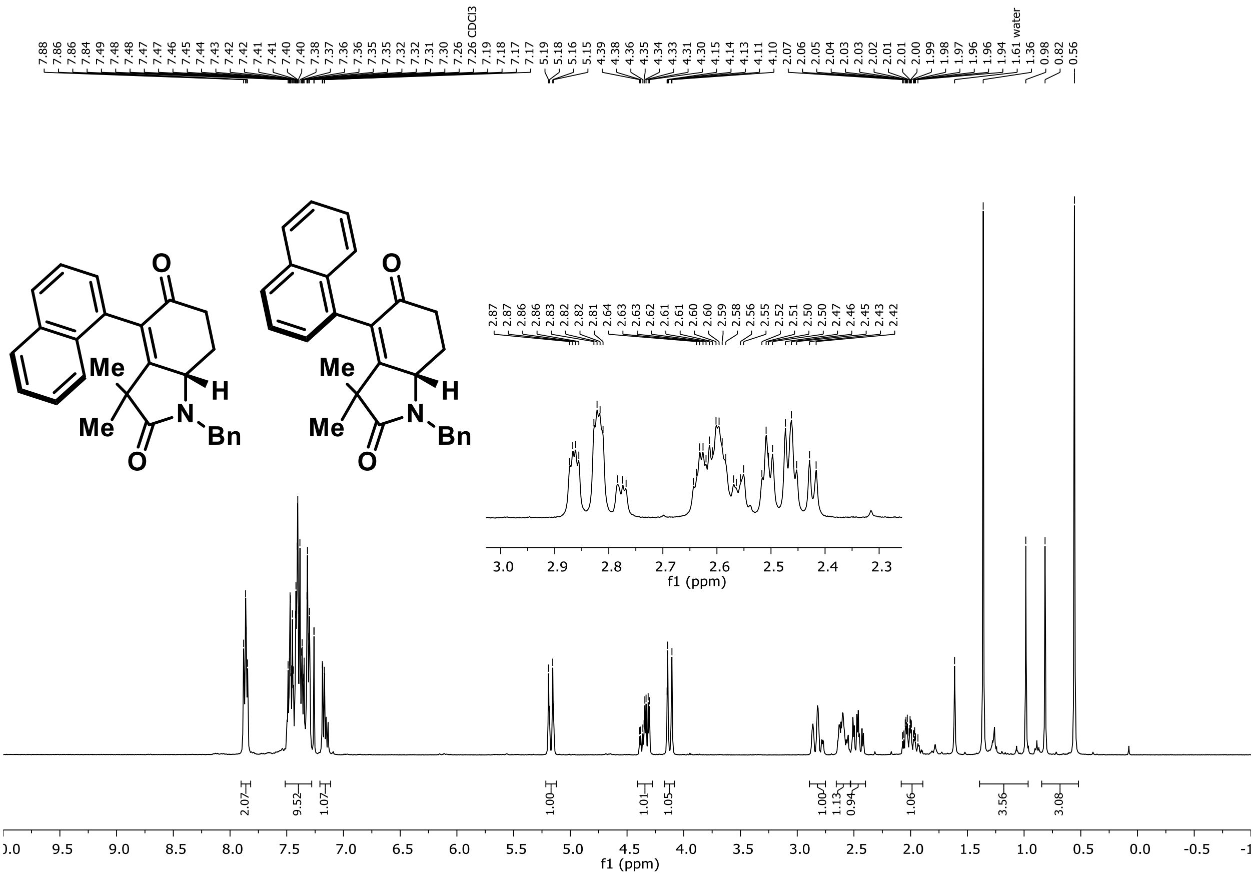

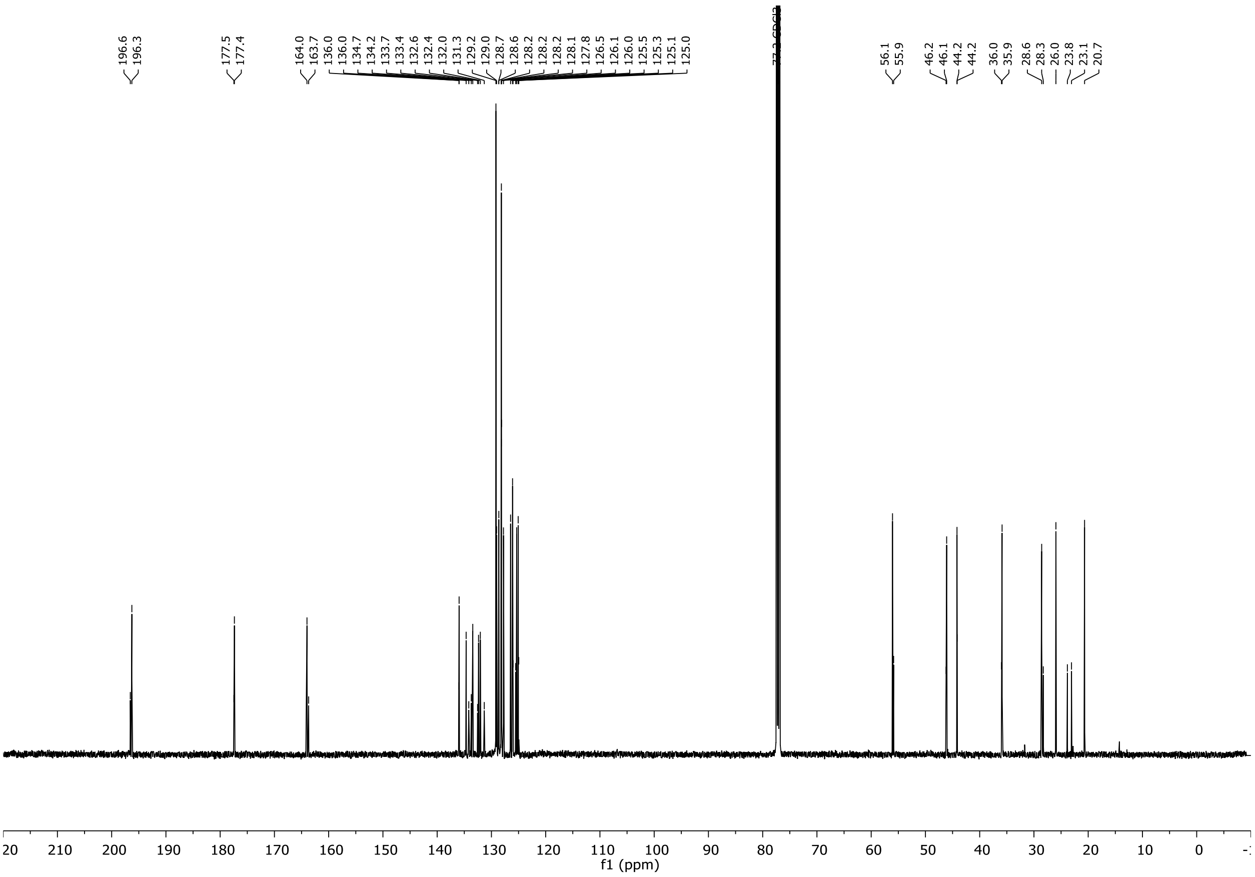

N-Benzyl-*N*-cyclopropyl-2,2-dimethylpent-3-ynamide (3i)

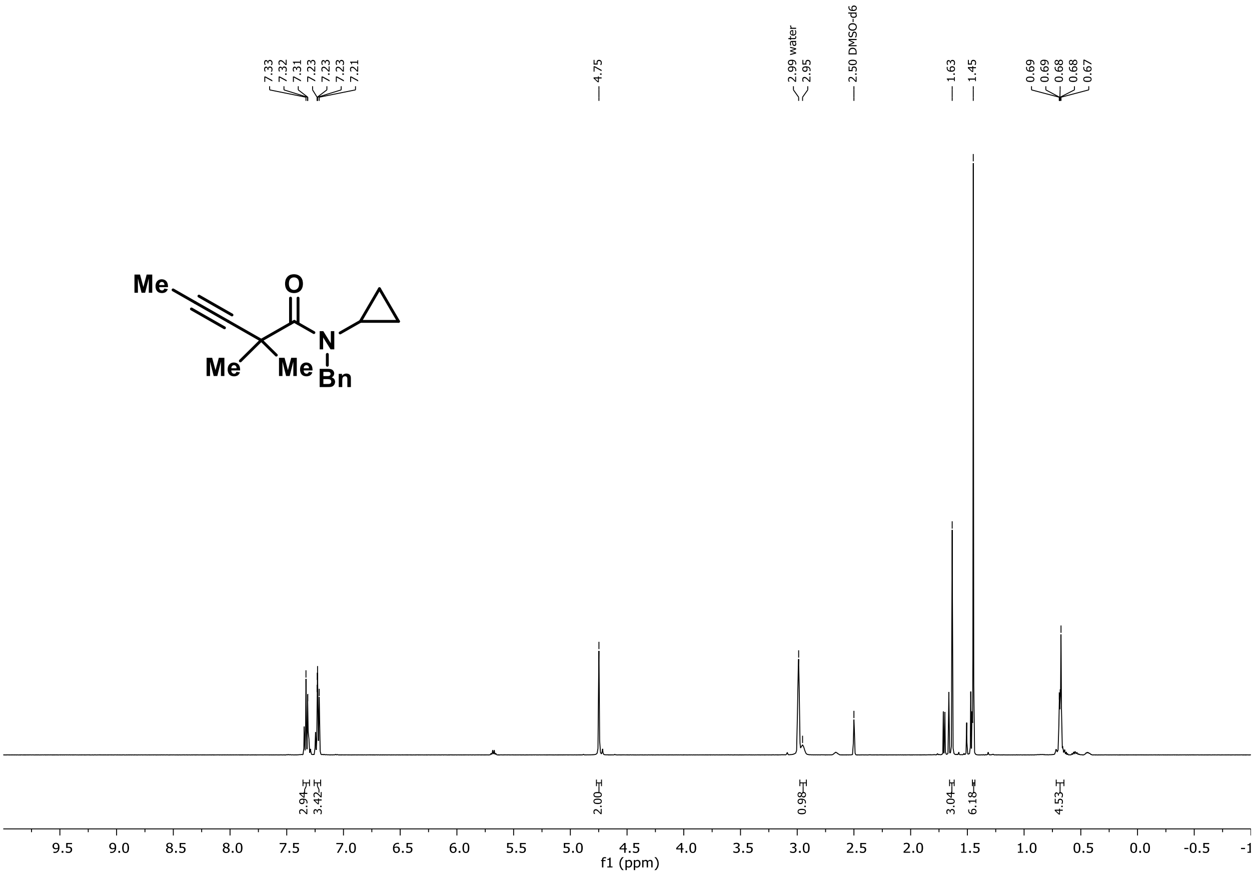

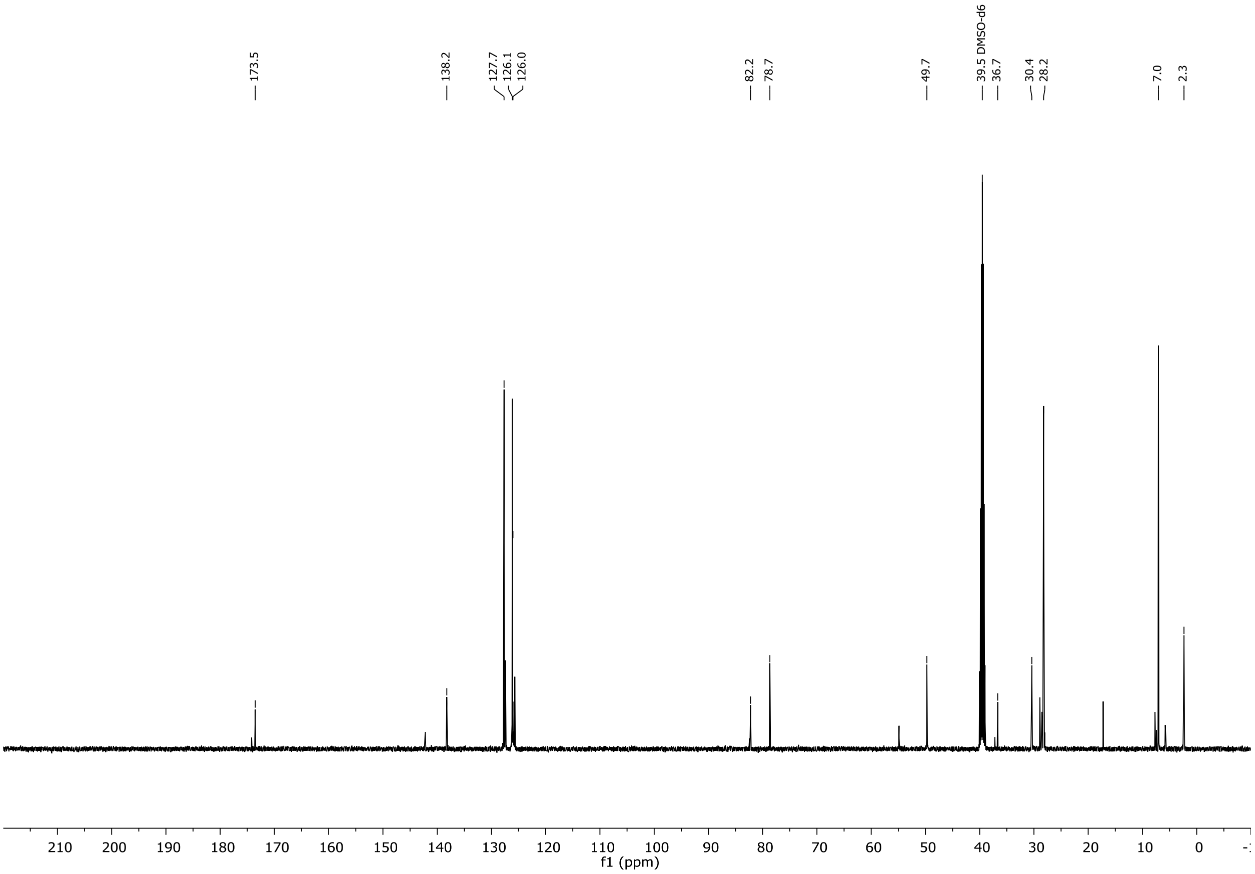

1-Benzyl-3,3,4-trimethyl-1,6,7,7a-tetrahydro-2*H*-indole-2,5(3*H*)-dione (4i)

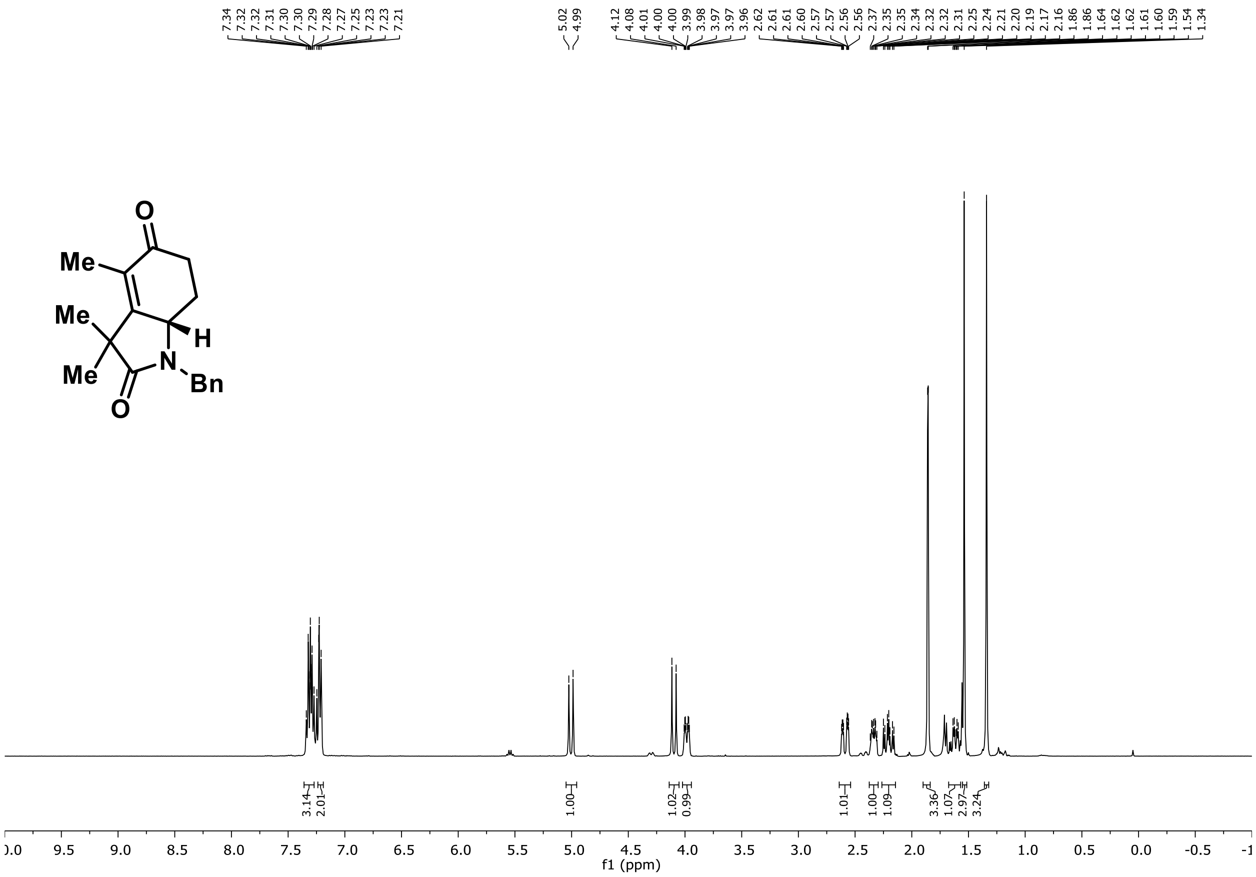

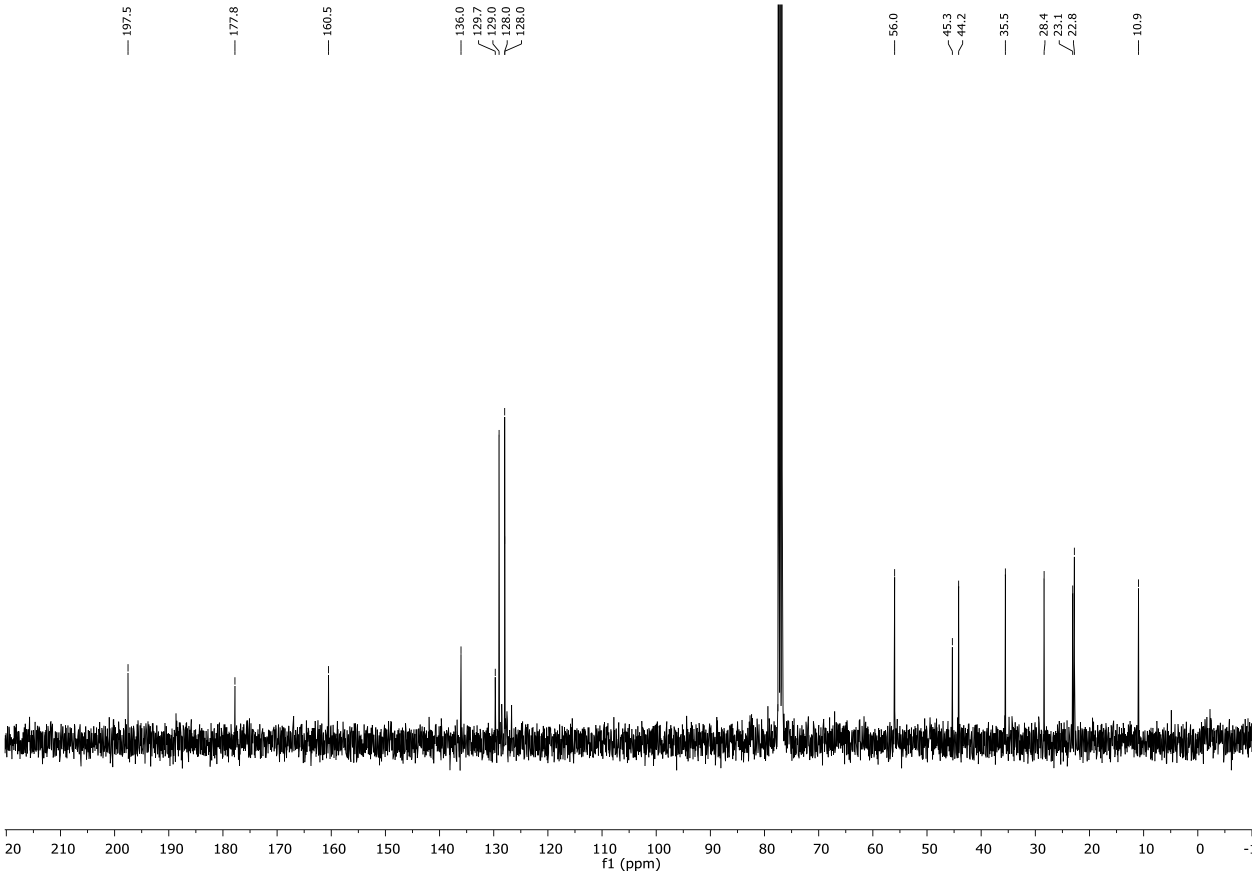

1-Benzyl-3,3-dimethyl-1,6,7,7a-tetrahydro-2*H*-indole-2,5(3*H*)-dione (4j)

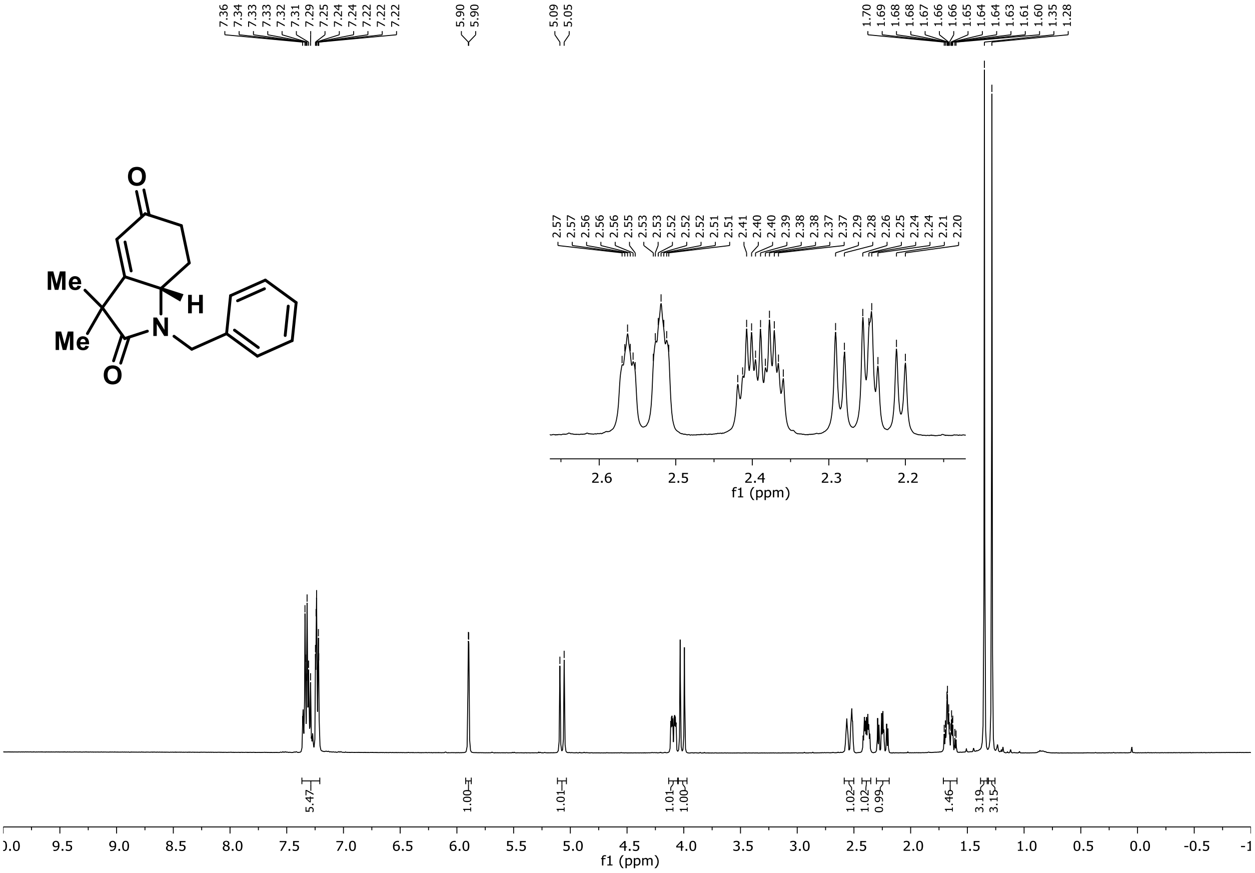

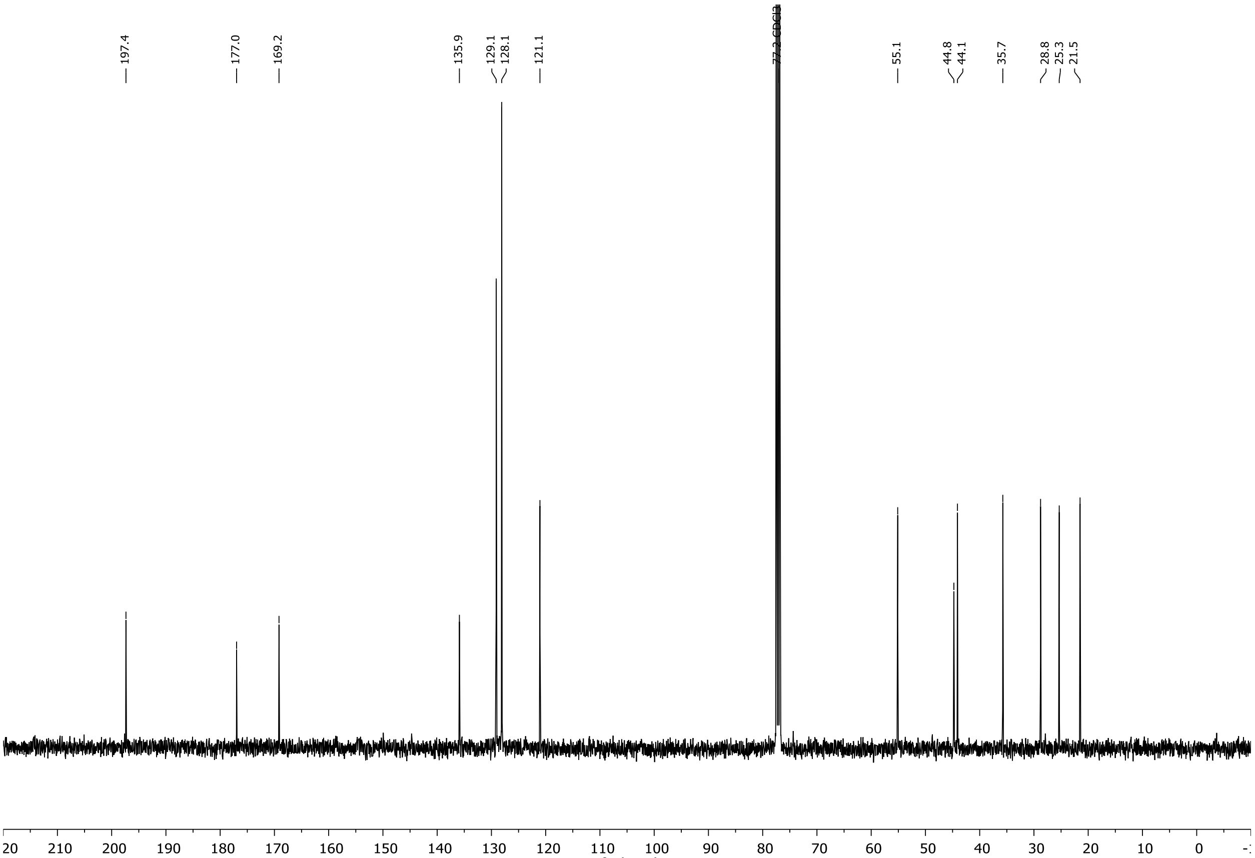

N-Benzyl-*N*-cyclopropyl-1-((trimethylsilyl)ethynyl)cyclopropane-1-carboxamide (3k)

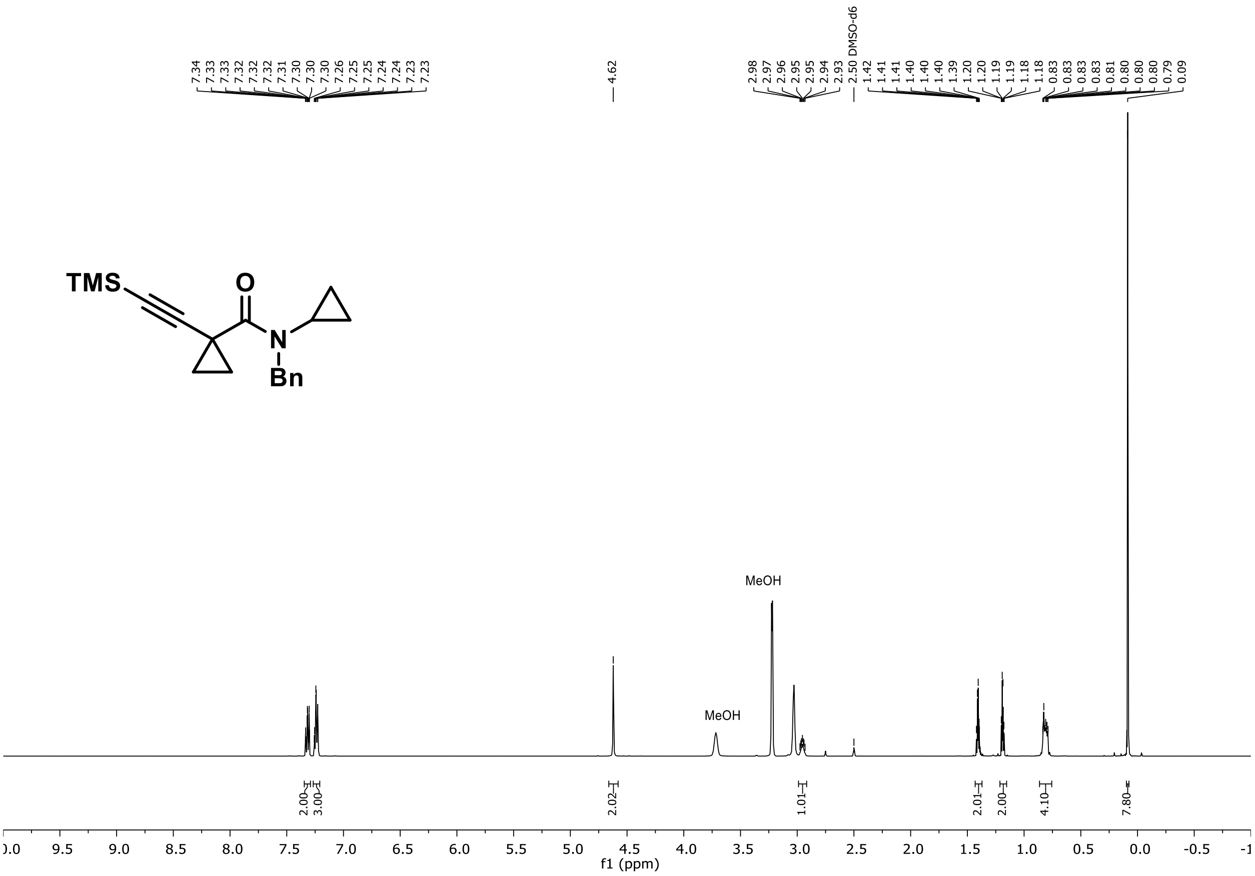

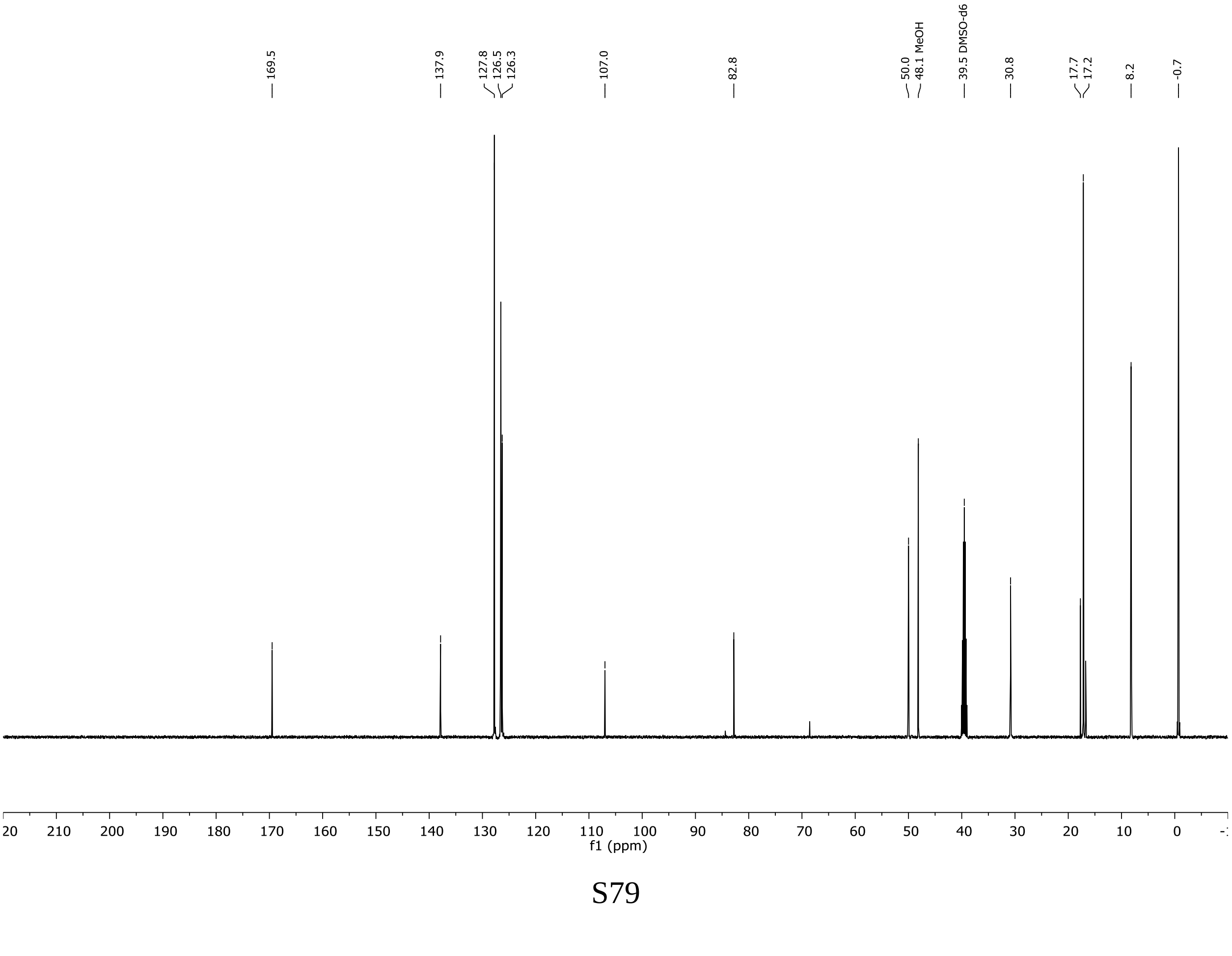

1'-Benzyl-1',6',7',7a'-tetrahydrospiro[cyclopropane-1,3'-indole]-2',5'-dione (4k)

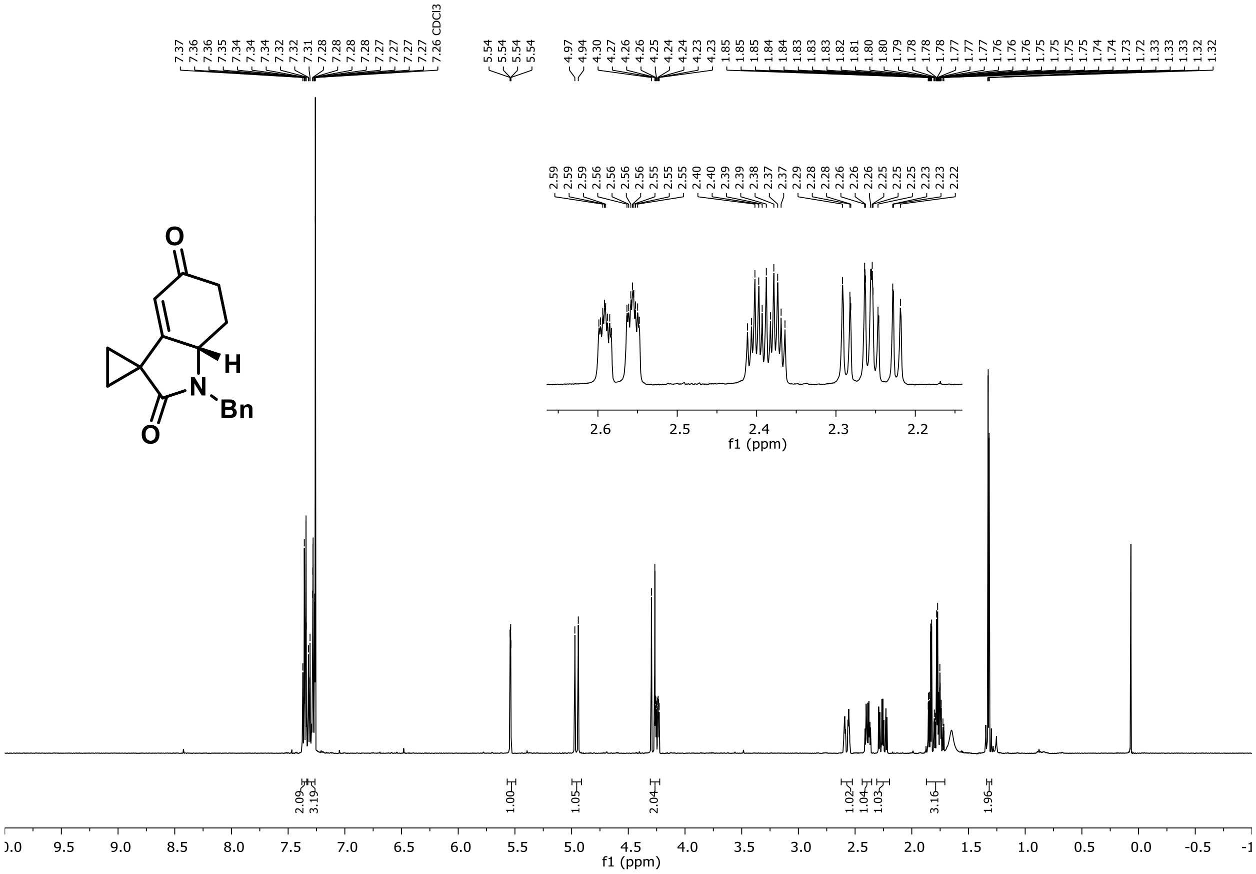

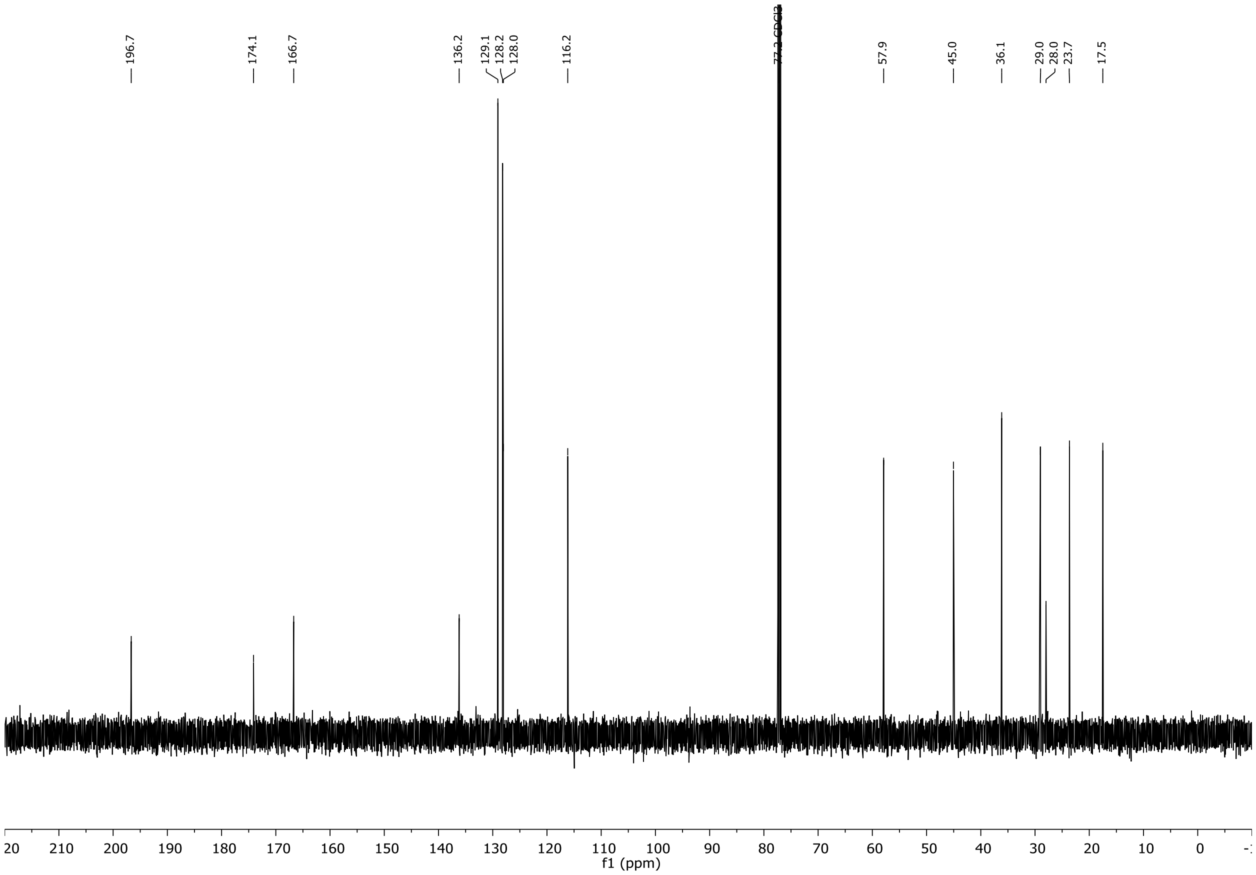

N-Benzyl-*N*-cyclopropyl-2,2-dimethylbut-3-enamide (3l)

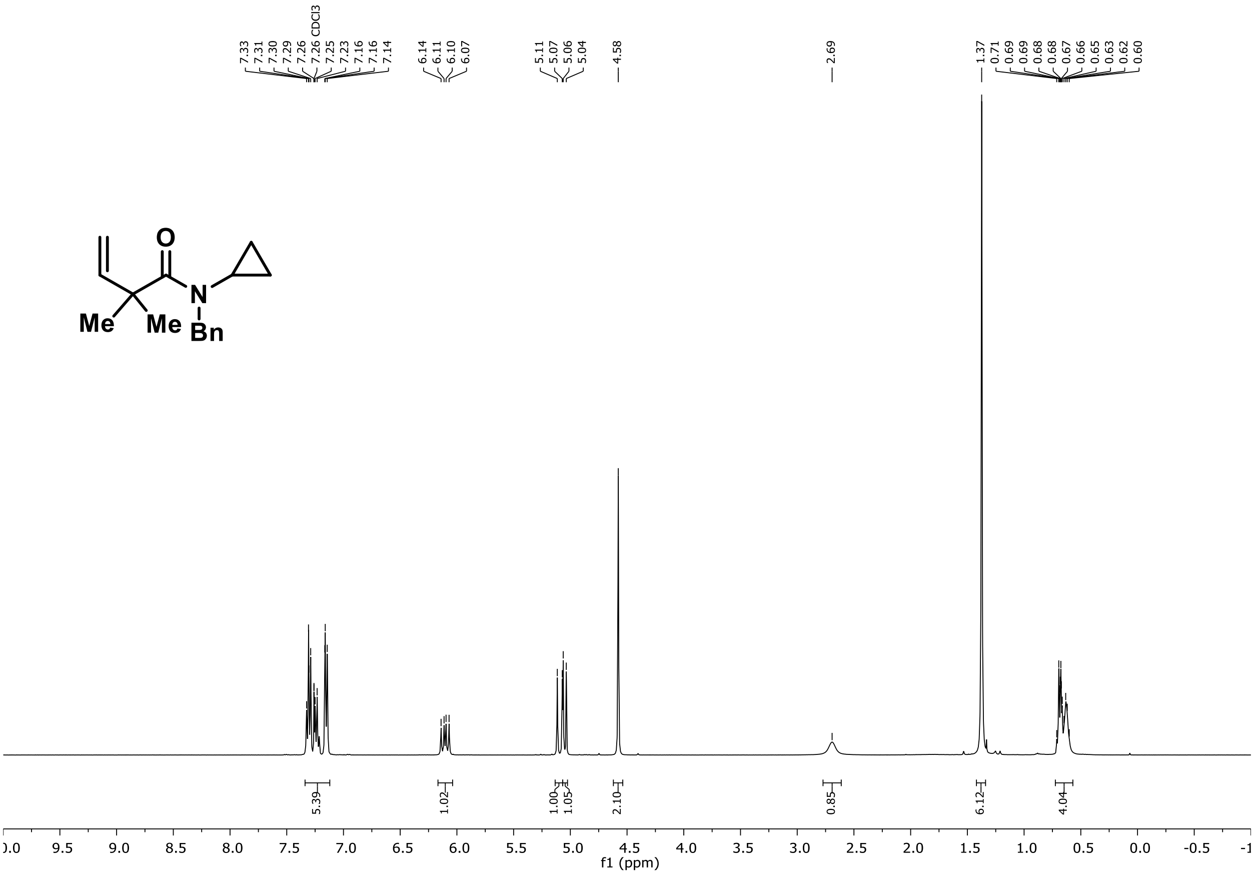

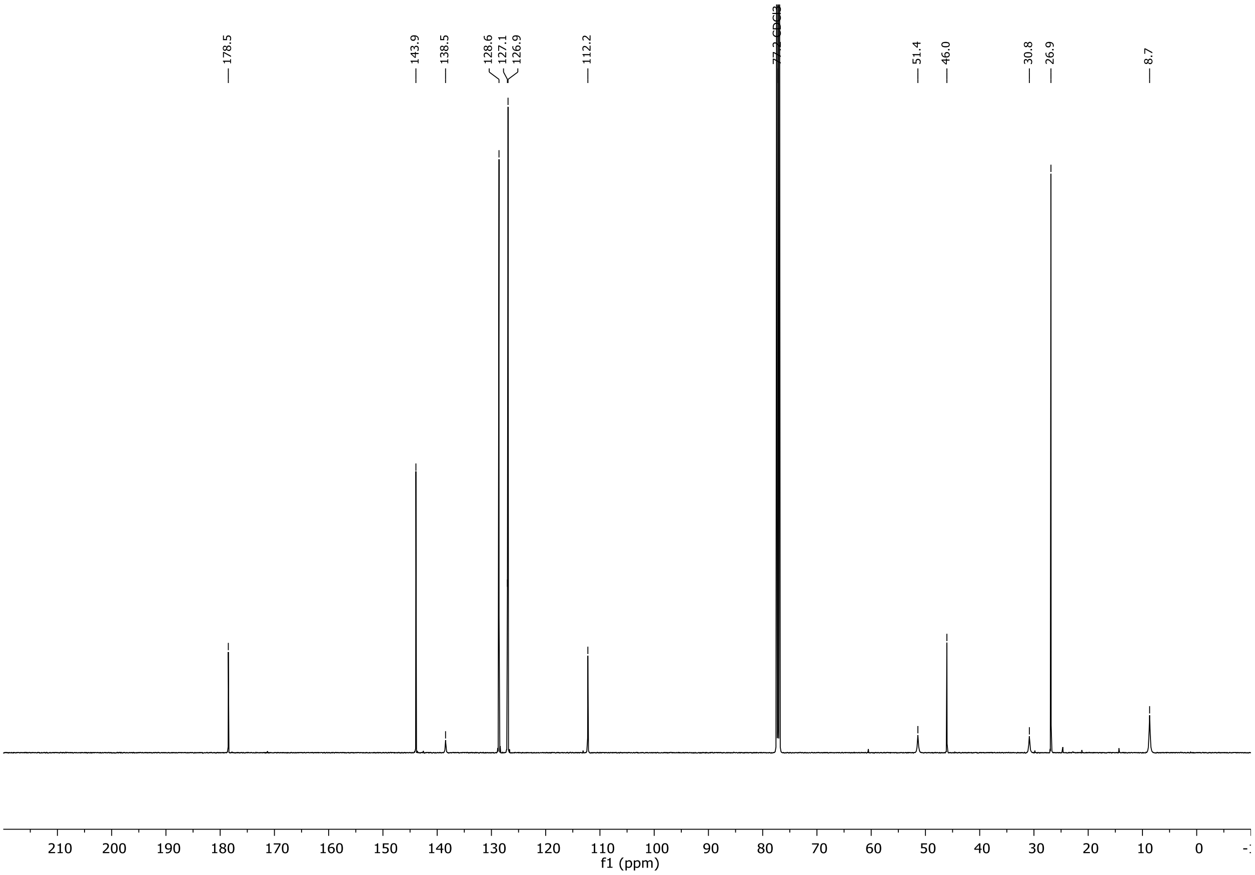

1-Benzyl-3,3-dimethylhexahydro-2*H*-indole-2,5(3*H*)-dione (4l)

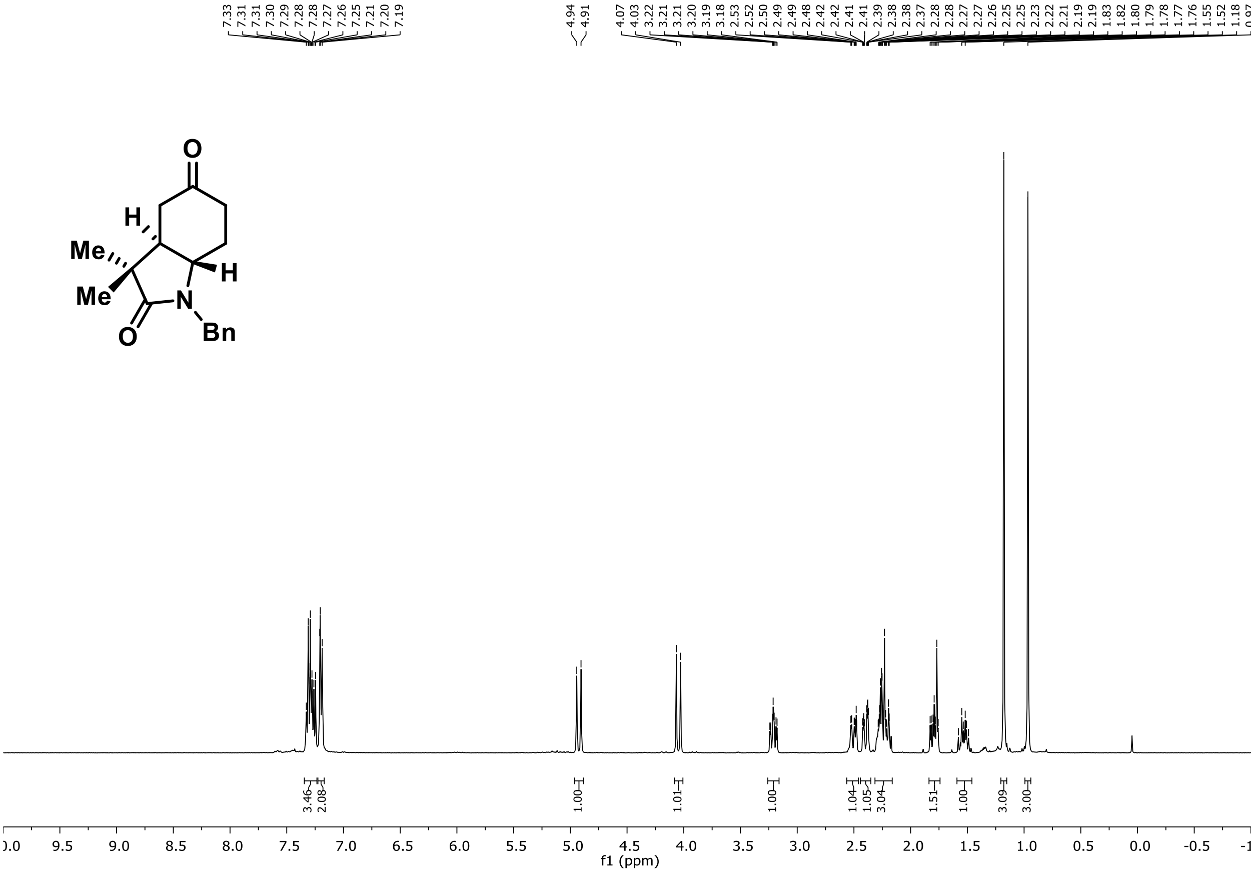

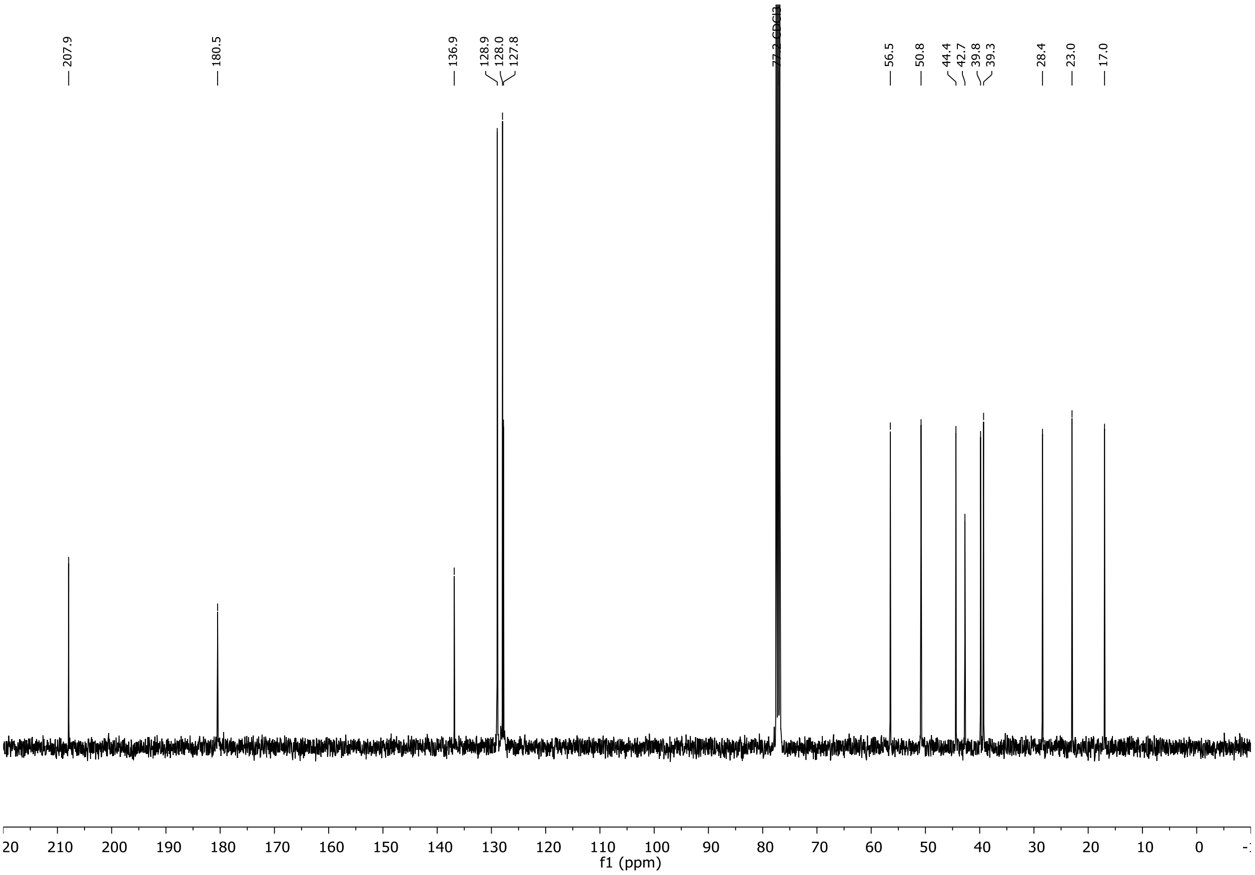

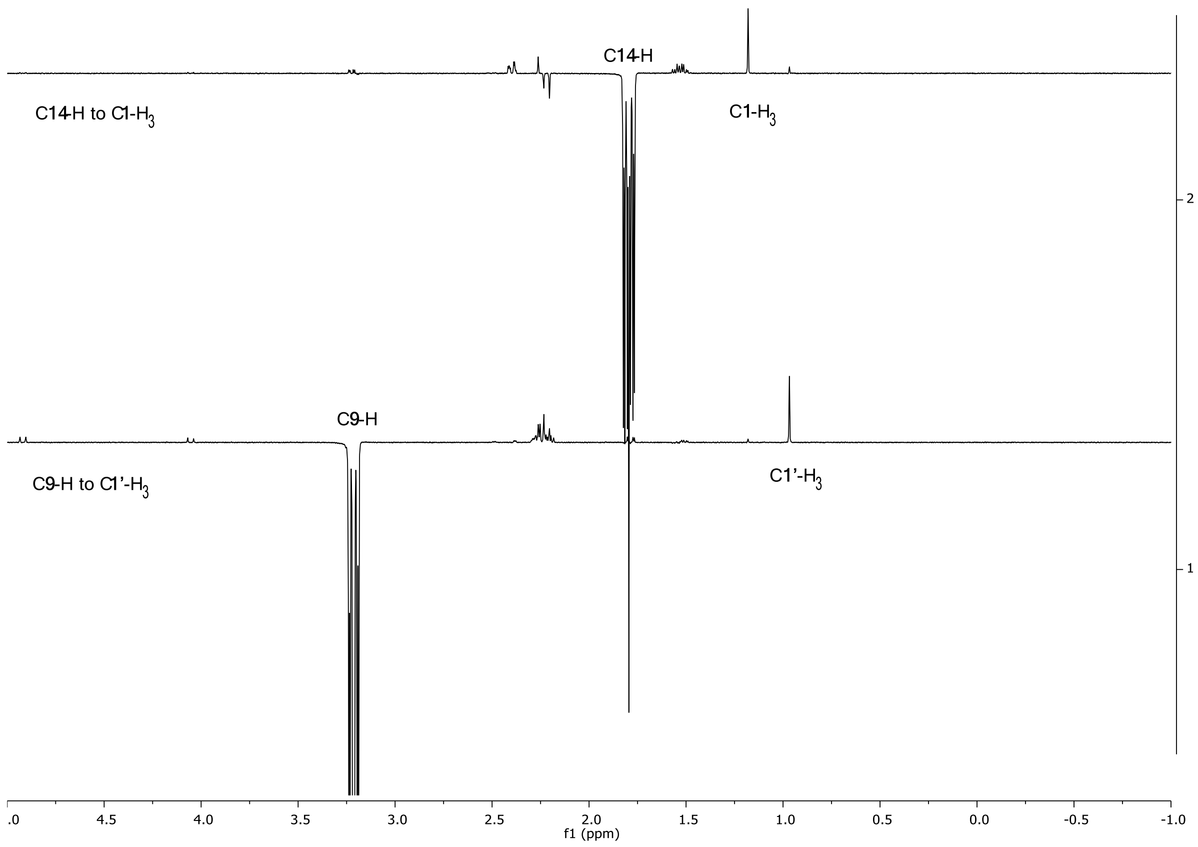
C14-H
C14-H to C1-H_3
C1-H_3
C9-H
C9-H to C1'-H_3
C1'-H_3
f1 (ppm)

N-Benzyl-2,2-dimethyl-*N*-((1*S**,2*S**)-2-methylcyclopropyl)-4-phenylbut-3-ynamide (3m)

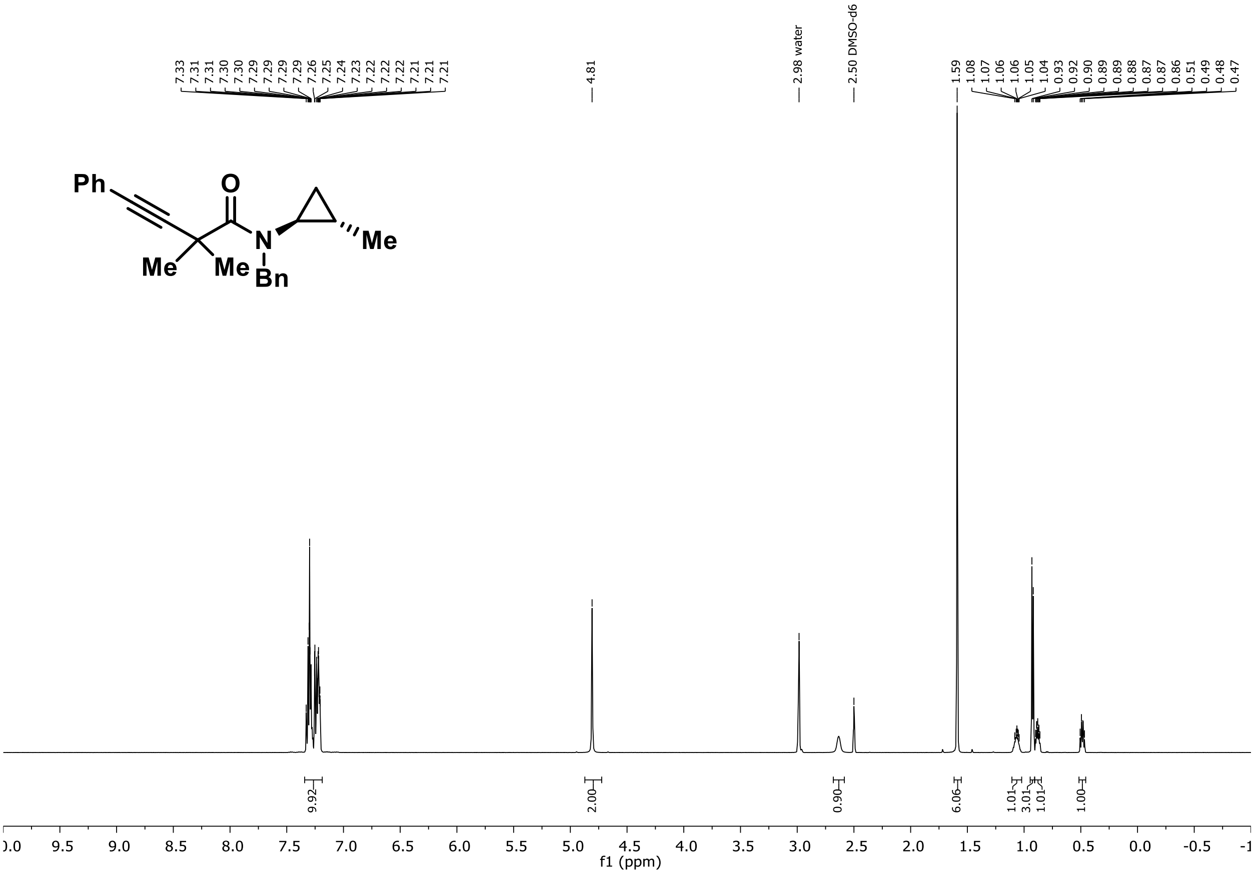

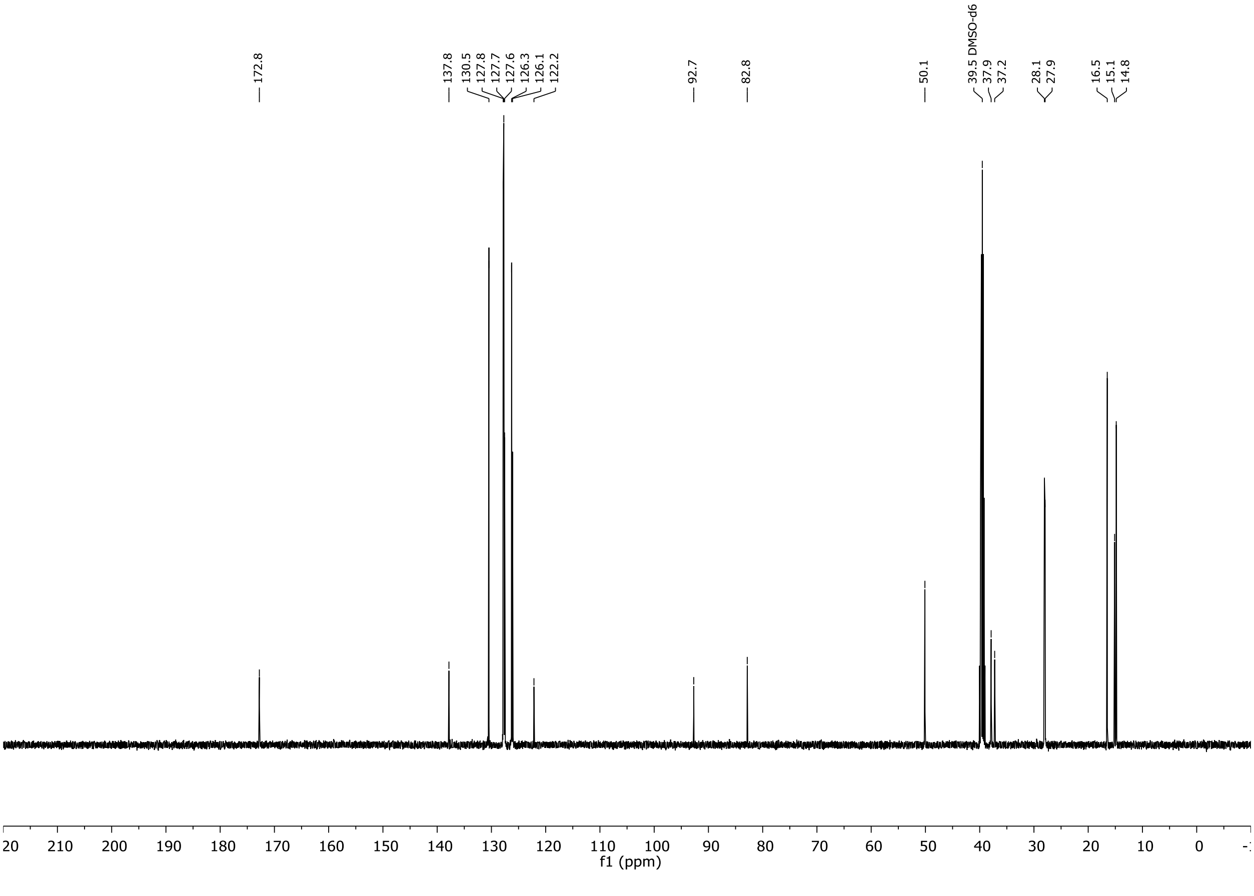

(7*S,7a*R**)-1-Benzyl-3,3,7,7a-tetramethyl-4-phenyl-1,6,7,7a-tetrahydro-2*H*-indole-2,5(3*H*)-dione (4m)**

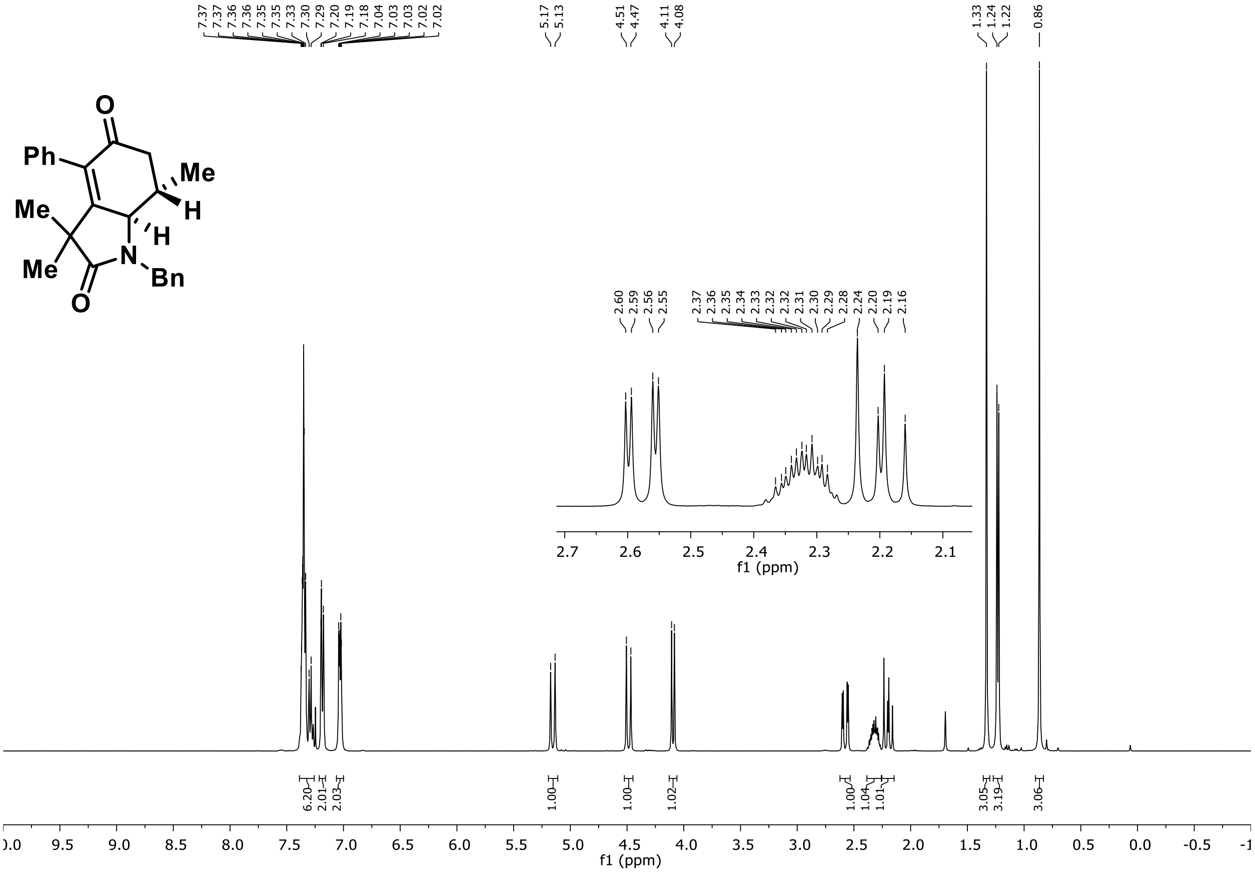

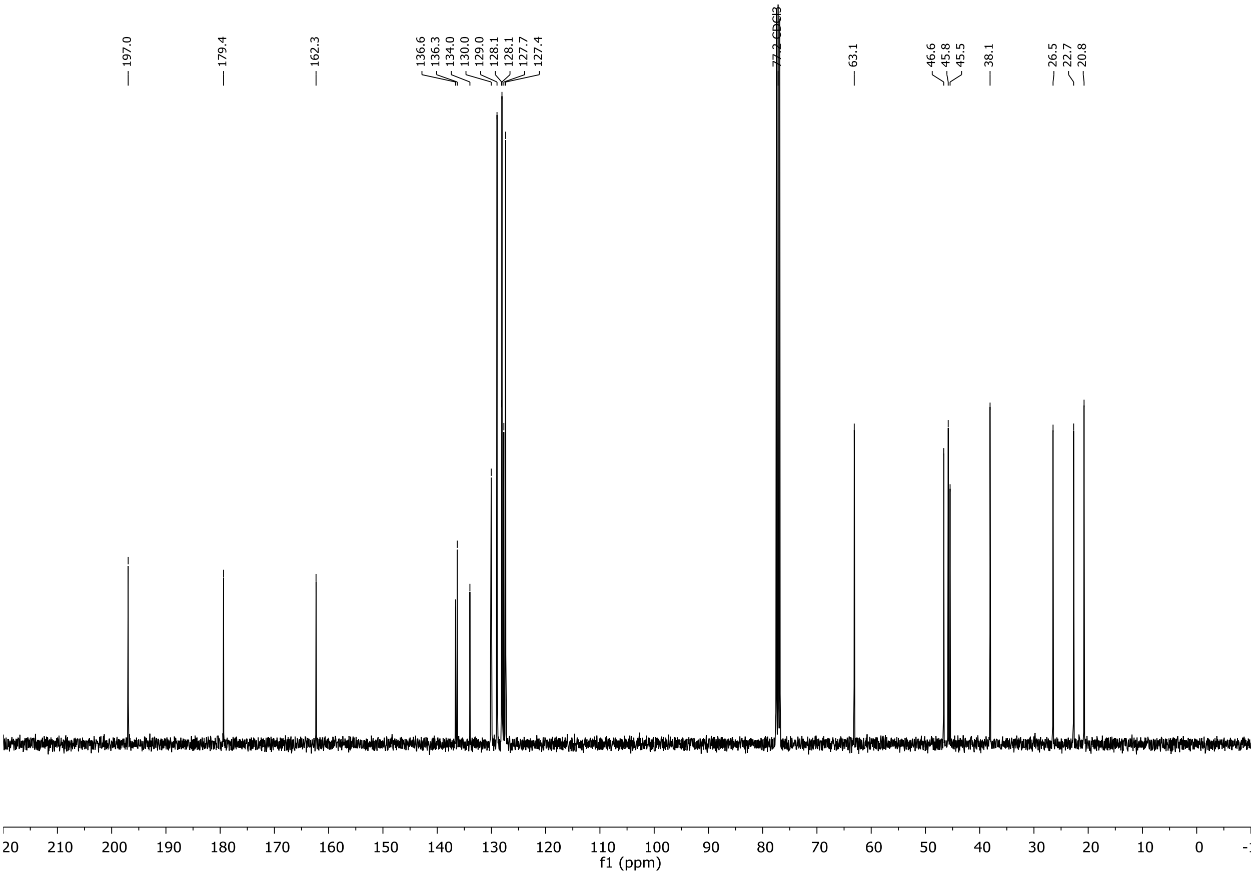

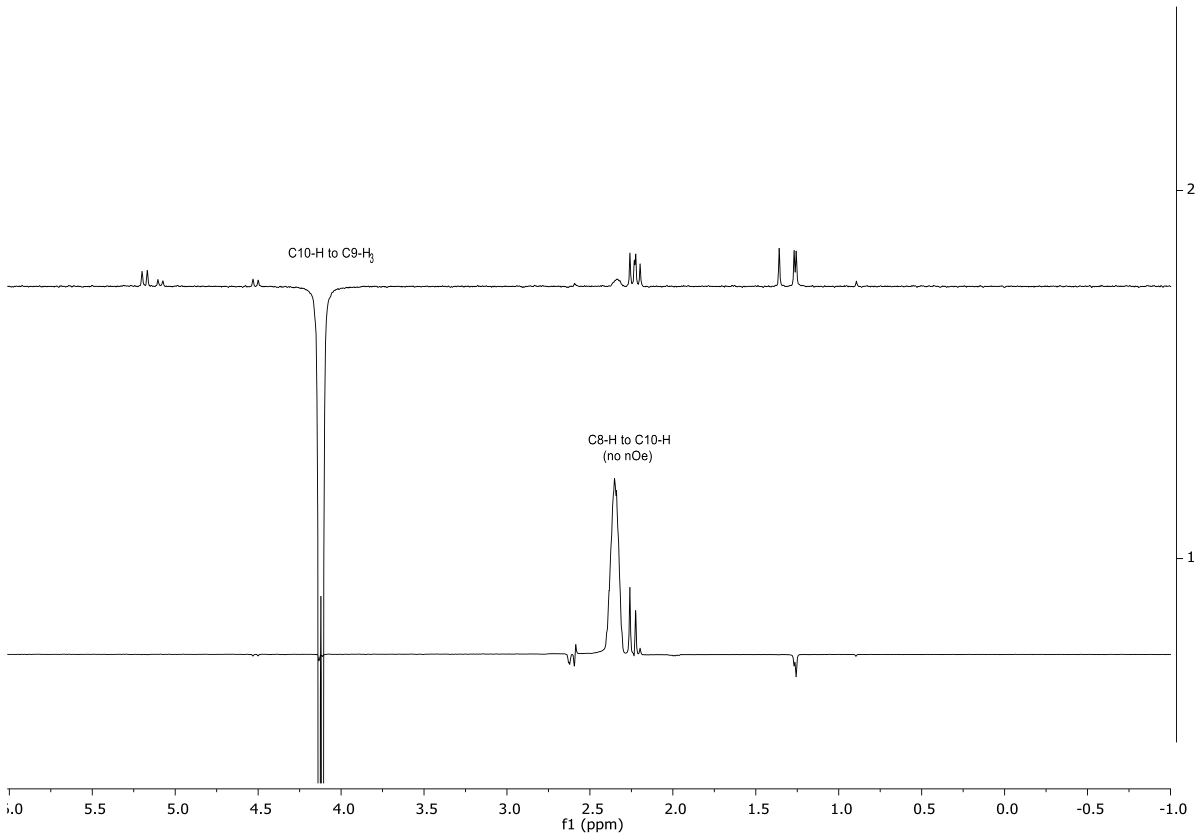
C10-H to C9-H3
C8-H to C10-H
(no nOe)
f1 (ppm)

N-Benzyl-*N*-((1*S**,2*S**)-2-benzylcyclopropyl)-2,2-dimethyl-4-phenylbut-3-ynamide (3n)

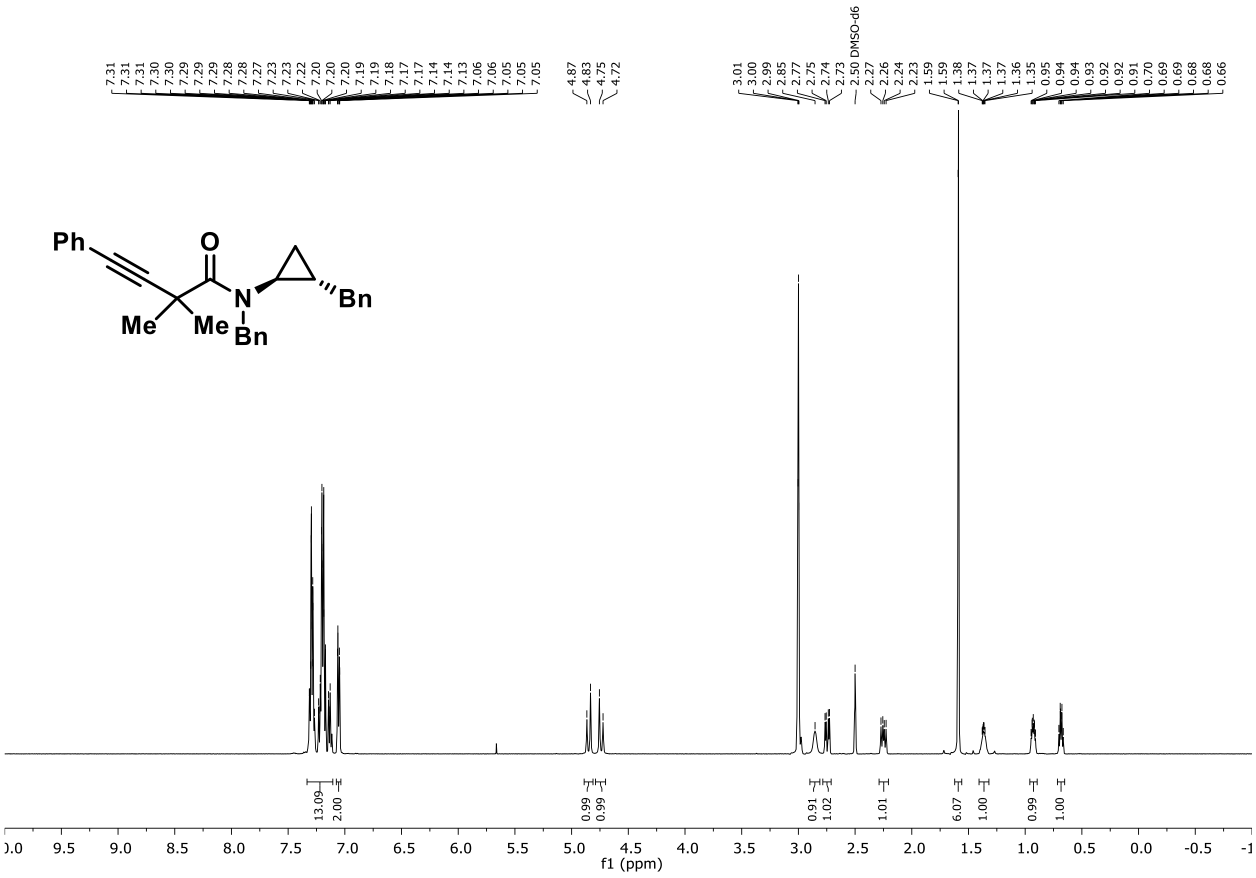

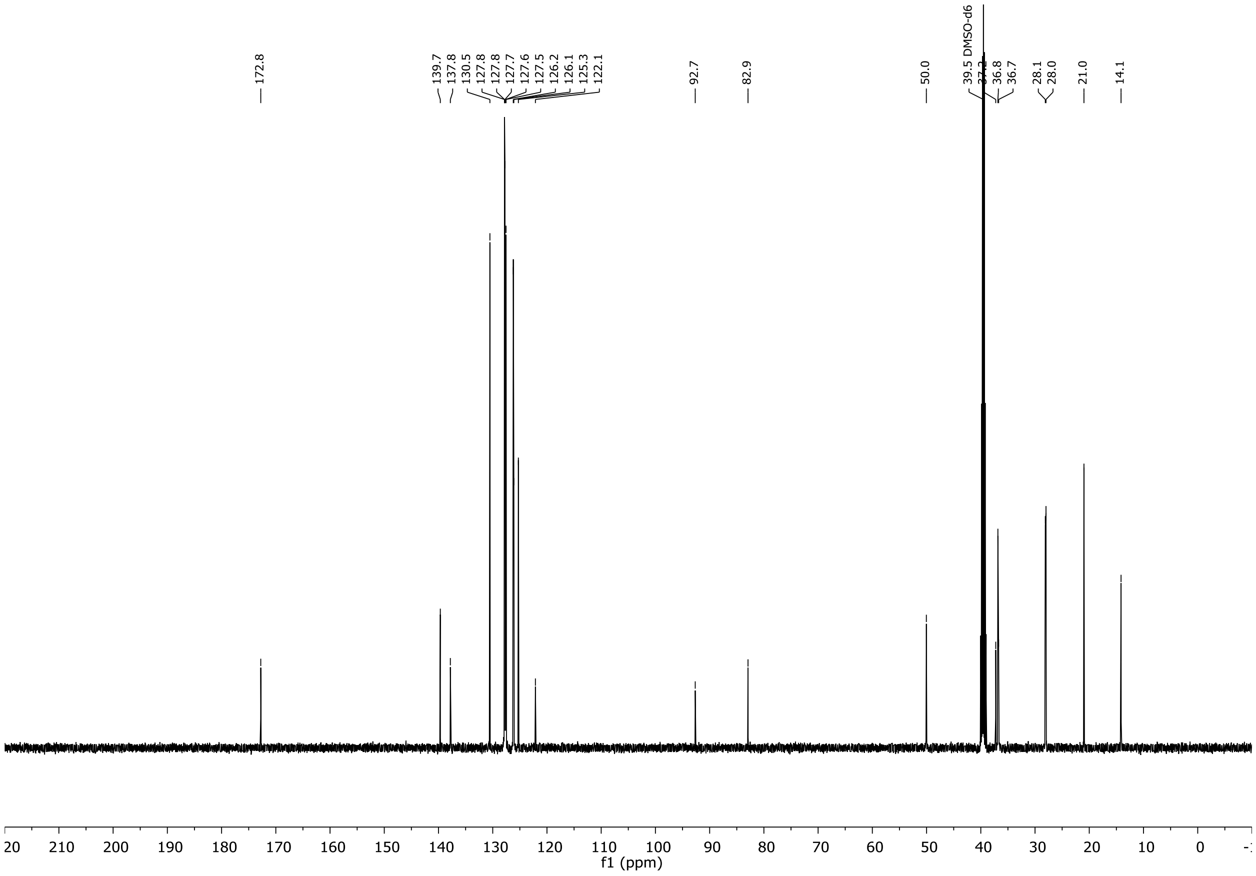

(7*R,7aS*)-1,7-Dibenzyl-3,3-dimethyl-4-phenyl-1,6,7,7a-tetrahydro-2*H*-indole-2,5(3*H*)-dione (4n)**

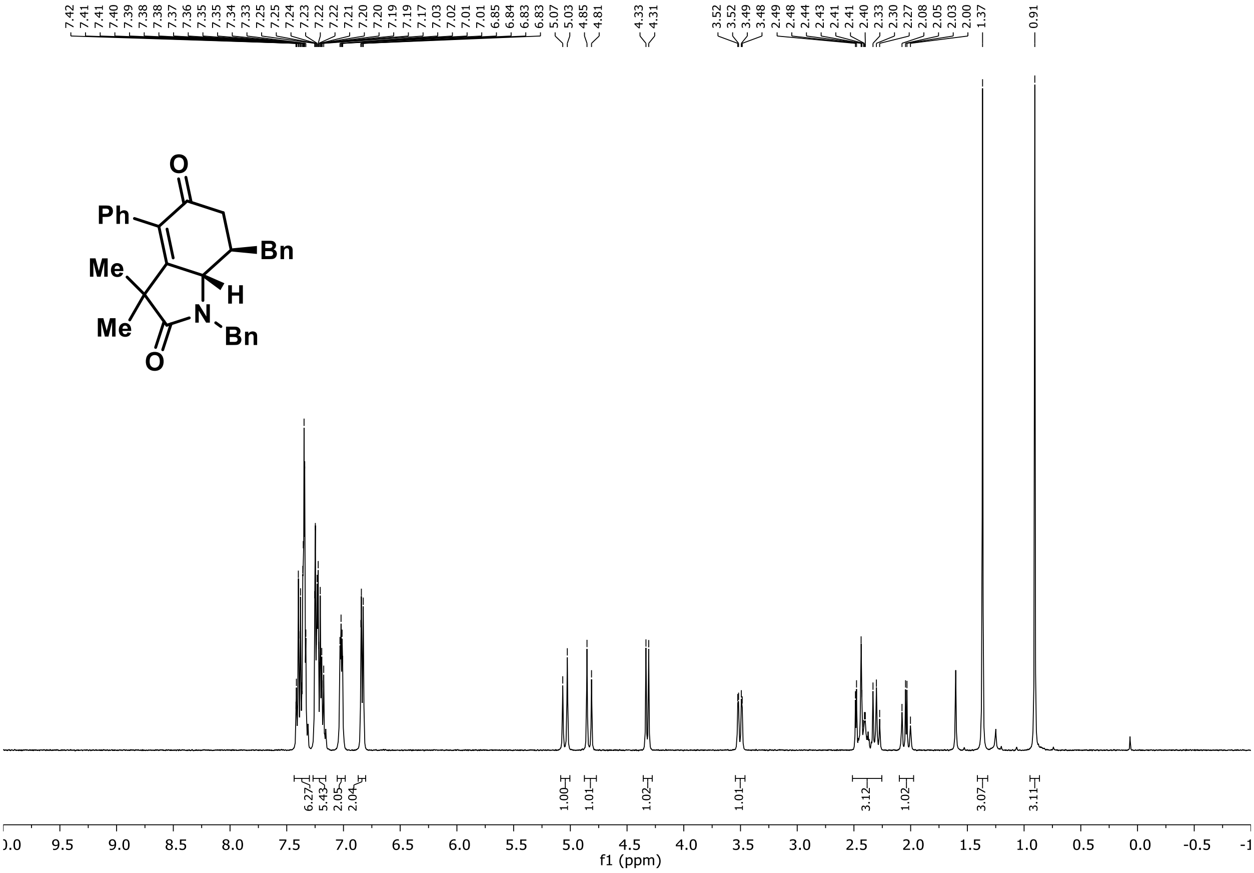

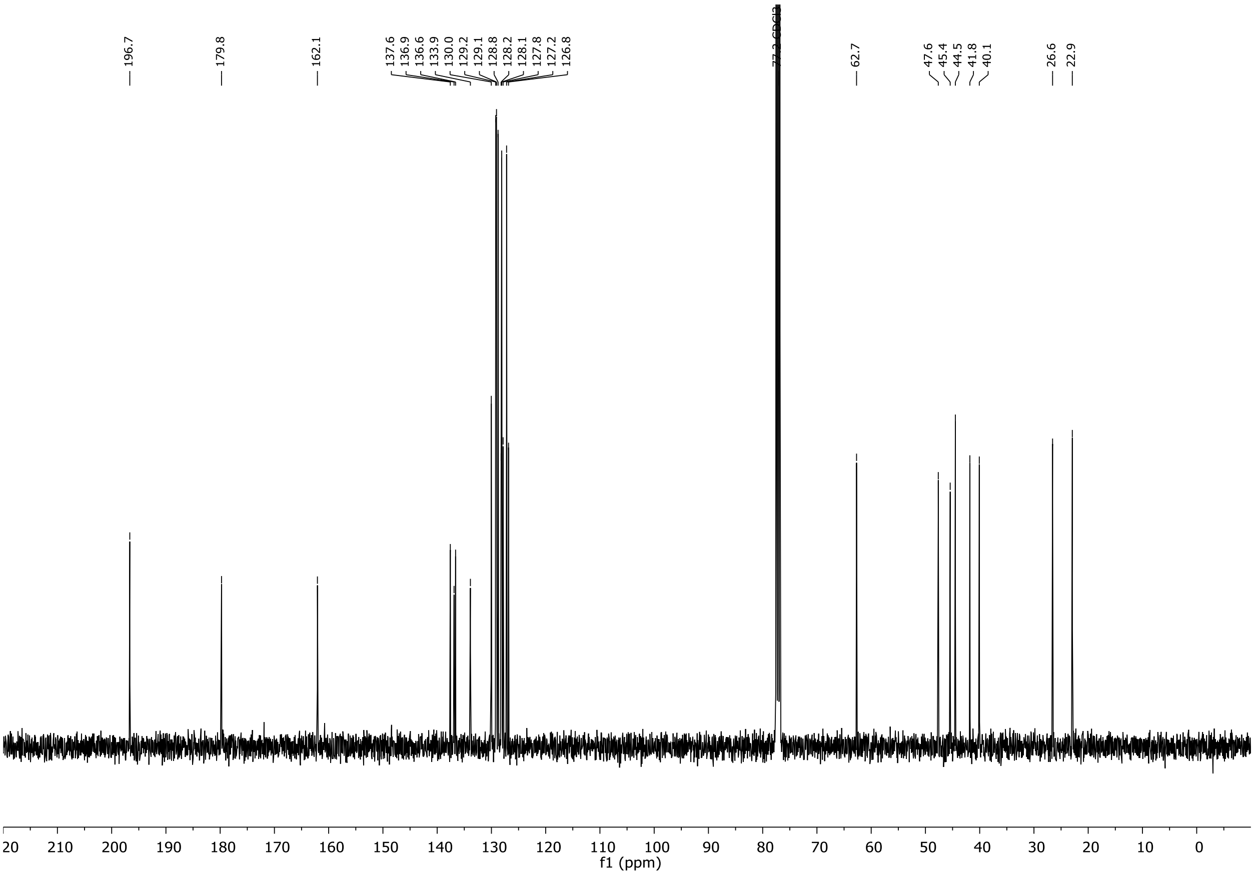

***N*-Benzyl-2,2-dimethyl-*N*-((1*S**,2*S**)-2-methylcyclopropyl)but-3-enamide (3o)**

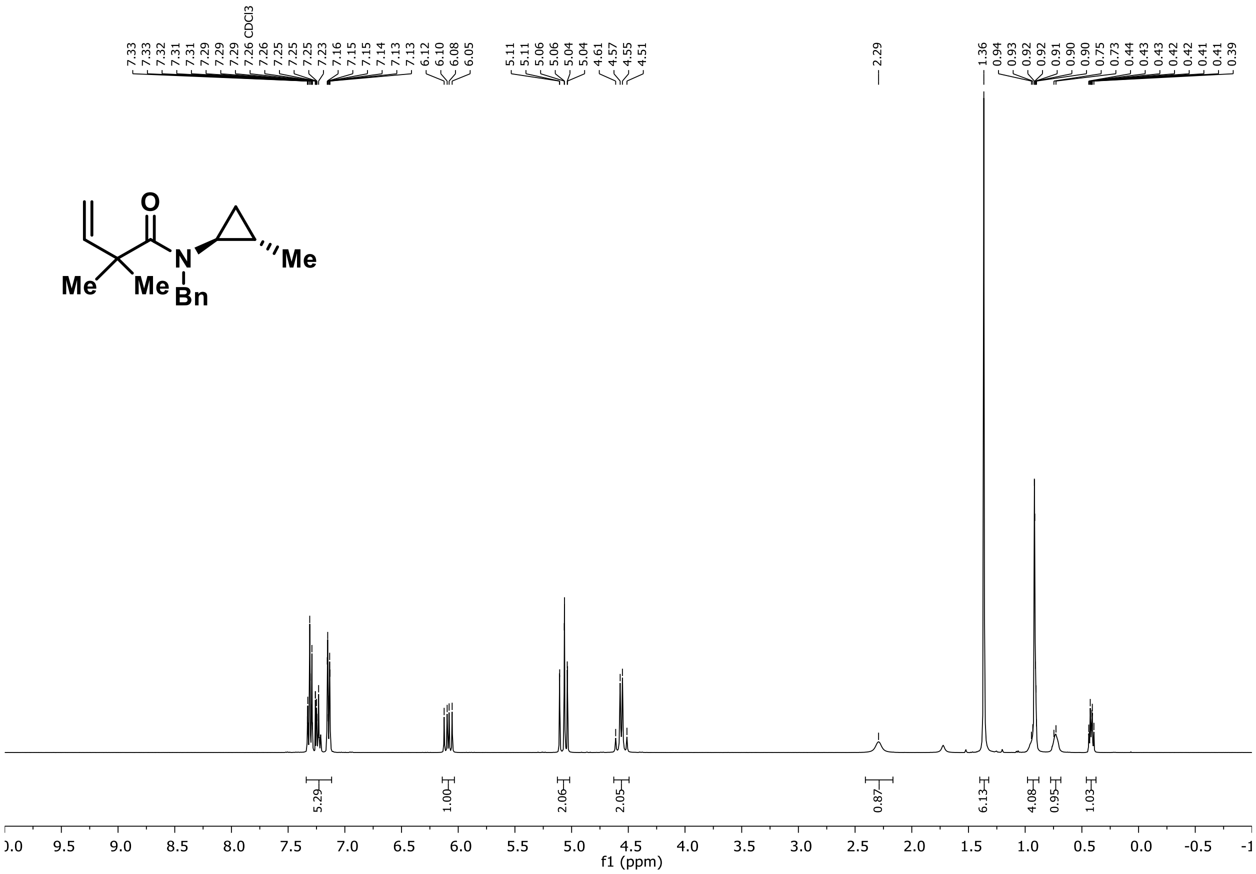

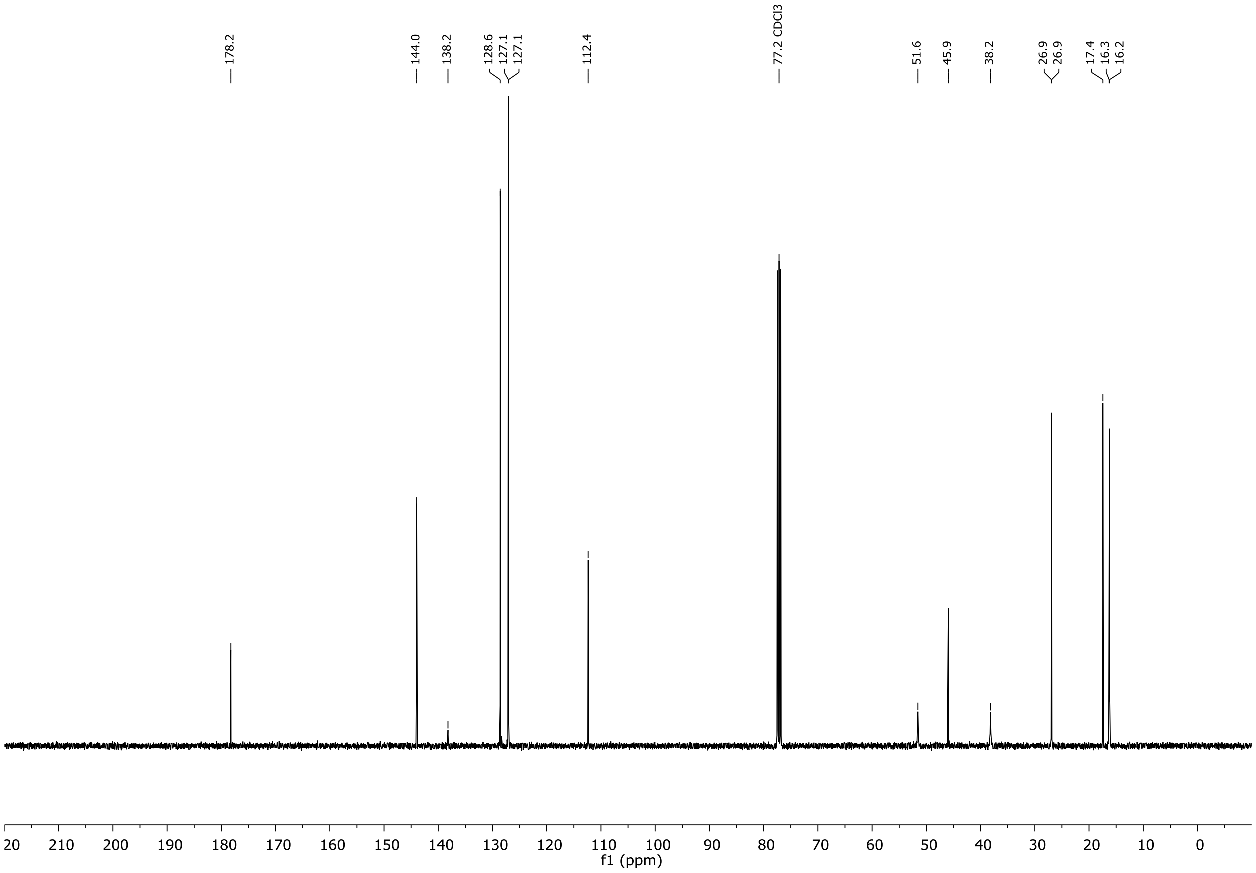

(3a*R**,7*R**,7a*S**)-1-Benzyl-3,3,7-trimethylhexahydro-2*H*-indole-2,5(3*H*)-dione (4o)

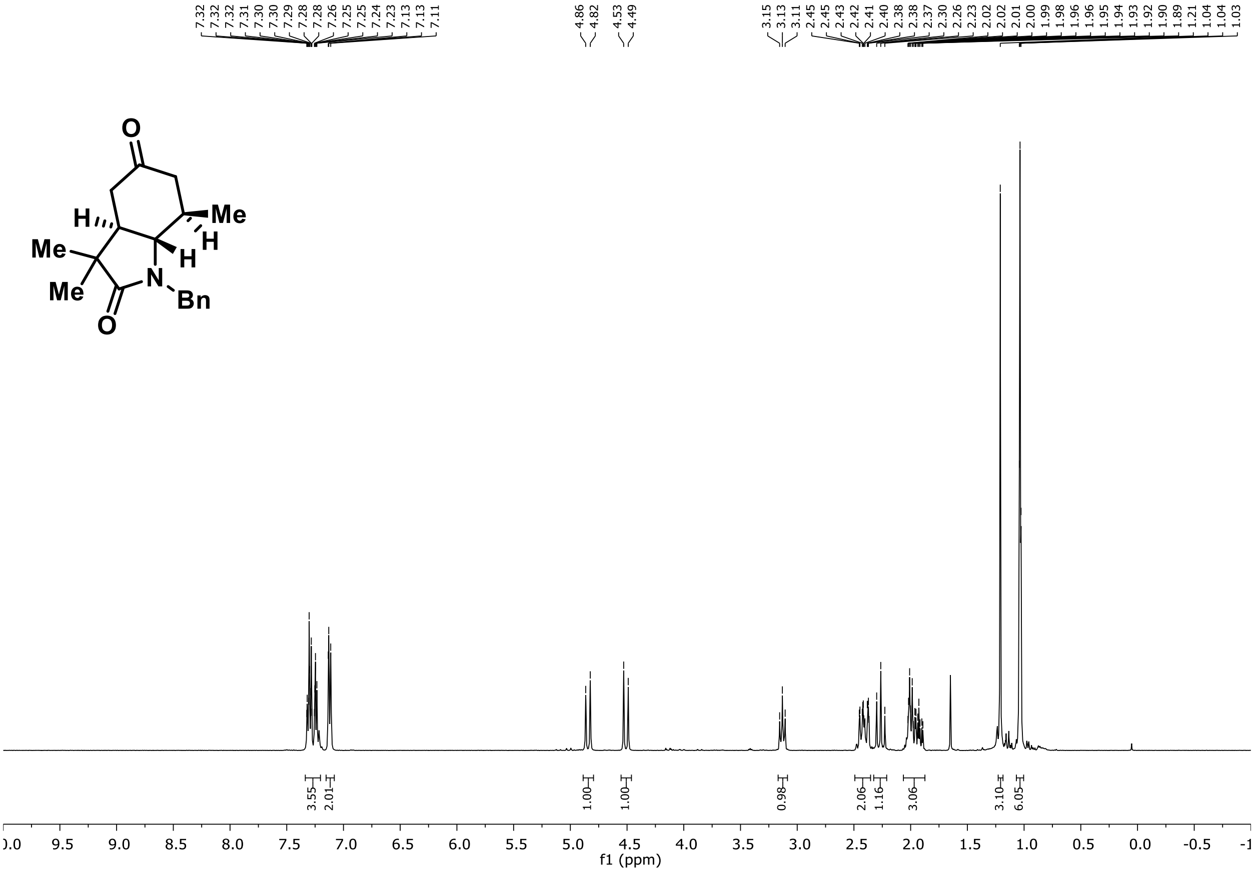

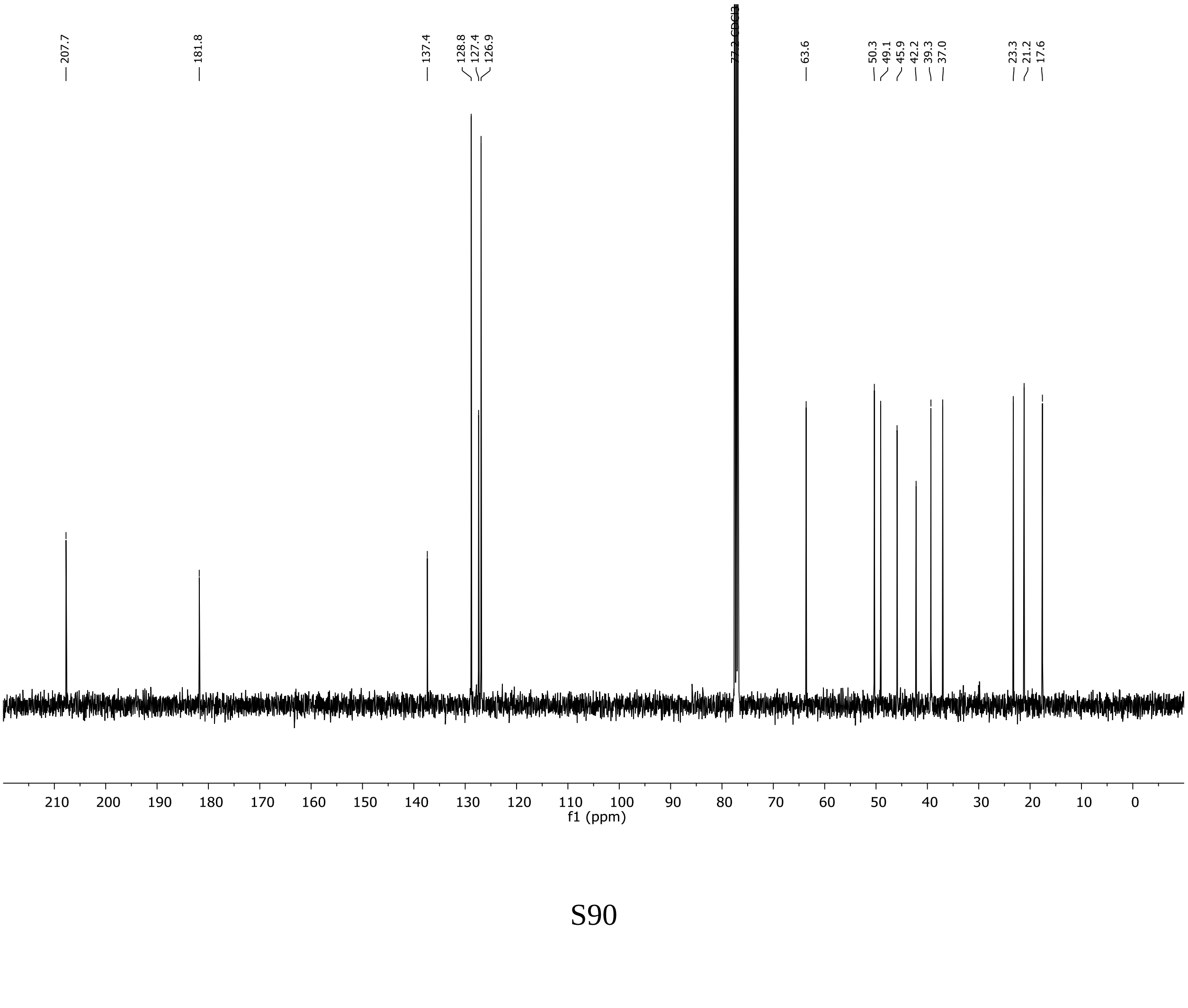

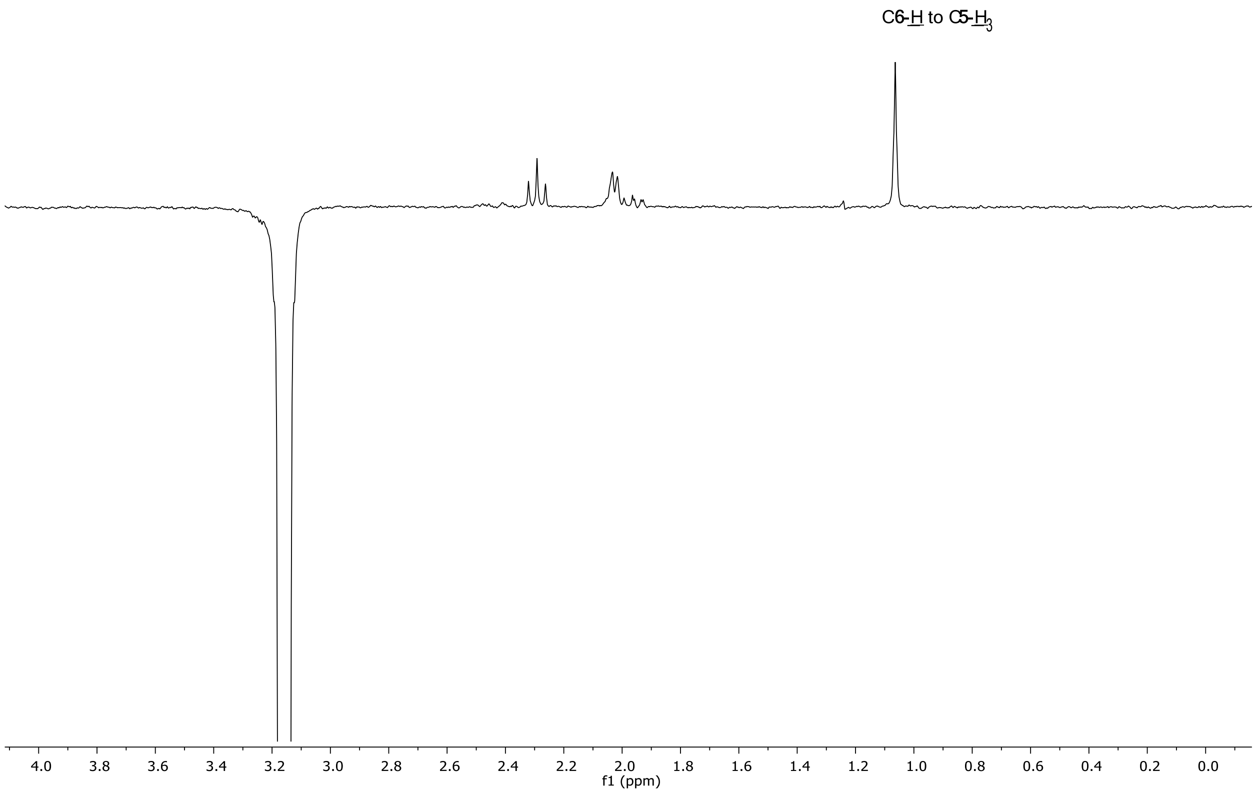
C6-H to C5-H3
4.0
3.8
3.6
3.4
3.2
3.0
2.8
2.6
2.4
2.2
2.0
1.8
1.6
1.4
1.2
1.0
0.8
0.6
0.4
0.2
0.0
f1 (ppm)

N-Benzyl-2,2-dimethyl-*N*-((1*S**,2*R**)-2-methylcyclopropyl)-4-phenylbut-3-ynamide (3p)

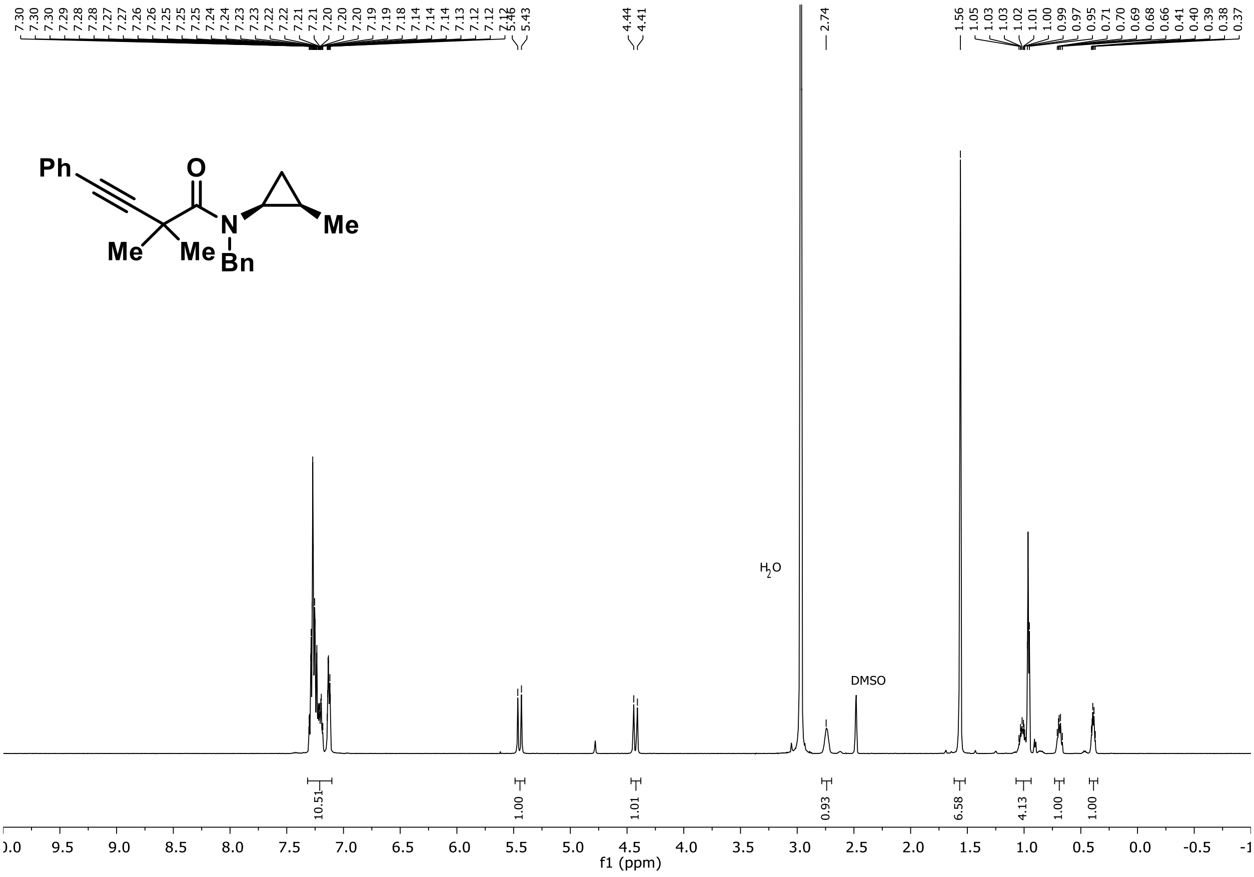

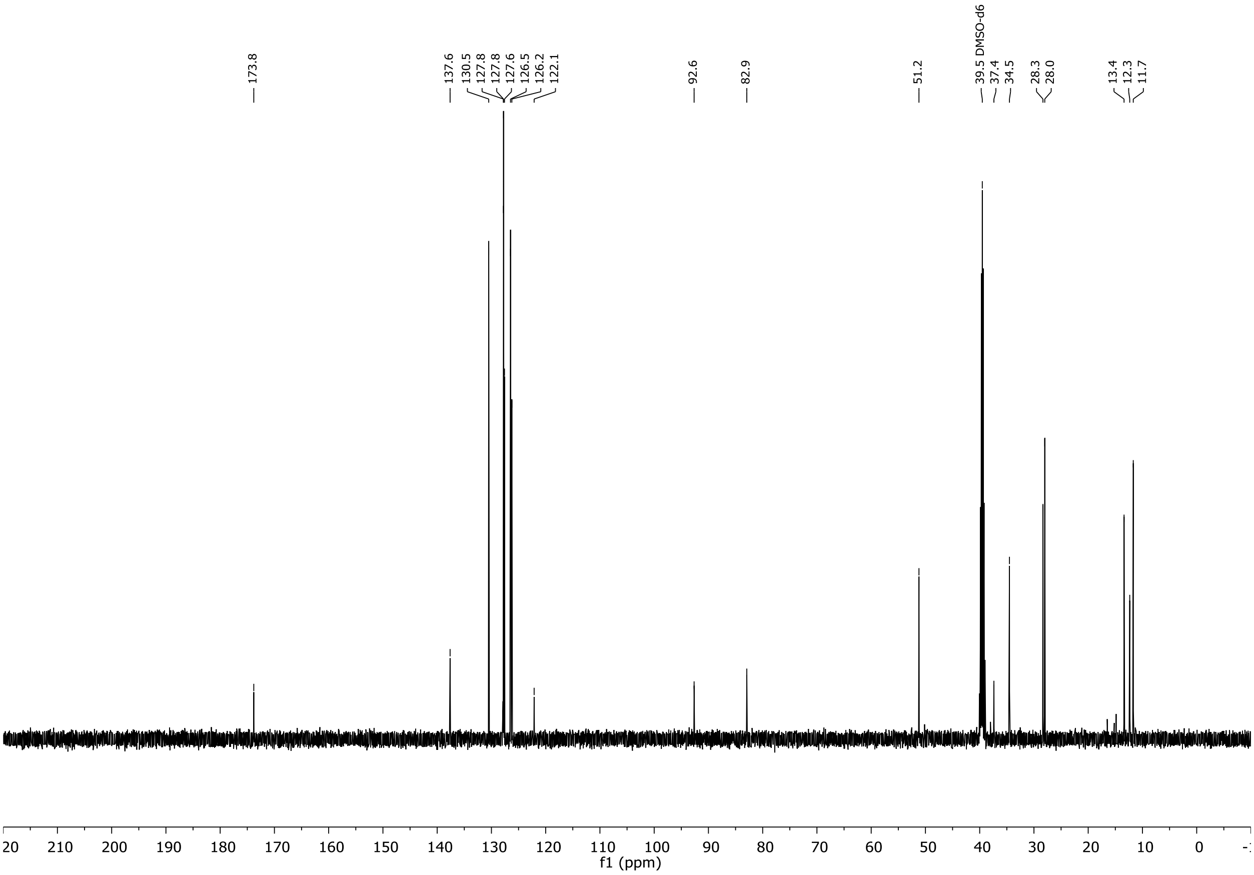

(6*S,7a*R**)-1-Benzyl-3,3,6-trimethyl-4-phenyl-1,6,7,7a-tetrahydro-2*H*-indole-2,5(3*H*)-dione (4p)**

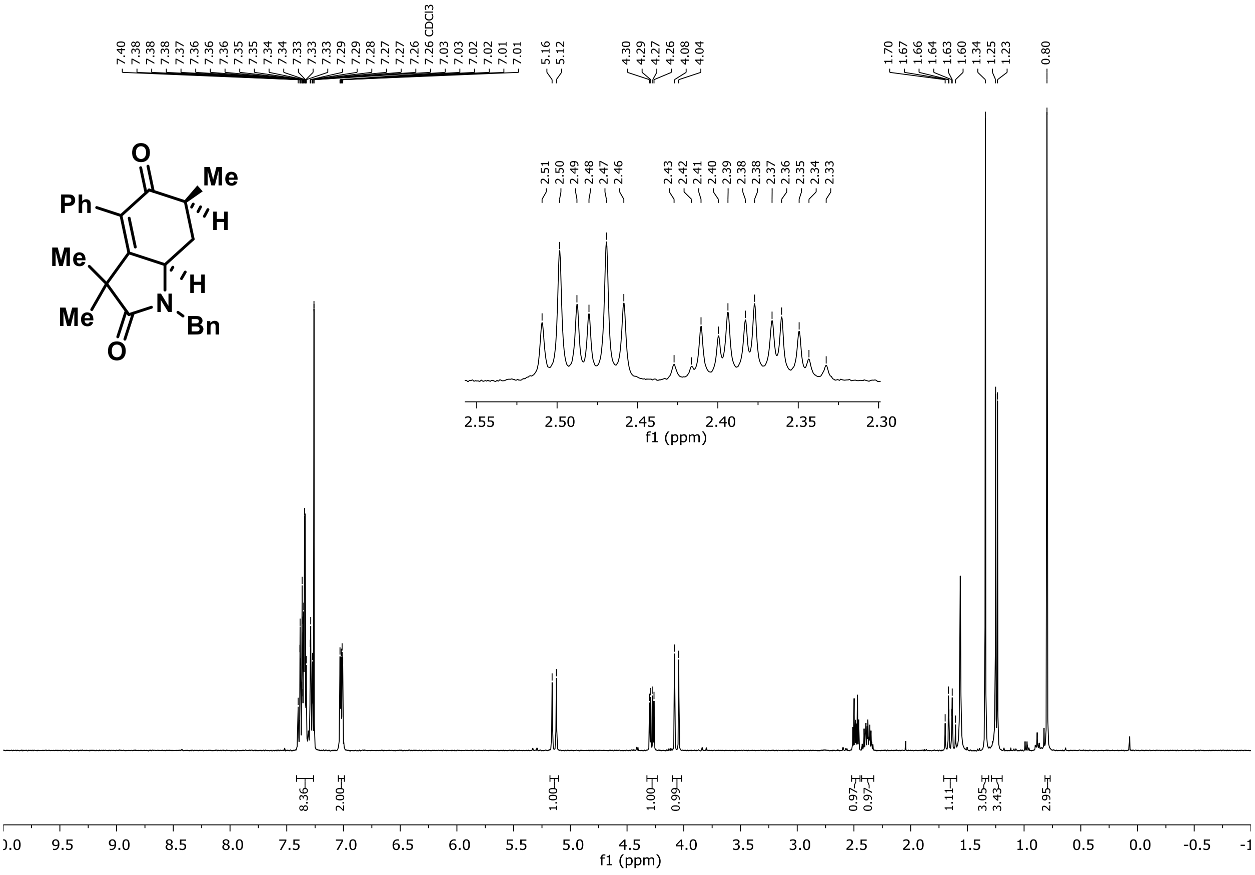

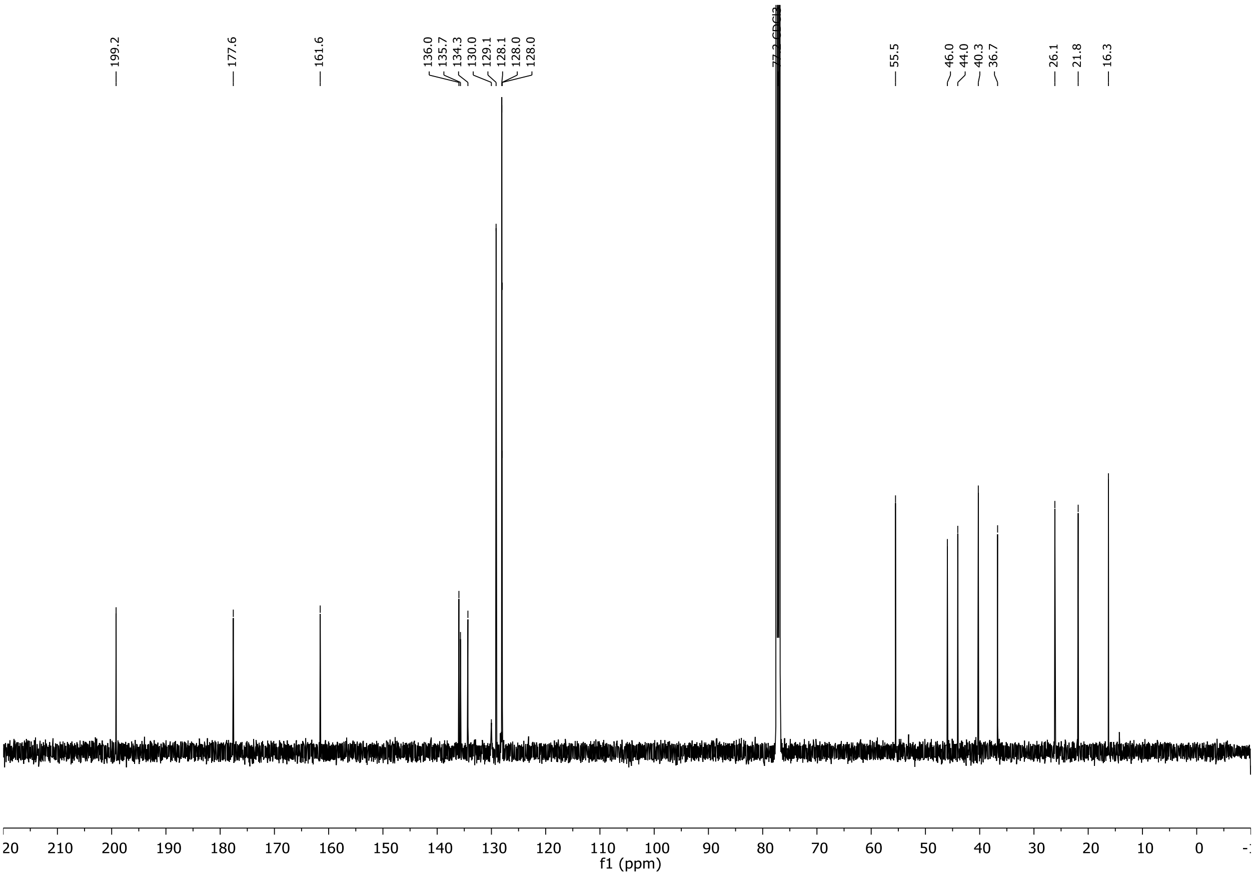

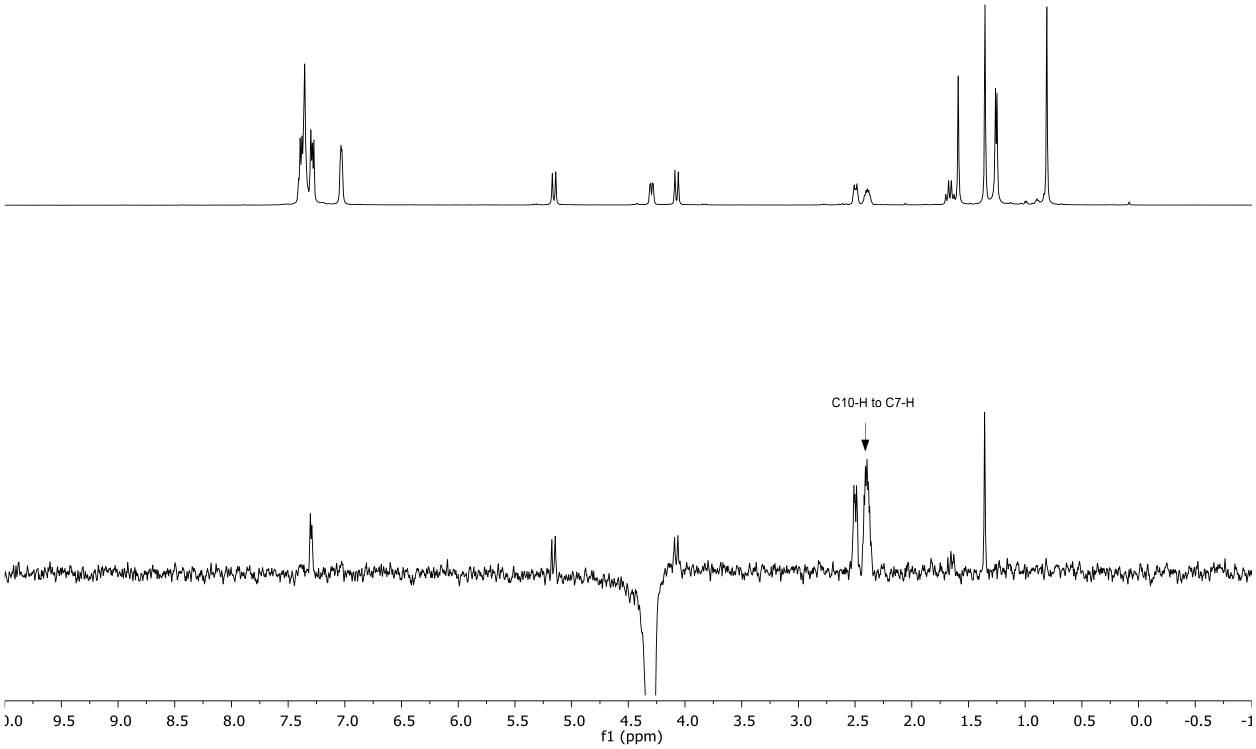
C10-H to C7-H
f1 (ppm)

_N_-Benzyl-_N_-((1_S_*,2_R_*)-2-butylcyclopropyl)-2,2-dimethyl-4-phenylbut-3-ynamide (3q)

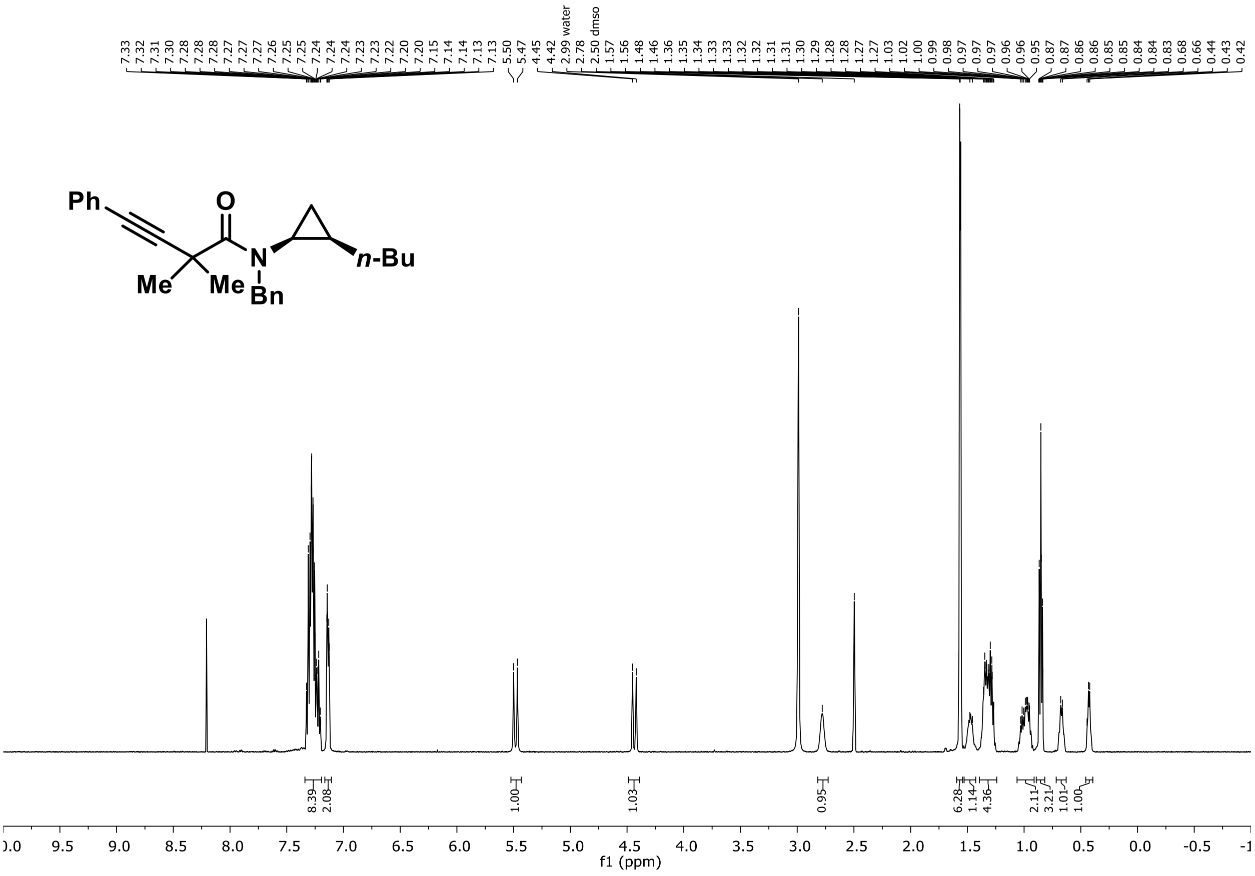

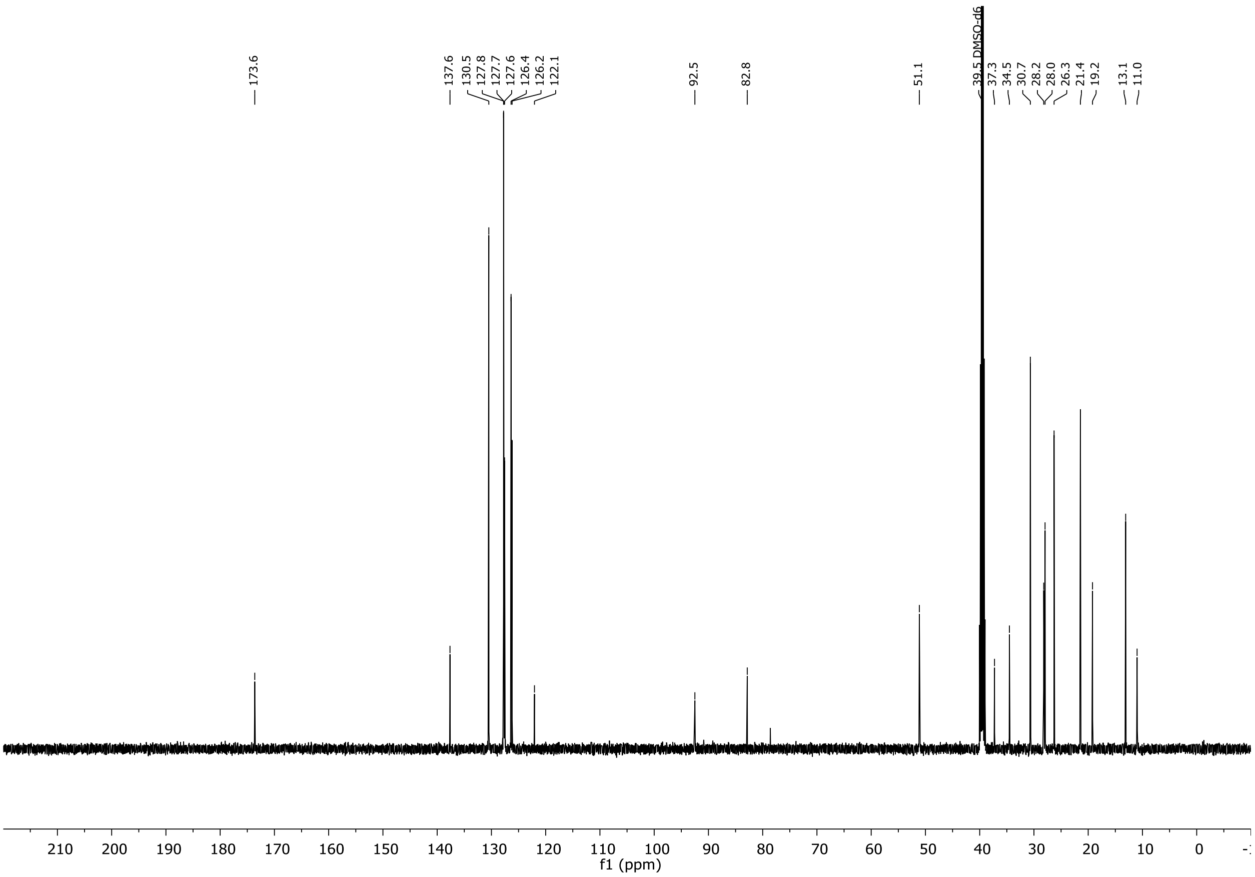

(6*S,7a*R**)-1-Benzyl-6-butyl-3,3-dimethyl-4-phenyl-1,6,7,7a-tetrahydro-2*H*-indole-2,5(3*H*)-dione (4q)**

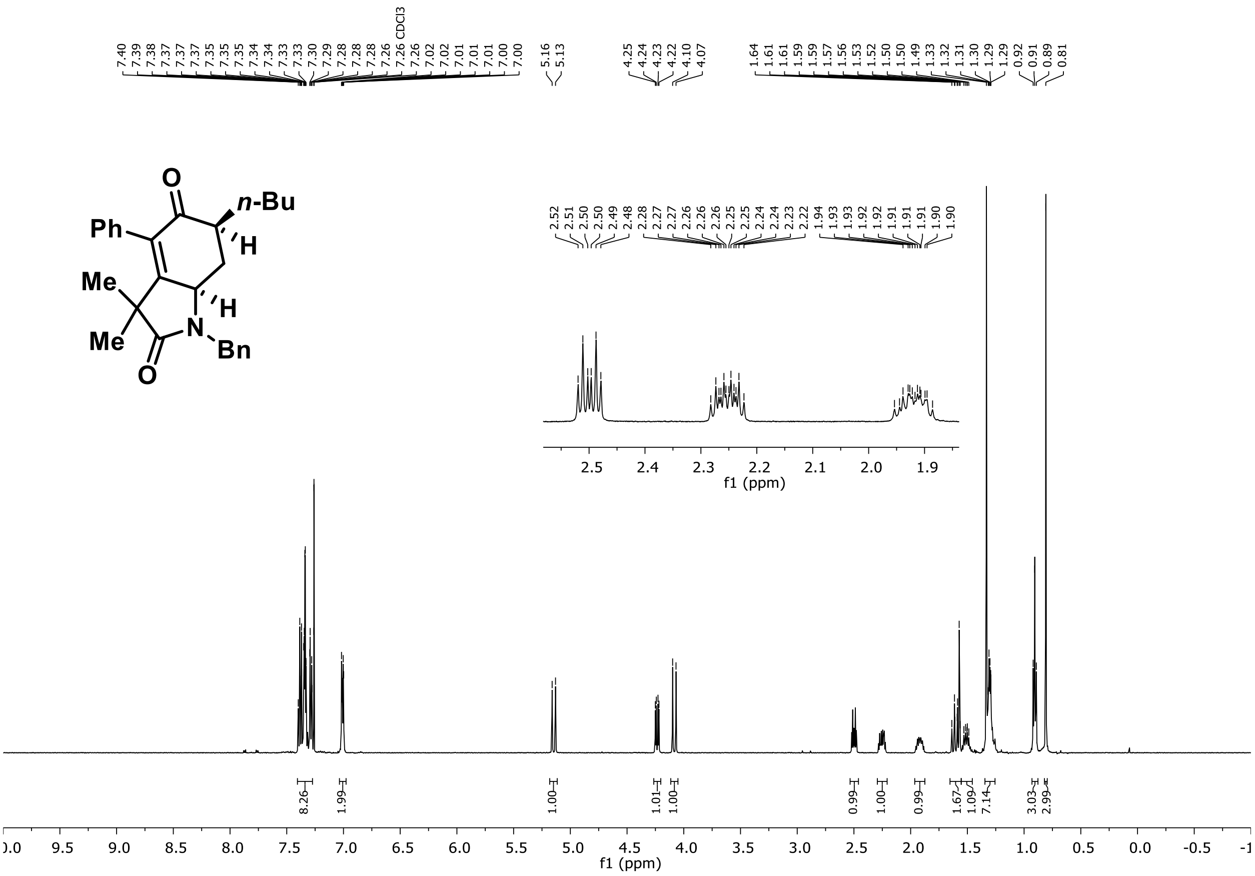

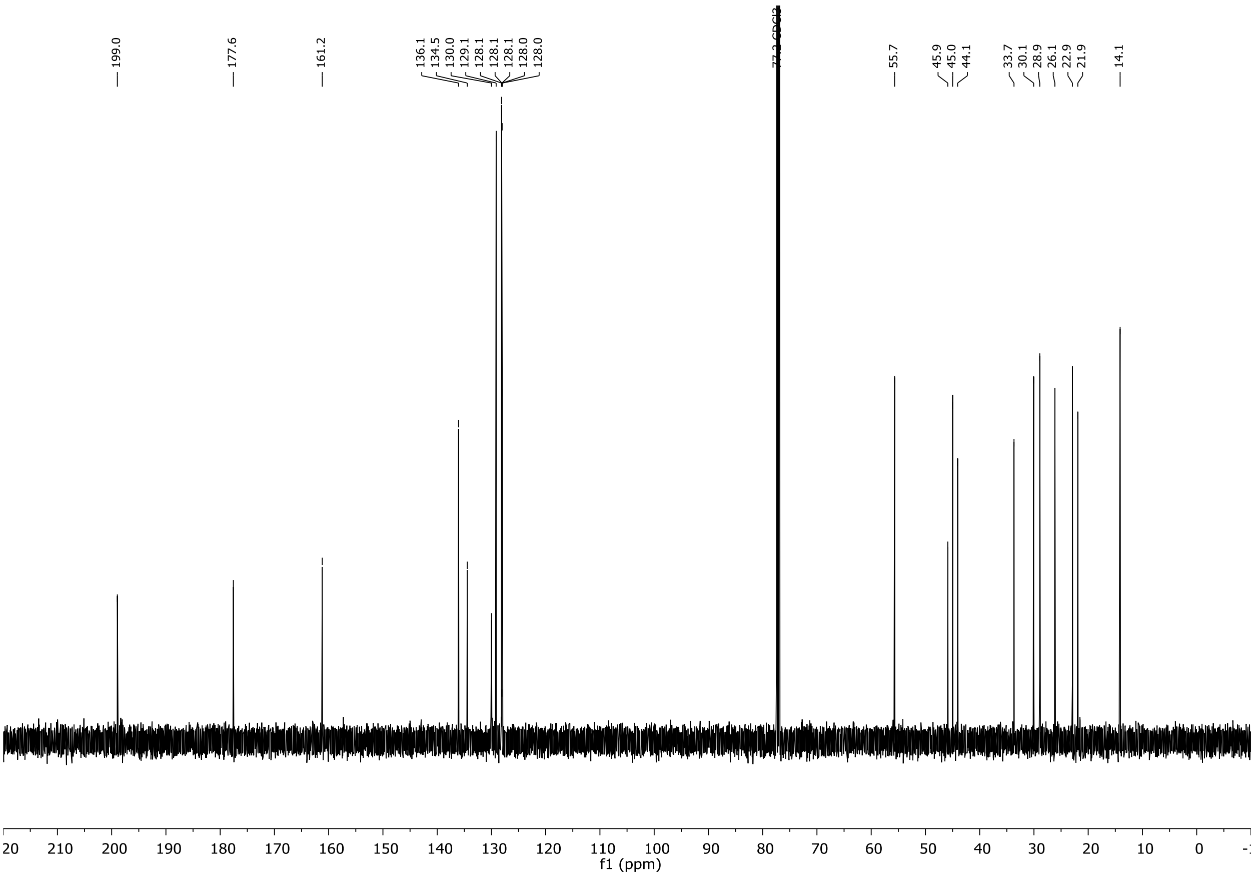

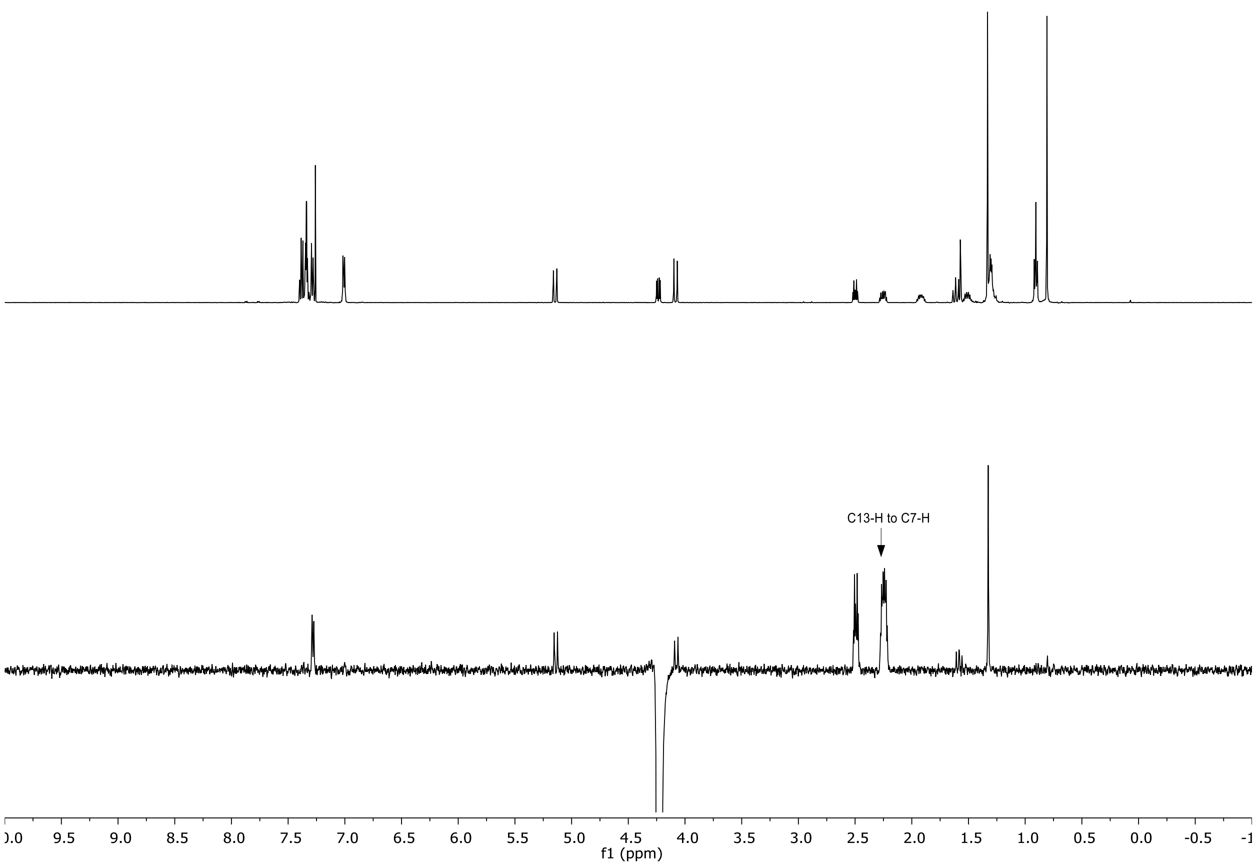
C13-H to C7-H
f1 (ppm)

_N_-Benzyl-_N_-((1_R_*,5_S_*,6_r_)-bicyclo[3.1.0]hexan-6-yl)-2,2-dimethyl-4-phenylbut-3-ynamide (3r)

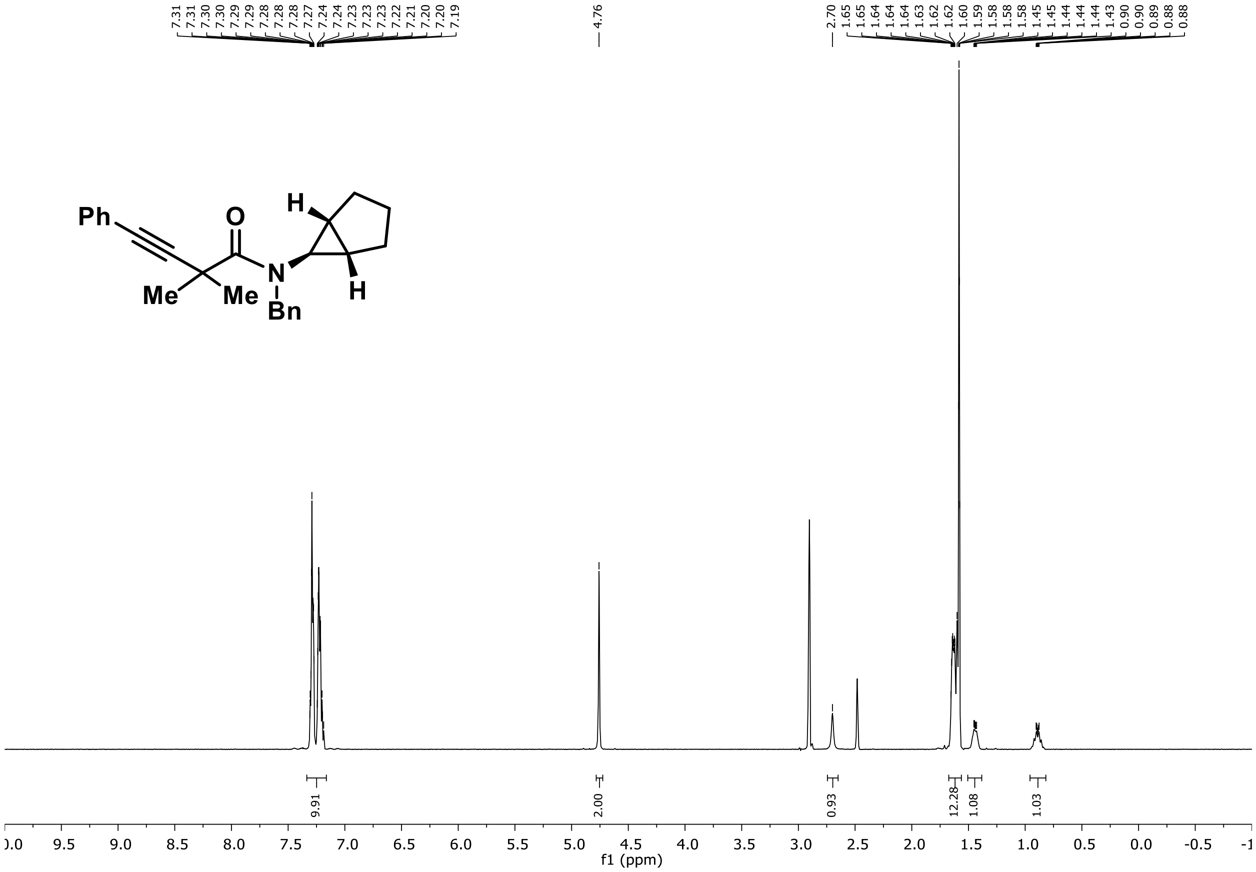

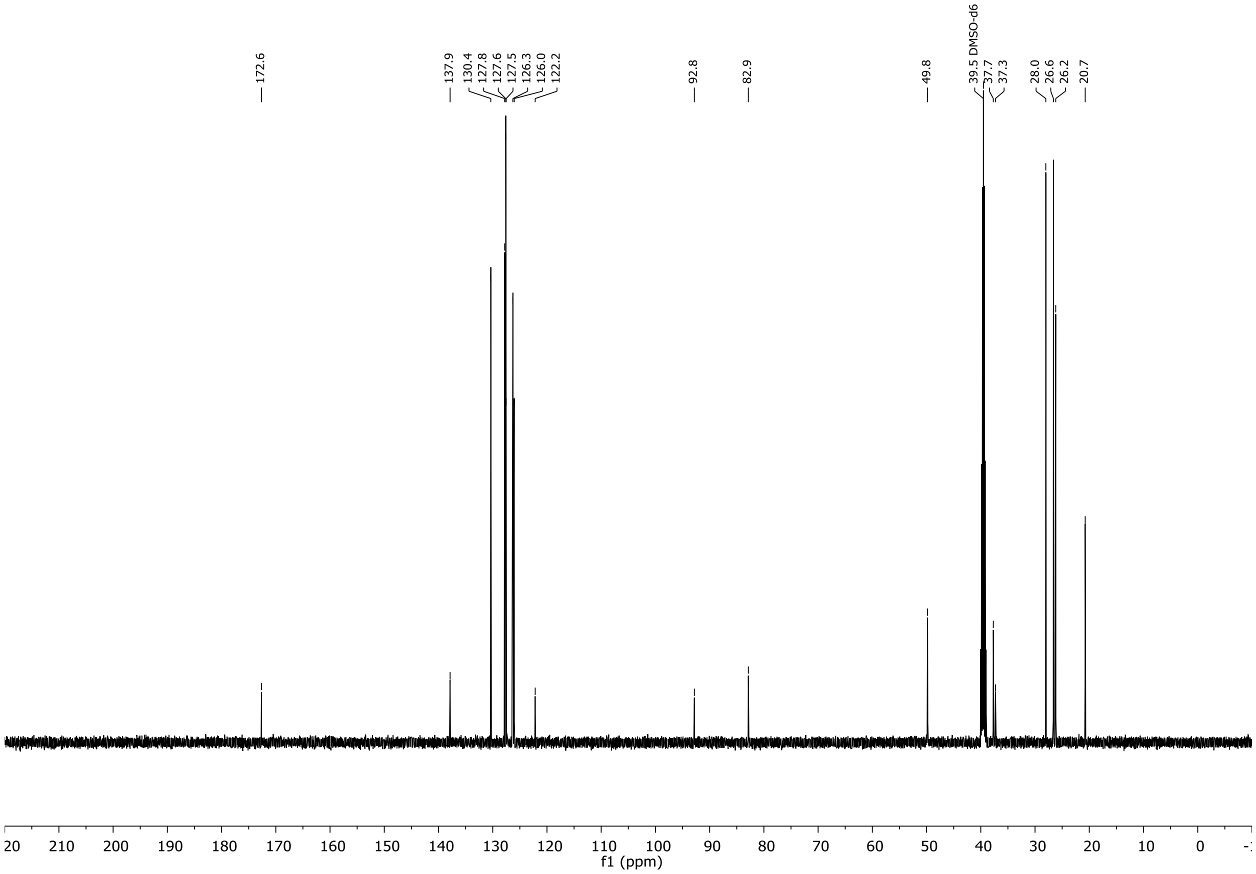

1-Benzyl-5a-hydroxy-3,3-dimethyl-4-phenyl-5a,6,7,8,8a,8b-hexahydrocyclopenta-[*g*]idole-2,5(1*H*,3*H*)-dione (4r')

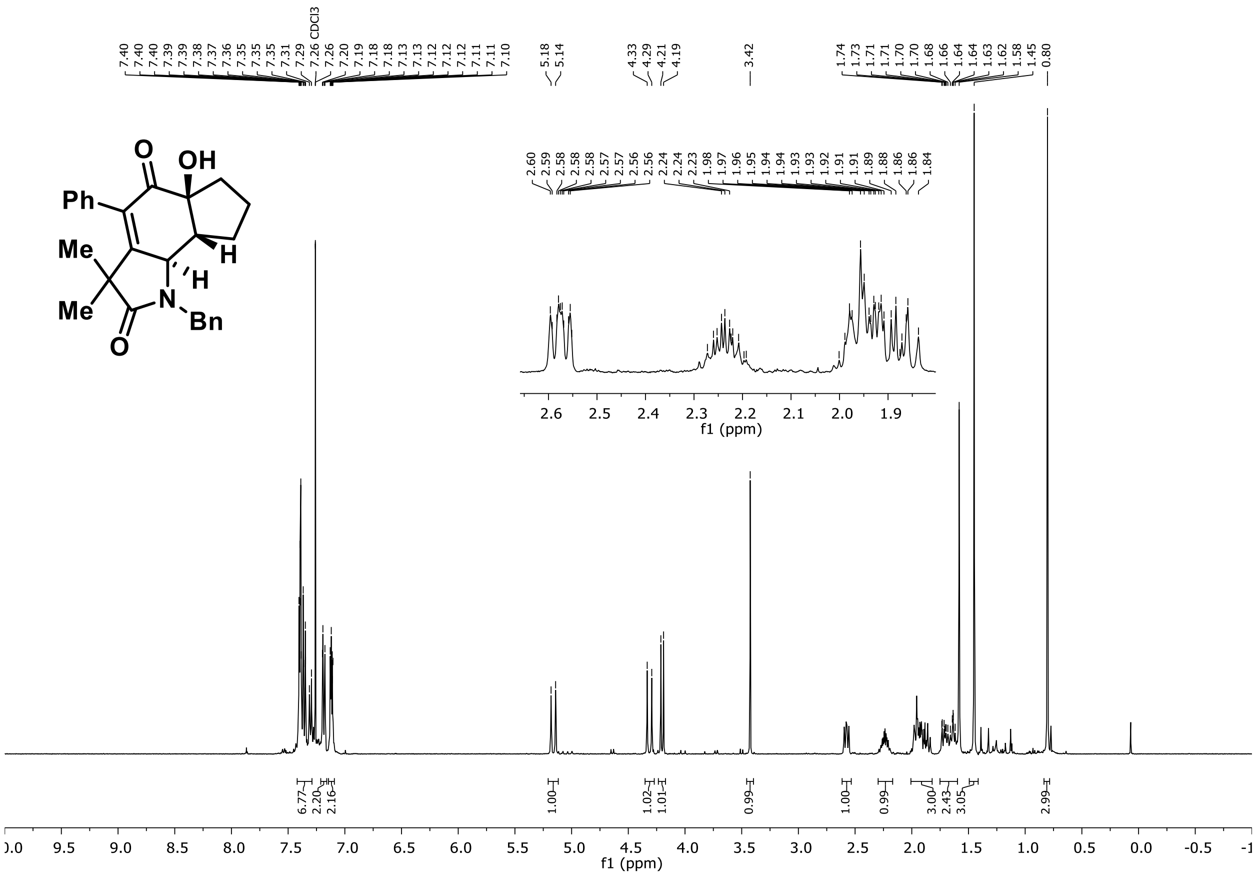

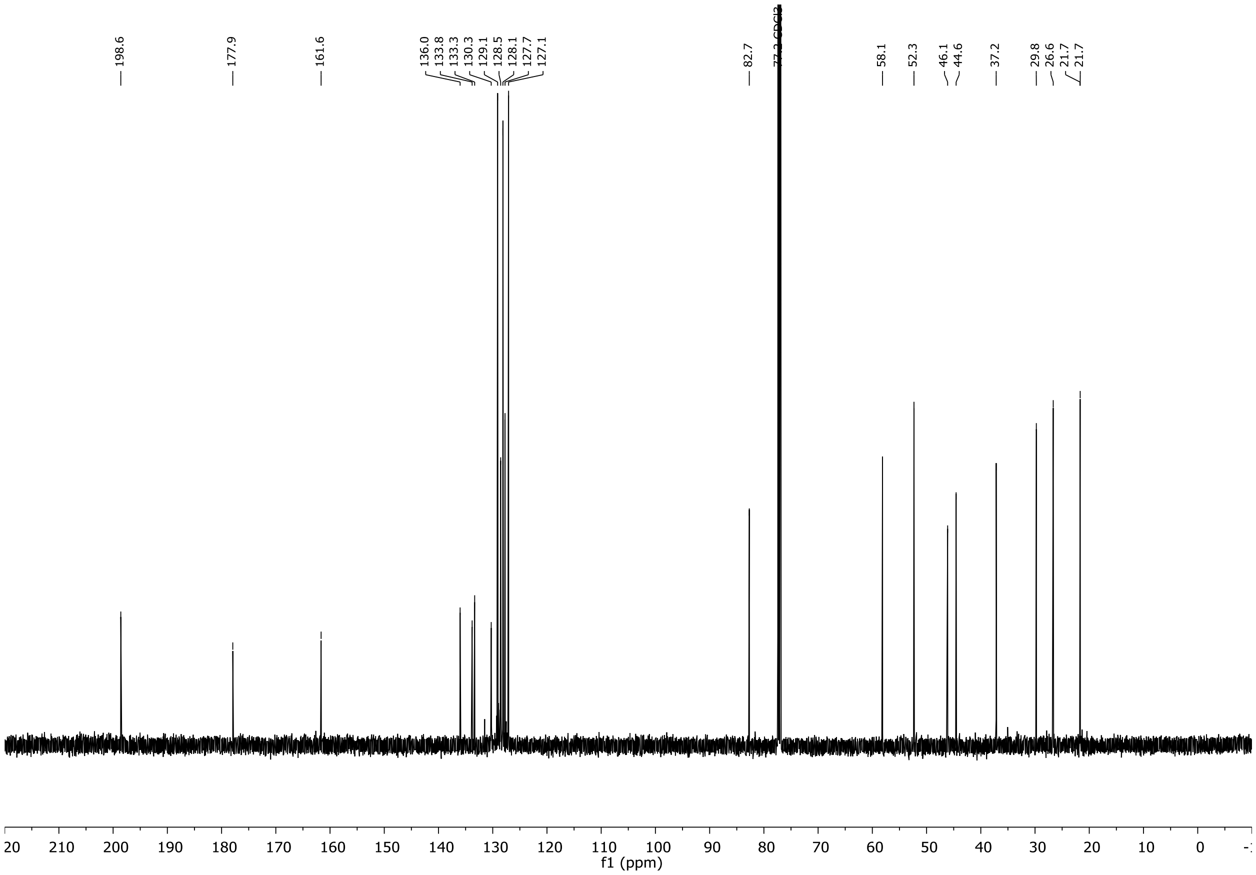

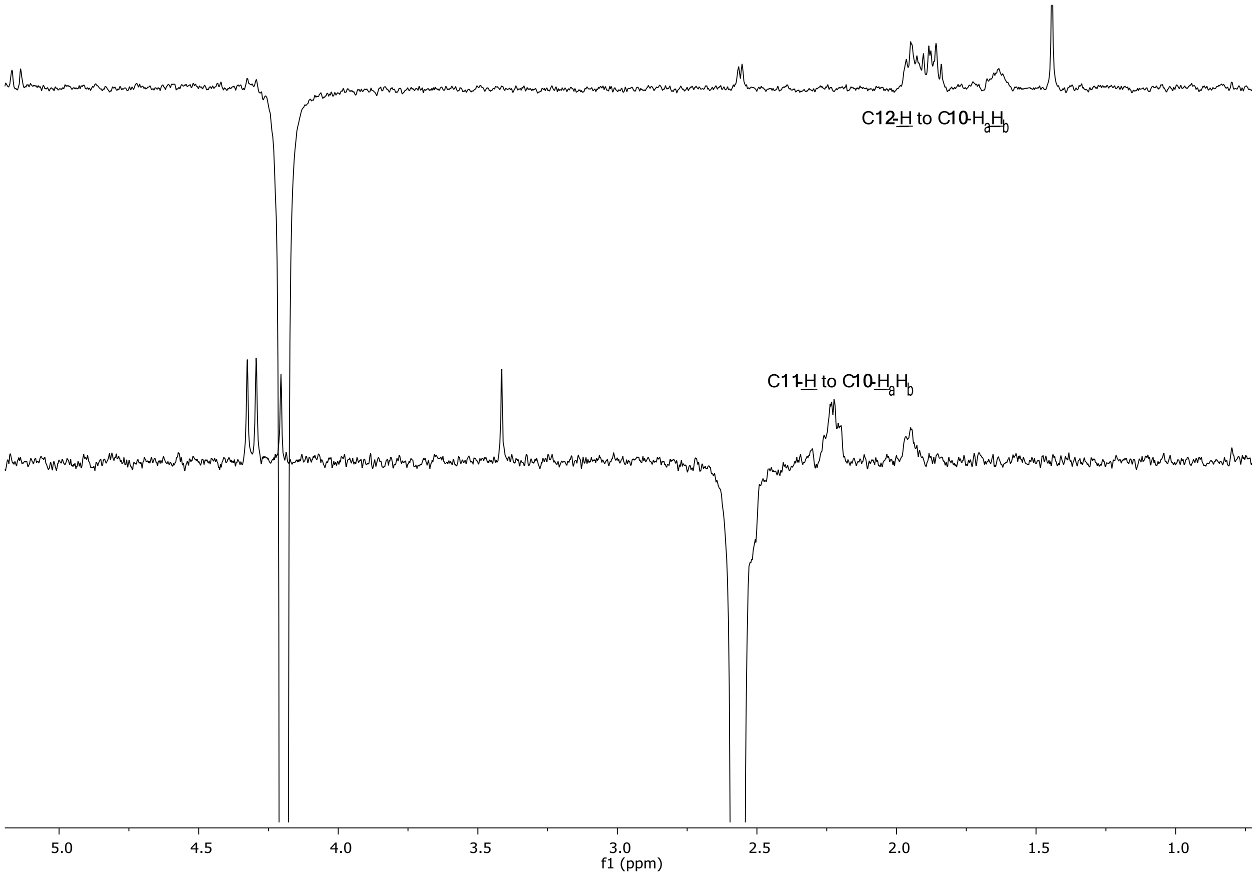
C12-H to C10-HaHb
C11-H to C10-HaHb
5.0
4.5
4.0
3.5
3.0
2.5
2.0
1.5
1.0
f1 (ppm)

***N*-Benzyl-*N*-cyclopropylpropiolamide**

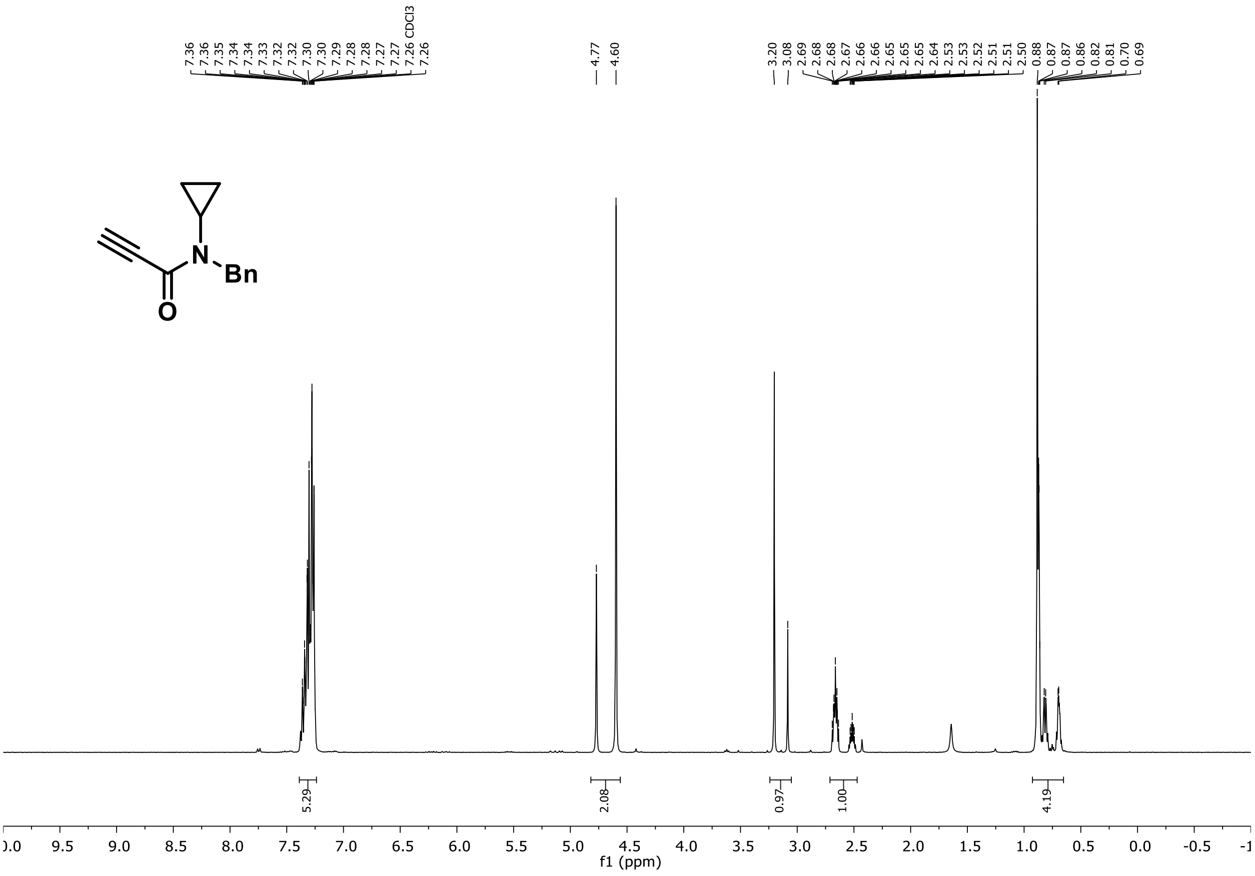

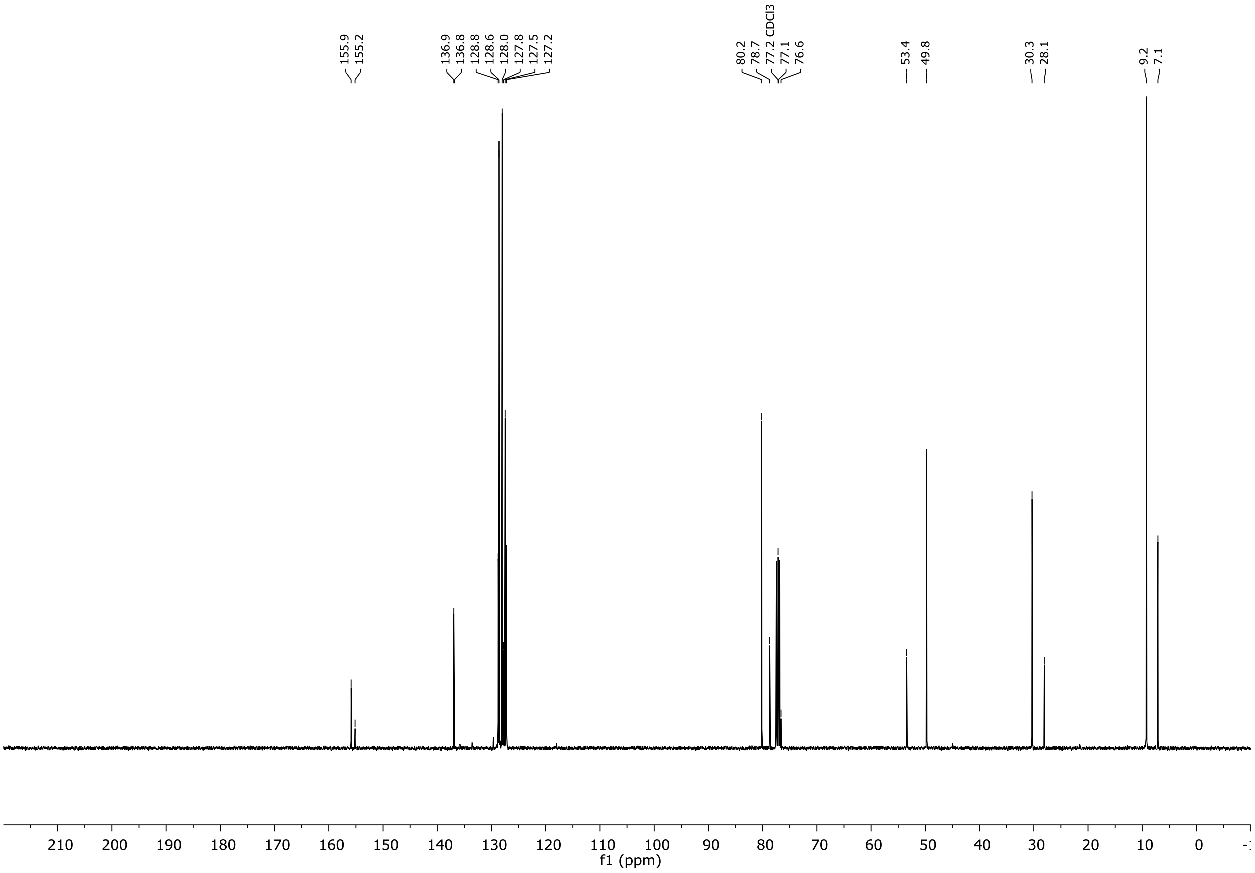

N-Benzyl-*N*-cyclopropyl-3-phenylpropiolamide

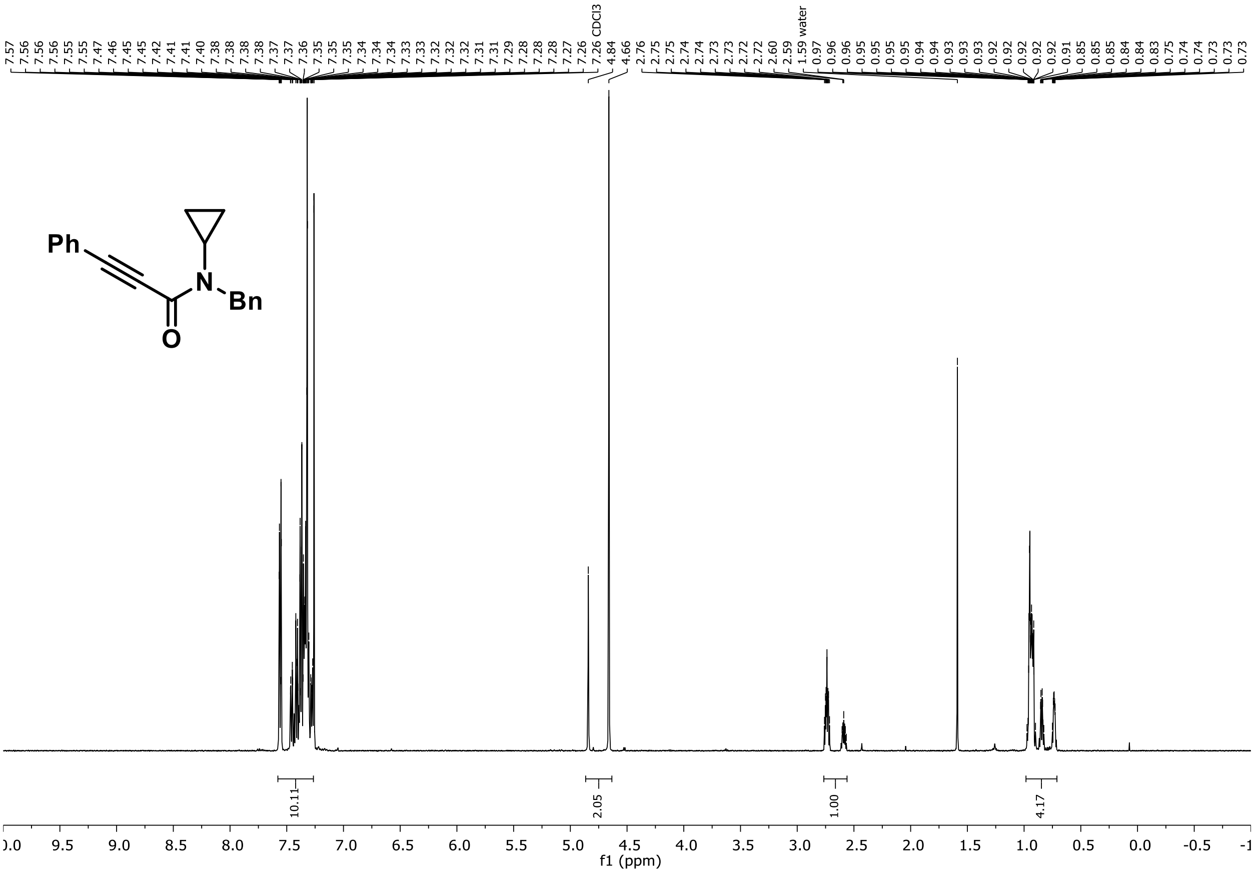

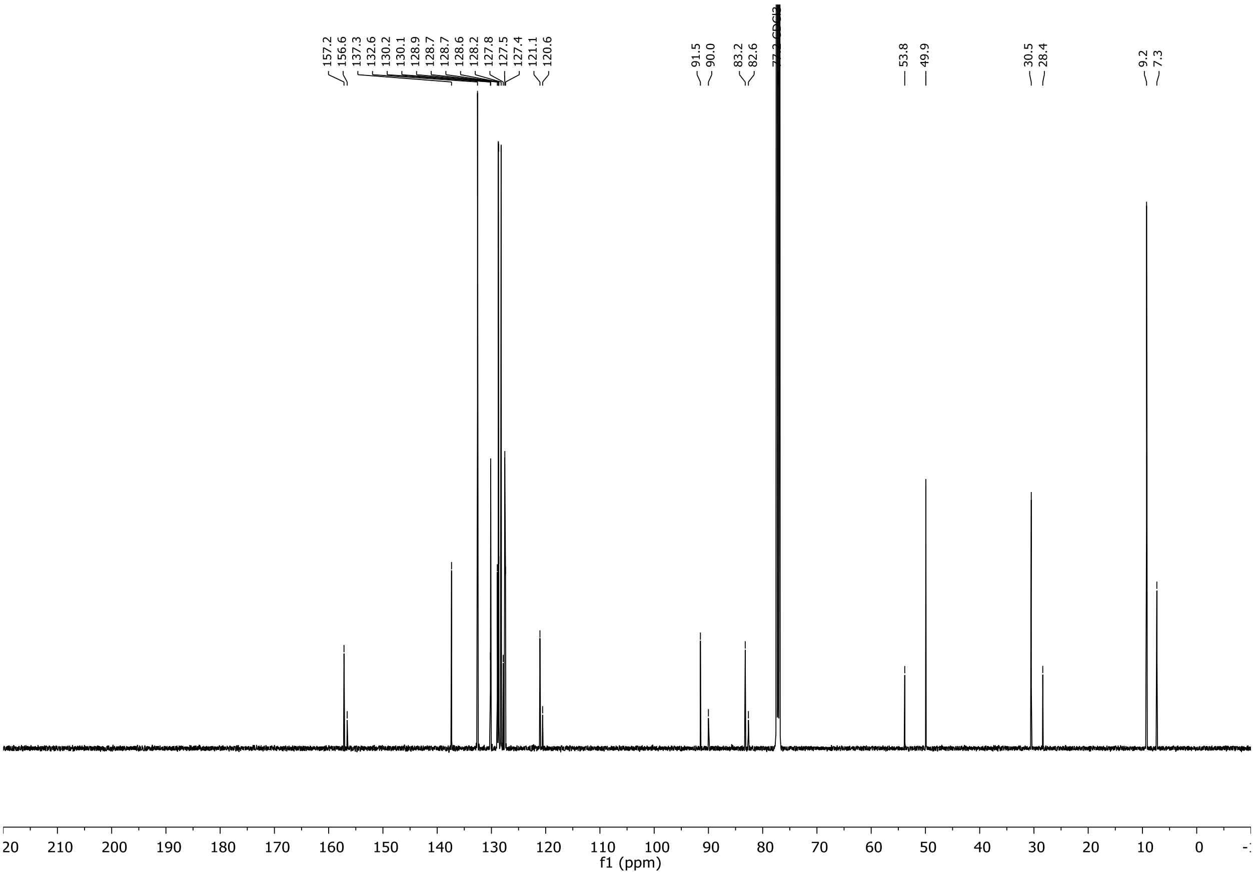

7-Benzyl-2-phenyl-7-azabicyclo[4.2.0]oct-1-ene-3,8-dione

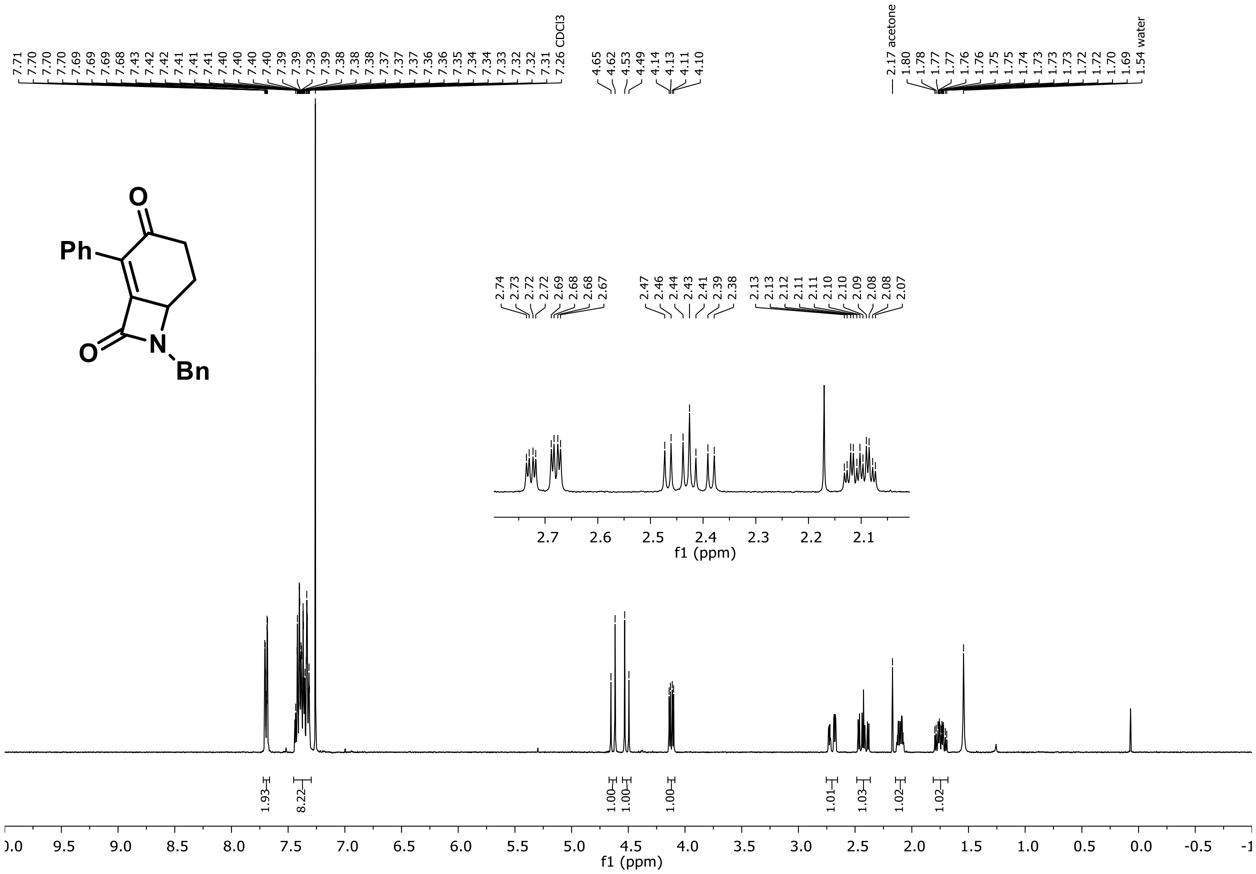

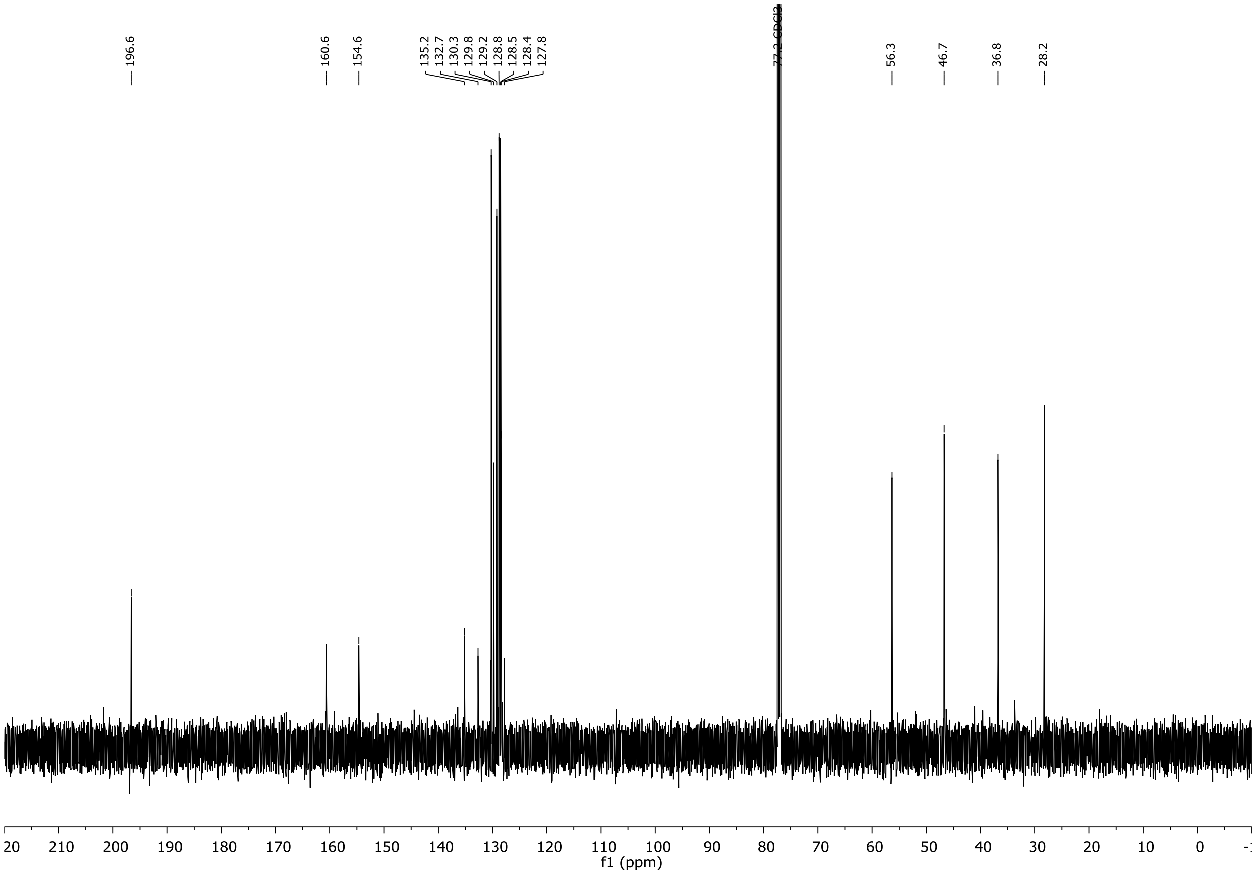

References

(1) Shaw, M. H.; Croft, R. A.; Whittingham, W. G.; Bower, J. F. *J. Am. Chem. Soc.* **2015**, *137*, 8054.

(2) Scheipers, I.; Mück-Lichtenfeld, C.; Studer, A. *Angew. Chem. Int. Ed.* **2019**, *58*, 6545.

(3) McCreanor, N. G.; Stanton, S.; Bower, J. F. *J. Am. Chem. Soc.* **2016**, *138*, 11465.

(4) Chen, G.-Q.; Zhang, X.-N.; Wei, Y.; Tang, X.-Y.; Shi, M. *Angew. Chem. Int. Ed.* **2014**, *53*, 8492.

(5) *TopiVert Pharma Ltd.* **2014**, *WO201433447 (A2)*.

(6) Dolomanov, O. V.; Bourhis, L. J.; Gildea, R. J.; Howard, J. A. K.; Puschmann, H. *J. Appl. Crystallogr.* **2009**, *42*, 339.

(7) Kozhushkov, S. I.; Wagner-Gillen, K.; Khlebnikov, A. F.; de Meijere, A. *Synthesis* **2010**, *2010*, 3967.

(8) Yonezawa, H.; Tashiro, S.; Shiraogawa, T.; Ehara, M.; Shimada, R.; Ozawa, T.; Shionoya, M. *J. Am. Chem. Soc.* **2018**, *140*, 16610.

(9) Shaw, M. H.; Melikhova, E. Y.; Kloer, D. P.; Whittingham, W. G.; Bower, J. F. *J. Am. Chem. Soc.* **2013**, *135*, 4992.

(10) Shaw, M. H.; McCreanor, N. G.; Whittingham, W. G.; Bower, J. F. *J. Am. Chem. Soc.* **2015**, *137*, 463.

(11) Delhaye, L.; Merschaert, A.; Delbeke, P.; Briône, W. *Org. Process Res. Dev.* **2007**, *11*, 689.

(12) McCreanor, N. G. *PhD Thesis* **2016**, University of Bristol.
